# Rhodium-catalysed vinyl 1,4-conjugate addition coupled with Sharpless asymmetric dihydroxylation in the synthesis of the CDE ring fragment of pectenotoxin-4†
†Electronic supplementary information (ESI) available: Synthetic procedures, compounds' characterisation data and NMR spectra. See DOI: 10.1039/c9sc01761e


**DOI:** 10.1039/c9sc01761e

**Published:** 2019-05-24

**Authors:** Melodie S. W. Richardson, Christopher J. Tame, Darren L. Poole, Timothy J. Donohoe

**Affiliations:** a Department of Chemistry , University of Oxford , Chemistry Research Laboratory , Mansfield Road , Oxford , OX1 3TA , UK . Email: timothy.donohoe@chem.ox.ac.uk; b GlaxoSmithKline Medicines Research Centre , Gunnels Wood Road , Stevenage , SG1 2NY , UK

## Abstract

Our synthesis of the CDE ring fragment of pectenotoxin-4 utilised two key steps to make the complex bicyclic ketal unit: (i) a rhodium-catalysed vinyl group 1,4-addition as the major C–C bond forming step; (ii) a stereoselective Sharpless Asymmetric Dihydroxylation (SAD) of the resulting 1,1-disubstituted homoallylic alcohol. Subsequent acid-catalysed cyclisation afforded the desired [5,6]-bicyclic ketal of the target molecule. This methodology was shown to be compatible with the desired E ring fragment **35** in order to construct the CDE fragment **37** of pectenotoxin-4.

## Abstract 2

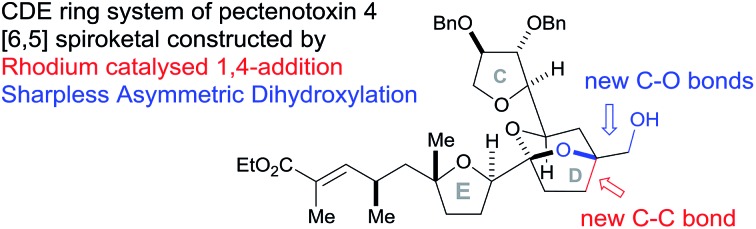
Rhodium and osmium catalysed C–C and C–O bond formation under mild conditions.

## Introduction

The pectenotoxins (PTXs) are a family of polyether macrolides containing a spiroketal (AB ring), three substituted tetrahydrofurans (C, E and F rings) and 19 or more stereocentres decorating the 40-carbon chain.^1^ These intriguing natural products were first isolated in 1985 by Yasumoto and coworkers,^2^ and have been shown to exhibit potent biological activity, including selective cytotoxicity against tumour cell lines.^3^

The architectural complexity of these highly functionalised macrolactones have garnered significant interest within the synthetic chemistry community,^4^ however only two total syntheses of these molecules have been completed to date: PTX-4 by Evans in 2002 (ref. 5) and PTX-2 by Fujiwara in 2014.^6^
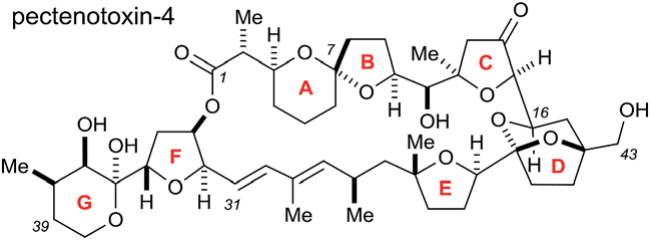


**Scheme 1:**
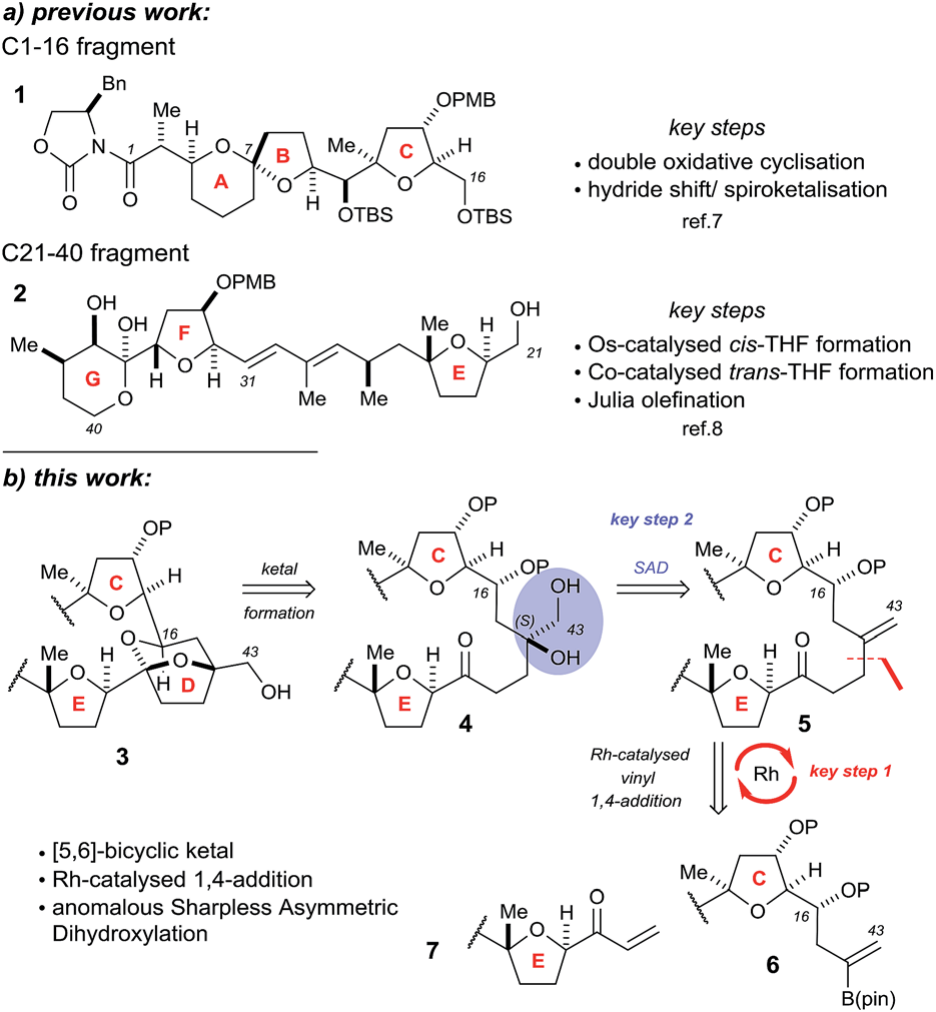
Previously synthesised fragments and key disconnections proposed in this work for the CDE fragments: (a) previous work; (b) this work.

The Donohoe group has made several significant advances towards the total synthesis of PTX-4. We have successfully synthesised the C-1 to C-16 ABC fragment *via* a double osmium catalyzed oxidative cyclisation together with a hydride-shift-initiated spiroketalisation,^7^ as well as preparing the C-21 to C-40 EFG fragment *via* stereodivergent catalytic cobalt and osmium oxidative cyclisations (Scheme 1a).^8^

The major challenge remaining in our synthesis of PTX-4 is uniting these two complex fragments to synthesise the final [5,6]-bicyclic ketal, the D ring. A handful of approaches to this bicyclic structure for PTX-2 (ref. 4*o*, *s*, *t*, *x*, *y*, *z* and 6) and PTX-4 (ref. 4*d*, *x* and 5) have been published in the literature. Herein, we propose a novel route which proceeds *via* an unusual rhodium-catalysed vinyl 1,4-addition^9^ as the key C–C bond formation step to join the ABC and E ring fragments. It is important to note that this key reaction has the potential to allow complex molecular fragments to be joined under relatively mild conditions and without using a large excess of either, extremely valuable, component. A subsequent stereoselective dihydroxylation–ketalisation sequence should then afford the desired [5,6]-bicyclic ketal structure of the D ring of PTX-4 (Scheme 1b). Note here that the sensitive diene containing FG ring fragment will be constructed as it is attached to the E-ring by a Julia reaction, after cyclisation of the D ring, because the 1,3-diene fragment itself would be unlikely to survive the conditions needed for dihydroxylation and/or cyclisation.

## Results and discussion

**Scheme 2:**
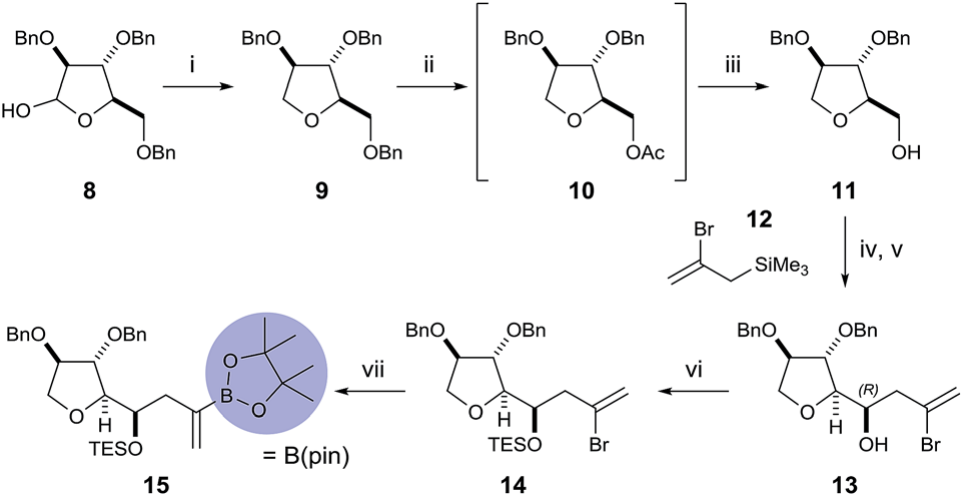
Synthesis of the model C ring boronate **15**. Reagents and conditions: (i) Et_3_SiH, BF_3_·OEt_2_, MeCN, 95%; (ii) Ac_2_O, TFA; (iii) NaOMe, MeOH, 89% over two steps; (iv) DMSO, SO_3_·py, Et_3_N, CH_2_Cl_2_, 0 °C; (v) **12**, BF_3_·OEt_2_, CH_2_Cl_2_, –78 °C, 56% over two steps; (vi) TESOTf, imidazole, CH_2_Cl_2_, 85%; (vii) (B(pin))_2_, PdCl_2_(PPh_3_)_2_ (3 mol%), PPh_3_ (6 mol%), KOPh, PhMe, 50 °C, 79%.

To begin, we chose to use model C ring boronate **15** as a substitute for the real ABC ring fragment required in the synthesis of PTX-4 (Scheme 2). Starting from commercially available enantiopure furanose **8**, the hemiacetal was reduced to the corresponding THF **9**, and the primary benzyl group removed in two steps, *via* acetate **10**, to reveal **11**.^10^ Oxidation of the primary alcohol to the aldehyde followed by a Hosomi–Sakurai reaction^11^ with bromoallylsilane **12** (ref. 12) afforded the (*R*)-homoallylic alcohol **13** as a single diastereoisomer in 56% yield over two steps. The stereochemistry arises from Felkin–Ahn-controlled addition of the bromoallylsilane **12** and was confirmed *via* Mosher's ester analysis.^13^ Direct conversion of the bromide to the desired boronate was unsuccessful; therefore protection of the secondary alcohol **13** with TESOTf was necessary. Miyaura borylation of TES-protected bromide **14** to the model C ring boronate **15** was then accomplished in 79% yield.^14^

**Scheme 3:**
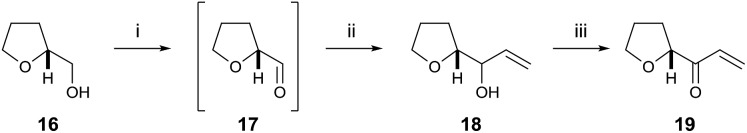
Synthesis of the model E ring enone **19**. Reagents and conditions: (i) DMSO, (COCl)_2_, Et_3_N, CH_2_Cl_2_, –78 °C; (ii) vinyl magnesium bromide, Et_2_O, –78 °C, 35% over two steps, 1.15 : 1 dr; (iii) DMP, CH_2_Cl_2_, 89%.

Similarly, we started with a less substituted THF ring in place of the desired E ring fragment in our initial studies (Scheme 3). Therefore, (*R*)-tetrahydrofurfuryl alcohol **16** was oxidised to the corresponding aldehyde **17**, and vinyl Grignard reagent was added to afford volatile allyl alcohol **18** in 35% yield (1.15 : 1 dr at the hydroxyl centre) over two steps. Oxidation using DMP furnished the model E ring enone **19** in 89% yield.

**Scheme 4:**
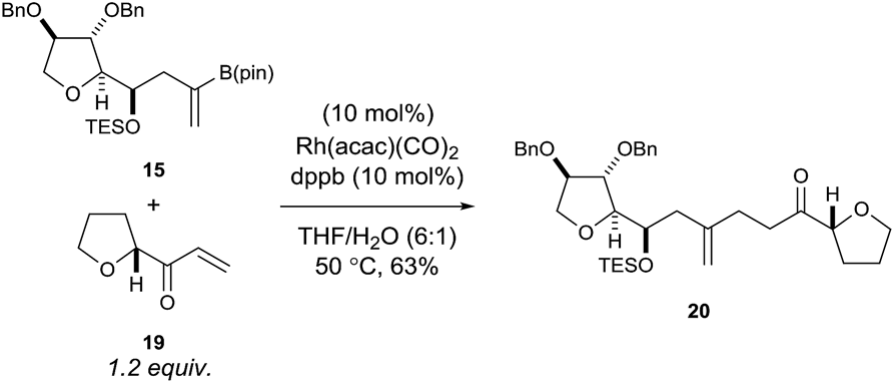
Rhodium-catalysed 1,4-addition reaction of model C ring boronate **15** with model E ring enone **19**.

Using rhodium-catalysed 1,4-addition conditions^9^ on model C ring boronate **15** with **19** was successful and afforded the desired adduct **20** in approximately 40% yield. However, the use of methanol as the solvent formed the methanol 1,4-addition adduct of compound **19** as a by-product, which often co-eluted with the desired products. Pleasingly, we found that replacing methanol with THF as the solvent prevented the formation of this by-product and improved the yield to 63% for the reaction between **15** and **19** (Scheme 4).

In order to ensure that no epimerisation had taken place adjacent to the ketone carbonyl, we repeated the coupling between **15** and racemic **19** (compound **S6** prepared separately, see ESI† for details). This reaction gave two diastereoisomeric compounds (in an approximately 1 : 1 ratio) and from the ^13^C NMR spectrum of this mixture we could rule out epimerisation in compound **20** formed from enantiopure **19**.

**Scheme 5:**
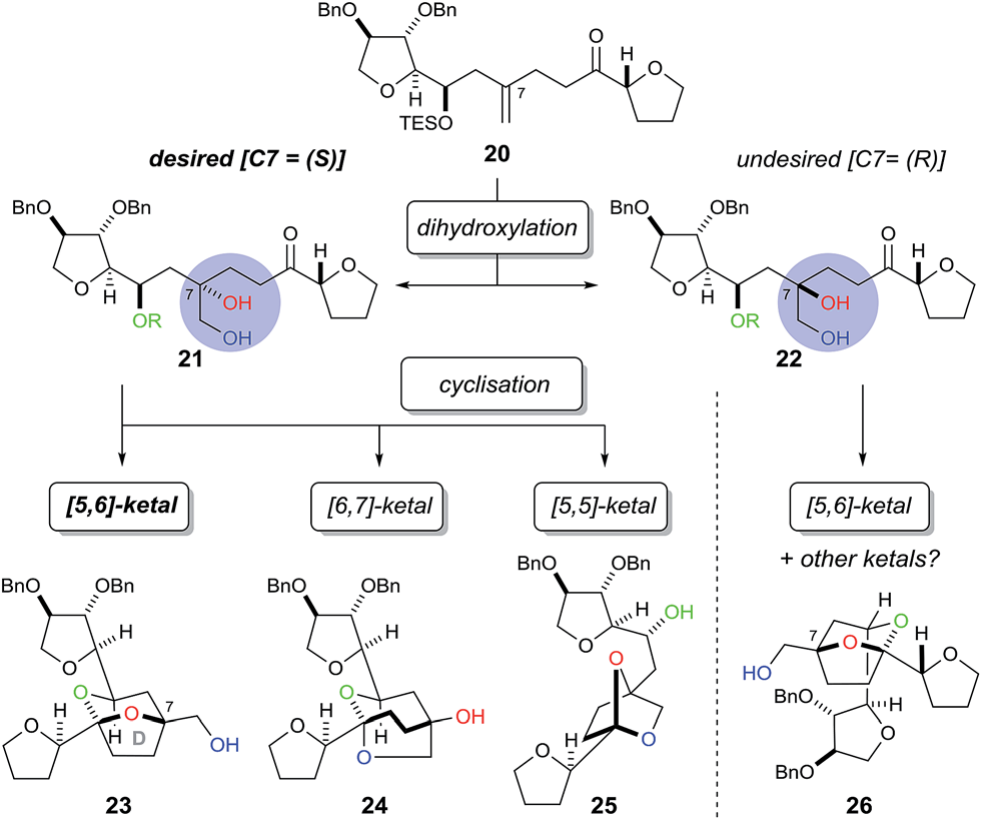
Possible bicyclic ketal structures arising from diols **21** and **22**.

In order to construct the bicyclic acetal D-ring system we next required a facially selective dihydroxylation of the alkene within **20** (to set the stereochemistry at C7, Scheme 5) followed by a ketalisation reaction. Although the stereochemical outcome of dihydroxylation of 1,1-disubstituted alkenes are difficult to predict,^15^ we chose to use the Sharpless Asymmetric Dihydroxylation (SAD) to control diol formation. It is worth noting that the original mnemonic proposed by Sharpless for the SAD reaction^16^ is often problematic when applied to 1,1-disubstituted alkenes, as first shown by Hale.15*a* In the case of substrate **20**, even if we could achieve near “perfect” facial selectivity for the correct diol **21**, acid-catalysed cyclisation could then result in three possible isomeric bicyclic ketal structures: the desired [5,6]-ketal **23**, [6,7]-ketal **24** and [5,5]-ketal **25** (Scheme 5).

At first the dihydroxylation of 1,1-disubstituted alkene **20** was attempted using Upjohn conditions^17^ to reveal any substrate bias during oxidation, however purification and identification of the desired diol was difficult as a complex mixture of products were obtained, possibly due to TES group migration. It was proposed to cyclise the crude diol using mildly acidic conditions5*b* while also removing the TES group; however the use of this procedure still produced in a complex mixture.

**Scheme 6:**
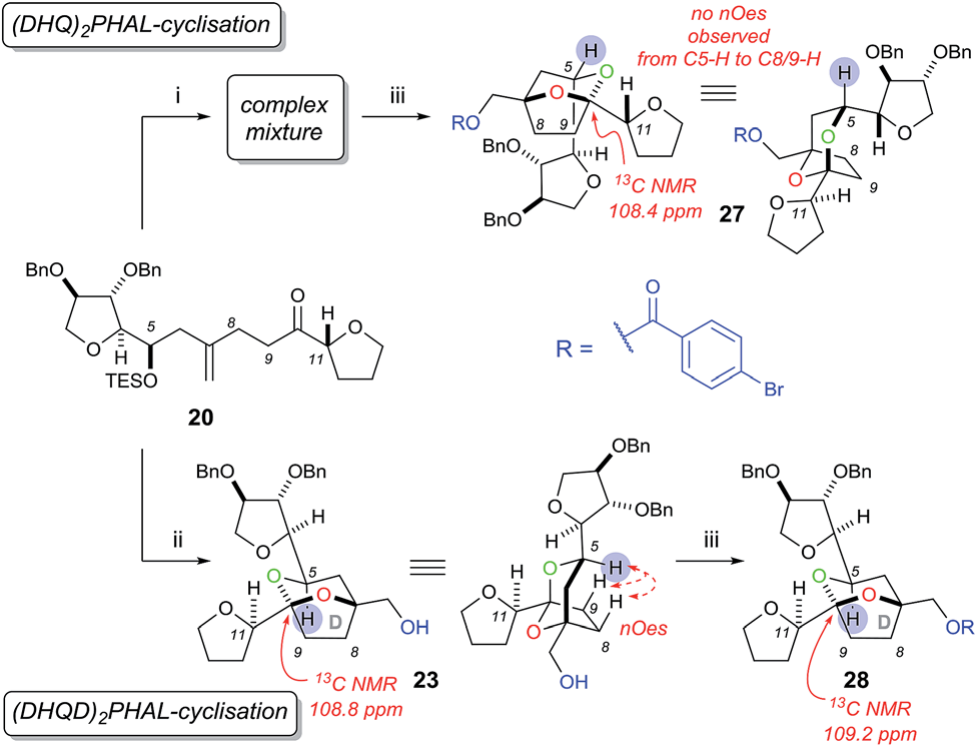
Sharpless asymmetric dihydroxylation and acid-catalysed cyclisation of **20**. Reagents and conditions: (i) K_2_OsO_2_(OH)_4_, (DHQ)_2_PHAL, K_3_Fe(CN)_6_, K_2_CO_3_, MeSO_2_NH_2_, *t*-BuOH/H_2_O (1 : 1), 0 °C then PPTS, CH_2_Cl_2_/MeOH (1 : 1); (ii) K_2_OsO_2_(OH)_4_, (DHQD)_2_PHAL, K_3_Fe(CN)_6_, K_2_CO_3_, MeSO_2_NH_2_, *t*-BuOH/H_2_O (1 : 1), 0 °C then PPTS, CH_2_Cl_2_/MeOH (1 : 1), 91%; (iii) 4-bromobenzoic acid, DIC, DMAP, CH_2_Cl_2_, 5% over two steps for **27**, 53% for **28**.

Undeterred, we chose the Sharpless Asymmetric Dihydroxylation (SAD) conditions to obtain the desired diol stereochemistry. As it is difficult to predict which ligand is required, we used both (DHQ)_2_PHAL and (DHQD)_2_PHAL separately. According to the mnemonic,^16^ we predicted (DHQ)_2_PHAL would produce the desired diol. However, using (DHQ)_2_PHAL ligand in the dihydroxylation and acid-induced cyclisation sequence produced a mixture of products (Scheme 6). Nevertheless, upon derivatisation of the mixture with 4-bromobenzoic acid we identified [5,6]-bicyclic ketal **27**, with a characteristic ^13^C NMR peak at 108.4 ppm.4*d* The connectivity, supported by COSY/HSQC/HMBC experiments, was shown to be the [5,6]-ketal over the [6,7] or [5,5] isomers. Moreover, the relative stereochemistry of dihydroxylation could also be assigned as shown, because within the [5,6]-ketal structure we did not observe an nOe enhancement across the ring system (*i.e.* between C-5 to either C-8 or C-9); this would be expected in the desired ketal structure. Looking at the full set of data we concluded that compound **27** contained the [5,6]-bicyclic ketal but with the opposite stereochemistry at C-7 (as set by the initial dihydroxylation).

Interestingly, the use of (DHQD)_2_PHAL in the dihydroxylation–cyclisation sequence (oxidation being followed by treatment with acid) also provided one bicyclic ketal diastereoisomer **23** with a ^13^C NMR peak at 108.8 ppm (Scheme 6). Upon detailed NMR (COSY/HSQC/HMBC) analysis, **23** was again confirmed to have the [5,6]-bicyclic ketal connectivity. However, this time the molecule did exhibit key nOe enhancements across the bicyclic ring (C-5 to C-8 and C-9), showing it to be the desired [5,6]-bicyclic ketal **23** originating from the correct stereochemistry at C7. Further derivatisation of **23** with 4-bromobenzoic acid gave compound **28** which was different to the related ketal (**27**) formed from the (DHQ)_2_PHAL derived experiments.

We note that other bicyclic ketals ([6,7] and [5,5]) were not isolated in any reaction, however there have been reports that these types of structures may undergo facile degradation upon purification and may not be isolatable.4*d* Our studies show that in this system it is the (DHQD)_2_PHAL ligand that delivers the correct facial selectivity during dihydroxylation, and that acid-catalysed ketalisation then forms the desired [5,6]-ketal system as found in the natural product. The fact that (DHQD)_2_PHAL has formed the (*S*)-diol **21** during dihydroxylation is consistent with the reversed stereoselectivity that has been reported during the SAD reaction of 1,1-disubstituted alkenes.15a–e,i

**Scheme 7:**
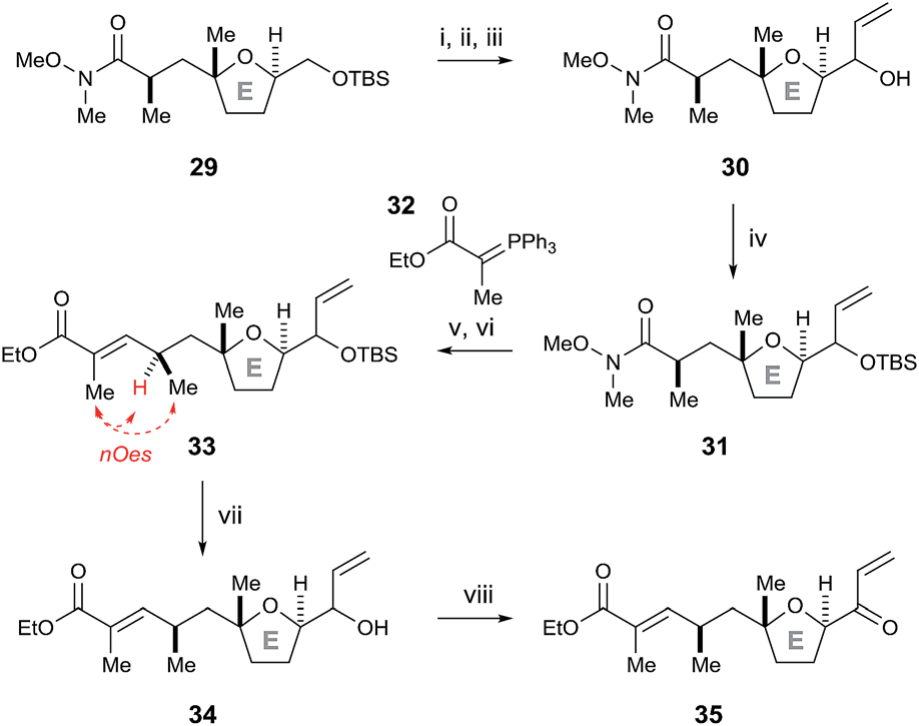
Synthesis of the E ring enone **35**. Reagents and conditions: (i) TBAF, THF, 0 °C, 96%; (ii) SO_3_·py, Et_3_N, DMSO, CH_2_Cl_2_, 0 °C; (iii) vinyl magnesium bromide, Et_2_O, 0 °C, 69% over two steps; (iv) TBSCl, imidazole, DMAP, CH_2_Cl_2_, 88%; (v) DIBAL-H, THF, –78 °C; (vi) **32**, benzene, Δ, 87% over two steps; (vii) TBAF, THF, 78%; (viii) DMP, NaHCO_3_, CH_2_Cl_2_, 90%.

To further test this methodology in the synthesis of pectenotoxin-4, we converted the desired E ring fragment **29** (ref. 8) into the desired enone **35**, with the unsaturated ethyl ester side chain serving as a precursor for a Julia olefination coupling with the FG ring fragment. Therefore, the previously reported E fragment enantiopure **29** (ref. 8) was deprotected with TBAF, before Parikh–Doering oxidation to the aldehyde and vinyl Grignard reagent was added to afford allyl alcohol **30** (Scheme 7). The hydroxyl group was protected with TBSCl, before the Weinreb amide was reduced to the aldehyde with DIBAL-H and a Horner–Wadsworth–Emmons reaction with ylide **32** furnished (*E*)-unsaturated ethyl ester **33** (stereochemistry proven by nOe analysis). Finally, removal of the TBS group with TBAF followed by oxidation with DMP afforded the E ring enone **35**.

**Scheme 8:**
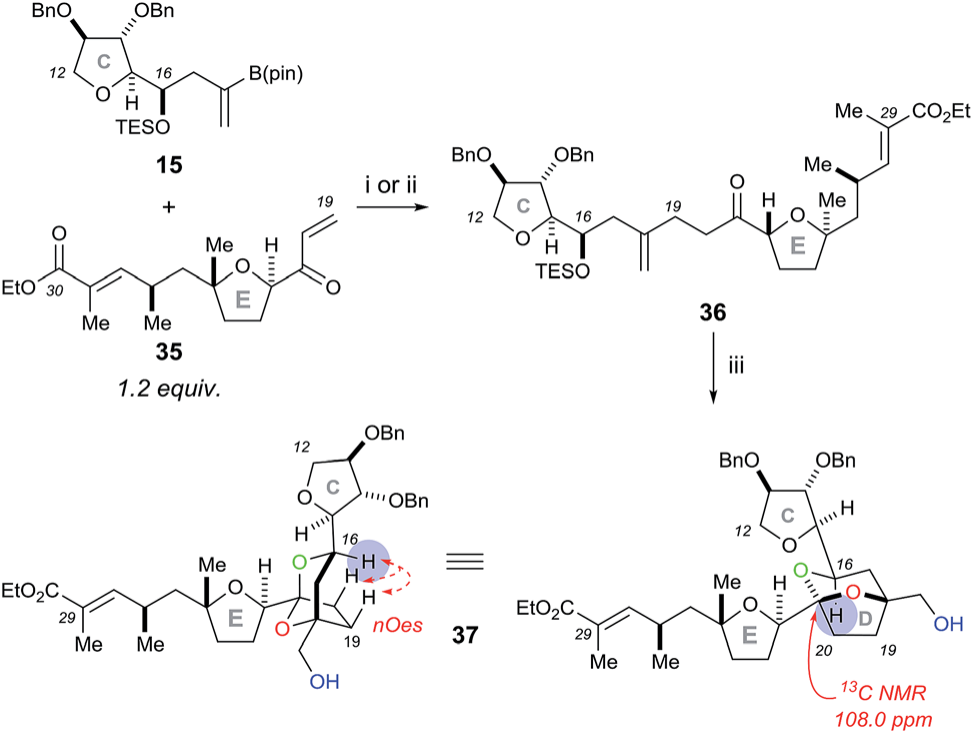
Rhodium-catalysed 1,4-addition reaction of the model C ring boronate **15** with E ring enone **35**, followed by the Sharpless asymmetric dihydroxylation and acid-catalysed cyclisation sequence to access the CDE fragment **37**. Reagents and conditions: (i) Rh(acac)(CO)_2_ (10 mol%), dppb (10 mol%), THF/H_2_O (6 : 1), 50 °C, 30%; (ii) [Rh(cod)OH]_2_ (15 mol%), THF/H_2_O (6 : 1), 50 °C, 53%; (iii) K_2_OsO_2_(OH)_4_, (DHQD)_2_PHAL, K_3_Fe(CN)_6_, K_2_CO_3_, *t*-BuOH/H_2_O (1 : 1), 0 °C then PPTS, CH_2_Cl_2_/MeOH (1 : 1), 40%.

Initial rhodium-catalysed 1,4-addition reaction conditions between model C ring boronate **15** and E ring enone **35** were moderately successful, affording adduct **36** in 30% yield (Scheme 8). Repeating the reaction with a more active catalyst system, [Rh(cod)OH]_2_,^18^ improved the yield of **36** to 53%. Pleasingly, the dihydroxylation–cyclisation sequence (using (DHQD)_2_PHAL ligand) then afforded the desired CDE fragment **37** in 40% yield, as a single compound, with a characteristic ^13^C NMR peak at 108.0 ppm. Other ligands tested in the osmium catalyzed dihydroxylation of **36**, such as (DHQD)_2_PYR and (DHQD)_2_AQN, did not improve the yield. It should be noted that the omission of methanesulfonamide and careful monitoring of the reaction progress was required to avoid over-oxidation of the ethyl ester substituted alkene. Moreover, the structure of **37** was confirmed with COSY/HSQC/HMBC NMR experiments to be the desired [5,6]-ketal and the stereochemistry was then assigned by the nOes observed across the bicyclic ring system (C-16 to C-19 and C-20) as was the case for compound **23**. In this case, experiments performed to dihydroxylate and cyclise **36** using the opposite chiral ligand (*i.e.* (DHQ)_2_PHAL) only resulted in the formation of a complex mixture of products.

## Conclusions

In conclusion, we have developed a novel route to the CDE fragment (C-12 to C-30) of PTX-4. The key C–C bond forming step was a rhodium catalysed 1,4-vinyl group addition to an enone which used a close to equimolar ratio of the two key components. Model studies revealed a reversal of ligand-facial selectivity during the SAD reaction of a 1,1-disubstituted homoallylic alcohol, resulting in the isolation of two different [5,6]-bicyclic ketals depending on the chiral ligand used. This methodology was then extended to incorporate the desired E ring fragment of PTX-4 in the synthesis of a CDE fragment of PTX-4. Further work is ongoing to utilise this methodology and complete the total synthesis of PTX-4.

## Conflicts of interest

There are no conflicts to declare.

## Supplementary Material

Supplementary information
