# Supplementary material for: Rhodium-catalysed vinyl 1,4-conjugate addition coupled with Sharpless asymmetric dihydroxylation in the synthesis of the CDE ring fragment of pectenotoxin-4
Source: Chem Sci. 2019 May 24;10(25):6336–40. doi: 10.1039/c9sc01761e (PMC6601420; doi:10.1039/c9sc01761e)
Supplement: Supplementary file 1 [file SC-010-C9SC01761E-s001.pdf]

Electronic Supplementary Material (ESI) for Chemical Science.
This journal is © The Royal Society of Chemistry 2019

Electronic Supplementary Information

Rhodium-catalysed vinyl 1,4-conjugate addition coupled with Sharpless Asymmetric Dihydroxylation in the synthesis of the CDE ring fragment of pectenotoxin-4

Melodie S. W. Richardson,[a] Christopher J Tame,[b] Darren L. Poole[b] and Timothy J. Donohoe[a]*

[a] Department of Chemistry, University of Oxford, Chemistry Research Laboratory, Mansfield Road, Oxford, OX1 3TA (UK)
[b] GlaxoSmithKline Medicines Research Centre, Gunnels Wood Road, Stevenage, SG1 2NY (UK)

*E-mail: timothy.donohoe@chem.ox.ac.uk

Table of Contents

I)	General Experimental	S2
II)	Experimental Procedures	S3
III)	^{1}H and ^{13}C NMR spectra	S16
IV)	Mosher's Ester Analysis of **13**	S46
V)	nOe analyses of **23**, **27**, **28**, **33** and **37**	S47
VI)	References	S57

I) General Experimental

Solvents and Reagents: All reactions were performed using flame-dried reaction vessels under an atmosphere of argon unless stated otherwise. Anhydrous diethyl ether (Et_2O), dichloromethane (CH_2Cl_2), methanol (MeOH), acetonitrile (MeCN), toluene (PhMe) and tetrahydrofuran (THF) were obtained from MBRAUN SPS-5 solvent purification system by passage through double filtration columns under N_2. Brine refers to a saturated aqueous solution of NaCl.
2,3-Dibromopropene was purchased from Sigma Aldrich (80% purity, technical grade, 106003-25G). Methyl vinyl ketone (MVK) was purchased from Alfa Aesar (90% purity, technical grade, stabilised with 0.5% hydroquinone, A11910). Potassium phenoxide (KOPh) was prepared according to the procedure described by Kornblum and Lurie.[1] Propargyl bromide was purchased from Sigma Aldrich (purum, ~80% in toluene, 81831-250ML). Other reagents were used as supplied.

Chromatography: Thin layer chromatography (TLC) was performed on pre-coated aluminium-backed Merck TLC Silica Gel 60 F_{254} plates (particle size 0.2 mm). Preparative TLC was performed using glass-backed (500 µm) Merck Kieselgel 60 F_{254} plates. Plates were visualised by the quenching of fluorescence under ultraviolet light (λ_{max} = 254 nm) and by staining and heating with potassium permanganate, anisaldehyde, vanillin, ammonium molybdate or ceric ammonium molybdate as appropriate. Flash column chromatography was performed using Merck Geduran® 60 silica gel (particle size 40–63 µm) unless stated otherwise, with the solvent system given in parentheses. All solvents used for chromatography purification were HPLC grade or equivalent and supplied by Honeywell, Sigma Aldrich or Thermo Fisher Scientific.

Optical rotation: Specific optical rotations were measured on a Schmidt Haensch Unipol L2000 Polarimeter with a 100 mm quartz glass microtube (1 cm^3 volume) at the sodium D line (589.3 nm) at 25 °C in $CHCl_3$ or ACS photo spectroscopic grade CH_2Cl_2 and are reported in the units of 10^{-1} deg cm^2 g^{-1}. Solution concentrations are given in units of 10^{-2} g mL^{-1}.

Melting points: Melting points (m.p.) were obtained using a Leica VMTG heated-stage microscope equipped with a Testo 720 thermometer and are uncorrected.

Infrared spectroscopy: Fourier-transform infrared (FT-IR) spectra were recorded using a Bruker Tensor 27 fourier-transform infrared (FT-IR) spectrometer as evaporated films on a Pike Miracle Attenuated Total Reflectance (ATR) diamond module. Absorption maxima (v_{max}) are reported in wavenumbers (cm^{-1}) in the range 3600–900 cm^{-1}.

NMR spectroscopy: All spectra were recorded on Bruker AVIII HD 400 nanobay equipped with a 5 mm z-gradient BBFO probe, Bruker AVIII HD 400 nanobay equipped with a 5 mm z-gradient BBFO "SMART" probe, Bruker AVIII HD 500 equipped with a 5 mm BBF/H "SMART" probe, or Bruker AVIII 700 with inverse TCI $^{1}H/^{13}C/^{15}N$ cryoprobe, with the deuterated solvent acting as internal deuterium lock.
^{1}H NMR spectra were recorded at 400 or 500 or 700 MHz, ^{13}C NMR spectra at 101 or 126 MHz with broadband decoupling and ^{19}F NMR spectra at 471 MHz without decoupling.
Residual protic solvent signal acted as an internal reference for ^{1}H NMR, and deuterated solvent carbon signal acted as an internal reference for ^{13}C NMR ($CDCl_3$: ^{1}H NMR = 7.26 ppm, ^{13}C NMR = 77.16 ppm; CD_2Cl_2: ^{1}H NMR = 5.32 ppm, ^{13}C NMR = 54.00 ppm; C_6D_6: ^{1}H NMR = 7.16 ppm, ^{13}C NMR = 128.06 ppm). ^{11}B and ^{19}F NMR spectra were not externally referenced. Chemical shifts δ are quoted in parts per million (ppm) to the nearest 0.01 ppm for ^{1}H and ^{19}F NMR, and 0.1 ppm for ^{13}C NMR. The multiplicity of signal is reported as such: s–singlet, d–doublet, dd–double doublet, ddd– doublet of doublet of doublets, dt–double triplet, ddt–doublet of doublet of triplets, t–triplet, q–quartet, qd– quadruple doublet, m–multiplet, br.–broad, or combinations thereof. Coupling constants (*J*) are quoted in Hz to the nearest 0.1 Hz.
Structural assignments were made with the aid of DEPT135, DEPTQ, COSY, HSQC, HMBC, NOESY and TOCSY experiments.

Mass spectrometry: High resolution mass spectra (HRMS) electrospray ionisation (ESI) was performed using a Thermo Exactive mass spectrometer equipped with Waters Acquity liquid chromatography system with a flow rate of 0.2 mL/min using water:methanol:formic acid (10:89.9:0.1) as the eluent. Instrument control and data processing were performed using Thermo Xcalibur Software. The mass spec was operated using the heated electrospray (HESI-II) probe and resolution was set to 50,000 FWHM. Electrospray source conditions were adjusted to maximise sensitivity. For the electron ionisation (EI) and Chemical ionisation (CI) accurate mass service, analyses were performed on an Agilent 7200 quadrupole time of flight (Q-ToF) instrument equipped with

a direct insertion probe supplied by Scientific Instrument Manufacturer (SIM) GmbH. Instrument control and data processing were performed using Agilent MassHunter software. Source conditions for both EI and CI were adjusted to maximise sensitivity, the reagent gas used in CI was ammonia. Both systems were calibrated on the day of the analysis and its mass accuracy with external calibration (as used for these experiments) is better than 5 ppm for 24 h following calibration. The mass reported is that containing the most abundant isotopes, with each value to 4 decimal places and within 5 ppm of the calculated mass. Mass to charge ratios (m/z) are reported in Daltons.

Reporting of compounds: Compounds that are not assigned a compound number are labelled S1, S2 etc. Systematic names were generated by the computer programme ChemDraw according to the guidelines specified by the International Union of Pure and Applied Chemistry (IUPAC). However, the numbering on the structures may not correspond to the systematic name. The NMR assignments follow the numbering system shown on the structures for straightforward comparison of data. Compounds **S10**, **30**, **31**, **33**, **34**, **35**, **36** and **37** follow the pectenotoxin natural product numbering,[2] with the protecting group numbering in prime notation.

II) Experimental Procedures

(2*R*,3*S*,4*R*)-3,4-Bis(benzyloxy)-2-((benzyloxy)methyl)tetrahydrofuran (9)

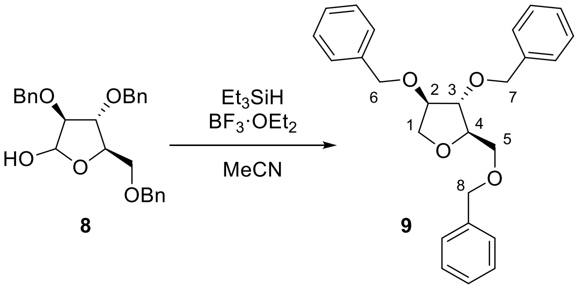

To a solution of commercially available furanose **8** (9.66 g, 23.0 mmol) in MeCN (100 mL) was added triethylsilane (14.7 mL, 91.9 mmol) followed by $BF_3{\cdot}OEt_2$ (5.7 mL, 46 mmol) dropwise. The reaction was stirred at rt for 22 h, after which sat. aq. $NaHCO_3$ (100 mL) was added. The layers were separated, and the aq. layer was extracted with Et_2O (3 × 100 mL). The combined organic layers were dried over $MgSO_4$, filtered and concentrated *in vacuo*. The crude product was purified by column chromatography (SiO_2, 90:10 pentane:EtOAc) to afford the title compound **9** (8.88 g, 95%) as a colourless oil.

R_f: 0.69 (50:50 pentane:EtOAc); **$[\alpha]_D^{25}$** –0.8 (*c* = 1.00, $CHCl_3$; lit.[5] **$[\alpha]_D^{25}$** 0.0, *c* = 1.00, $CHCl_3$); **^{1}H NMR:** δ_H (400 MHz, $CDCl_3$) 7.37–7.25 (15H, m, ArH), 4.62–4.42 (6H, m, C-6H_2, C-7H_2, C-8H_2), 4.10–3.99 (3H, m, C-2H, C-3H, C-4H), 4.02 (1H, dd, *J* = 10.1, 2.0 Hz, C-1H_AH_B), 3.94 (1H, dd, *J* = 10.0, 4.4 Hz, C-1H_AH_B), 3.65 (1H, dd, *J* = 10.0, 6.1 Hz, C-5H_AH_B), 3.60 (1H, dd, *J* = 10.0, 5.7 Hz, C-5H_AH_B); **^{13}C NMR:** δ_C (101 MHz, $CDCl_3$) 138.3 (quaternary Ar), 137.9 (2C, 2 quaternary Ar), 128.6 (2C, Ar), 128.5 (2C, Ar), 128.0 (Ar), 127.9 (5C, 4 Ar), 127.8 (2C, Ar), 127.7 (Ar), 84.7 (C-3), 83.3, 82.9 (C-2/4), 71.9 (C-1), 73.5, 71.7, 71.3 (C-6/7/8), 70.5 (C-5); Spectroscopic data were consistent with those in the literature.[5]

((2*R*,3*R*,4*R*)-3,4-Bis(benzyloxy)tetrahydrofuran-2-yl)methanol (11)

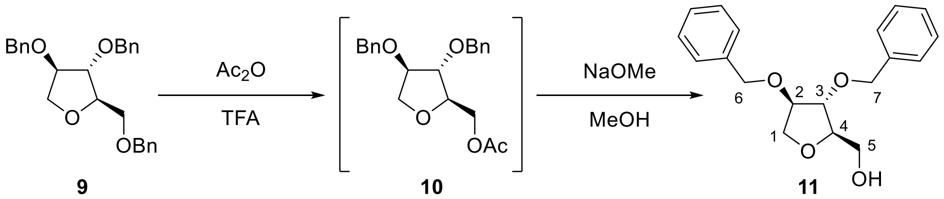

To a solution of **9** (8.81 g, 21.8 mmol) in acetic anhydride (88.0 mL, 931 mmol) was added trifluoroacetic acid (21.7 mL, 283 mmol) and stirred at rt for 6 h. The reaction was poured into a stirred mixture of Na_2CO_3 (100 g) in ice water (500 mL). The layers were separated, and the aq. layer was extracted with Et_2O (3 × 250 mL). The combined organic layers were washed with sat. aq. $NaHCO_3$ (2 × 250 mL), dried over $MgSO_4$, filtered and concentrated *in vacuo*. The crude product **10** was obtained as a yellow oil and was carried into the next step without further purification.
Crude product **10** (assumed quant., 21.8 mmol) was redissolved in MeOH (58 mL) and NaOMe (2.35 g, 43.6 mmol) in MeOH (8.9 mL) was added. The reaction was stirred at rt for 17 h before concentrated *in vacuo*. The mixture was redissolved in Et_2O (100 mL) and H_2O (100 mL) and the layers were separated. The aq. layer was extracted with Et_2O (3 × 100 mL). The combined organic layers were dried over $MgSO_4$, filtered and concentrated *in vacuo*. The crude product was purified by column chromatography (SiO_2, 90:10 → 80:20 → 1:1 pentane:EtOAc) to afford the title compound **11** (6.08 g, 89% over two steps) as a colourless oil.

R_f: 0.40 (50:50 pentane:EtOAc); **$[\alpha]_D^{25}$** +22.0 (*c* = 1.00, $CHCl_3$; lit.[5] **$[\alpha]_D^{25}$** +24.0, *c* = 1.00, $CHCl_3$); **^{1}H NMR:** δ_H (400 MHz, $CDCl_3$) 7.40–7.26 (10H, m, ArH), 4.56 (2H, d, *J* = 1.5 Hz, C-6H_2), 4.52 (2H, s, C-7H_2), 4.09–3.96 (4H, m, C-1H_AH_B, C-2H, C-3H, C-4H), 3.93 (1H, dd, *J* = 10.3, 4.2 Hz, C-1H_AH_B), 3.80 (1H, dd, *J* = 11.8, 3.2 Hz, C-5H_AH_B), 3.69 (1H, dd, *J* = 11.7, 4.6 Hz, C-5H_AH_B); **^{13}C NMR:** δ_C

(101 MHz, $CDCl_3$) 137.7 (quaternary Ar), 137.5 (quaternary Ar), 128.7 (4C, 2Ar), 128.1 (2C, Ar), 127.9 (2C, Ar), 127.8 (2C, 2Ar), 84.7, 83.8, 82.9 (C-2/3/4), 72.2 (C-6), 71.8 (C-1), 71.3 (C-7), 63.1 (C-5); Spectroscopic data were consistent with those in the literature.[5]

(2-Bromoallyl)trimethylsilane (12)

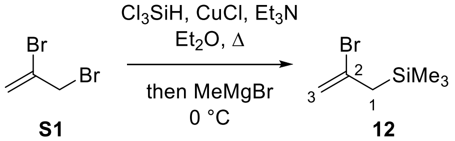

To a three-necked 1 L flask attached to a condenser and an overhead stirrer, was charged with CuCl (665 mg, 6.71 mmol) and Et_3N (18.7 mL, 134 mmol) in Et_2O (64 mL). To this mechanically stirred suspension was added a mixture of 2,3-dibromopropene **S1** (80% purity, 13.1 mL, 134 mmol) and trichorosilane (14.9 mL, 148 mmol) dropwise at a rate-maintaining gentle reflux. The reaction was stirred for 6 h then cooled to 0 °C. Methyl magnesium bromide solution (3 M in Et_2O, 201 mL, 604 mmol) was added carefully *via* cannula dropwise. The reaction was warmed to rt over 15 h before cooling back to 0 °C. The reaction was quenched carefully with sat. aq. NH_4Cl (320 mL, gas released *via* vent) and this was poured into Et_2O (120 mL) and H_2O (120 mL). The layers were separated and the organic layer was washed with H_2O (3 × 120 mL). The combined aq. layers were extracted Et_2O (5 × 100 mL). The combined organic layers were dried over $MgSO_4$, filtered and concentrated *in vacuo* (volatile, 400 mbar, rt). The crude product was purified by vacuum distillation (26 mbar, 52 °C) to afford the title compound **12** (10.75 g, 38%) as a colourless oil.

R_f: 0.75 (50:50 pentane:Et_2O); **^{1}H NMR:** δ_H (400 MHz, $CDCl_3$) 5.31 (1H, dt, J = 1.3, 0.7 Hz, C-3H_AH$_B$), 5.22 (1H, dd, J = 1.6, 0.5 Hz, C-3H$_A$$H_B$), 2.11 (2H, s, C-1$H_2$), 0.11 (9H, s, $Si(CH_3)_3$); **^{13}C NMR:** δ_C (101 MHz, $CDCl_3$) 131.3 (C-2), 114.1 (C-3), 33.5 (C-1), –1.4 (3C, $Si(CH_3)_3$); Spectroscopic data were consistent with those in the literature.[6]

(*R*)-1-((2*R*,3*S*,4*R*)-3,4-Bis(benzyloxy)tetrahydrofuran-2-yl)-3-bromobut-3-en-1-ol (13)

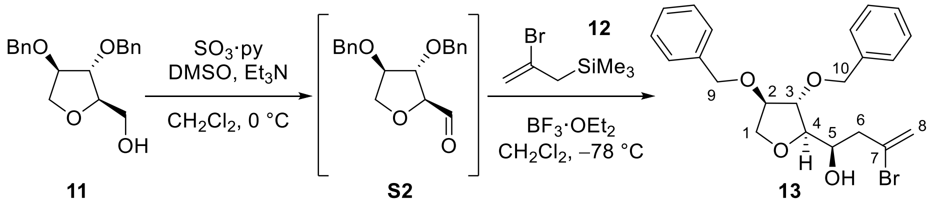

11 **S2** **13**

To a solution of **11** (105 mg, 0.33 mmol) and Et_3N (0.55 mL, 3.18 mmol) in CH_2Cl_2 (2 mL) at 0 °C was added SO_3·py (253 mg, 1.59 mmol) in DMSO (1.69 mL, 23.9 mmol). The reaction was warmed to rt and stirred for 3 h 30 min before it was diluted with a 1:1 mixture of hexane:EtOAc (20 mL). The solution was washed with 1 M aq. HCl (2 × 15 mL), sat. aq. $NaHCO_3$ (20 mL) and brine containing ice (5 × 20 mL). The organic layer was dried over $MgSO_4$, filtered and concentrated *in vacuo*. The crude product **S2** was obtained as a pale yellow oil and was carried into the next step without further purification.
Crude product **S2** (assume quant., 0.33 mmol) was transferred to a flask and was dried azeotropically with anhydrous benzene (3 × 3 mL) before redissolving in anhydrous CH_2Cl_2 (3 mL) and cooled to –78 °C. Bromide **12** (258 mg, 1.33 mmol) in CH_2Cl_2 (2 mL) was added, followed by BF_3·OEt_2 (82 µL, 0.67 mmol) dropwise. The reaction was warmed to rt for 17 h before it was quenched with sat. aq. $NaHCO_3$ (10 mL). The mixture was diluted with Et_2O (30 mL) and H_2O (10 mL). The layers were separated, and the aq. layer was extracted with Et_2O (2 × 30 mL). The combined organic layers were dried over $MgSO_4$, filtered and concentrated *in vacuo*. The crude product was purified by column chromatography (SiO_2, 70:30 pentane:Et_2O) to afford the title compound **13** (78.9 mg, 56% over two steps, >20:1 dr) as a colourless oil.

The stereochemistry of C-5 was assigned the (*R*)-configuration by Mosher's ester analysis.[7]

R_f: 0.37 (50:50 pentane:Et_2O); **$[\alpha]_D^{25}$** +25.4 (c = 1.00, $CHCl_3$); **IR:** ν_{max} (thin film/cm^{-1}) 3450, 2866, 1632, 1496, 1454, 1207, 1075, 1028, 893, 737, 698; **^{1}H NMR:** δ_H (400 MHz, $CDCl_3$) 7.43–7.26 (10H, m, ArH), 5.68 (1H, q, J = 1.3 Hz, C-8H_AH$_B$), 5.55 (1H, d, J = 1.7 Hz, C-8H$_A$$H_B$), 4.57 (2H, q, J = 11.8 Hz, C-10H_2), 4.55 (2H, s, C-9H_2), 4.22–4.15 (1H, m, C-5H), 4.17 (1H, d, J = 3.2 Hz, C-3H), 4.12–4.06 (2H, m, C-1H_AH$_B$, C-2H), 3.89 (1H, dd, J = 9.9, 3.5 Hz, C-1H$_A$$H_B$), 3.87–3.83 (1H, m, C-4H), 2.67 (1H, dd, J = 14.8, 3.8 Hz, C-6H_AH$_B$), 2.48 (1H, ddd, J = 14.7, 8.9, 0.8 Hz, C-6H$_A$$H_B$), 2.44 (1H, d, J = 2.0 Hz, OH); **^{13}C NMR:** δ_C (101 MHz, $CDCl_3$) 137.6 (quaternary Ar), 137.5 (quaternary Ar), 130.1 (C-7), 128.7 (4C, 2Ar), 128.2 (2C, Ar), 128.1 (2C, 2Ar), 127.9 (2C, Ar), 119.7 (C-8), 86.7 (C-4), 82.6 (C-3), 82.4 (C-2), 71.9 (C-10), 71.6 (C-1), 71.1 (C-9), 68.9 (C-5), 45.3 (C-6); **HRMS:** (Ammonia CI-MS, m/z) $C_{22}H_{25}{}^{79}BrO_4$ (MH^+) Calculated: 433.1009, Found: 433.1009 (Δ +0.00 ppm).

(((*R*)-1-((2*S*,3*S*,4*R*)-3,4-Bis(benzyloxy)tetrahydrofuran-2-yl)-3-bromobut-3-en-1-yl)oxy)triethylsilane (14)

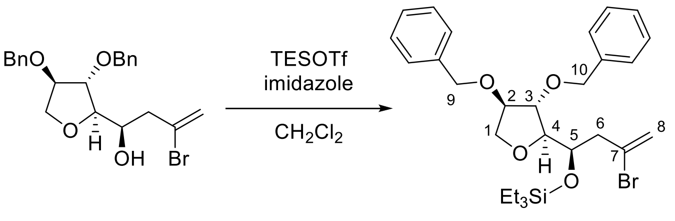

13 14

To a stirred solution of **13** (708 mg, 1.64 mmol) and imidazole (446 mg, 6.55 mmol) in CH_2Cl_2 (17 mL) was added TES triflate (593 μL, 3.28 mmol) and stirred at rt for 22 h. The TLC showed some starting material present so more TES triflate was added (300 μL) and stirred for a further 2 h. The reaction was quenched with H_2O (40 mL), the layers separated, and the aq. layer was extracted with CH_2Cl_2 (3 × 40 mL). The combined organic layers were dried over $MgSO_4$, filtered and concentrated *in vacuo*. The crude product was purified by column chromatography (SiO_2, 100:0 → 99:1 → 95:5 pentane:Et_2O) to afford the title compound **14** (764 mg, 85%) as a colourless oil.

R_f: 0.58 (70:30 pentane:Et_2O); **$[\alpha]_D^{25}$** +11.1 (*c* = 1.00, $CHCl_3$); **IR:** ν_{max} (thin film/cm^{-1}) 2954, 2875, 1632, 1496, 1455, 1392, 1240, 1101, 1009, 891, 735, 698; **^{1}H NMR:** δ_H (400 MHz, $CDCl_3$) 7.40–7.23 (10H, m, ArH), 5.59 (1H, d, *J* = 1.3 Hz, C-8H_AH$_B$), 5.43 (1H, d, *J* = 1.5 Hz, C-8H$_A$$H_B$), 4.59 (1H, d, *J* = 11.6 Hz, C-10H_AH$_B$), 4.56 (1H, d, *J* = 11.6 Hz, C-10H$_A$$H_B$), 4.54 (1H, d, *J* = 12.0 Hz, C-9H_AH$_B$), 4.49 (1H, d, *J* = 12.0 Hz, C-9H$_A$$H_B$), 4.21 (1H, dt, *J* = 7.3, 4.3 Hz, C-5H), 4.14 (1H, ddd, *J* = 5.0, 2.1, 0.9 Hz, C-3H), 4.09 (1H, dt, *J* = 4.6, 2.3 Hz, C-2H), 3.99 (1H, dd, *J* = 10.1, 2.4 Hz, C-1H_AH$_B$), 3.90 (1H, dd, *J* = 10.0, 4.8 Hz, C-1H$_A$$H_B$), 3.78 (1H, dd, *J* = 4.9, 3.9 Hz, C-4H), 2.67 (1H, ddd, *J* = 14.6, 4.5, 1.1 Hz, C-6H_AH$_B$), 2.54 (1H, ddd, *J* = 14.5, 7.3, 0.8 Hz, C-6H$_A$$H_B$), 0.96 (9H, dt, *J* = 13.3, 7.9 Hz, $Si(CH_2CH_3)_3$), 0.66–0.55 (6H, m, $Si(CH_2CH_3)_3$); **^{13}C NMR:** δ_C (101 MHz, $CDCl_3$) 138.0 (2C, 2 quaternary Ar), 131.0 (C-7), 128.6 (2C, Ar), 128.5 (2C, Ar), 127.9 (4C, 3 Ar), 127.8 (2C, Ar), 119.8 (C-8), 86.2 (C-4), 83.9 (C-3), 83.6 (C-2), 72.0 (C-10), 71.6 (C-1), 71.4 (C-9), 69.9 (C-5), 46.0 (C-6), 7.1 (3C, $Si(CH_2CH_3)_3$), 5.3 (3C, $Si(CH_2CH_3)_3$); **HRMS:** (ESI+, *m/z*) $C_{28}H_{39}O_4{}^{79}Br^{28}SiNa$ (MNa^+) Calculated: 569.16932, Found: 569.16917 (Δ –0.26 ppm).

(((*R*)-1-((2*S*,3*S*,4*R*)-3,4-Bis(benzyloxy)tetrahydrofuran-2-yl)-3-(4,4,5,5-tetramethyl-1,3,2-dioxaborolan-2-yl)but-3-en-1-yl)oxy)triethylsilane (15)[3]

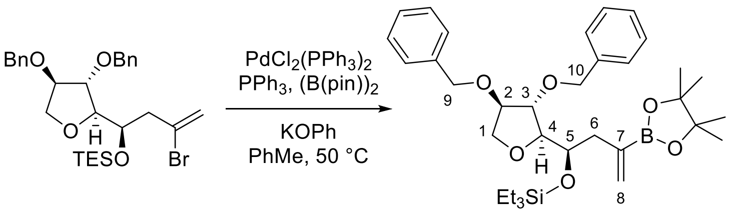

14 15

To a flask charged with $PdCl_2(PPh_3)_2$ (59 mg, 84 μmol), PPh_3 (44 mg, 0.17 mmol), bis(pinacolato)diboron (785 mg, 3.09 mmol) and KOPh[1] (557 mg, 4.21 mmol) was added compound **14** (1.54 g, 2.80 mmol) in PhMe (17 mL). The reaction was flushed under argon and heated to 50 °C for 19 h before adding additional bis(pinacolato)diboron (150 mg, 0.591 mmol). The reaction was heated to 50 °C for a further 21 h and then cooled to rt. Water (20 mL) was added and the layers were separated. The aq. layer was extracted with Et_2O (3 × 20 mL). The combined organic layers were washed with brine (20 mL), dried over $MgSO_4$, filtered and concentrated *in vacuo*. The crude product was purified by column chromatography (SiO_2, 100:0 → 90:10 pentane:Et_2O) to afford the title compound **15** (1.32 g, 79%) as a colourless oil.

R_f: 0.48 (80:20 pentane:Et_2O); **$[\alpha]_D^{25}$** +2.8 (*c* = 1.00, $CHCl_3$); **IR:** ν_{max} (thin film/cm^{-1}) 2951, 2875, 1617, 1455, 1390, 1370, 1310, 1209, 1143, 1095, 967, 899, 864, 736, 698; **^{1}H NMR:** δ_H (400 MHz, $CDCl_3$) 7.36–7.25 (10H, m, ArH), 5.82 (1H, d, *J* = 3.7 Hz, C-8H_AH$_B$), 5.63 (1H, d, *J* = 3.6 Hz, C-8H$_A$$H_B$), 4.62 (1H, d, *J* = 11.4 Hz, C-10H_AH$_B$), 4.57 (1H, d, *J* = 11.4 Hz, C-10H$_A$$H_B$), 4.51 (2H, s, C-9H$_2$), 4.27 (1H, dd, *J* = 5.0, 2.2 Hz, C-3H), 4.12–4.15 (2H, m, C-2H, C-5H), 3.93 (1H, dd, *J* = 9.8, 3.3 Hz, C-1H_AH$_B$), 3.89 (1H, dd, *J* = 9.8, 5.2 Hz, C-1H$_A$$H_B$), 3.78 (1H, dd, *J* = 5.1, 3.6 Hz, C-4H), 2.45 (1H, dd, *J* = 13.2, 6.4 Hz, C-6H_AH$_B$), 2.28 (1H, dd, *J* = 13.4, 6.3 Hz, C-6H$_A$$H_B$), 1.23 (12H, s, 2 × $C(CH_3)_2$), 0.93 (9H, t, *J* = 7.9 Hz, $Si(CH_2CH_3)_3$), 0.59 (6H, q, *J* = 7.8 Hz, $Si(CH_2CH_3)_3$); **^{13}C NMR:** δ_C (101 MHz, $CDCl_3$) 138.3 (quaternary Ar), 138.2 (quaternary Ar), 137.9 (v. br., C-7), 132.8 (C-8), 129.7 (Ar), 128.5 (4C, 2Ar), 127.9 (2C, Ar), 127.8 (3C, 2Ar), 86.2 (C-4), 84.2 (C-2), 83.9 (C-3), 83.5 (2C, 2 × $C(CH_3)_2$), 72.1 (C-10), 71.4 (2C, C-1, C-9), 71.3 (C-5), 41.0 (C-6), 25.0 (2C, 2 × $C(CH_3)_2$), 24.9 (2C, 2 × $C(CH_3)_2$), 7.1 (3C, $Si(CH_2CH_3)_3$), 5.3 (3C, $Si(CH_2CH_3)_3$); **HRMS:** (ESI+, *m/z*) $C_{34}H_{51}O_6{}^{11}B^{28}SiNa$ (MNa^+) Calculated: 617.34402, Found: 617.34363 (Δ –0.63 ppm).

1-((*R*)-Tetrahydrofuran-2-yl)prop-2-en-1-ol (18)

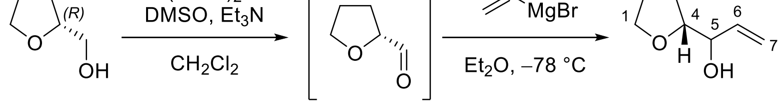

16 17 18

To a solution of DMSO (1.89 mL, 26.6 mmol) in CH_2Cl_2 (26.6 mL) under argon at –78 °C was added oxalyl chloride (1.03 mL, 11.8 mmol) dropwise. The mixture was stirred for 30 min before (*R*)-tetrahydrofurfuryl alcohol **16** (0.92 mL, 9.49 mmol) was added dropwise. The reaction was stirred at –78 °C for a further 30 min before Et_3N (6.8 mL, 49 mmol) was added. The solution was warmed to rt and H_2O (27 mL) was added. When the solution cleared, the layers were separated, and the aq. layer was extracted with CH_2Cl_2 (3 × 30 mL). NaCl was added to the aq. layer until saturated, and then the aq. layer was extracted further with Et_2O (3 × 30 mL). The combined organic layers were dried over Na_2SO_4, filtered and concentrated under a stream of N_2 (volatile). The crude product **17** was obtained as a pale yellow oil and white precipitate and was carried into the next step without further purification.[8] Crude **17** (assumed quant., 9.49 mmol) was transferred to a flask in anhydrous Et_2O (14.3 mL) and cooled to –78 °C. Vinyl magnesium bromide (1 M in THF, 14.3 mL, 14.3 mmol) was added at –78 °C and was warmed to rt over 15 h. The reaction was quenched with sat. aq. NH_4Cl (30 mL) and the layers were separated. The aq. layer was extracted with Et_2O (3 × 30 mL) and CH_2Cl_2 (3 × 30 mL). The combined organic layers were dried over Na_2SO_4, filtered and concentrated under a stream of N_2 (azeotropes). The crude product was purified by column chromatography (SiO_2, 1:1 pentane:Et_2O) to afford the title compound **18** (431 mg, 35% over two steps, 1:1.15 dr) as a colourless oil.

Diastereomeric ratio at C-5 was ascertained by ^1H NMR spectroscopy of the crude mixture; δ_H 5.29 (1 H, dt, *J* = 6.1, 1.7 Hz, C-7H$_A$*H$_B$* minor), 5.20–5.17 (1 H, m, C-7'*H$_A$*H$_B$ major). The diastereoisomers are identified by prime notation.

R*f*: 0.34 (50:50 pentane:EtOAc); **[*α*]$_D^{25}$** +6.4 (*c* = 1.00, CH_2Cl_2); **IR:** ν_{max} (thin film/cm^{-1}) 3437, 3088, 2976, 2875, 1644, 1428, 1261, 1185, 1064, 995, 924, 802, 617; **^{1}H NMR:** δ_H (400 MHz, CD_2Cl_2) 5.82 (2H, dddd, *J* = 17.3, 10.5, 5.8, 4.8 Hz, C-6H, C-6'H), 5.34 (1H, dt, *J* = 6.1, 1.7 Hz, C-7*H$_A$*H$_B$), 5.29 (1H, dt, *J* = 6.1, 1.7 Hz, C-7H$_A$*H$_B$*), 5.20–5.17 (1H, m, C-7'*H$_A$*H$_B$), 5.17–5.14 (1H, m, C-7'H$_A$*H$_B$*), 4.27–4.22 (1H, m, C-5H), 3.92–3.69 (7H, m, C-1H$_2$, C-1'H$_2$, C-4H, C-4'H, C-5'H), 2.45 (1H, d, *J* = 3.8 Hz, OH), 2.10 (1H, d, *J* = 3.3 Hz, OH'), 1.95–1.60 (8H, m, C-2H$_2$, C-2'H$_2$, C-3H$_2$, C-3'H$_2$); **^{13}C NMR:** δ_C (101 MHz, CD_2Cl_2) 138.0 (C-6), 137.5 (C-6'), 116.7 (C-7), 116.2 (C-7'), 82.5 (C-4), 82.2 (C-4'), 75.8 (C-5), 74.0 (C-5'), 69.3 (C-1), 68.7 (C-1'), 28.1 (C-3), 26.6 (2C, C-2, C-3'), 25.6 (C-2'); **HRMS:** (Ammonia CI-MS, *m/z*) $C_7H_{16}O_2N$ (MNH_4^+) Calculated: 146.1176, Found: 146.1171 (Δ +3.42 ppm).

(*R*)-1-(Tetrahydrofuran-2-yl)prop-2-en-1-one (19)

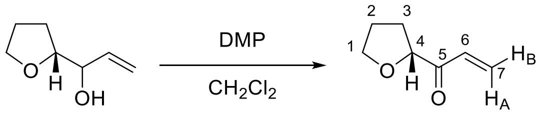

18 **19**

To a solution of **18** (431 mg, 3.36 mmol) in wet CH_2Cl_2 (39 mL) was added DMP (2.85 g, 6.73 mmol) and was stirred at rt for 2 h. Sat. aq. $NaHCO_3$ (40 mL) was added and the layers were separated. The aq. layer was extracted with CH_2Cl_2 (3 × 40 mL). The combined organic layers were dried over Na_2SO_4, filtered and concentrated by a stream of N_2 (volatile). The crude product was purified by column chromatography (SiO_2, 100:0 → 90:10 pentane:Et_2O) to afford the title compound **19** (376 mg, 89%) as a colourless oil.

R*f*: 0.30 (50:50 pentane:Et_2O); **[*α*]$_D^{25}$** +49.4 (*c* = 1.00, CH_2Cl_2); **IR:** ν_{max} (thin film/cm^{-1}) 2955, 2874, 1698, 1612, 1458, 1403, 1178, 1056, 990, 929, 792, 683; **^{1}H NMR:** δ_H (400 MHz, CD_2Cl_2) 6.65 (1H, dd, *J* = 17.5, 10.6 Hz, C-6H), 6.32 (1H, dd, *J* = 17.5, 1.7 Hz, C-7*H$_A$*H$_B$), 5.79 (1H, dd, *J* = 10.6, 1.7 Hz, C-7H$_A$*H$_B$*), 4.53–4.47 (1H, m, C-4H), 3.94–3.86 (2H, m, C-1H$_2$), 2.25–2.12 (1H, m, C-3*H$_A$*H$_B$), 1.97–1.83 (3H, m, C-2H$_2$, C-3H$_A$*H$_B$*); **^{13}C NMR:** δ_C (101 MHz, CD_2Cl_2) 200.7 (C-5), 132.5 (C-6), 129.5 (C-7), 82.7 (C-4), 69.8 (C-1), 29.7 (C-3), 26.1 (C-2); **HRMS:** (ESI+, *m/z*) $C_7H_{11}O_2$ (MH$^+$) Calculated: 127.07536, Found: 127.07551 (Δ +1.18 ppm).

(*R*)-6-((2*S*,3*S*,4*R*)-3,4-Bis(benzyloxy)tetrahydrofuran-2-yl)-4-methylene-1-((*R*)-tetrahydrofuran-2-yl)-6-((triethylsilyl)oxy)hexan-1-one (20)[4]

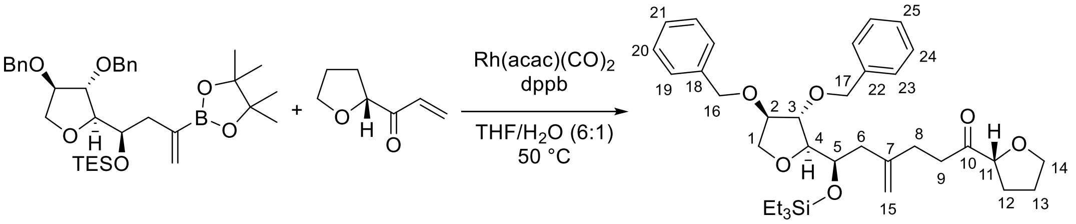

15 **19** **20**

To a solution of compound **15** (50 mg, 84 μmol), Rh(acac)(CO)$_2$ (2.2 mg, 8.4 μmol) and dppb (3.6 mg, 8.4 μmol) in THF (0.30 mL) and H_2O (0.05 mL) was added enone **19** (12.7 mg, 101 μmol) and the vial was flushed with argon. The reaction heated to 50 °C for 23 h and then cooled to rt. EtOAc (1 mL) and H_2O (1 mL) were added and the layers were separated. The aq. layer was extracted with EtOAc (3 × 2 mL). The combined organic layers were dried over $MgSO_4$, filtered and concentrated *in vacuo*. The crude product was purified by column chromatography (SiO_2, 100:0 → 80:20 → 70:30 pentane:Et_2O) to afford the title compound **20** (31.4 mg, 63%, >20:1 dr) as a colourless oil.

R*f*: 0.22 (70:30 pentane:Et_2O); **[*α*]$_D^{25}$** +12.1 (*c* = 1.00, $CHCl_3$); **IR:** ν_{max} (thin film/cm^{-1}) 2952, 2911, 2875, 1716, 1645, 1496, 1455, 1412, 1368, 1308, 1261, 1207, 1077, 1016, 970, 896, 801, 736, 698; **^{1}H NMR:** δ_H (400 MHz, $CDCl_3$) 7.41–7.23 (10H, m, ArH), 4.83 (1H, s, C-15*H$_A$*H$_B$), 4.79 (1H, q, *J* = 1.6 Hz, C-15H$_A$*H$_B$*), 4.59 (1H, d, *J* = 11.6 Hz, C-17*H$_A$*H$_B$), 4.55 (1H, d, *J* = 11.6 Hz, C-17H$_A$*H$_B$*), 4.53 (1H, d, *J* = 11.9 Hz, C-16*H$_A$*H$_B$), 4.49 (1H, d, *J* = 11.9 Hz, C-16H$_A$*H$_B$*), 4.30 (1H, dd, *J* = 8.1, 6.0 Hz, C-11H), 4.18 (1H, ddd, *J* =

4.6, 2.0, 0.8 Hz, C-3H), 4.08 (1H, dt, *J* = 4.6, 2.2 Hz, C-2H), 4.02 (1H, ddd, *J* = 6.5, 5.6, 4.6 Hz, C-5H), 3.99–3.85 (4H, m, C-1H$_A$H$_B$, C-14H$_2$), 3.76 (1H, t, *J* = 4.5 Hz, C-4H), 2.76–2.58 (2H, m, C-9H$_2$), 2.39–2.26 (3H, m, C-6*H*$_A$H$_B$, C-8H$_2$), 2.26–2.10 (2H, m, C-6H$_A$*H*$_B$, C-12*H*$_A$H$_B$), 1.88 (3H, ddd, *J* = 6.1, 3.7, 2.1 Hz, C-12H$_A$*H*$_B$, C-13H$_2$), 0.93 (9H, t, *J* = 7.9 Hz, Si(CH$_2$C*H*$_3$)$_3$), 0.58 (6H, q, *J* = 7.8 Hz, Si(C*H*$_2$CH$_3$)$_3$); **^{13}C NMR:** δ_C (101 MHz, CDCl$_3$) 211.8 (C-10), 144.9 (C-7), 138.1 (2C, C-18, C-22), 128.5 (4C, 2 × C-20, 2 × C-24), 127.9 (2C, 2 × C-23), 127.8 (4C, 2 × C-19, C-21, C-25), 112.6 (C-15), 86.1 (C-4), 83.9 (C-3), 83.8 (C-2), 83.5 (C-11), 71.9 (C-17), 71.4 (2C, C-1, C-16), 70.8 (C-5), 69.5 (C-14), 41.0 (C-6), 36.7 (C-9), 29.8 (C-8), 29.2 (C-12), 25.8 (C-13), 7.1 (3C, Si(CH$_2$*C*H$_3$)$_3$), 5.3 (3C, Si(*C*H$_2$CH$_3$)$_3$); **HRMS:** (ESI+, *m/z*) $C_{35}H_{50}O_6{}^{28}SiNa$ (MNa$^+$) Calculated: 617.32689, Found: 617.32655 (Δ –0.55 ppm).

1-(Tetrahydrofuran-2-yl)prop-2-en-1-ol (S5)

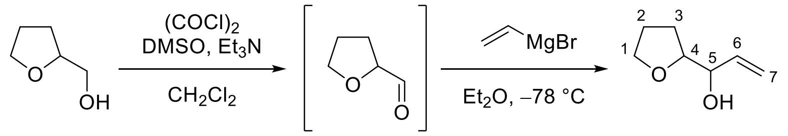

S3 **S4** **S5**

To a solution of DMSO (8.90 mL, 103 mmol) in CH$_2$Cl$_2$ (140 mL) under argon at –78 °C was added oxalyl chloride (5.40 mL, 61.9 mmol) dropwise. The mixture was stirred for 30 min before tetrahydrofurfuryl alcohol **S3** (5.0 mL, 52 mmol) was added dropwise. The reaction was stirred at –78 °C for a further 30 min before Et$_3$N (36.0 mL, 258 mmol) was added. The solution was warmed to rt and H$_2$O (140 mL) was added. The layers were separated and the aq. layer was extracted with CH$_2$Cl$_2$ (3 × 100 mL). NaCl was added to the aq. layer until saturated, and then the aq. layer was extracted further with Et$_2$O (3 × 100 mL). The combined organic layers were dried over Na$_2$SO$_4$, filtered and concentrated *in vacuo* (volatile, 400 mbar, rt). The crude product **S4** was obtained as a pale yellow oil and white precipitate and was carried into the next step without further purification.[8]
Crude **S4** (assumed quant., 51.6 mmol) was transferred to a flask in anhydrous Et$_2$O (55 mL) and cooled to –78 °C. Vinyl magnesium bromide (1 M in THF, 77 mL, 77.0 mmol) was added at –78 °C and was warmed to rt over 2 h. TLC analysis indicated incomplete reaction so additional vinyl magnesium bromide (20.0 mL, 20.0 mmol) was added, and stirring was continued for 15 h. The reaction was quenched with sat. aq. NH$_4$Cl (80 mL) and the layers were separated. The aq. layer was extracted with Et$_2$O (3 × 100 mL) and CH$_2$Cl$_2$ (3 × 100 mL). The combined organic layers were dried over Na$_2$SO$_4$, filtered and concentrated *in vacuo* (azeotropes, 400 mbar, rt). The crude product was purified by column chromatography (SiO$_2$, 1:1 pentane:Et$_2$O) to afford the title compound **S5** (1.55 g, 23% over two steps, 1:1.15 dr) as a colourless oil.

Diastereomeric ratio at C-5 was ascertained by ^{1}H NMR spectroscopy of the crude mixture; δ_H 5.29 (1 H, dt, *J* = 6.1, 1.7 Hz, C-7H$_A$*H*$_B$ minor), 5.20–5.17 (1 H, m, C-7'*H*$_A$H$_B$ major). The diastereoisomers are identified by prime notation.

R$_f$: 0.34 (50:50 pentane:EtOAc); **IR:** ν_{max} (thin film/cm^{-1}) 3424, 3082, 2977, 2872, 1645w, 1424, 1291, 1185, 1136, 1061, 994, 924, 702; **^{1}H NMR:** δ_H (400 MHz, CD$_2$Cl$_2$) 5.82 (2H, dddd, *J* = 17.2, 10.6, 5.8, 5.0 Hz, C-6H, C-6'H), 5.34 (1H, dt, *J* = 6.2, 1.6 Hz, C-7*H*$_A$H$_B$), 5.29 (1H, dt, *J* = 6.3, 1.7 Hz, C-7H$_A$*H*$_B$), 5.20–5.17 (1H, m, C-7'*H*$_A$H$_B$), 5.17–5.14 (1H, m, C-7'H$_A$*H*$_B$), 4.24 (1H, dtt, *J* = 5.3, 3.4, 1.6 Hz, C-5H), 3.93–3.69 (7H, m, C-1H$_2$, C-1'H$_2$, C-4H, C-4'H, C-5'H), 2.46 (1H, t, *J* = 3.2 Hz, OH), 2.13 (1 H, td, *J* = 3.7, 2.0 Hz, OH'), 1.95–1.57 (8H, m, C-2H$_2$, C-2'H$_2$, C-3H$_2$, C-3'H$_2$); **^{13}C NMR:** δ_C (101 MHz, CD$_2$Cl$_2$) 138.1 (C-6), 137.6 (C-6'), 116.7 (C-7), 116.2 (C-7'), 82.5 (C-4), 82.2 (C-4'), 75.8 (C-5), 74.0 (C-5'), 69.3 (C-1), 68.8 (C-1'), 28.1 (C-3), 26.6 (2 C, C-2, C-3'), 25.7 (C-2'); **HRMS:** (ESI+, *m/z*) $C_7H_{12}O_2Na$ (MNa$^+$) Calculated: 151.07295, Found: 151.07300 (Δ +0.33 ppm).

1-(Tetrahydrofuran-2-yl)prop-2-en-1-one (S6)

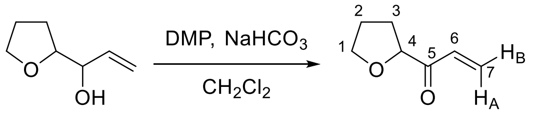

S5 **S6**

To a solution of **S5** (1.14 g, 8.93 mmol) and NaHCO$_3$ (3.75 g, 44.6 mmol) in CH$_2$Cl$_2$ (from Winchester, 103 mL) was added DMP (7.57 g, 17.9 mmol) and the reaction was stirred at rt for 28 h. The reaction was quenched with sat. aq. NaHCO$_3$ (50 mL) and the layers were separated. The aq. layer was extracted with CH$_2$Cl$_2$ (3 × 100 mL). The combined organic layers were dried over Na$_2$SO$_4$, filtered and concentrated *in vacuo* (volatile, cool bath). The crude product was purified by column chromatography (SiO$_2$, 100:0 → 90:10 → 80:20 pentane:Et$_2$O) to afford the title compound **S6** (736 mg, 65%) as a colourless oil.

R$_f$: 0.30 (50:50 pentane:Et$_2$O); **IR:** ν_{max} (thin film/cm^{-1}) 2979, 2874, 1697, 1611, 1448, 1403, 1289, 1177, 1053, 1027, 989, 927, 788, 682; **^{1}H NMR:** δ_H (400 MHz, CD$_2$Cl$_2$) 6.65 (1H, dd, *J* = 17.5, 10.6 Hz, C-6H), 6.32 (1H, dd, *J* = 17.5, 1.7 Hz, C-7*H*$_A$H$_B$), 5.79 (1H, dd, *J* = 10.6, 1.6 Hz, C-7H$_A$*H*$_B$), 4.54–4.46 (1H, m, C-4H), 3.94–3.85 (2H, m, C-1H$_2$), 2.24–2.12 (1H, m, C-3*H*$_A$H$_B$), 1.97–1.83 (3H, m, C-2H$_2$, C-3H$_A$*H*$_B$); **^{13}C NMR:** δ_C (101 MHz, CD$_2$Cl$_2$) 200.7 (C-5), 132.5 (C-6), 129.5 (C-7), 82.7 (C-4), 69.8 (C-1), 29.7 (C-3), 26.1 (C-2); **HRMS:** (ESI+, *m/z*) $C_7H_{10}O_2Na$ (MNa$^+$) Calculated: 149.05730, Found: 149.05746 (Δ +1.07 ppm).

(6*R*)-6-((2*S*,3*S*,4*R*)-3,4-bis(benzyloxy)tetrahydrofuran-2-yl)-4-methylene-1-(tetrahydrofuran-2-yl)-6-((triethylsilyl)oxy)hexan-1-one (S7)[4]

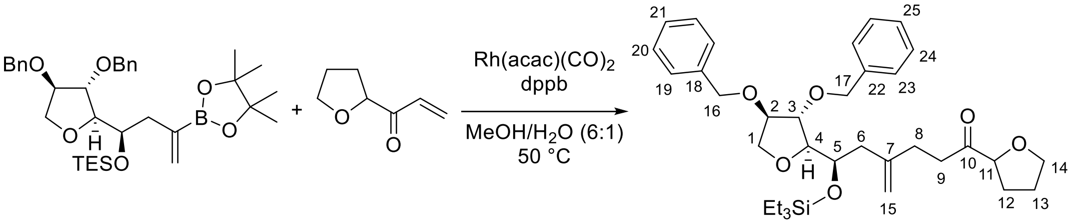

15 **S6** **S7**

To a solution of compound **15** (50 mg, 84 µmol), Rh(acac)(CO)$_2$ (2.2 mg, 8.4 µmol) and dppb (3.6 mg, 8.4 µmol) in THF (0.30 mL) and H_2O (0.05 mL) was added enone **S6** (12.7 mg, 101 µmol) and the vial was flushed with argon. The reaction heated to 50 °C for 21 h and then cooled to rt. EtOAc (1 mL) and H_2O (1 mL) were added and the layers were separated. The aq. layer was extracted with EtOAc (3 × 2 mL). The combined organic layers were dried over $MgSO_4$, filtered and concentrated *in vacuo*. The crude product was purified by column chromatography (SiO_2, 100:0 → 80:20 → 70:30 pentane:Et_2O) to afford the title compound **S7** (31.4 mg, 52%) as a colourless oil contaminated with **S8** (**S7:S8**, 1:0.18). The diastereomeric ratio could not be determined at C-11. The diastereoisomers are identified by prime notation. The * in the ^{13}C NMR denotes defined twinned peaks for each diastereomer.

R_f: 0.22 (70:30 pentane:Et_2O); **IR:** ν_{max} (thin film/cm^{-1}) 2952, 2911, 2875, 1716, 1645, 1496, 1455, 1412, 1368, 1308, 1261, 1207, 1077, 1016, 970, 896, 801, 736, 698; **^{1}H NMR:** δ_H (500 MHz, $CDCl_3$) 7.38–7.24 (10H, m, ArH, ArH'), 4.83 (1H, s, C-15H_AH_B, C-15'H_AH_B), 4.79 (1H, q, *J* = 1.6 Hz, C-15H_AH_B, C-15'H_AH_B), 4.59 (1H, d, *J* = 11.6 Hz, C-17H_AH_B, C-17'H_AH_B), 4.56 (1H, d, *J* = 11.6 Hz, C-17H_AH_B, C-17'H_AH_B), 4.53 (1H, d, *J* = 12.0 Hz, C-16H_AH_B, C-16'H_AH_B), 4.50 (1H, d, *J* = 12.0 Hz, C-16H_AH_B, C-16'H_AH_B), 4.30 (1H, dd, *J* = 8.2, 6.0 Hz, C-11H, C-11'H), 4.18 (1H, ddd, *J* = 4.6, 2.0, 0.7 Hz, C-3H, C-3'H), 4.08 (1H, dt, *J* = 4.6, 2.2 Hz, C-2H, C-2'H), 4.02 (1H, ddd, *J* = 6.5, 5.6, 4.6 Hz, C-5H, C-5'H), 3.99–3.86 (4H, m, C-1H_AH_B, C-1'H_AH_B, C-14H_2, C-14'H_2), 3.76 (1H, t, *J* = 4.6 Hz, C-4H, C-4'H), 2.76–2.67 (1H, m, C-9H_AH_B, C-9'H_AH_B), 2.66–2.58 (1H, C-9H_AH_B, C-9'H_AH_B), 2.37–2.25 (3H, m, C-6H_AH_B, C-6'H_AH_B, C-8H_2, C-8'H_2), 2.25–2.19 (1H, m, C-6H_AH_B, C-6'H_AH_B) 2.19–2.10 (1H, m, C-12H_AH_B, C-12'H_AH_B), 1.93–1.83 (3H, m, C-12H_AH_B, C-12'H_AH_B, C-13H_2, C-13'H_2), 0.93 (9H, t, *J* = 7.9 Hz, Si(CH_2CH_3)$_3$, Si(CH_2CH_3)$_3$'), 0.58 (6H, q, *J* = 8.1 Hz, Si(CH_2CH_3)$_3$, Si(CH_2CH_3)$_3$'); **^{13}C NMR:** δ_C (126 MHz, $CDCl_3$) 211.8* (C-10, C-10'), 144.9 (C-7, C-7'), 138.1 (2 × 2C, C-18, C-18', C-22, C-22'), 128.5 (2 × 4C, 2 × C-20, 2 × C-20', 2 × C-24, 2 × C-24'), 127.9 (2 × 2C, 2 × C-23, 2 × C-23'), 127.8 (4C, 2 × C-19, C-21, C-25), 112.6* (C-15, C-15'), 86.1 (C-4, C-4'), 83.9* (C-3, C-3'), 83.8 (C-2, C-2'), 83.5* (C-11, C-11'), 71.9 (C-17, C-17'), 71.4 (2 × 2C, C-1, C-1', C-16, C-16'), 70.8 (C-5, C-5'), 69.5 (C-14, C-14'), 41.0* (C-6, C-6'), 36.7 (C-9, C-9'), 29.8* (C-8, C-8'), 29.2* (C-12, C-12'), 25.8 (C-13, C-13'), 7.1 (2 × 3C, Si(CH_2CH_3)$_3$, Si(CH_2CH_3)$_3$'), 5.3 (2 × 3C, Si(CH_2CH_3)$_3$, Si(CH_2CH_3)$_3$'); **HRMS:** (ESI+, *m/z*) $C_{35}H_{50}O_6{}^{28}SiNa$ (MNa$^+$) Calculated: 617.32689, Found: 617.32644 (Δ –0.73 ppm).

3-Methoxy-1-(tetrahydrofuran-2-yl)propan-1-one (S8)

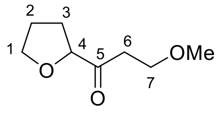

S8

A small amount of **S8** was isolated from the rhodium-catalysed 1,4-addition reactions in MeOH/H_2O with boronate **15** and enones **19** and **S6**.

R_f: 0.11 (70:30 pentane:Et_2O); **IR:** ν_{max} (thin film/cm^{-1}) 2954, 2929, 2876, 1718, 1456, 1390, 1227, 1118, 1075, 1020, 968, 933, 741, 700; **^{1}H NMR:** δ_H (400 MHz, $CDCl_3$) 4.33 (1H, dd, *J* = 8.4, 6.3 Hz, C-4H), 3.99–3.88 (2H, m, C-1H_2), 3.71–3.61 (2H, m, C-7H_2), 3.33 (3H, s, OCH_3), 2.87–2.72 (2H, m, C-6H_2), 2.23–2.11 (1H, m, C-3H_AH_B), 2.00–1.85 (3H, m, H-2, C-3H_AH_B); **^{13}C NMR:** δ_C (101 MHz, $CDCl_3$) 210.8 (C-5), 83.7 (C-4), 69.6 (C-1), 67.3 (C-7), 59.0 (OCH_3), 38.6 (C-6), 28.9 (C-3), 25.8 (C-2); **HRMS:** (ESI+, *m/z*) $C_8H_{14}O_3Na$ (MNa$^+$) Calculated: 181.08352, Found: 181.08349 (Δ –0.17 ppm).

(((*R*)-1-((2*S*,3*S*,4*R*)-3,4-Bis(benzyloxy)tetrahydrofuran-2-yl)but-3-en-1-yl)oxy)triethylsilane (S9)

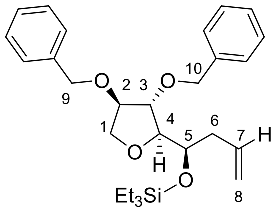

S9

A small amount of **S9** was isolated from the rhodium-catalysed 1,4-addition reactions with boronate **15**.

R_f: 0.66 (70:30 pentane:Et_2O); **IR:** ν_{max} (thin film/cm^{-1}) 3066, 3031, 2953, 2911, 2876, 1641, 1497, 1455, 1414, 1334, 1239, 1208, 1100, 1078, 1006, 913, 734, 697; **^{1}H NMR:** δ_H (400 MHz, $CDCl_3$) 7.39–7.23 (10H, m, ArH), 5.89 (1H, ddt, *J* = 17.3, 10.2, 7.1 Hz, C-

7H), 5.14–5.03 (2H, m, C-8H$_A$H$_B$), 4.60 (1H, d, *J* = 11.7 Hz, C-10*H$_A$*H$_B$), 4.52 (2H, s, C-9H$_2$), 4.51 (1H, d, *J* = 11.7 Hz, C-10H$_A$*H$_B$*), 4.15 (1H, ddd, *J* = 3.4, 1.5, 0.7 Hz, C-3H), 4.08–4.05 (1H, m, C-2H), 3.97 (1H, dd, *J* = 10.0, 2.2 Hz, C-1*H$_A$*H$_B$), 3.92 (1H, dd, *J* = 9.9, 4.3 Hz, C-1H$_A$*H$_B$*), 3.88 (1H, td, *J* = 5.1, 2.5 Hz, C-5H), 3.80 (1H, dd, *J* = 6.6, 3.3 Hz, C-4H), 2.35 (2H, m, C-6H$_A$H$_B$), 0.94 (9H, t, *J* = 7.9 Hz, Si(CH$_2$C*H$_3$*)$_3$), 0.60 (6H, q, *J* = 8.1 Hz, Si(C*H$_2$*CH$_3$)$_3$); **^{13}C NMR:** δ_C (101 MHz, CDCl$_3$) 138.2 (quaternary Ar), 138.1 (quaternary Ar), 134.5 (C-7), 130.9 (Ar), 128.5 (2C, Ar), 127.8 (5C, 3 Ar), 127.7 (2C, Ar), 117.6 (C-8), 85.8 (C-4), 84.3 (C-3), 83.6 (C-2), 71.7 (C-10), 71.6 (2C, C-1, C-5), 71.4 (C-9), 38.6 (C-6), 7.1 (3C, Si(CH$_2$*C*H$_3$)$_3$), 5.3 (3C, Si(*C*H$_2$CH$_3$)$_3$); **HRMS:** (ESI+, *m/z*) $C_{28}H_{40}O_4{}^{28}SiNa$ (MNa$^+$) Calculated: 491.25881, Found: 491.25856 (Δ –0.51 ppm).

((3*R*,5*S*)-3-((2*R*,3*S*,4*R*)-3,4-Bis(benzyloxy)tetrahydrofuran-2-yl)-1-((*R*)-tetrahydrofuran-2-yl)-2,8-dioxabicyclo[3.2.1]octan-5-yl)methyl 4-bromobenzoate (27)

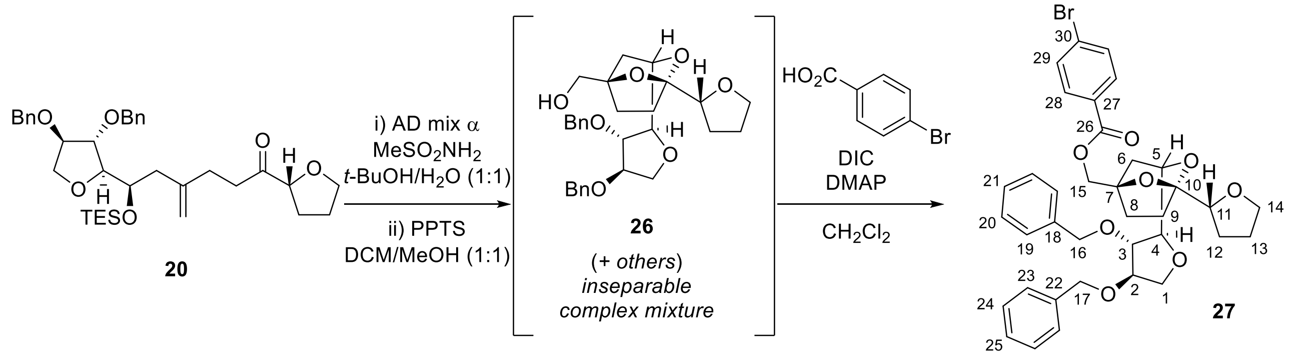

To a suspension of compound **20** (21 mg, 36 µmol), $K_3Fe(CN)_6$ (33 mg, 0.10 mmol), K_2CO_3 (14 mg, 0.10 mmol), methanesulfonamide (3.2 mg, 34 µmol), (DHQ)$_2$PHAL (5.2 mg, 6.7 µmol) in *t*-BuOH (0.5 mL) at 0 °C was added $K_2OsO_2(OH)_4$ (1.2 mg, 3.4 µmol) in H_2O (0.5 mL). The reaction was warmed to rt for 19 h before the reaction was quenched with sat. aq. Na_2SO_3 (1 mL) and stirred for a further 30 min. The mixture was extracted with EtOAc (3 × 2 mL). The combined organic layers were dried over $MgSO_4$, filtered and concentrated *in vacuo*. The crude diol product was obtained as a pale yellow oil and was carried into the next step without further purification.
Crude diol (assumed quant., 36 µmol) was transferred to a flask in CH_2Cl_2 (1 mL) and MeOH (1 mL). PPTS (9.7 mg, 39 mmol) was added and the reaction was stirred at rt for 22 h. The reaction was quenched with sat. aq. $NaHCO_3$ (2 mL), diluted with EtOAc (2 mL) and the layers were separated. The aq. layer was extracted with EtOAc (3 × 2 mL). The combined organic layers were dried over $MgSO_4$, filtered and concentrated *in vacuo*. The crude product was purified by column chromatography (SiO_2, 100:0 → 60:40 → 25:75 pentane:EtOAc) to afford an inseparable mixture of compounds including **26** (9.2 mg) as a colourless oil.
To the inseparable mixture of compounds including **26** (9.2 mg, 19 µmol) in CH_2Cl_2 (2.5 mL), were added 4-bromobenzoic acid (7.5 mg, 37 µmol), DMAP (1 crystal, cat.), and DIC (10 µL, 65 µmol). The reaction was stirred at rt for 28 h before the reaction was diluted with H_2O (3 mL) and CH_2Cl_2 (3 mL). The mixture was extracted with CH_2Cl_2 (3 × 5 mL). The combined organic layers were dried over $MgSO_4$, filtered and concentrated *in vacuo*. The crude product was purified by column chromatography (SiO_2, 100:0 → 90:10 → 80:20 pentane:EtOAc) to afford the title compound **27** (1.1 mg, 5% over three steps) as a colourless oil.

The bicyclic ring configuration was confirmed by the lack of nOes between C-5H to C-8H$_{A/B}$ and C-9H$_{A/B}$ in C_6D_6.

R$_f$: 0.67 (60:40 pentane:EtOAc); **[α]$_D^{25}$** +11.9 (*c* = 0.10, CHCl$_3$); **IR:** ν_{max} (thin film/cm^{-1}) 3064, 3028, 2946, 2870, 2360, 2338, 1722, 1651, 1590, 1496, 1484, 1454, 1398, 1368, 1271, 1207, 1173, 1102, 1070, 1028, 1012, 957, 903, 848, 830, 755, 738, 698, 638, 612; **^{1}H NMR:** δ_H (500 MHz, C_6D_6) 7.80–7.73 (2H, m, C-28H), 7.38–6.98 (12H, m ,C-29H, ArH), 4.56 (1H, d, *J* = 12.2 Hz, C-16*H$_A$*H$_B$), 4.51 (1H, d, *J* = 12.3 Hz, C-16H$_A$*H$_B$*), 4.33 (1H, d, *J* = 2.1 Hz, C-3H), 4.26 (1H, d, *J* = 11.7 Hz, C-15*H$_A$*H$_B$), 4.26 (1H, d, *J* = 11.9 Hz, C-17*H$_A$*H$_B$), 4.23–4.16 (1H, m, C-5H), 4.20 (1H, d, *J* = 11.9 Hz, C-17H$_A$*H$_B$*), 4.16 (1H, d, *J* = 11.7 Hz, C-15H$_A$*H$_B$*), 4.12–4.06 (2H, m, C-4H, C-11H), 3.98–3.86 (3H, m, C-1H$_2$, C-2H), 3.81–3.75 (1H, m, C-14*H$_A$*H$_B$), 3.64 (1H, m, C-14H$_A$*H$_B$*), 2.41 (1H, dd, *J* = 13.8, 5.5 Hz, C-6*H$_A$*H$_B$), 2.30 (1H, td, *J* = 12.5, 4.4 Hz, C-9*H$_A$*H$_B$), 2.09–1.99 (1H, m, C-12*H$_A$*H$_B$), 1.87 (1H, ddd, *J* = 12.7, 9.2, 5.1 Hz, C-9H$_A$*H$_B$*), 1.76–1.60 (4H, m, C-6H$_A$*H$_B$*, C-8*H$_A$*H$_B$, C-12H$_A$*H$_B$*, C-13*H$_A$*H$_B$), 1.47 (2H, m, C-8H$_A$*H$_B$*, C-13H$_A$*H$_B$*); **^{1}H NMR:** δ_H (500 MHz, CD_2Cl_2) 7.90–7.86 (2H, m, C-28H), 7.58–7.54 (2H, m, C-29H), 7.41–7.23 (10H, m, ArH), 4.57 (1H, d, *J* = 11.8 Hz, C-16*H$_A$*H$_B$), 4.56 (1H, d, *J* = 11.8 Hz, C-16H$_A$*H$_B$*), 4.50 (1H, d, *J* = 11.7 Hz, C-17*H$_A$*H$_B$), 4.48 (1H, d, *J* = 11.7 Hz, C-17H$_A$*H$_B$*), 4.42 (1H, d, *J* = 11.7 Hz, C-15*H$_A$*H$_B$), 4.31 (1H, d, *J* = 11.7 Hz, C-15H$_A$H$_B$), 4.09 (1H, d, *J* = 2.7 Hz, C-3H), 4.08–4.05 (1H, m, C-2H), 3.95–3.87 (4H, m, C-1H$_2$, C-5H, C-11H), 3.80 (1H, dd, *J* = 8.5, 2.7 Hz, C-4H), 3.77–3.70 (2H, m, C-14H$_2$), 2.32 (1H, dd, *J* = 13.4, 5.7 Hz, C-6*H$_A$*H$_B$), 2.04 (1H, td, *J* = 11.4, 3.9 Hz, C-9*H$_A$*H$_B$), 1.96 (1H, dd, *J* = 11.3, 3.6 Hz, C-8*H$_A$*H$_B$), 1.93–1.79 (5H, m, C-8H$_A$*H$_B$*, C-9H$_A$*H$_B$*, C-12H$_2$, C-13*H$_A$*H$_B$), 1.79–1.69 (1H, m, C-13H$_A$*H$_B$*), 1.73 (1H, dd, *J* = 13.4, 8.5 Hz, C-6H$_A$*H$_B$*); **^{13}C NMR**: δ_C (126 MHz, C_6D_6) 165.3 (C-26), 138.7 (C-18), 138.5 (C-22), 131.9 (2C, 2 × C-29), 131.4 (2C, 2 × C-28), 129.5 (Ar), 128.7 (Ar), 128.6 (2C, Ar), 128.4 (3C, 2Ar), 128.2 (2C, Ar), 128.0 (2C, Ar), 127.5 (Ar), 108.4 (C-10), 86.8 (C-4), 84.5 (C-3), 83.8 (C-2), 80.7 (C-7), 80.1 (C-11), 71.9 (C-1), 71.6 (C-16), 71.2 (C-17), 69.2 (C-14), 68.5 (C-5), 68.1 (C-15), 37.5 (C-6), 33.3 (C-9), 32.7 (C-8), 26.7 (C-12), 26.3 (C-13); **^{13}C NMR:** δ_C (126 MHz, CD_2Cl_2) 165.9 (C-26), 138.7 (C-18), 138.6 (C-22), 132.3 (2C, 2 × C-29), 131.7 (2C, 2 × C-28), 129.6 (Ar), 128.9 (4C, 2Ar), 128.5 (Ar), 128.4 (2C, Ar), 128.3 (2C, 2Ar), 128.2 (2C, Ar), 108.4 (C-10), 86.7 (C-4), 84.7 (C-3), 83.9 (C-2), 81.0 (C-7), 80.7 (C-11), 72.2 (C-1), 72.0 (C-16), 71.7 (C-17), 69.6 (C-14), 68.5 (C-15), 68.4 (C-5), 37.6 (C-6), 33.8 (C-9), 33.0 (C-8), 26.9 (C-12), 26.5 (C-13); **HRMS:** (ESI+, *m/z*) $C_{36}H_{39}O_8{}^{79}BrNa$ (MNa$^+$) Calculated: 701.17205, Found: 701.17162 (Δ –0.61 ppm).

((1*S*,3*R*,5*R*)-3-((2*R*,3*S*,4*R*)-3,4-Bis(benzyloxy)tetrahydrofuran-2-yl)-1-((*R*)-tetrahydrofuran-2-yl)-2,8-dioxabicyclo[3.2.1]octan-5-yl)methanol (23)

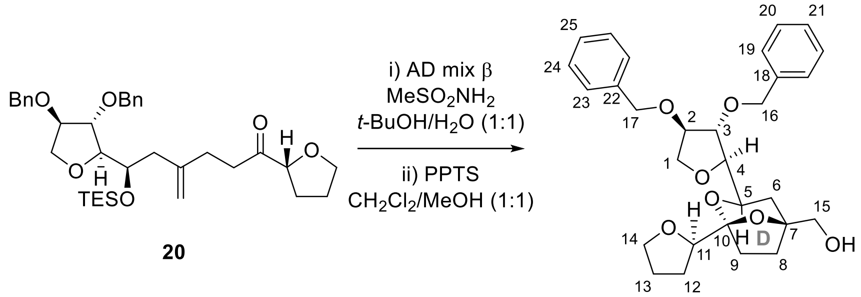

20 **23**

To a suspension of compound **20** (23 mg, 39 µmol), $K_3Fe(CN)_6$ (33 mg, 0.10 mmol), K_2CO_3 (14 mg, 0.10 mmol), methanesulfonamide (3.2 mg, 34 µmol), $(DHQD)_2PHAL$ (5.2 mg, 6.7 µmol) in *t*-BuOH (0.5 mL) at 0 °C was added $K_2OsO_2(OH)_4$ (1.2 mg, 3.4 µmol) in H_2O (0.5 mL). The reaction was warmed to rt for 20 h before the reaction was quenched with sat. aq. Na_2SO_3 (1 mL) and stirred for a further 3 h. The mixture was extracted with EtOAc (3 × 2 mL). The combined organic layers were dried over $MgSO_4$, filtered and concentrated *in vacuo*. The crude diol product was obtained as a pale yellow oil and was carried into the next step without further purification.
Crude diol (assumed quant., 39 µmol) was transferred to a flask in CH_2Cl_2 (1 mL) and MeOH (1 mL). PPTS (9.7 mg, 38.7 µmol) was added and the reaction was stirred at rt for 17 h. The reaction was quenched with sat. aq. $NaHCO_3$ (2 mL), diluted with EtOAc (2 mL) and the layers were separated. The aq. layer was extracted with EtOAc (3 × 2 mL). The combined organic layers were dried over $MgSO_4$, filtered and concentrated *in vacuo*. The crude product was purified by column chromatography (SiO_2, 100:0 → 60:40 → 50:50 → 40:60 → 25:75 → 0:100 pentane:EtOAc) to afford the title compound **23** (17.5 mg, 91%) as a colourless oil.

The bicyclic ring configuration was confirmed by nOes between C-5H to C-8H_B and C-9H_B in CD_2Cl_2.

R_f: 0.12 (60:40 pentane:EtOAc); **$[\alpha]_D^{25}$** +2.0 (*c* = 1.00, $CHCl_3$); **IR:** ν_{max} (thin film/cm^{-1}) 3543, 2919, 2871, 1496, 1455, 1327, 1210, 1076, 1028, 979, 908, 738, 699; **^{1}H NMR:** δ_H (500 MHz, C_6D_6) 7.38–7.32 (2H, m, ArH), 7.28–7.22 (2H, m, ArH), 7.22–7.06 (6H, m, ArH), 4.59 (1H, d, *J* = 12.0 Hz, C-16H_AH_B), 4.51 (1H, d, *J* = 12.1 Hz, C-16H_AH_B), 4.34 (1H, m, C-3H), 4.25 (2H, s, C-17H_2), 4.12–4.03 (3H, m, C-4H, C-5H, C-11H), 4.00–3.94 (2H, m, 2H, C-1H_AH_B, C-2H), 3.93–3.89 (1H, m, C-1H_AH_B), 3.72 (1H, dt, *J* = 8.2, 6.9 Hz, C-14H_AH_B), 3.61 (td, *J* = 7.6, 5.7 Hz, 1H, C-14H_AH_B), 3.54 (1H, d, *J* = 11.8 Hz, C-15H_AH_B), 3.35 (1H, d, *J* = 11.8 Hz, 1H, C-15H_AH_B), 2.02 (1H, td, *J* = 13.2, 4.6 Hz, C-9H_AH_B), 1.97–1.88 (2H, m, C-9H_AH_B, C-12H_AH_B), 1.84 (1H, tdd, *J* = 12.9, 4.9, 1.6 Hz, C-8H_AH_B), 1.81–1.72 (2H, m, C-6H_AH_B, C-12H_AH_B), 1.64–1.58 (1H, m, 1H, C-13H_AH_B), 1.55 (dd, *J* = 12.8, 3.4 Hz, C-6H_AH_B), 1.50–1.40 (1H, m, C-13H_AH_B), 1.27 (1H, ddd, *J* = 12.2, 9.4, 4.5 Hz, C-8H_AH_B); **^{1}H NMR:** δ_H (500 MHz, CD_2Cl_2) 7.45–7.06 (10H, m, ArH), 4.62–4.44 (4H, m, C-16H_2, C-17H_2), 4.12–4.04 (2H, m, C-2H, C-3H), 4.00–3.85 (4H, m, C-1H_2, C-5H, C-11H), 3.83–3.70 (3H, m, C-4H, C-14H_2), 3.63 (1H, d, *J* = 11.9 Hz, C-15H_AH_B), 3.51 (1H, d, *J* = 11.9 Hz, C-15H_AH_B), 2.01 (1H, dd, *J* = 13.4, 5.1 Hz, C-9H_AH_B), 1.97–1.74 (6H, m, C-8H_AH_B, C-9H_AH_B, C-12H_2, C-13H_2), 1.69–1.60 (2H, m, C-8H_AH_B, C-6H_AH_B), 1.51 (1H, dd, *J* = 12.9, 3.9 Hz, C-6H_AH_B); **^{13}C NMR:** δ_C (126 MHz, C_6D_6) 138.8 (C-18), 138.6 (C-22), 128.7 (2C, 2Ar), 128.4 (2C, Ar), 128.2 (2C, Ar), 128.0 (4C, 2Ar), 108.4 (C-10), 87.4 (C-4), 84.5 (C-3), 84.1 (C-2), 84.0 (C-7), 81.4 (C-11), 71.8 (C-1), 71.7 (C-16), 71.4 (C-17), 69.5 (C-5), 69.0 (C-14), 66.9 (C-15), 36.0 (C-6), 31.8 (C-9), 28.5 (C-8), 27.4 (C-12), 26.3 (C-13); **^{13}C NMR:** δ_C (126 MHz, CD_2Cl_2) 138.8 (C-18), 138.7 (C-22), 128.9 (4C, 2Ar), 128.3 (3C, 2Ar), 128.2 (3C, 2Ar), 108.8 (C-10), 87.3 (C-4), 84.5 (C-3), 84.1 (C-2), 83.9 (C-7), 81.0 (C-11), 72.2 (C-1), 72.1 (C-16), 71.8 (C-17), 69.5 (C-14), 69.3 (C-5), 67.3 (C-15), 35.6 (C-6), 31.0 (C-9), 28.9 (C-8), 27.8 (C-12), 26.5 (C-13); **HRMS:** (ESI+, *m/z*) $C_{29}H_{37}O_7$ (MH^+) Calculated: 497.25338, Found: 497.25380 (Δ +0.84 ppm).

((1*S*,3*R*,5*R*)-3-((2*R*,3*S*,4*R*)-3,4-Bis(benzyloxy)tetrahydrofuran-2-yl)-1-((*R*)-tetrahydrofuran-2-yl)-2,8-dioxabicyclo[3.2.1]octan-5-yl)methyl 4-bromobenzoate (28)

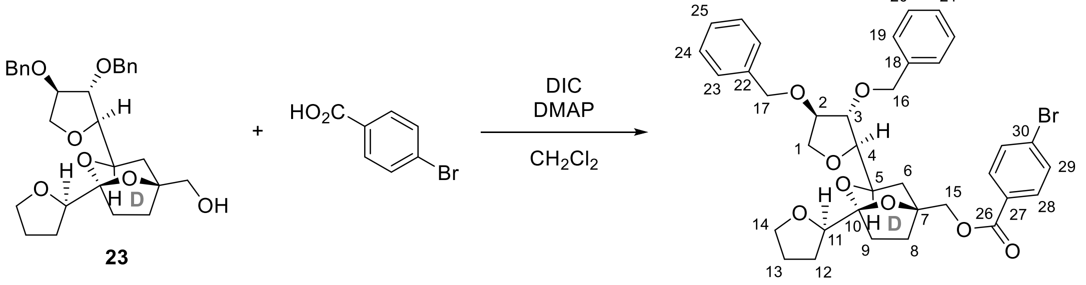

23 **28**

To compound **23** (10.8 mg, 21.7 µmol) in CH_2Cl_2 (2.5 mL), were added 4-bromobenzoic acid (8.8 mg, 44 µmol), DMAP (1 crystal, cat.), and DIC (6.7 µL, 43.5 µmol). The reaction was stirred at rt for 21 h before the reaction was diluted with H_2O (3 mL) and CH_2Cl_2 (3 mL). The mixture was extracted with CH_2Cl_2 (3 × 5 mL). The combined organic layers were dried over $MgSO_4$, filtered and

concentrated *in vacuo*. The crude product was purified by column chromatography (SiO_2, 100:0 → 80:20 pentane:EtOAc) to afford the title compound **28** (7.8 mg, 53%) as a colourless oil.

The bicyclic ring configuration was confirmed by nOes between C-5H to C-8H_B and C-9H_B in CD_2Cl_2.

***R**f*: 0.58 (60:40 pentane:EtOAc); **[*α*]$_D^{25}$** –1.6 (*c* = 0.50, $CHCl_3$); **IR:** ν_{max} (thin film/cm^{-1}) 2872, 1721, 1590, 1484, 1454, 1398, 1271, 1174, 1101, 1071, 1012, 956, 848, 756, 699; **^{1}H NMR:** δ_H (500 MHz, C_6D_6) 7.75–7.69 (2H, m, C-28H), 7.35–7.29 (2H, m, ArH), 7.27–7.21 (2H, m, ArH), 7.21–7.18 (3H, m, ArH), 7.14–7.08 (3H, m, ArH), 7.08–7.04 (2H, m, C-29H), 4.59 (1H, d, *J* = 12.1 Hz, C-16H_AH_B), 4.51 (1H, d, *J* = 12.0 Hz, C-16H_AH_B), 4.37 (1H, s, C-3H), 4.32 (1H, d, *J* = 11.7 Hz, C-15H_AH_B), 4.24 (2H, s, C-17H_2), 4.20 (1H, d, *J* = 11.7 Hz, C-15H_AH_B), 4.14–4.06 (3H, m, C-4H, C-5H, C-11H), 3.99 (1H, dt, *J* = 9.7, 1.0 Hz, C-1H_AH_B), 3.95 (1H, dt, *J* = 4.0, 1.3 Hz, C-2H), 3.93–3.87 (1H, dd, *J* = 9.7, 3.9 Hz, C-1H_AH_B), 3.75 (1H, dt, *J* = 8.1, 6.9 Hz, C-14H_AH_B), 3.62 (1H, td, *J* = 7.7, 5.7 Hz, C-14H_AH_B), 2.11–1.91 (4H, m, C-9H_2, C-6H_AH_B, C-12H_AH_B), 1.82–1.71 (2H, m, C-7H_AH_B, C-12H_AH_B), 1.69–1.60 (1H, m, C-13H_AH_B), 1.50–1.40 (2H, m, C-8H_AH_B, C-13H_AH_B), 1.33 (1H, ddd, *J* = 12.4, 9.2, 4.9 Hz, C-8H_AH_B); **^{1}H NMR:** δ_H (500 MHz, CD_2Cl_2) 7.94–7.87 (2H, m, C-28H), 7.62–7.55 (2H, m, C-29H), 7.39–7.21 (10H, m, ArH), 4.57 (1H, d, *J* = 11.8 Hz, C-16H_AH_B), 4.52 (1H, d, *J* = 11.8 Hz, C-17H_AH_B), 4.51 (1H, d, *J* = 11.8 Hz, C-16H_AH_B), 4.49 (1H, d, *J* = 11.8 Hz, C-17H_AH_B), 4.38 (1H, d, *J* = 11.7 Hz, C-15H_AH_B), 4.30 (1H, d, *J* = 11.7 Hz, C-15H_AH_B), 4.12 (1H, dt, *J* = 2.3, 1.0 Hz, C-3H), 4.08 (1H, dt, *J* = 3.9, 1.5 Hz, C-2H), 4.00–3.89 (4H, m, C-5H, C-11H, C-14H_2) 3.82–3.72 (3H, m, C-1H_2, C-4H), 2.08 (1H, ddd, *J* = 13.1, 10.3, 7.2 Hz, C-9H_AH_B), 1.95 (1H, ddd, *J* = 13.3, 7.8, 6.4 Hz, C-9H_AH_B), 1.90–1.73 (7H, m, C-8H_2, C-6H_AH_B, C-12H_2, C-13H_2), 1.70 (1H, dd, *J* = 12.9, 3.8 Hz, C-6H_AH_B); **^{13}C NMR:** δ_C (126 MHz, C_6D_6) 165.3 (C-26), 138.8 (C-18), 138.5 (C-22), 131.9 (2C, 2 × C-29), 131.5 (2C, 2 × C-28), 129.4 (Ar), 128.7 (2C, 2Ar), 128.4 (4C, 2Ar), 128.2 (2C, Ar), 128.0 (3C, 2Ar), 108.8 (C-10), 87.4 (C-4), 84.5 (C-3), 84.0 (C-2), 81.9 (C-7), 81.7 (C-11), 71.9 (C-1), 71.7 (C-16), 71.4 (C-17), 69.5 (C-5), 69.0 (C-14), 68.9 (C-15), 36.2 (C-6), 32.1 (C-9), 30.4 (C-8), 27.2 (C-12), 26.4 (C-13); **^{13}C NMR:** δ_C (126 MHz, CD_2Cl_2) 165.9 (C-26), 138.7 (C-18), 138.6 (C-22), 132.3 (2C, 2 × C-29), 131.7 (2C, 2 × C-28), 129.6 (quaternary Ar), 129.0 (2C, 2Ar), 128.9 (2C, Ar), 128.6 (quaternary Ar), 128.3 (2C, Ar), 128.2 (4C, 2Ar), 109.2 (C-10), 87.3 (C-4), 84.5 (C-3), 84.0 (C-2), 82.0 (C-7), 81.0 (C-11), 72.3 (C-16), 72.0 (C-17), 71.8 (C-14), 69.5 (C-1), 69.4 (C-5), 69.3 (C-15), 36.0 (C-6), 30.8 (C-8), 30.7 (C-9), 27.8 (C-12), 26.5 (C-13); **HRMS:** (ESI+, *m/z*) $C_{36}H_{40}O_8{}^{79}Br$ (MH^+) Calculated: 679.19011, Found: 679.19004 (Δ –0.10 ppm).

(*R*)-3-((2*R*,5*R*)-5-(Hydroxymethyl)-2-methyltetrahydrofuran-2-yl)-*N*-methoxy-*N*,2-dimethylpropanamide (S10)

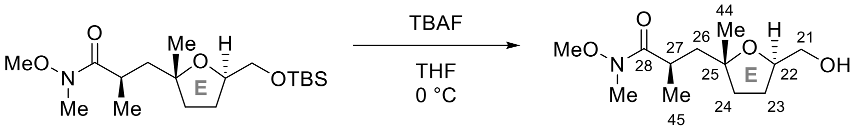

29 **S10**

To a stirred solution of **29** (503 mg, 1.40 mmol) in THF (6.75 mL) was added TBAF (1 M in THF, 70 µL, 69 µmol) at 0 °C. The reaction was stirred for 2 h 30 min before the reaction was quenched with sat. aq. NH_4Cl (15 mL). The mixture was extracted with EtOAc (3 × 20 mL). The combined organic layers were dried over $MgSO_4$, filtered and concentrated *in vacuo*. The crude product was purified by column chromatography (SiO_2, 100:0 → 98:2 CH_2Cl_2:MeOH) to afford the title compound **S10** (331 mg, 96%) as a colourless oil.

***R**f*: 0.56 (90:10 CH_2Cl_2:MeOH); **[*α*]$_D^{25}$** –33.2 (*c* = 1.00, $CHCl_3$); **IR:** ν_{max} (thin film/cm^{-1}) 3438, 2967, 2935, 2873, 16556, 1462, 1387, 1180, 1150, 1104, 1041, 996, 883, 747; **^{1}H NMR:** δ_H (400 MHz, $CDCl_3$) 4.09–3.99 (1H, m, C-22H), 3.71 (3H, s, OCH_3), 3.64 (1H, ddd, *J* = 15.2, 7.4, 4.1 Hz, C-21H_AH_B), 3.44 (1H, dt, *J* = 11.0, 5.2 Hz, C-21H_AH_B), 3.17 (3H, s, NCH_3), 3.14 (1H, s, C-27H), 2.15 (1H, dd, *J* = 14.1, 9.1 Hz, C-26H_AH_B), 2.09–2.01 (1H, m, OH), 1.96–1.84 (1H, m, C-23H_AH_B), 1.84–1.70 (2H, m, C-23H_AH_B, C-24H_AH_B), 1.70–1.58 (1H, m, C-24H_AH_B), 1.51 (1H, dd, *J* = 14.2, 3.5 Hz, C-26H_AH_B), 1.16 (3H, s, C-44H_3), 1.15 (3H, d, *J* = 7.0 Hz, C-45H_3); **^{13}C NMR:** δ_C (101 MHz, $CDCl_3$) 179.1 (C-28), 83.2 (C-25), 79.2 (C-22), 65.4 (C-21), 61.6 (OCH_3), 44.2 (C-26), 37.9 (C-24), 32.7 (NCH_3), 31.1 (C-27), 27.4 (C-23), 26.9 (C-44), 19.9 (C-45); **HRMS:** (ESI+, *m/z*) $C_{12}H_{23}O_4NNa$ (MNa^+) Calculated: 268.15193, Found: 268.15235 (Δ +1.57 ppm).

(2*R*)-3-((2*R*,5*R*)-5-(1-Hydroxyallyl)-2-methyltetrahydrofuran-2-yl)-*N*-methoxy-*N*,2-dimethylpropanamide (30)

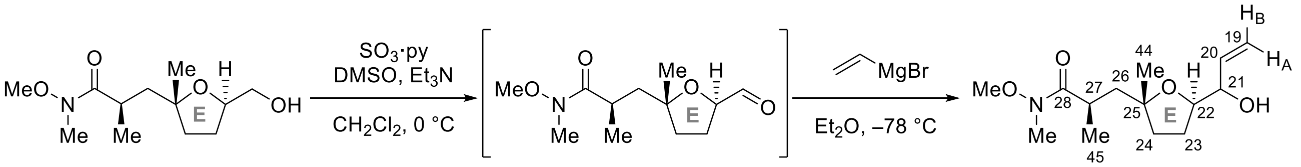

S10 **S11** **30**

To a solution of **S10** (100 mg, 0.41 mmol) and Et_3N (0.55 mL, 3.18 mmol) in CH_2Cl_2 (2 mL) at 0 °C was added SO_3·py (253 mg, 1.59 mmol) in DMSO (1.69 mL, 23.9 mmol). The reaction was warmed to rt and stirred for 2 h 20 min before it was diluted with a 1:1 mixture of hexane:EtOAc (15 mL). The solution was washed with 1 M aq. HCl (2 × 5 mL), sat. aq. $NaHCO_3$ (10 mL) and brine (10 mL). The aq. layer was saturated with NaCl, extracted with EtOAc (5 × 10 mL) and CH_2Cl_2 (3 × 20 mL). The combined organic layers were dried over $MgSO_4$, filtered and concentrated *in vacuo*. The crude product **S11** was obtained as a colourless oil and was carried into the next step without further purification.
Crude **S11** (assumed quant., 0.41 mmol) was transferred to a flask in anhydrous Et_2O (4.0 mL) and cooled to –78 °C. Vinyl magnesium bromide (1 M in THF, 0.45 mL, 0.45 mmol) was added at –78 °C and was stirred for 1 h. TLC analysis still indicated

incomplete reaction so additional vinyl magnesium bromide (1 M in THF, 0.45 mL, 0.45 mmol) was added and stirred for a further 40 min. The reaction was quenched with sat. aq. NH_4Cl (10 mL) and the layers were separated. The aq. layer was extracted with EtOAc (3 × 15 mL). The combined organic layers were dried over $MgSO_4$, filtered and concentrated *in vacuo*. The crude product was purified by column chromatography (SiO_2, 1:1 pentane:Et_2O) to afford the title compound **30** (76 mg, 69% over two steps) as a colourless oil. The diastereomeric ratio could not be determined at C-21.

R_f: 0.42 (90:10 CH_2Cl_2:MeOH); **IR:** ν_{max} (thin film/cm^{-1}) 3441, 2969, 2937, 1659, 1462, 1387, 1316, 1180, 1102, 1048, 996, 924, 746; **^{1}H NMR:** δ_H (400 MHz, $CDCl_3$) 5.83–5.69 (1H, m, C-20H), 5.31 (1H, dt, *J* = 17.3, 1.7 Hz, C-19H_AH_B), 5.16 (1H, dt, *J* = 10.6, 1.6 Hz, C-19H_AH_B), 4.23 (1H, dtd, *J* = 5.7, 2.9, 1.6 Hz, C-21H), 3.99–3.89 (1H, m, C-22H), 3.71 (3H, s, OCH_3), 3.16 (3H, s, NCH_3), 3.13 (1H, s, C-27H), 2.30 (d, *J* = 2.8 Hz, OH), 2.19– 2.13 (1H, dd, *J* = 14.3, 9.0 Hz, C-26H_AH_B), 1.94–1.80 (1H, m, C-23H_AH_B), 1.80–1.66 (2H, m, C-23H_AH_B, C-24H_AH_B), 1.66–1.56 (1H, m, C-24H_AH_B), 1.50 (1H, dd, *J* = 14.2, 3.6 Hz, C-26H_AH_B), 1.16 (3H, s, C-44H_3), 1.14 (3H, d, *J* = 7.0 Hz, C-45H_3); **^{13}C NMR:** δ_C (101 MHz, $CDCl_3$) 178.8 (C-28), 136.4 (C-20), 116.3 (C-19), 83.2 (C-25), 81.5 (C-22), 72.8 (C-21), 61.6 (OCH_3), 44.1 (C-26), 37.8 (C-24), 32.7 (NCH_3), 31.1 (C-27), 26.4 (C-44), 24.9 (C-23), 19.9 (C-45); **HRMS:** (ESI+, *m/z*) $C_{14}H_{26}O_4N$ (MH^+) Calculated: 272.18563, Found: 272.18497 (Δ –2.42 ppm).

(2*R*)-3-((2*R*,5*R*)-5-(1-((*tert*-butyldimethylsilyl)oxy)allyl)-2-methyltetrahydrofuran-2-yl)-*N*-methoxy-*N*,2-dimethylpropanamide (31)

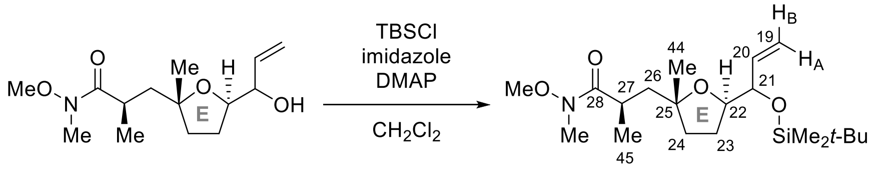

30 **31**

To a stirred solution of **30** (307 mg, 1.13 mmol) in CH_2Cl_2 (9.3 mL) was added imidazole (462 mg, 6.78 mmol), DMAP (13.8 mg, 0.11 mmol) and TBSCl (511 mg, 3.39 mmol) at rt. The reaction was stirred for 7 h before the reaction was quenched with H_2O (20 mL). The mixture was diluted with EtOAc (30 mL), separated and the aq. layer was extracted with EtOAc (3 × 25 mL). The combined organic layers were dried over Na_2SO_4, filtered and concentrated *in vacuo*. The crude product was purified by column chromatography (SiO_2, 100:0 → 85:15 pentane:EtOAc) to afford the title compound **31** (384 mg, 88%) as a colourless oil. The diastereomeric ratio could not be determined at C-21.

R_f: 0.61 (50:50 pentane:EtOAc); **IR:** ν_{max} (thin film/cm^{-1}) 2958, 2931, 2856, 2363, 2335, 1670, 1463, 1409, 1387, 1322, 1253, 1219, 1161, 1105, 1064, 1027, 1002, 938, 922, 837, 775, 679, 669; **^{1}H NMR:** δ_H (400 MHz, $CDCl_3$) 5.81 (1H, ddd, *J* = 16.4, 10.4, 5.5 Hz, C-20H), 5.22 (1H, d, *J* = 17.2 Hz, C-19H_AH_B), 5.08 (1H, d, *J* = 10.5 Hz, C-19H_AH_B), 4.11 (1H, t, *J* = 4.8 Hz, C-21H), 3.79 (1H, td, *J* = 7.2, 4.4 Hz, C-22H), 3.72 (3H, s, OCH_3), 3.17 (3H, s, NCH_3), 3.12 (1H, s, C-27H), 2.07 (1H, dd, *J* = 14.0, 8.6 Hz, C-26H_AH_B), 1.94–1.73 (2H, m, C-23H_2), 1.70–1.55 (2H, m, C-24H_2), 1.49 (1H, dd, *J* = 14.1, 3.8 Hz, C-26H_AH_B), 1.14 (3H, s, C-44H_3), 1.14 (3H, d, *J* = 7.4 Hz, C-45H_3), 0.89 (9H, s, $Si(CH_3)_2C(CH_3)_3$), 0.05 (3H, s, $Si(CH_3)(CH_3)C(CH_3)_3$), 0.02 (3H, s, $Si(CH_3)(CH_3)C(CH_3)_3$); **^{13}C NMR:** δ_C (101 MHz, $CDCl_3$) 171.2 (C-28), 139.3 (C-20), 114.9 (C-19), 82.9 (C-25), 82.1 (C-22), 75.2 (C-21), 61.5 (OCH_3), 44.0 (C-26), 38.0 (C-24), 32.6 (NCH_3), 31.4 (C-27), 26.2 (C-44), 26.2 (C-23), 26.0 (3C, $Si(CH_3)_2C(CH_3)_3$), 19.8 (C-11), 18.3 ($Si(CH_3)_2C(CH_3)_3$), –4.3 ($Si(CH_3)(CH_3)C(CH_3)_3$), –4.5 ($Si(CH_3)(CH_3)C(CH_3)_3$); **HRMS:** (ESI+, *m/z*) $C_{20}H_{39}O_4N^{28}SiNa$ (MNa^+) Calculated: 408.25406, Found: 408.25379 (Δ –0.66 ppm).

Ethyl (4*R*,*E*)-5-((2*R*,5*R*)-5-(1-((*tert*-butyldimethylsilyl)oxy)allyl)-2-methyltetrahydrofuran-2-yl)-2,4-dimethylpent-2-enoate (33)

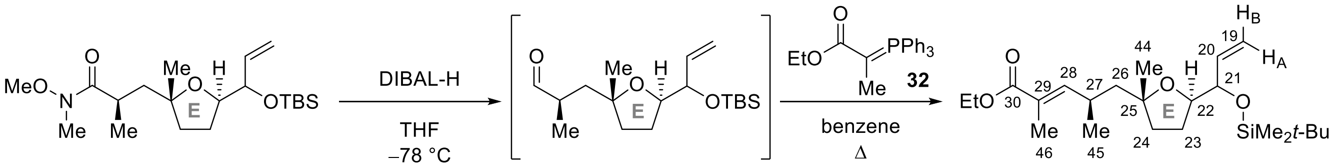

31 **S12** **33**

To a solution of **31** (952 mg, 2.47 mmol) in THF (45 mL) at –78 °C was added DIBAL-H (1 M in THF, 12.3 mL, 12.3 mmol) dropwise. The reaction was stirred for 2 h 30 min. TLC analysis indicated incomplete reaction so additional DIBAL-H (1 M in THF, 5.0 mL, 5.0 mmol) was added and stirred for a further 1 h. TLC analysis still indicated incomplete reaction so additional DIBAL-H (1 M in THF, 5 mL, 5 mmol) was added and stirred for a further 1 h. The reaction was quenched with EtOAc (90 mL) and warmed to rt before sat. aq. Rochelle's salt (potassium sodium tartrate, 100 mL) was added and the layers were separated. The aq. layer was extracted with EtOAc (5 × 100 mL). The combined organic layers were washed with H_2O (100 mL), dried over Na_2SO_4, filtered and concentrated *in vacuo*. The crude product **S12** was obtained as a colourless oil and was carried into the next step without further purification.
Crude **S12** (assumed quant., 2.47 mmol) was dried azeotropically in benzene (3 × 5 mL). To a 2-necked flask equipped with a condenser, was dissolved (carboxyethylidene)triphenylphosphorane **32** (3.13 g, 8.64 mmol) in benzene (56 mL). Crude **S12** was added as a solution in THF (12.2 mL) and the reaction was heated to reflux and stirred for 15 h. The reaction was cooled to rt before it was quenched with sat. aq. NH_4Cl (60 mL). The mixture was diluted with EtOAc (75 mL) and the layers were separated. The aq. layer was extracted with EtOAc (3 × 75 mL). The combined organic layers were dried over $MgSO_4$, filtered and concentrated *in vacuo*. The crude product was purified by column chromatography (SiO_2, 100:0 → 97:3 → 95:5 pentane:Et_2O) to afford the title compound

33 (877 mg, 87% over two steps) as a colourless oil. The diastereomeric ratio could not be determined at C-21. The diastereoisomers are identified by prime notation.

Aldehyde **S12** is identified by the presence of ^{1}H NMR (200 MHz, $CDCl_3$) 9.39 ppm (*J* = 4.0 Hz) for the aldehyde proton. Epimerisation at the α-keto stereocentre is diagnosed by the presence of ^{1}H NMR (200 MHz, $CDCl_3$) 9.57 (*J* = 3.2 Hz, epimer of **S12**) and 9.39 ppm (*J* = 4.0 Hz) for the aldehyde protons.

The (*E*)-geometry of the double bond was confirmed by nOes between C-46H_3 to C-27H and C-45H_3 in $CDCl_3$.

R*f*: 0.40 (85:15 pentane:Et_2O); **IR:** ν_{max} (thin film/cm^{-1}) 2958, 2930, 2858, 1711, 1648, 1462, 1388, 1368, 1251, 1151, 1118, 1098, 106, 1028, 939, 922, 837, 776, 751, 671; **^{1}H NMR:** δ_H (500 MHz, $CDCl_3$) 6.63 (1H, dd, *J* = 6.5, 1.5 Hz, C-28'H), 6.61 (dd, *J* = 6.5, 1.5 Hz, C-28H), 5.79 (1H, ddd, *J* = 17.3, 10.5, 5.7 Hz, C-20H), 5.79 (1H, ddd, *J* = 17.3, 10.5, 5.6 Hz, C-20'H), 5.22 (1H, dq, *J* = 17.2, 1.9 Hz, C-19H_AH_B, C-19'H_AH_B), 5.08 (1H, dt, *J* = 10.5, 1.6 Hz, C-19H_AH_B), 5.07 (dt, *J* = 10.5, 1.6 Hz, C-19'H_AH_B), 4.18 (2H, q, *J* = 7.1 Hz, OCH_2CH_3, OCH_2CH_3'), 4.13–4.06 (1H, m, C-21H, C-21'H), 3.74 (1H, ddd, *J* = 10.8, 8.4, 4.4 Hz, C-22H, C-22'H), 2.74–2.61 (1H, m, C-27H, C-27'H), 1.94–1.85 (1H, m, C-23H_AH_B, C-23'H_AH_B), 1.85 (3H, d, *J* = 1.4 Hz, C-46H_3), 1.84 (3H, d, *J* = 1.4 Hz, C-46'H_3), 1.82–1.74 (1H, m, C-23H_AH_B, C-23'H_AH_B), 1.73–1.52 (4H, m, C-26H_2, C-26'H_2, C-24H_2, C-24'H_2), 1.29 (2H, t, *J* = 7.1 Hz, OCH_2CH_3, OCH_2CH_3'), 1.16 (3H, s, C-44H_3), 1.15 (3H, s, C-44'H_3), 1.01 (3H, d, *J* = 6.7 Hz, C-45H_3), 1.00 (3H, d, *J* = 6.8 Hz, C-45'H_3), 0.90 (9H, s, $Si(CH_3)_2C(CH_3)_3$), 0.89 (9H, s, $Si(CH_3)_2C(CH_3)_3$'), 0.06 (6H, s, $Si(CH_3)(CH_3)C(CH_3)_3$), 0.04 (6H, s, $Si(CH_3)(CH_3)C(CH_3)_3$'), 0.02 (3H, s, $Si(CH_3)(CH_3)C(CH_3)_3$), 0.01 (3H, s, $Si(CH_3)(CH_3)C(CH_3)_3$'); **^{13}C NMR:** δ_C (126 MHz, $CDCl_3$) 168.7 (C-30, C-30'), 149.5 (C-28'), 149.2 (C-28), 139.4 (C-20, C-20'), 125.0 (C-29), 124.7 (C-29'), 115.1 (C-19), 115.0 (C-19'), 83.2 (C-25), 83.0 (C-25'), 82.4 (C-22'), 82.2 (C-22), 75.4 (C-21), 75.2 (C-21'), 60.5 (OCH_2CH_3, OCH_2CH_3'), 48.0 (C-26, C-26'), 37.8 (C-24), 37.5 (C-24'), 30.3 (C-27), 30.1 (C-27'), 27.3 (C-44'), 26.9 (C-44), 26.5 (C-23), 26.4 (C-23'), 26.1 (3C, 3C', $Si(CH_3)_2C(CH_3)_3$, $Si(CH_3)_2C(CH_3)_3$'), 21.7 (C-45'), 21.6 (C-45), 18.4 ($Si(CH_3)_2C(CH_3)_3$, $Si(CH_3)_2C(CH_3)_3$'), 14.5 (OCH_2CH_3, OCH_2CH_3'), 12.5 (C-46, C-46'), –4.3 ($Si(CH_3)(CH_3)C(CH_3)_3$, $Si(CH_3)(CH_3)C(CH_3)_3$'), –4.4 ($Si(CH_3)(CH_3)C(CH_3)_3$), –4.5 ($Si(CH_3)(CH_3)C(CH_3)_3$'); **HRMS:** (ESI+, *m/z*) $C_{23}H_{42}O_4{}^{28}SiNa$ (MNa^+) Calculated: 433.27446, Found: 433.27433 (Δ –0.30 ppm).

Ethyl (4*R*,*E*)-5-((2*R*,5*R*)-5-(1-hydroxyallyl)-2-methyltetrahydrofuran-2-yl)-2,4-dimethylpent-2-enoate (34)

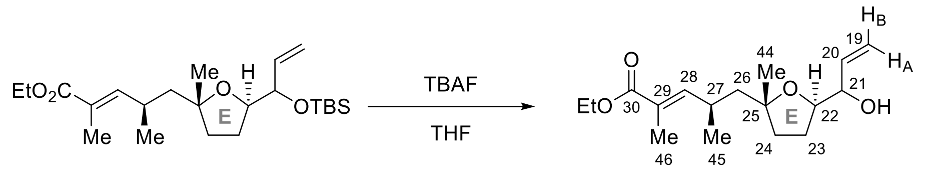

33 **34**

To a stirred solution of **33** (877 mg, 2.14 mmol) in THF (85 mL) was added TBAF (1 M in THF, 6.95 mL, 6.95 mmol) at rt. The reaction was stirred for 8 h at rt. TLC analysis indicated incomplete reaction so additional TBAF (1 M in THF, 6.95 mL, 6.95 mmol) was added and stirred for a further 18 h. The reaction was quenched with sat. aq. NH_4Cl (100 mL). The mixture was extracted with EtOAc (3 × 100 mL). The combined organic layers were dried over $MgSO_4$, filtered and concentrated *in vacuo*. The crude product was purified by column chromatography (SiO_2, 100:0 → 85:15 pentane:EtOAc) to afford the title compound **34** (494 mg, 78%) as a colourless oil. The diastereomeric ratio could not be determined at C-21. The diastereoisomers are identified by prime notation.

R*f*: 0.21 (85:15 pentane:EtOAc); **IR:** ν_{max} (thin film/cm^{-1}) 3466, 2963, 2927, 2871, 2362, 2340, 1709, 1647, 1456, 1372, 1273, 1245, 1098, 1046, 996, 924, 751, 668; **^{1}H NMR:** δ_H (500 MHz, $CDCl_3$) 6.64 (1H, dd, *J* = 10.0, 1.4 Hz, C-28H'), 6.64 (1H, dd, *J* = 10.1, 1.5 Hz, C-28H), 5.78 (1H, ddd, *J* = 16.8, 10.7, 5.8 Hz, C-20, C-20'), 5.32 (1H, dq, *J* = 17.4, 1.8 Hz, C-19H_AH_B, C-19'H_AH_B), 5.19 (1H, dt, *J* = 10.7, 1.5 Hz, C-19H_AH_B, C-19'H_AH_B), 4.28–4.17 (1H, m, C-21H, C-21'H), 4.18 (2H, qd, *J* = 7.1, 1.5 Hz, OCH_2CH_3), 4.18 (2H, q, *J* = 7.1 Hz, OCH_2CH_3'), 3.93 (1H, ddd, *J* = 8.6, 5.7, 3.6 Hz, C-22'H), 3.88 (1H, ddd, *J* = 9.4, 5.7, 3.6 Hz, C-22H), 2.77–2.64 (1H, m, C-27H, C-27'H), 2.22 (1H, d, *J* = 3.0 Hz, OH'), 2.19 (1H, d, *J* = 2.8 Hz, OH), 1.94–1.84 (1H, m, C-23H_AH_B, C-23'H_AH_B), 1.86 (3H, d, *J* = 1.5 Hz, C-46H_3), 1.85 (3H, d, *J* = 1.4 Hz, C-46'H_3), 1.81–1.57 (5H, m, C-26H_2, C-26'H_2, C-24H_2, C-24'H_2, C-23H_AH_B, C-23'H_AH_B), 1.29 (3H, t, *J* = 7.1 Hz, OCH_2CH_3, OCH_2CH_3'), 1.19 (3H, s, C-44H_3, C-44'H_3), 1.03 (3H, d, *J* = 6.8 Hz, C-45H_3), 1.02 (3H, d, *J* = 6.9 Hz, C-45'H_3); **^{13}C NMR:** δ_C (126 MHz, $CDCl_3$) 168.7 (C-30'), 168.6 (C-30), 149.1 (C-28'), 148.8 (C-28), 136.4 (C-20, C-20'), 125.1 (C-29), 124.9 (C-29'), 116.5 (C-19, C-19'), 83.5 (C-25), 83.4 (C-25'), 81.6 (C-22'), 81.5 (C-22), 72.9 (C-21, C-21'), 60.6 (OCH_2CH_3, OCH_2CH_3'), 48.0 (C-26), 47.8 (C-26'), 37.8 (C-24), 37.7 (C-24'), 30.2 (C-27), 30.0 (C-27'), 27.1 (C-44'), 26.9 (C-44), 25.2 (C-23, C-23'), 21.7 (C-45'), 21.5 (C-45), 14.5 (OCH_2CH_3, OCH_2CH_3'), 12.5 (C-46, C-46'); **HRMS:** (ESI+, *m/z*) $C_{17}H_{28}O_4Na$ (MNa^+) Calculated: 319.18798, Found: 319.18760 (Δ –1.19 ppm).

Ethyl (*R*,*E*)-5-((2*R*,5*R*)-5-acryloyl-2-methyltetrahydrofuran-2-yl)-2,4-dimethylpent-2-enoate (35)

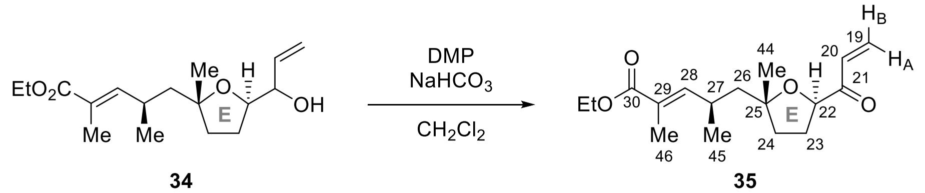

34 **35**

To a solution of **34** (62.8 mg, 0.212 mmol) in CH_2Cl_2 (from Winchester, 2.4 mL) was added $NaHCO_3$ (89 mg, 1.06 mmol) and DMP (180 mg, 0.424 mmol) and the reaction was stirred at rt for 3 h. The reaction was quenched with sat. aq. $NaHCO_3$ (10 mL) and the layers were separated. The aq. layer was extracted with CH_2Cl_2 (3 × 10 mL). The combined organic layers were washed with sat. aq. $Na_2S_2O_3$ (2 × 10 mL), dried over $MgSO_4$, filtered and concentrated *in vacuo*. The crude product was purified by column chromatography (SiO_2, 100:0 → 90:10 pentane:EtOAc) to afford the title compound **35** (56 mg, 90%) as a colourless oil.

R_f: 0.35 (85:15 pentane:EtOAc); **$[\alpha]_D^{25}$** –5.6 (*c* = 1.00, $CHCl_3$); **IR:** ν_{max} (thin film/cm^{-1}) 2970, 2930, 2872, 1774, 1708, 1648, 1613, 1454, 1401, 1368, 1271, 1245, 1176, 1143, 1095, 1071, 1000, 937, 858, 752; **^{1}H NMR:** δ_H (400 MHz, $CDCl_3$) 6.74 (1H, dd, *J* = 17.5, 10.6 Hz, C-20H), 6.64 (1H, dq, *J* = 10.1, 1.5 Hz, C-28H), 6.37 (1H, dd, *J* = 17.5, 1.8 Hz, C-19H_AH_B), 5.78 (1H, dd, *J* = 10.6, 1.7 Hz, C-19H_AH_B), 4.46 (1H, t, *J* = 7.8 Hz, C-22H), 4.18 (2H, qd, *J* = 7.1, 1.4 Hz, OCH_2CH_3), 2.80–2.67 (1H, m, C-27H), 2.20 (1H, dtd, *J* = 12.9, 7.8, 5.1 Hz, C-23H_AH_B), 2.05 (1H, dq, *J* = 12.7, 8.1 Hz, C-23H_AH_B), 1.86 (3H, d, *J* = 1.4 Hz, C-46H_3), 1.82–1.59 (4H, m, C-26H_2, C-24H_2), 1.28 (3H, t, *J* = 7.1 Hz, OCH_2CH_3), 1.22 (3H, s, C-44H_3), 1.04 (3H, d, *J* = 6.8 Hz, C-45H_3); **^{13}C NMR:** δ_C (101 MHz, $CDCl_3$) 200.6 (C-21), 168.6 (C-30), 148.6 (C-28), 132.0 (C-20), 129.6 (C-19), 125.3 (C-29), 85.3 (C-25), 82.3 (C-22), 60.6 (OCH_2CH_3), 48.1 (C-26), 37.4 (C-24), 30.2 (C-27), 29.4 (C-23), 26.5 (C-44), 21.5 (C-45), 14.5 (OCH_2CH_3), 12.5 (C-46); **HRMS:** (ESI+, *m/z*) $C_{17}H_{26}O_4Na$ (MNa^+) Calculated: 317. 17233, Found: 317.17228 (Δ –0.16 ppm).

Ethyl (*R*,*E*)-5-((2*R*,5*R*)-5-((*R*)-6-((2*S*,3*S*,4*R*)-3,4-bis(benzyloxy)tetrahydrofuran-2-yl)-4-methylene-6-((triethylsilyl)oxy)hexanoyl)-2-methyltetrahydrofuran-2-yl)-2,4-dimethylpent-2-enoate (36)

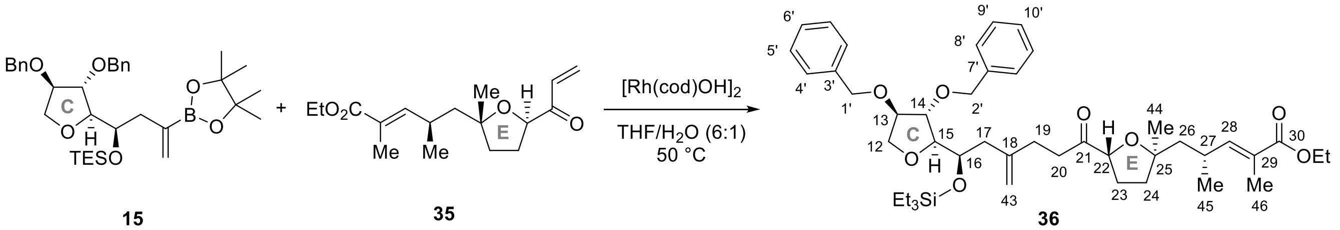

To compound **15** (33.6 mg, 56.6 μmol) and $[Rh(cod)OH]_2$ (3.9 mg, 8.5 μmol) in a vial was added enone **35** (20 mg, 67.9 μmol) in THF/H_2O (6:1, 0.24 mL) and the vial was flushed with argon. The reaction heated to 50 °C for 24 h. The reaction was cooled to rt and EtOAc (1 mL) and H_2O (1 mL) were added and the layers were separated. The aq. layer was extracted with EtOAc (3 × 2 mL). The combined organic layers were dried over $MgSO_4$, filtered and concentrated *in vacuo*. The crude product was purified by column chromatography (SiO_2, 100:0 → 95:5 → 90:10 → 80:20 pentane:EtOAc) to afford the title compound **36** (22.9 mg, 53%) as a colourless oil.

R_f: 0.48 (80:20 pentane:EtOAc); **$[\alpha]_D^{25}$** –7.1 (*c* = 1.00, $CHCl_3$); **IR:** ν_{max} (thin film/cm^{-1}) 3438, 2970, 1708, 1647, 1454, 1369. 1251, 1095, 751, 699; **^{1}H NMR:** δ_H (500 MHz, $CDCl_3$) 7.39–7.24 (10H, m, ArH), 6.62 (1H, dq, *J* = 10.1, 1.4 Hz, C-28H), 4.82 (1H, s, C-43H_AH_B), 4.77 (1H, q, *J* = 1.6 Hz, C-43H_AH_B), 4.59 (1H, d, *J* = 11.6 Hz, C-2'H_AH_B), 4.55 (1H, d, *J* = 11.7 Hz, C-2'H_AH_B), 4.53 (1H, d, *J* = 12.4 Hz, C-1'H_AH_B), 4.49 (1H, d, *J* = 12.0 Hz, C-1'H_AH_B), 4.25 (1H, t, *J* = 7.8 Hz, C-22H), 4.21–4.14 (3H, m, C-3H, OCH_2CH_3), 4.08 (1H, dt, *J* = 4.6, 2.3 Hz, C-13H), 4.04–3.99 (1H, m, C-16H), 3.95 (1H, dd, *J* = 9.9, 2.4 Hz, C-12H_AH_B), 3.89 (1H, dd, *J* = 9.9, 4.8 Hz, C-12H_AH_B), 3.76 (1H, t, *J* = 4.5 Hz, C-15H), 2.77–2.64 (3H, m, C-20H_2, C-27H), 2.36–2.24 (3H, m, C-17H_AH_B, C-19H_2), 2.24–2.10 (2H, m, C-17H_AH_B, C-23H_AH_B), 1.99–1.88 (1H, m, C-23H_AH_B), 1.86 (3H, d, *J* = 1.5 Hz, C-46H_3), 1.76–1.66 (2H, m, C-24H_AH_B, C-26H_AH_B), 1.66–1.58 (2H, m, C-24H_AH_B, C-26H_AH_B), 1.28 (3H, t, *J* = 7.1 Hz, OCH_2CH_3), 1.21 (3H, s, C-44H_3), 1.04 (3H, d, *J* = 6.7 Hz, C-45H_3), 0.92 (9H, t, *J* = 8.0 Hz, $Si(CH_2CH_3)_3$), 0.58 (6H, q, *J* = 8.0 Hz, $Si(CH_2CH_3)_3$); **^{13}C NMR:** δ_C (126 MHz, $CDCl_3$) 211.9 (C-21), 168.5 (C-30), 148.6 (C-28), 145.0 (C-18), 138.2 (C-7'), 138.1 (C-3'), 128.5 (4C, 3Ar), 127.9 (2C, Ar), 127.8 (4C, 2Ar), 125.3 (C-29), 112.4 (C-43), 86.1 (C-15), 85.1 (C-25), 83.9 (C-14), 83.8 (C-13), 83.3 (C-22), 71.9 (C-2'), 71.4 (2C, C-12, C-1'), 70.8 (C-16), 60.6 (OCH_2CH_3), 47.9 (C-4), 41.1 (C-17), 37.6 (C-24), 36.6 (C-20), 30.2 (C-27), 29.8 (C-19), 29.3 (C-23), 26.4 (C-44), 21.5 (C-45), 14.5 (OCH_2CH_3), 12.5 (C-46), 7.1 (3C, $Si(CH_2CH_3)_3$), 5.3 (3C, $Si(CH_2CH_3)_3$); **HRMS:** (ESI+, *m/z*) $C_{45}H_{66}O_8{}^{28}SiNa$ (MNa^+) Calculated: 785.44192, Found: 785.44164 (Δ –0.36 ppm).

Ethyl (*R*,*E*)-5-((2*R*,5*R*)-5-((1*S*,3*R*,5*R*)-3-((2*R*,3*S*,4*R*)-3,4-bis(benzyloxy)tetrahydrofuran-2-yl)-5-(hydroxymethyl)-2,8-dioxabicyclo[3.2.1]octan-1-yl)-2-methyltetrahydrofuran-2-yl)-2,4-dimethylpent-2-enoate (37)

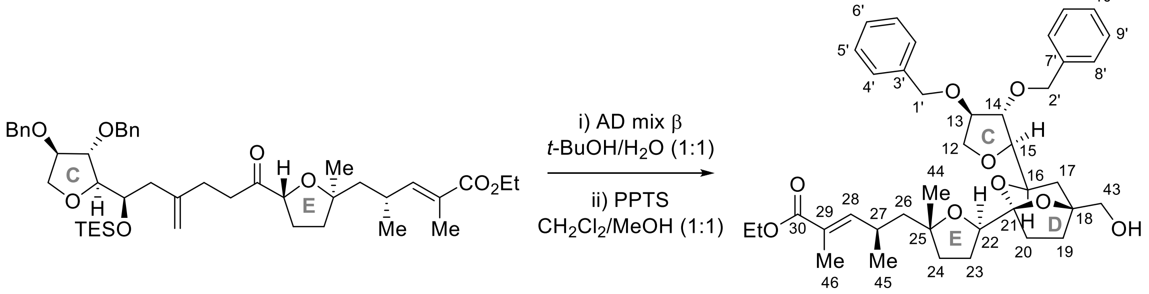

To a suspension of compound **36** (11.6 mg, 15.2 μmol), $K_3Fe(CN)_6$ (14.9 mg, 45.6 μmol), K_2CO_3 (6.3 mg, 46 μmol), $(DHQD)_2PHAL$ (2.4 mg, 3.0 μmol) in *t*-BuOH (0.5 mL) at 0 °C was added $K_2OsO_2(OH)_4$ (0.6 mg, 1.5 μmol) in H_2O (0.5 mL). The reaction was stirred and monitored closely for 1 h 25 min before the reaction was quenched with sat. aq. Na_2SO_3 (1 mL) and stirred for a further 15 min. The mixture was extracted with EtOAc (3 × 2 mL). The combined organic layers were dried over $MgSO_4$, filtered and concentrated *in vacuo*. The crude diol product was obtained as a pale yellow oil and was carried into the next step without further purification. Crude diol (assumed quant., 15.2 μmol) was transferred to a flask in CH_2Cl_2 (1 mL) and MeOH (1 mL). PPTS (3.8 mg, 15.2 μmol) was added and the reaction was stirred at rt for 17 h. The reaction was quenched with sat. aq. $NaHCO_3$ (2 mL), diluted with EtOAc (2 mL) and the layers were separated. The aq. layer was extracted with EtOAc (3 × 2 mL). The combined organic layers were dried over $MgSO_4$, filtered and concentrated *in vacuo*. The crude product was purified by column chromatography (SiO_2, 100:0 → 90:10 → 80:20 → 70:30 → 60:40 → 50:50 → 0:100 pentane:EtOAc) to afford the title compound **37** (4.0 mg, 40%) as a colourless oil.

The bicyclic ring configuration was confirmed by nOes between C-16H to C-19H_B and C-20H_B in $CDCl_3$.

R_f: 0.14 (50:50 pentane:EtOAc); **$[\alpha]_D^{25}$** –3.4 (*c* = 0.50, $CHCl_3$); **IR:** ν_{max} (thin film/cm^{-1}) 3734, 3690, 3475, 3030, 2962, 2926, 2361, 2342, 1706, 1648, 1497, 1455, 1369, 1272, 1246, 1177, 1095, 1028, 909, 751, 698, 669, 655, 636, 608; **^{1}H NMR:** δ_H (500 MHz, $CDCl_3$) 7.39–7.21 (10H, m, ArH), 6.63 (1H, dd, *J* = 10.1, 1.6 Hz, C-28H), 4.57 (1H, d, *J* = 11.9 Hz, C-2'H_AH_B), 4.51 (2H, s, C-1'H_2), 4.49 (1H, d, *J* = 11.7 Hz, C-2'H_AH_B), 4.18 (2H, q, *J* = 7.0 Hz, OCH_2CH_3), 4.15 (1H, s, C-14H), 4.09–4.06 (1H, m, C-13H), 3.99–3.92 (3H, m, C-12H_2, C-16H), 3.92–3.87 (1H, m, C-22H), 3.83–3.77 (1H, m, C-15H), 3.66 (1H, d, *J* = 12.0 Hz, C-9H_AH_B), 3.55 (1H, d, *J* = 11.9 Hz, C-43H_AH_B), 2.73–2.62 (1H, m, C-27H), 2.12–2.03 (1H, m, C-20H_AH_B), 1.99–1.77 (7H, m, C-19H_AH_B, C-20H_AH_B, C-23H_2, C-46H_3), 1.71–1.50 (7H, m, C-17H_2, C-19H_AH_B, C-24H_2, C-26H_2), 1.29 (3H, t, *J* = 7.1 Hz, OCH_2CH_3), 1.15 (3H, s, C-44H_3), 1.01 (3H, d, *J* = 6.7 Hz, C-45H_3); **^{1}H NMR:** δ_H (500 MHz, C_6D_6) 7.38–7.08 (10H, m, ArH), 6.94–6.90 (1H, m, C-28H), 4.63 (1H, d, *J* = 12.1 Hz, C-2'H_AH_B), 4.56 (1H, d, *J* = 12.1 Hz, C-2'H_AH_B), 4.43 (1H, s, C-14H), 4.29 (d, *J* = 11.6 Hz, C-1'H_AH_B), 4.27 (1H, d, *J* = 11.7 Hz, C-1'H_AH_B), 4.14–4.04 (4H, m, C-15H, C-16H, OCH_2CH_3), 4.03–3.93 (4H, m, C-12H_2, C-13H, C-22H), 3.45 (1H, d, *J* = 11.7 Hz, C-43H_AH_B), 3.31 (1H, d, *J* = 11.7 Hz, C-43H_AH_B), 2.69–2.58 (1H, m, C-27H), 2.14–2.03 (2H, m, C-20H_AH_B, C-23H_AH_B), 2.00–1.93 (1H, m, C-20H_AH_B), 1.91 (3H, d, *J* = 1.5 Hz, C-46H_3), 1.90–1.71 (3H, m, 1H, C-17H_AH_B, C-19H_AH_B, C-23H_AH_B), 1.57–1.38 (5H, m, C-17H_AH_B, C-24H_2, C-26H_2), 1.27–1.19 (1H, m, C-19H_AH_B), 1.16 (3H, s, C-44H_3), 1.02 (3H, t, *J* = 7.1 Hz, OCH_2CH_3), 0.89 (3H, d, *J* = 6.7 Hz, C-45H_3); **^{13}C NMR:** δ_C (126 MHz, $CDCl_3$) 168.7 (C-30), 149.0 (C-28), 138.1 (C-7'), 137.9 (C-3'), 128.6 (4C, 2Ar), 128.0 (Ar), 127.9 (3C, 2Ar), 127.8 (2C, Ar), 125.0 (C-29), 108.0 (C-21), 87.0 (C-15), 83.9 (C-18), 83.7 (C-25), 83.6 (C-14), 83.3 (C-13), 80.5 (C-22), 71.9 (C-12), 71.7 (C-2'), 71.4 (C-1'), 68.8 (C-16), 66.9 (C-43), 60.6 (OCH_2CH_3), 48.0 (C-26), 37.4 (C-24), 35.5 (C-17), 30.5 (C-20), 30.2 (C-27), 28.4 (C-19), 27.6 (C-23), 27.0 (C-44), 21.6 (C-45), 14.5 (OCH_2CH_3), 12.5 (C-46); **^{13}C NMR:** δ_C (126 MHz, C_6D_6) 168.2 (C-30), 149.2 (C-28), 138.9 (C-7'), 138.6 (C-3'), 128.7 (2C, 2Ar), 128.4 (3C, Ar), 128.2 (2C, Ar), 128.0 (2C, Ar), 127.5 (Ar), 125.3 (C-29), 107.9 (C-21), 87.5 (C-15), 84.4 (C-14), 83.9 (2C, C-13, C-25), 83.4 (C-18), 83.1 (C-22), 81.7 (C-12), 71.9 (C-2'), 71.7 (C-1'), 71.4 (C-16), 69.4 (C-43), 60.4 (OCH_2CH_3), 48.1 (C-26), 37.8 (C-24), 36.0 (C-17), 32.0 (C-20), 30.2 (C-27), 28.7 (C-19), 27.5 (C-23), 27.4 (C-44), 21.4 (C-45), 14.4 (OCH_2CH_3), 12.6 (C-46); **HRMS:** (ESI+, *m/z*) $C_{39}H_{52}O_9Na$ (MNa^+) Calculated: 687.35035, Found: 687.35055 (Δ +0.29 ppm).

III) ^{1}H and ^{13}C NMR spectra

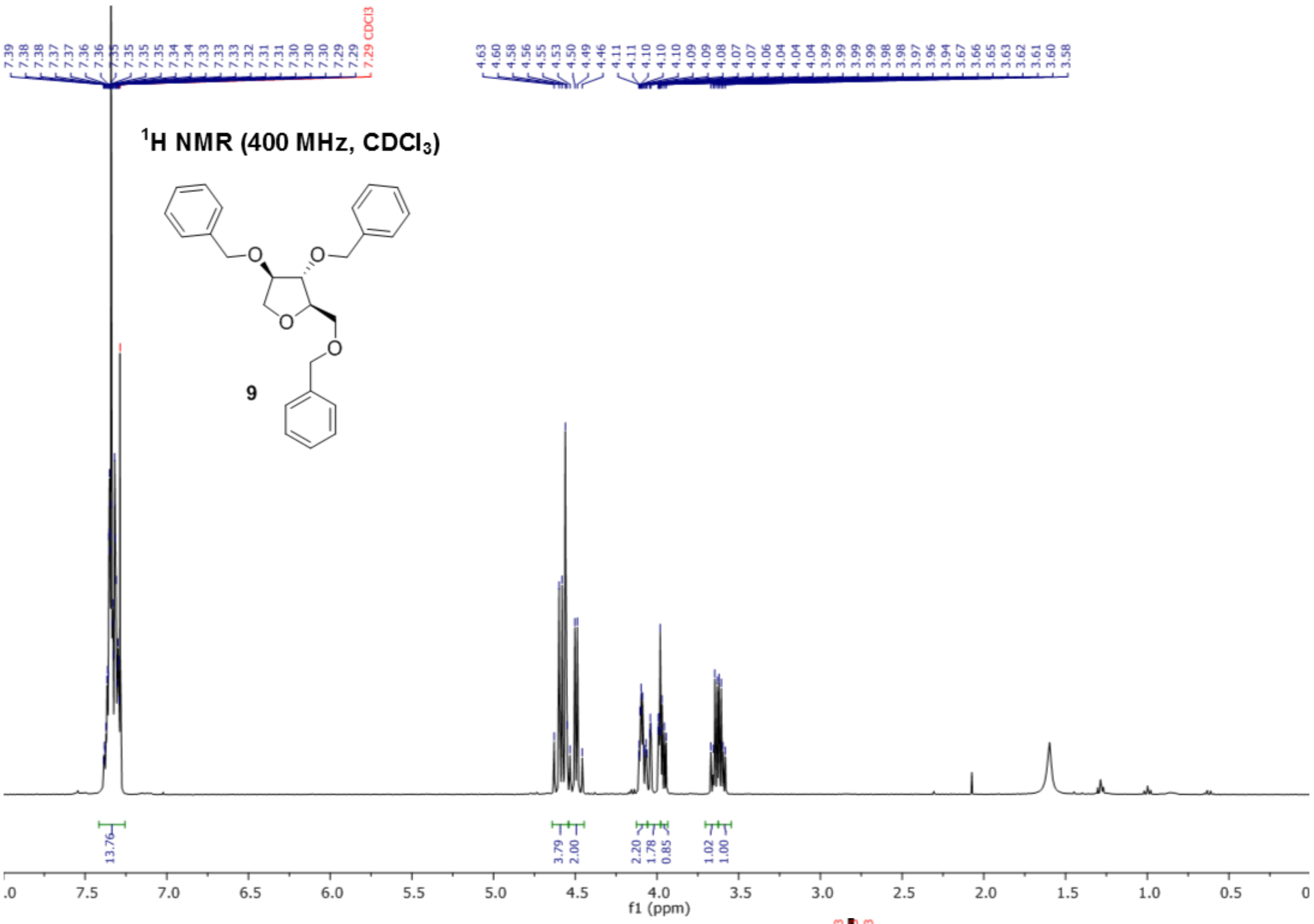
^{1}H NMR (400 MHz, CDCl$_3$)
9
f1 (ppm)

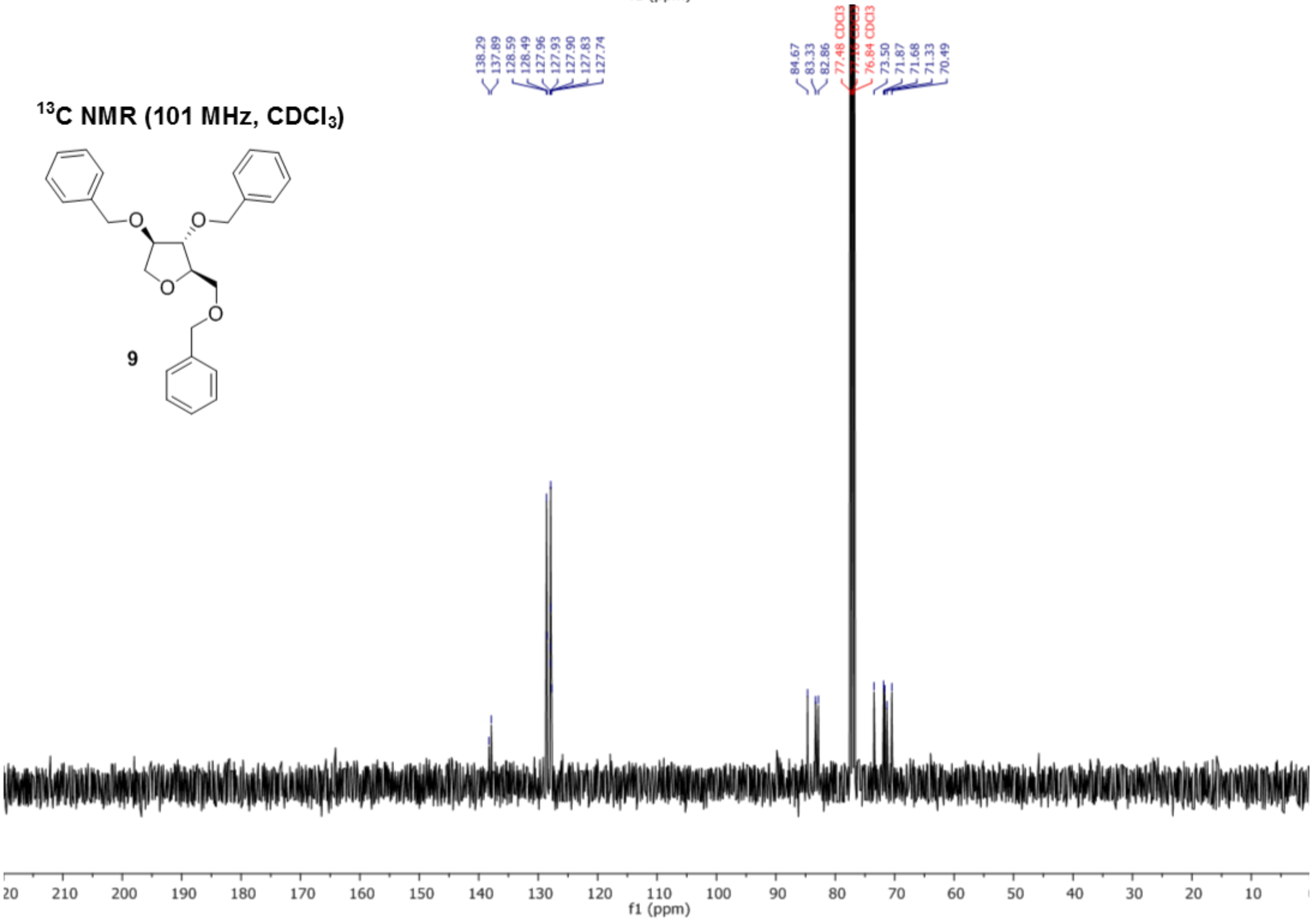
^{13}C NMR (101 MHz, CDCl$_3$)
9
f1 (ppm)

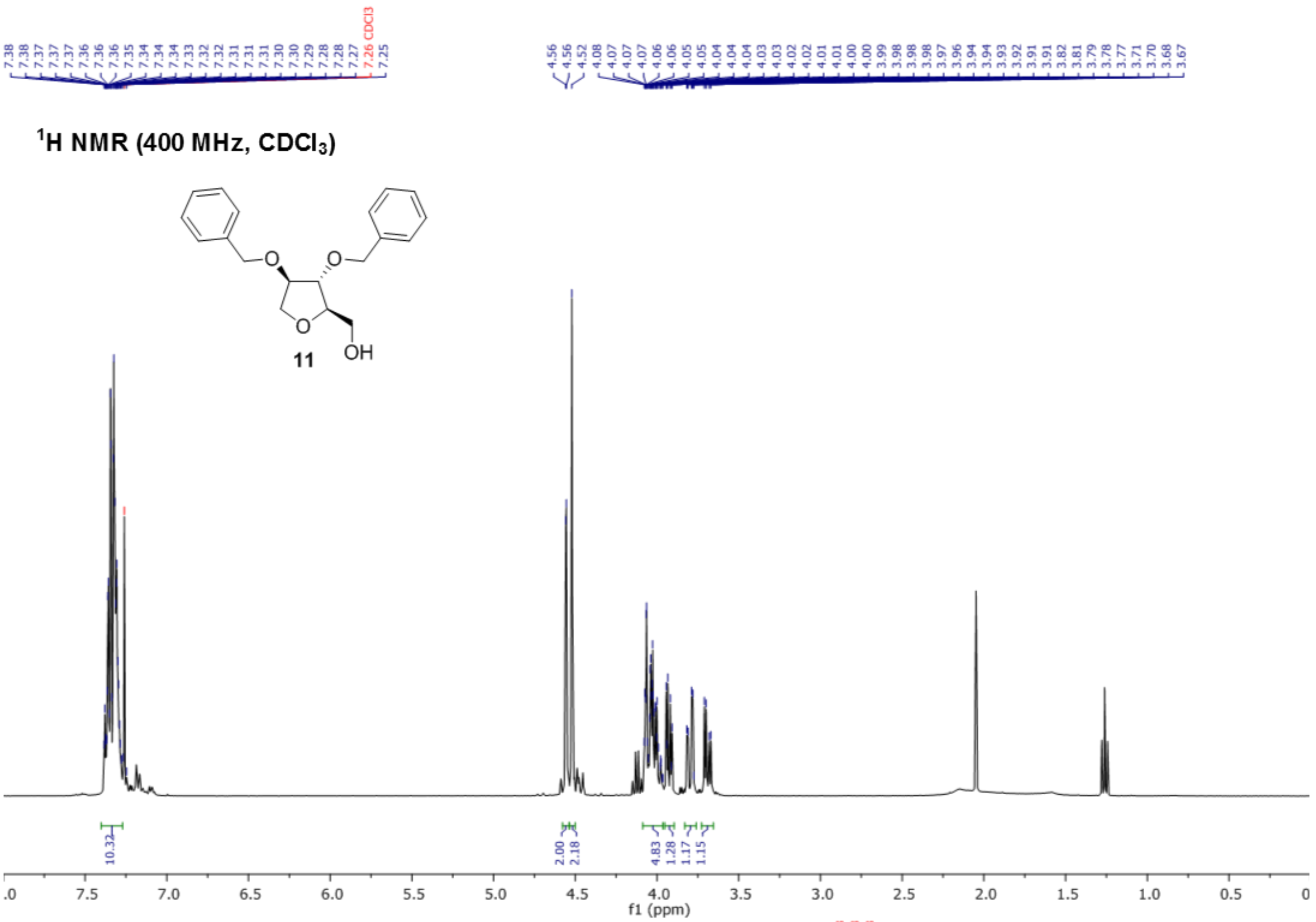
7.38 7.38 7.37 7.37 7.37 7.36 7.36 7.36 7.35 7.34 7.34 7.34 7.33 7.32 7.32 7.31 7.31 7.31 7.30 7.30 7.29 7.28 7.28 7.27 7.26 CDCl3 7.25
4.56 4.56 4.52 4.08 4.07 4.07 4.07 4.06 4.06 4.05 4.05 4.04 4.04 4.04 4.03 4.03 4.02 4.02 4.01 4.01 4.00 4.00 3.99 3.98 3.98 3.98 3.97 3.96 3.94 3.94 3.93 3.92 3.91 3.91 3.82 3.81 3.79 3.78 3.77 3.71 3.70 3.68 3.67
1H NMR (400 MHz, CDCl3)
O
O
O
11
OH
10.32
2.00
2.18
4.83
1.28
1.17
1.15
f1 (ppm)

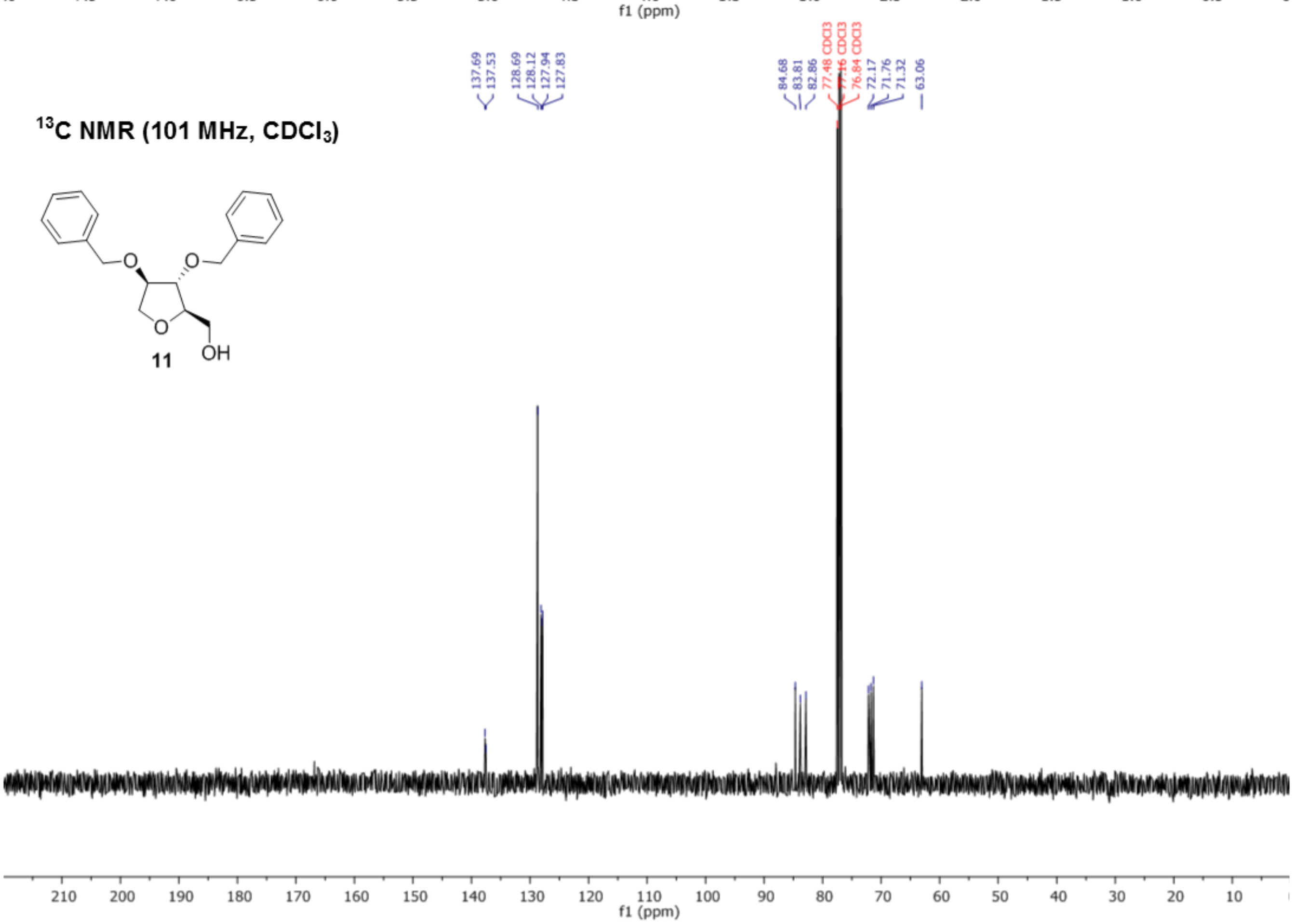
137.69 137.53
128.69 128.12 127.94 127.83
84.68 83.81 82.86
77.48 CDCl3 77.16 CDCl3 76.84 CDCl3
72.17 71.76 71.32
63.06
13C NMR (101 MHz, CDCl3)
O
O
O
11
OH
f1 (ppm)

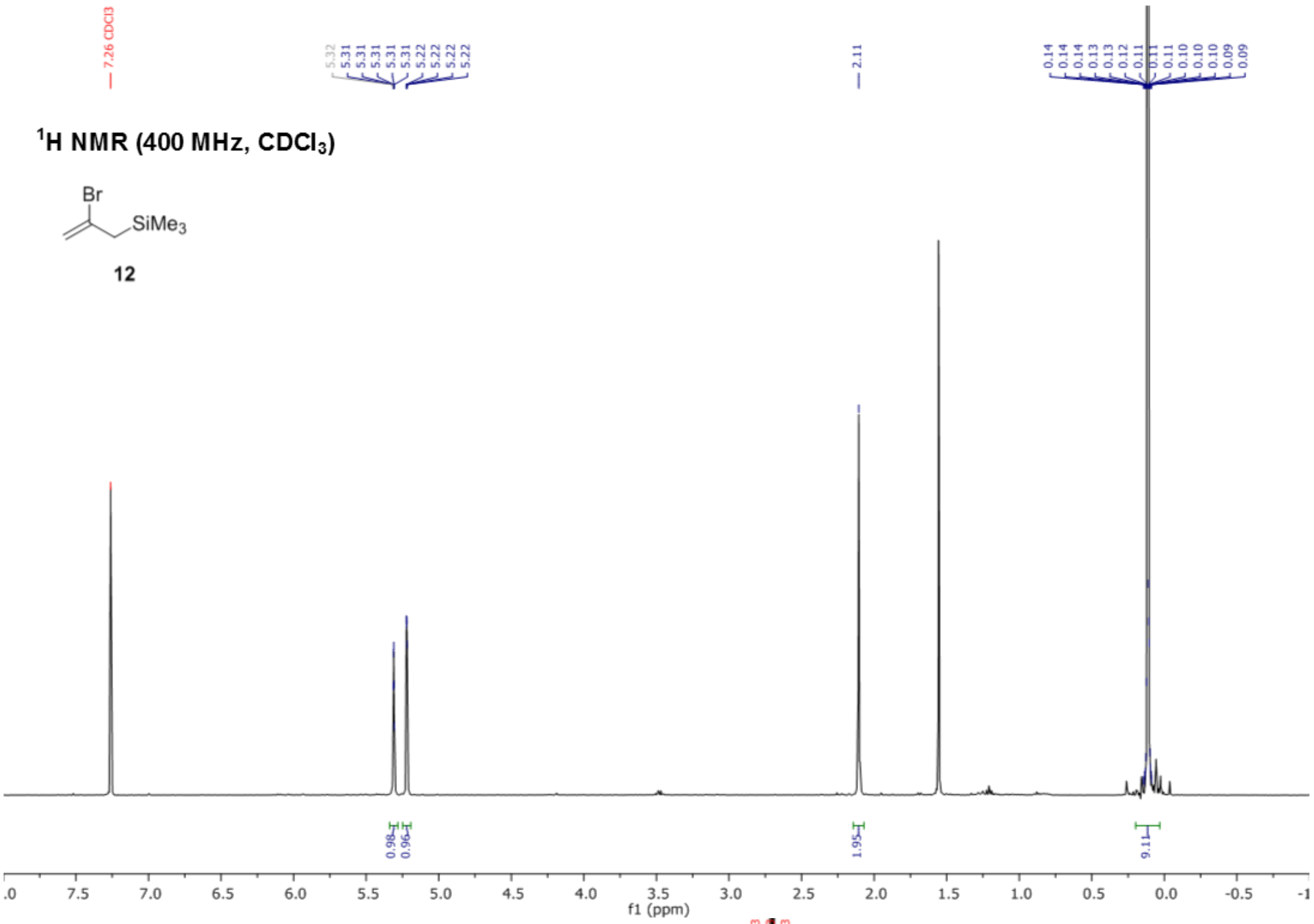

7.26 CDCl3
5.32
5.31
5.31
5.31
5.31
5.31
5.22
5.22
5.22
5.22
2.11
0.14
0.14
0.14
0.13
0.13
0.12
0.11
0.11
0.11
0.10
0.10
0.09
0.09
^{1}H NMR (400 MHz, $CDCl_3$)
Br
$SiMe_3$
12
0.98
0.96
1.95
9.11
f1 (ppm)

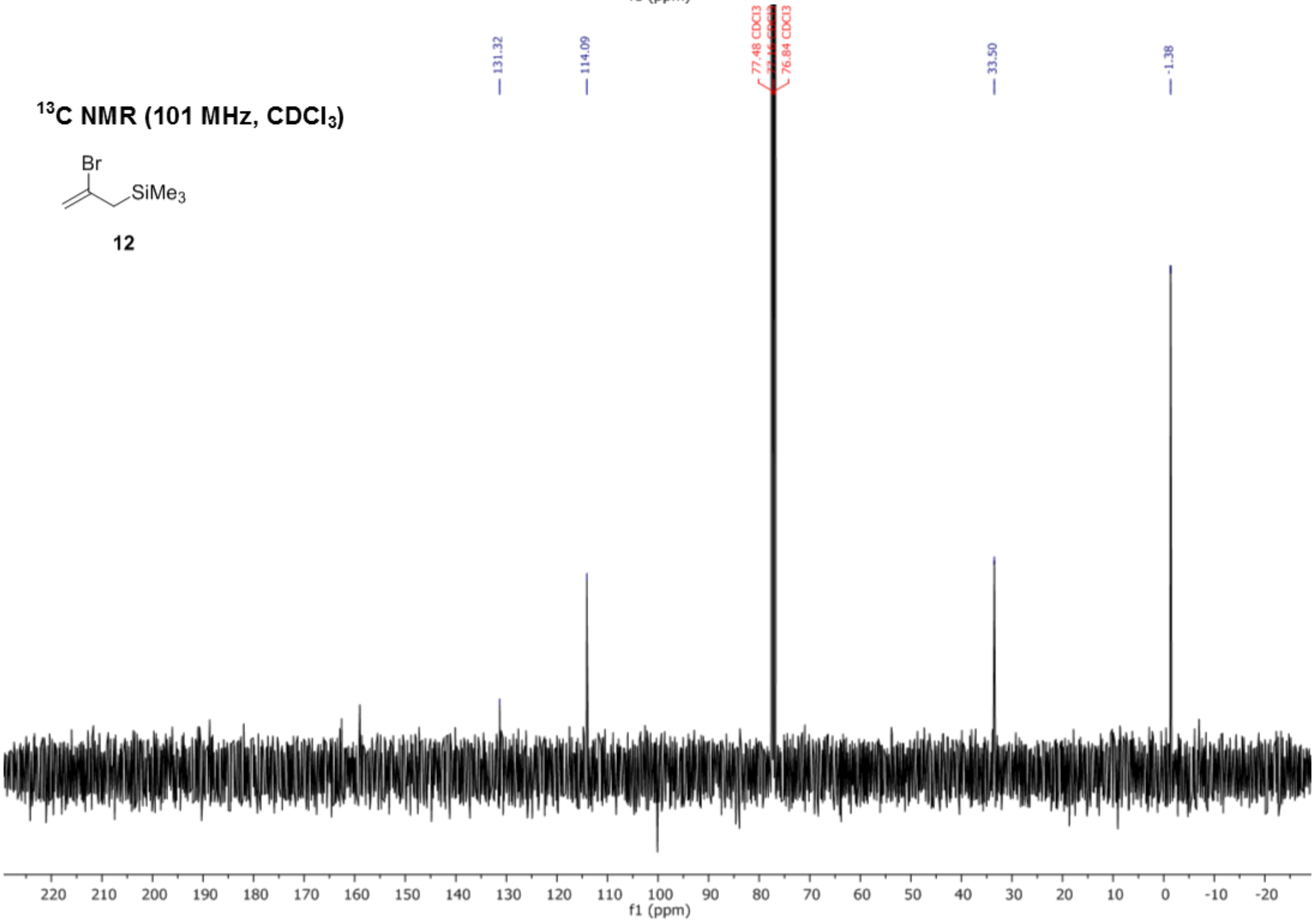

131.32
114.09
77.48 CDCl3
76.84 CDCl3
33.50
-1.38
^{13}C NMR (101 MHz, $CDCl_3$)
Br
$SiMe_3$
12
f1 (ppm)

^{1}H NMR (400 MHz, $CDCl_3$)

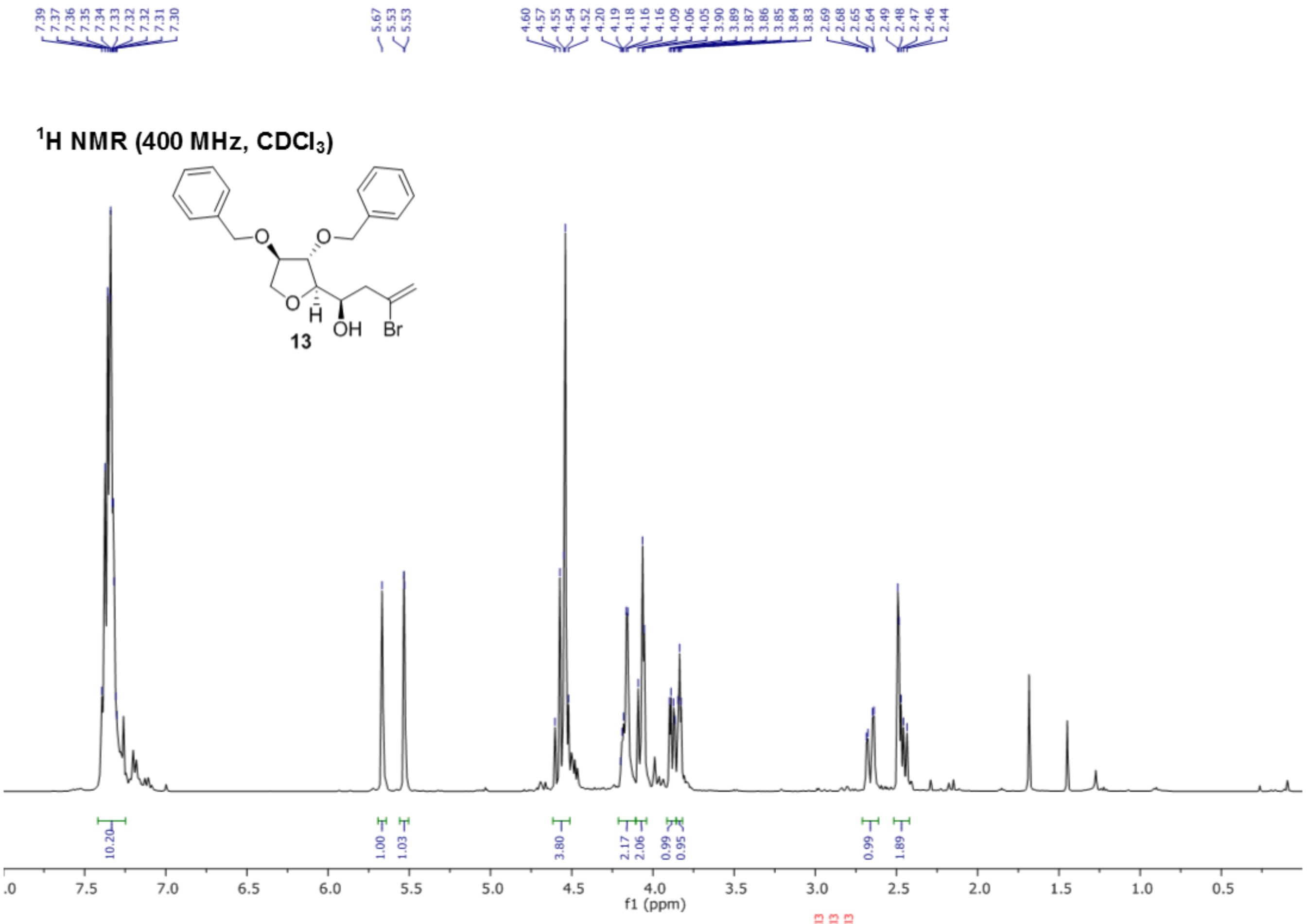

^{13}C NMR (101 MHz, $CDCl_3$)

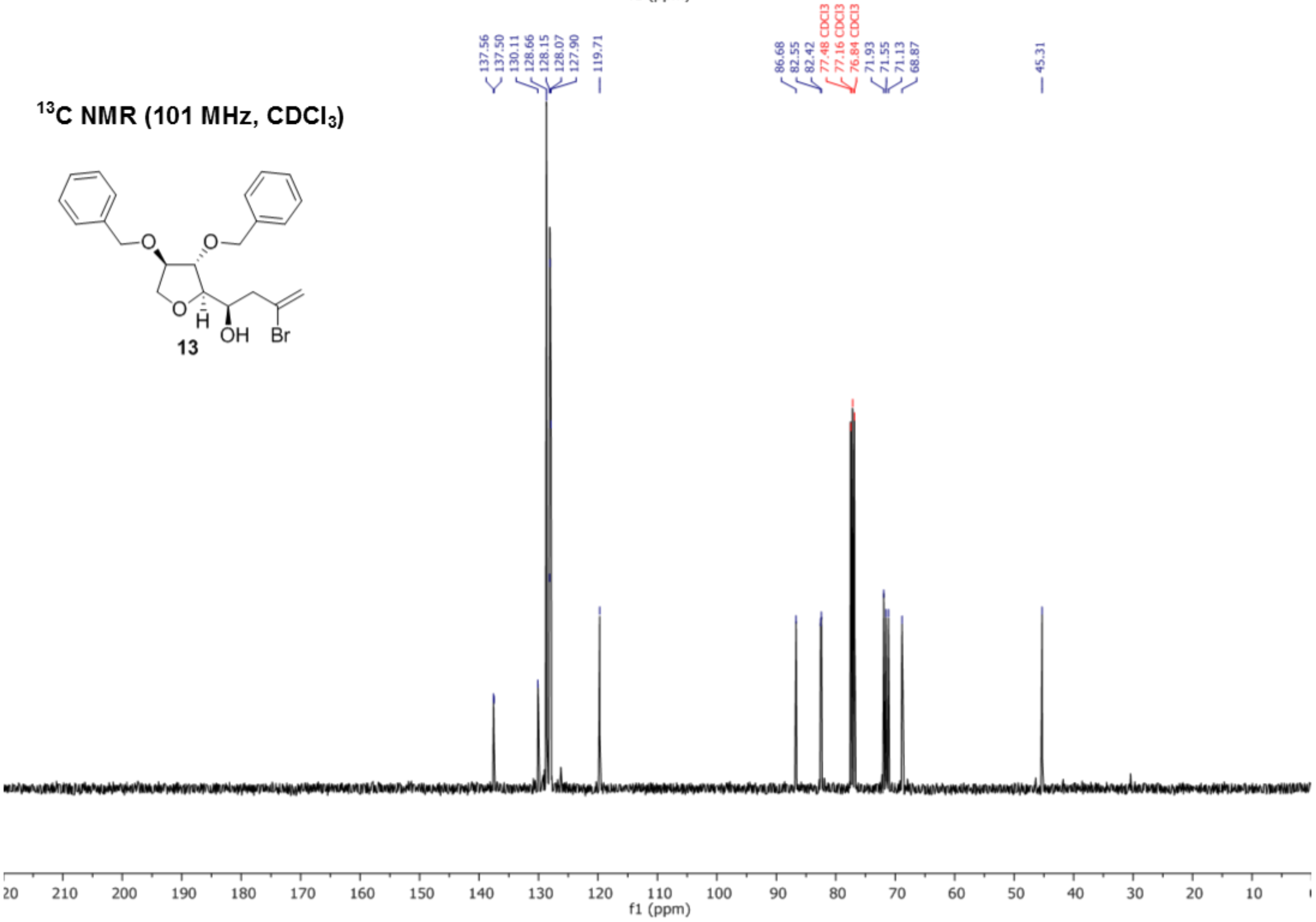

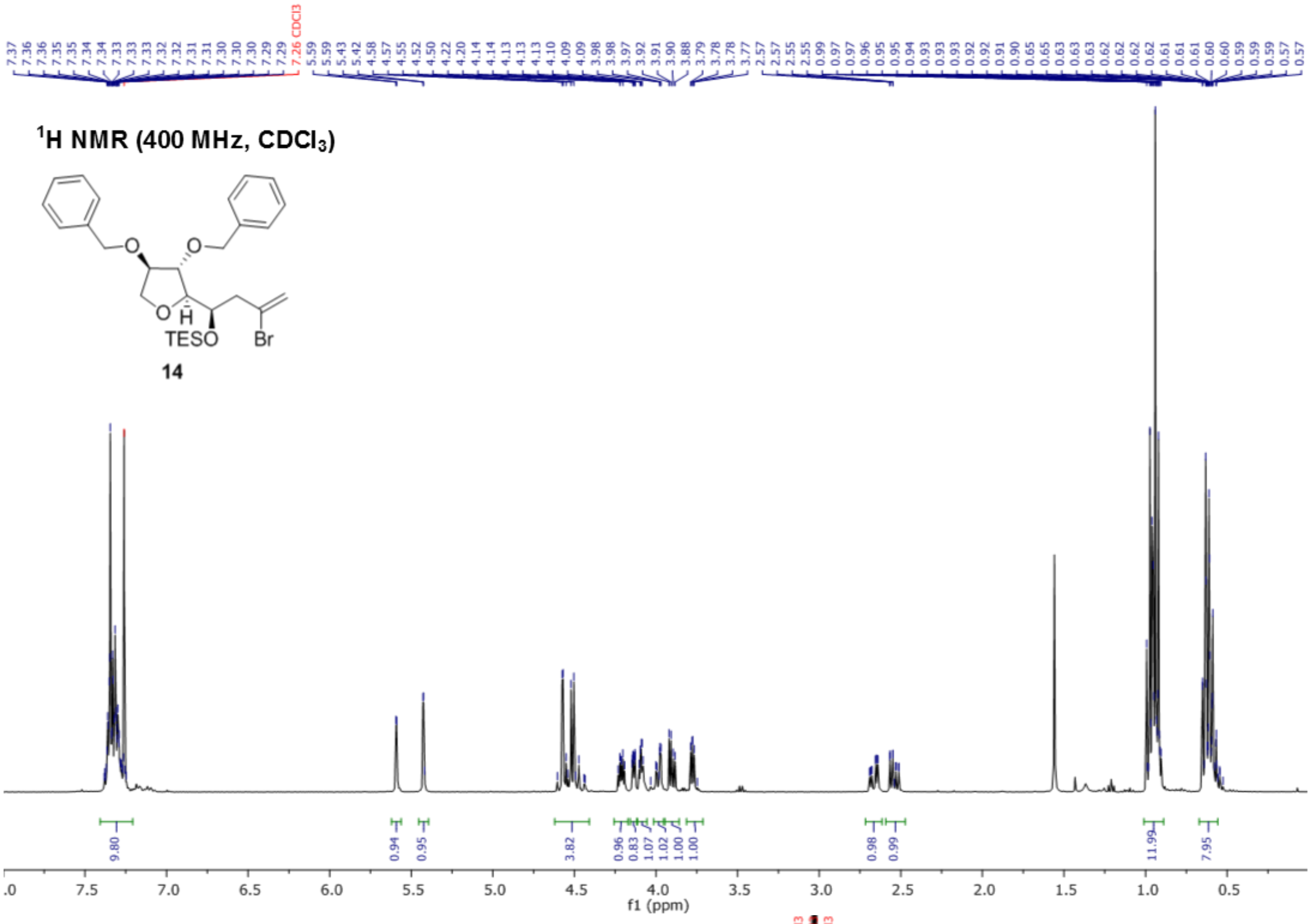

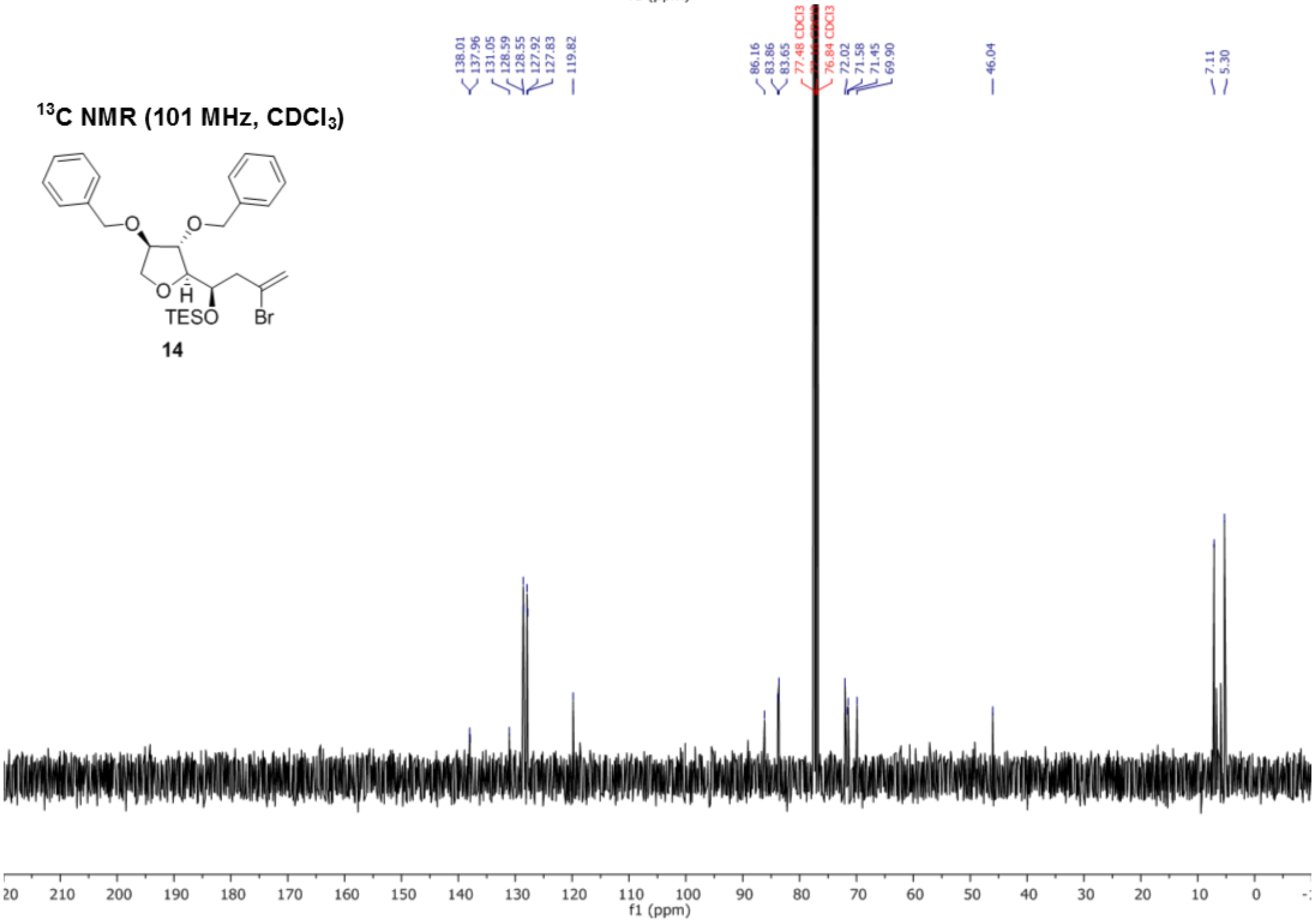

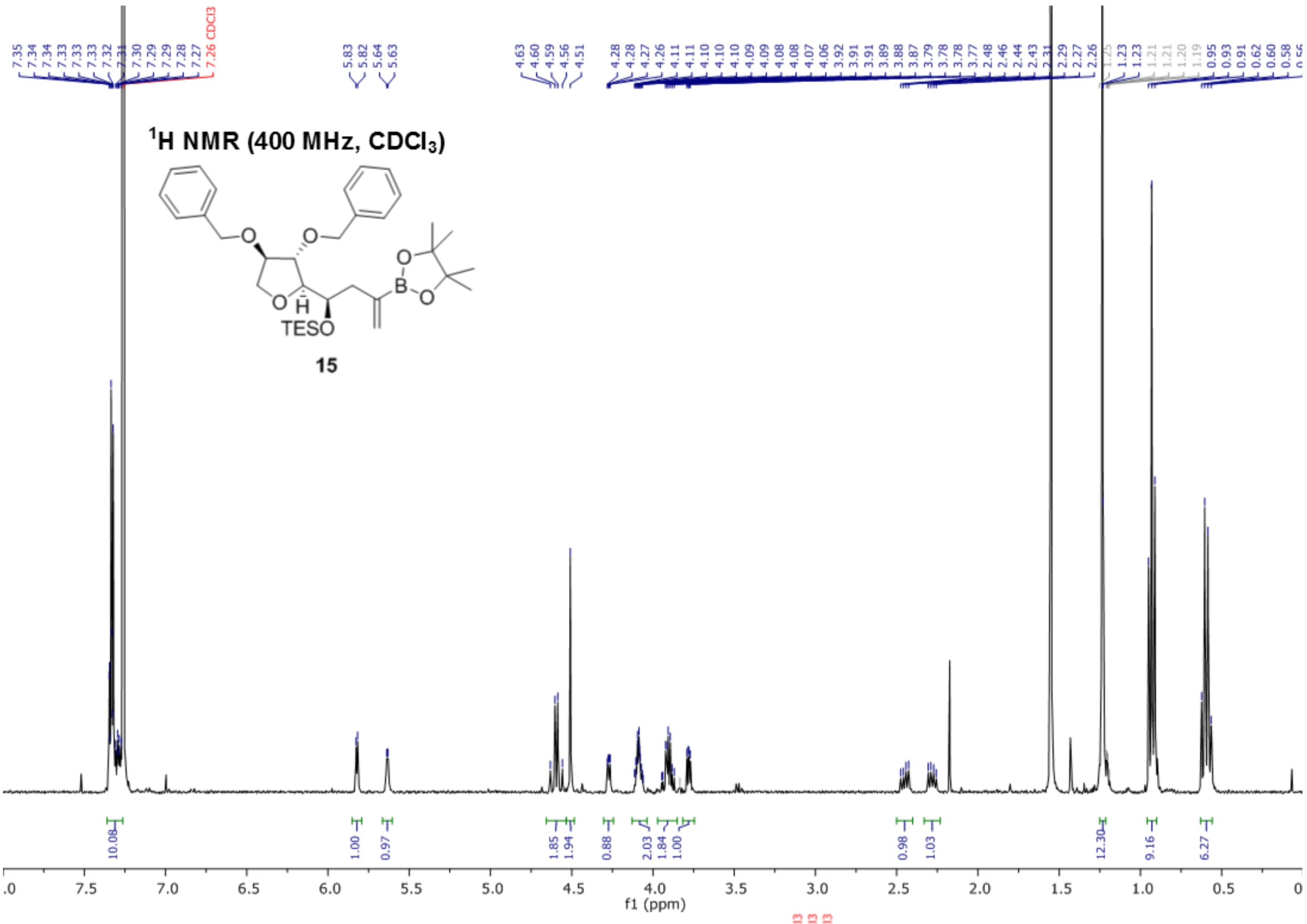
1H NMR (400 MHz, CDCl3)
15
f1 (ppm)

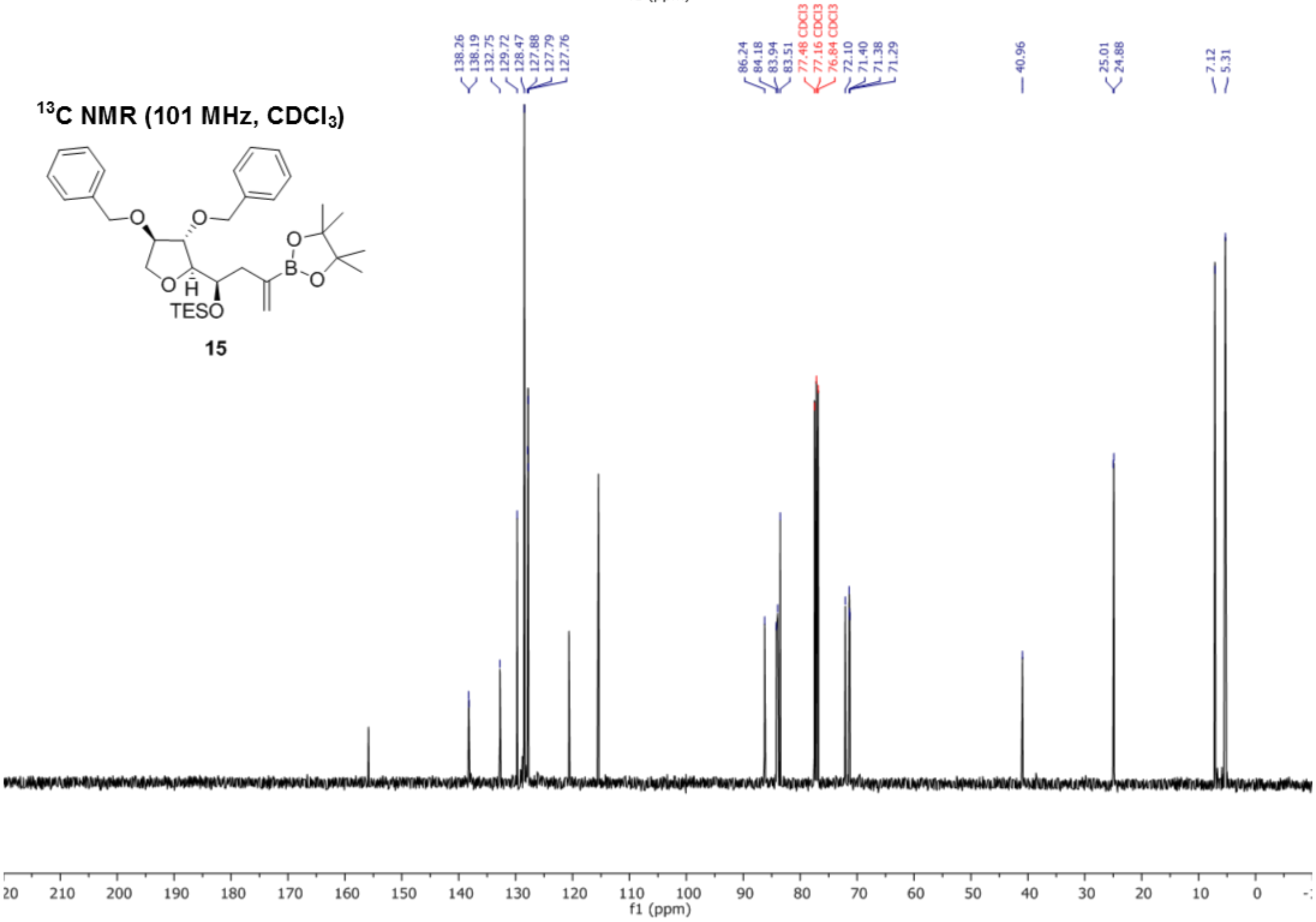
13C NMR (101 MHz, CDCl3)
15
f1 (ppm)

^1H NMR (400 MHz, CD_2Cl_2)

18

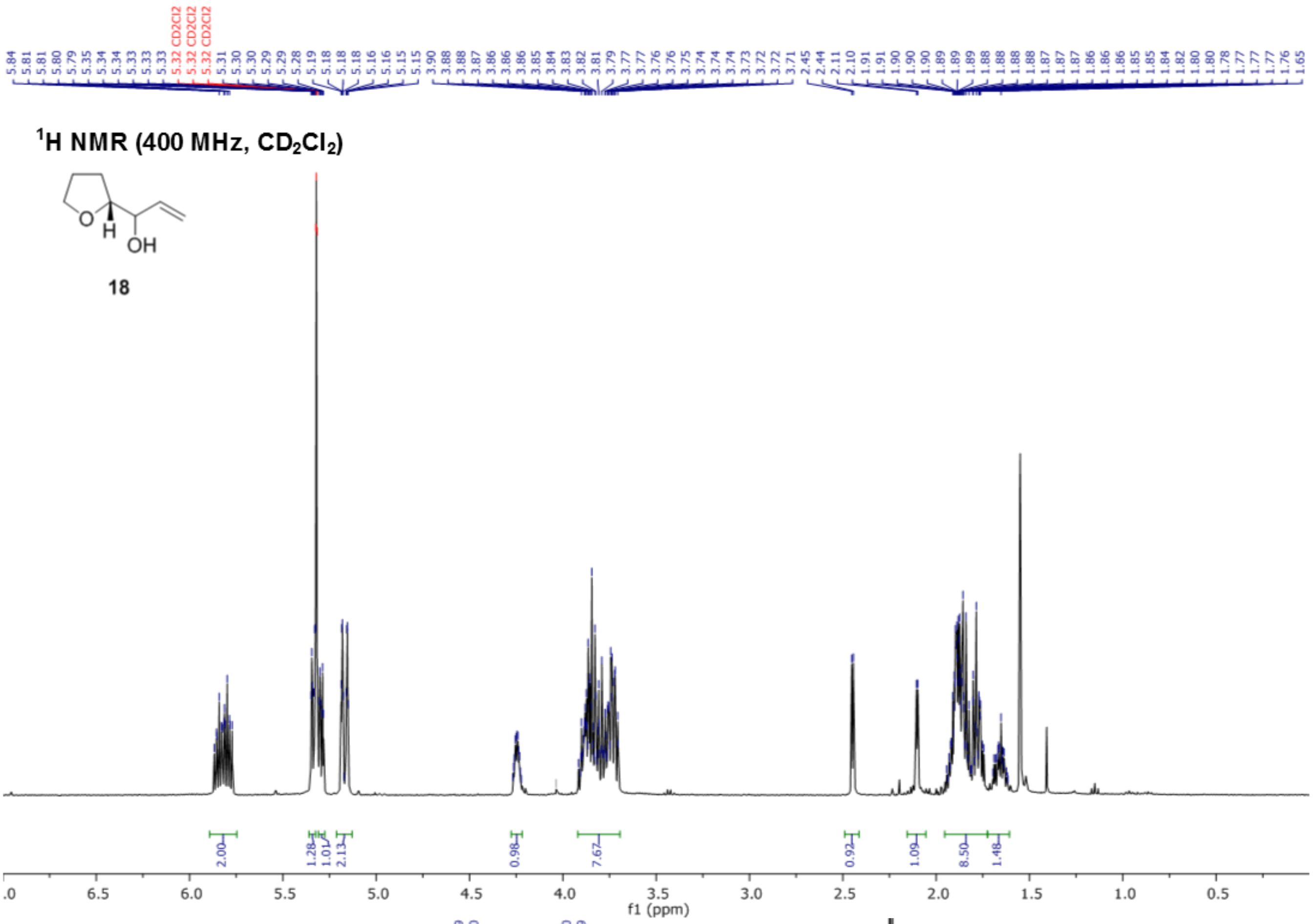

^{13}C NMR (101 MHz, CD_2Cl_2)

18

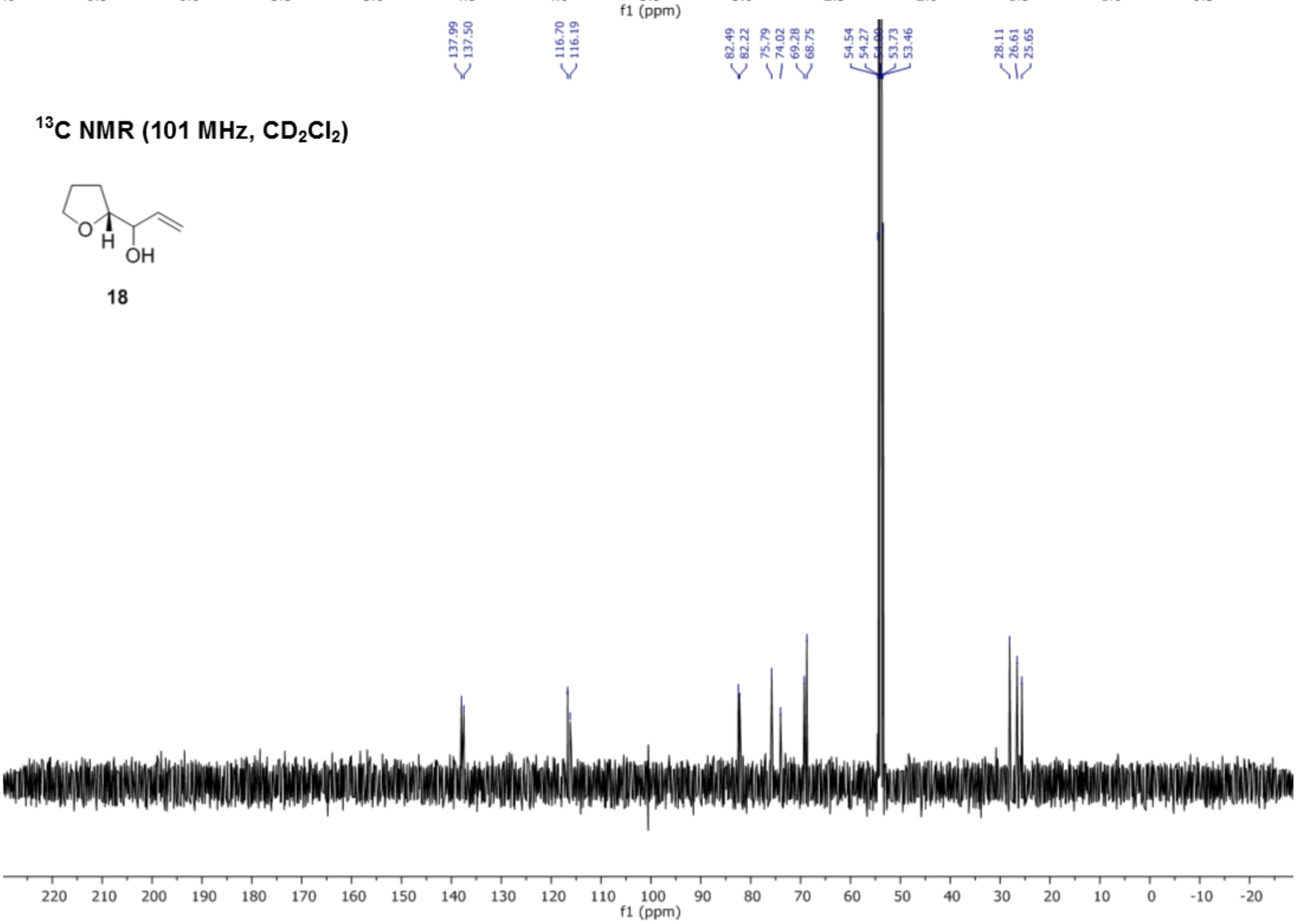

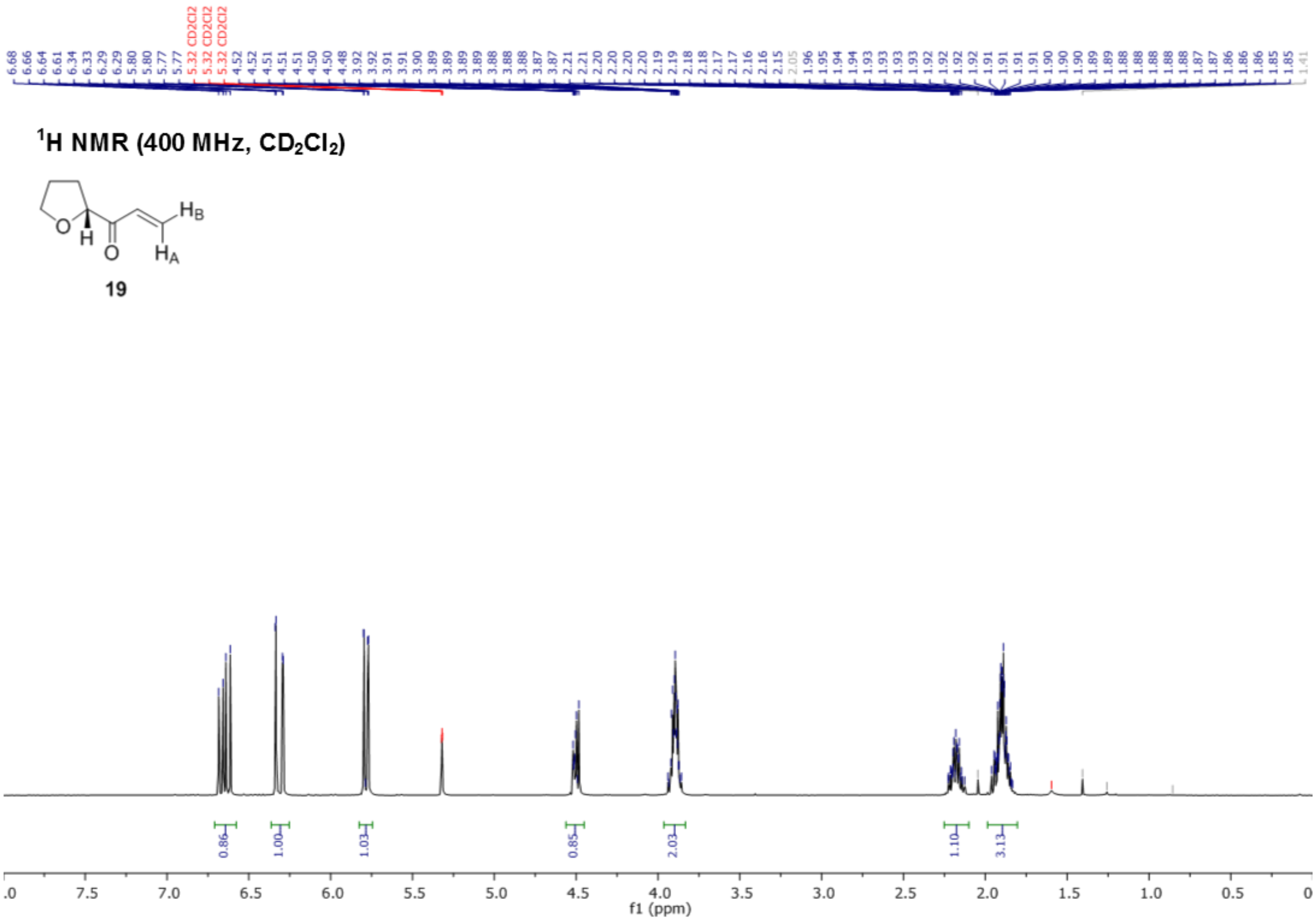

^{1}H NMR (400 MHz, CD_2Cl_2)

19

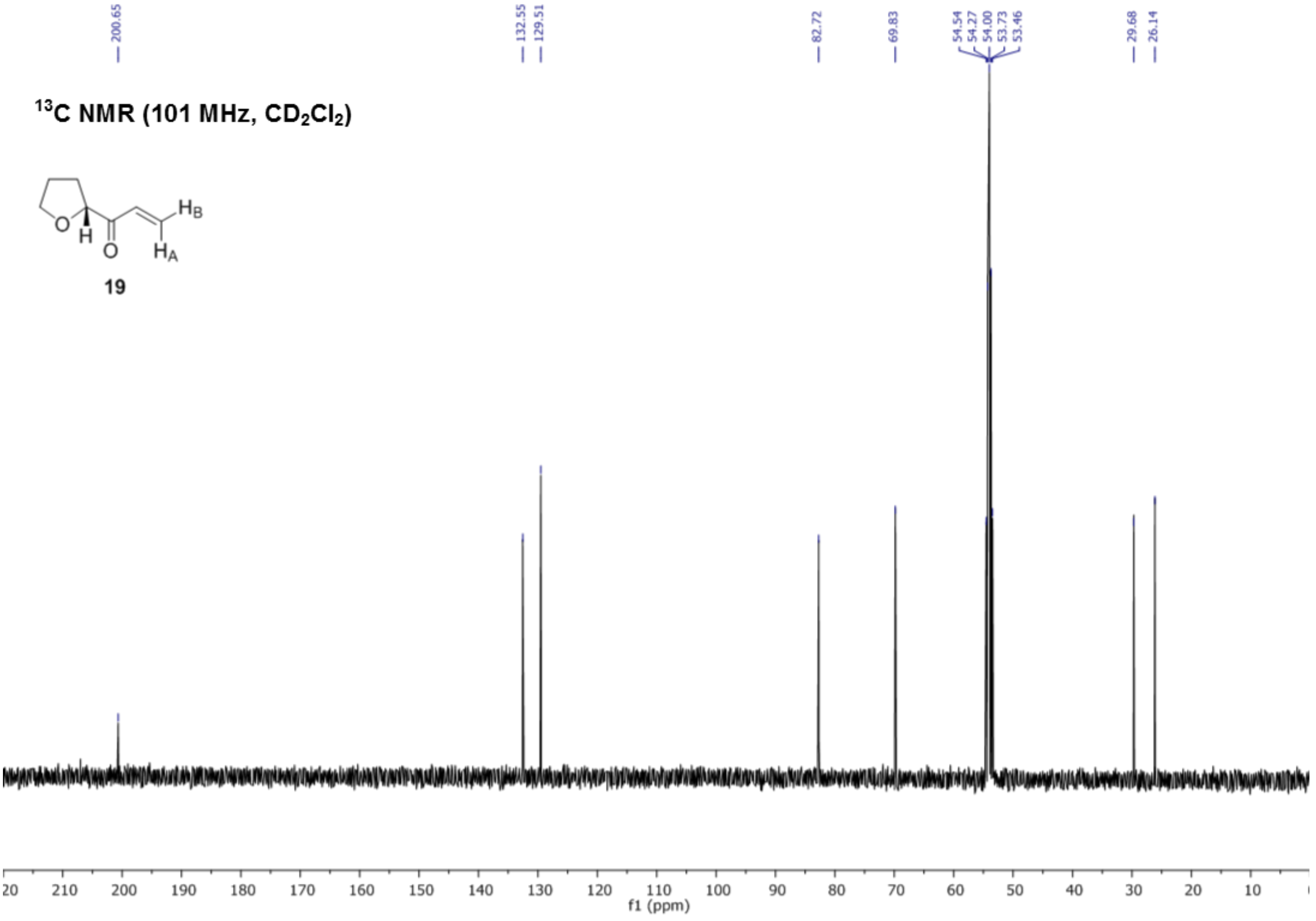

^{13}C NMR (101 MHz, CD_2Cl_2)

19

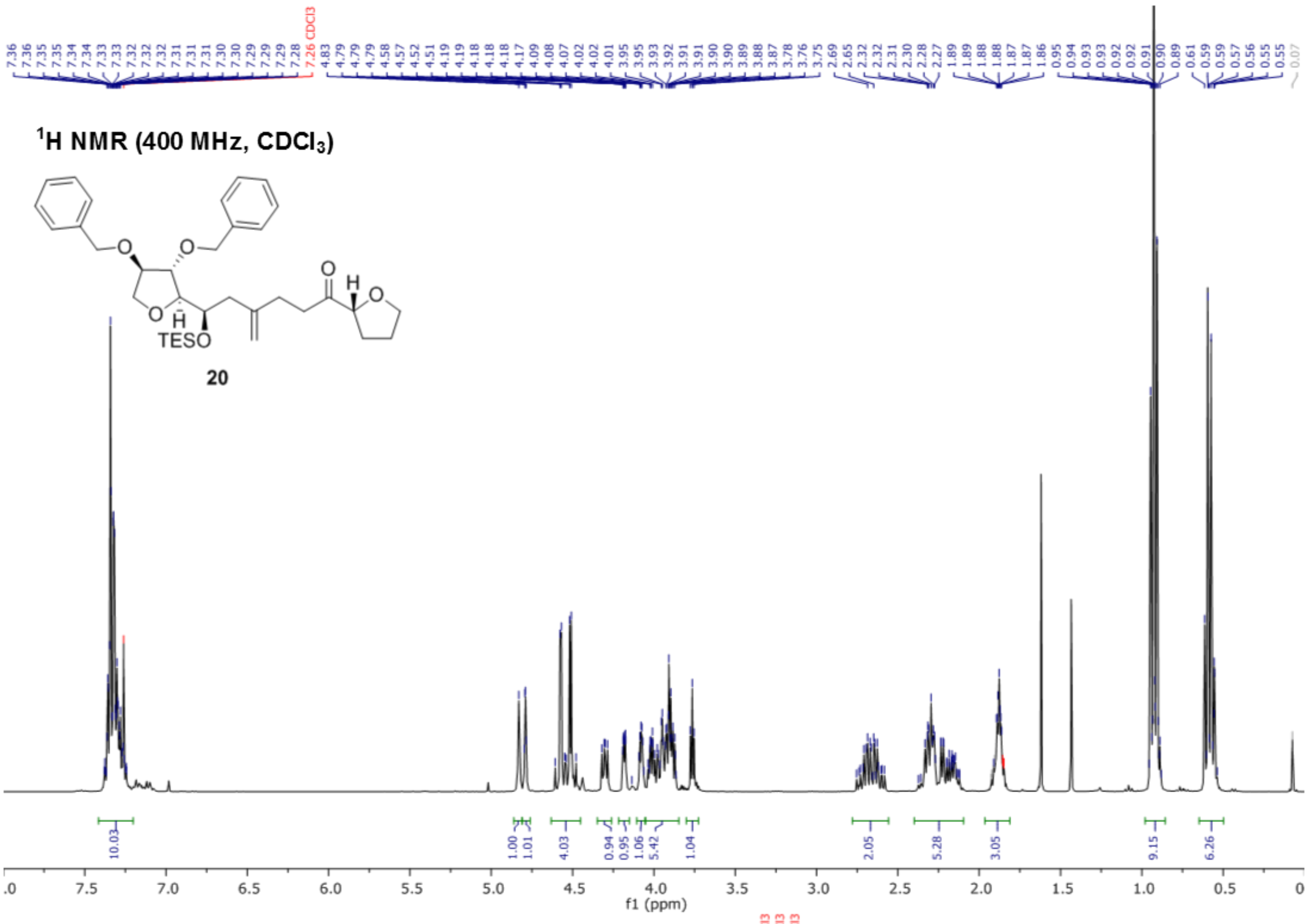
^{1}H NMR (400 MHz, CDCl$_3$)
20
f1 (ppm)

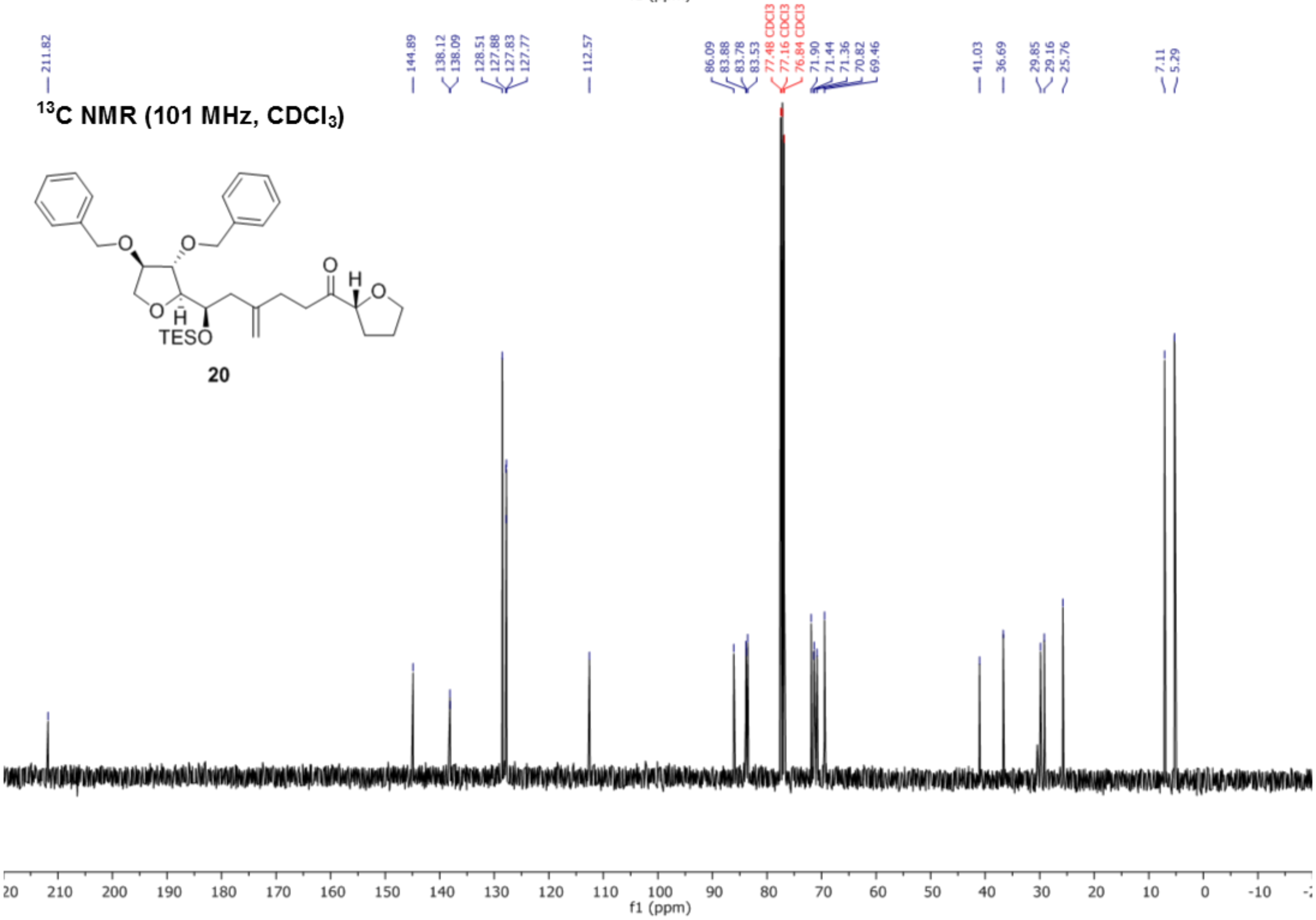
^{13}C NMR (101 MHz, CDCl$_3$)
20
f1 (ppm)

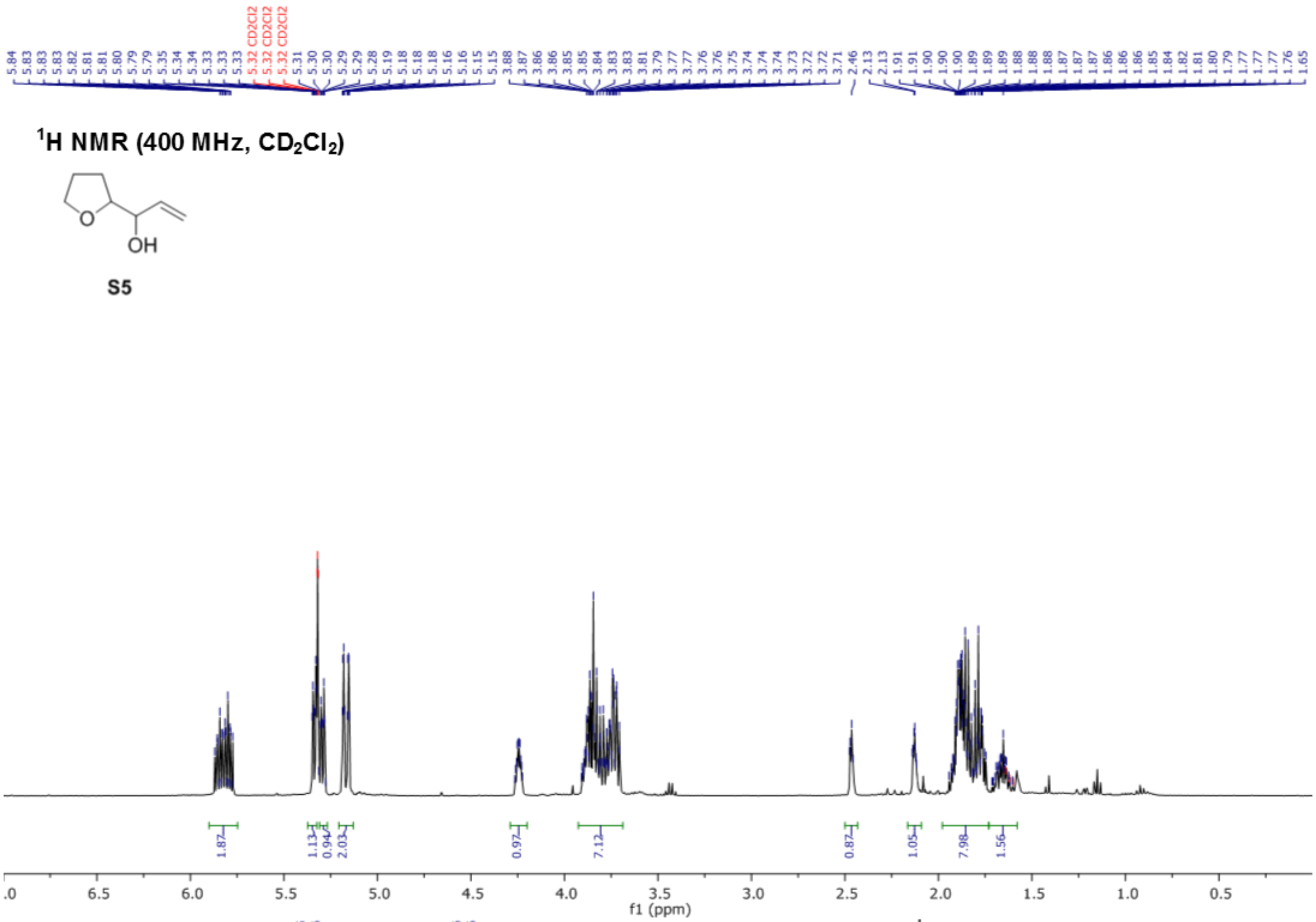
1H NMR (400 MHz, CD2Cl2)
O
OH
S5

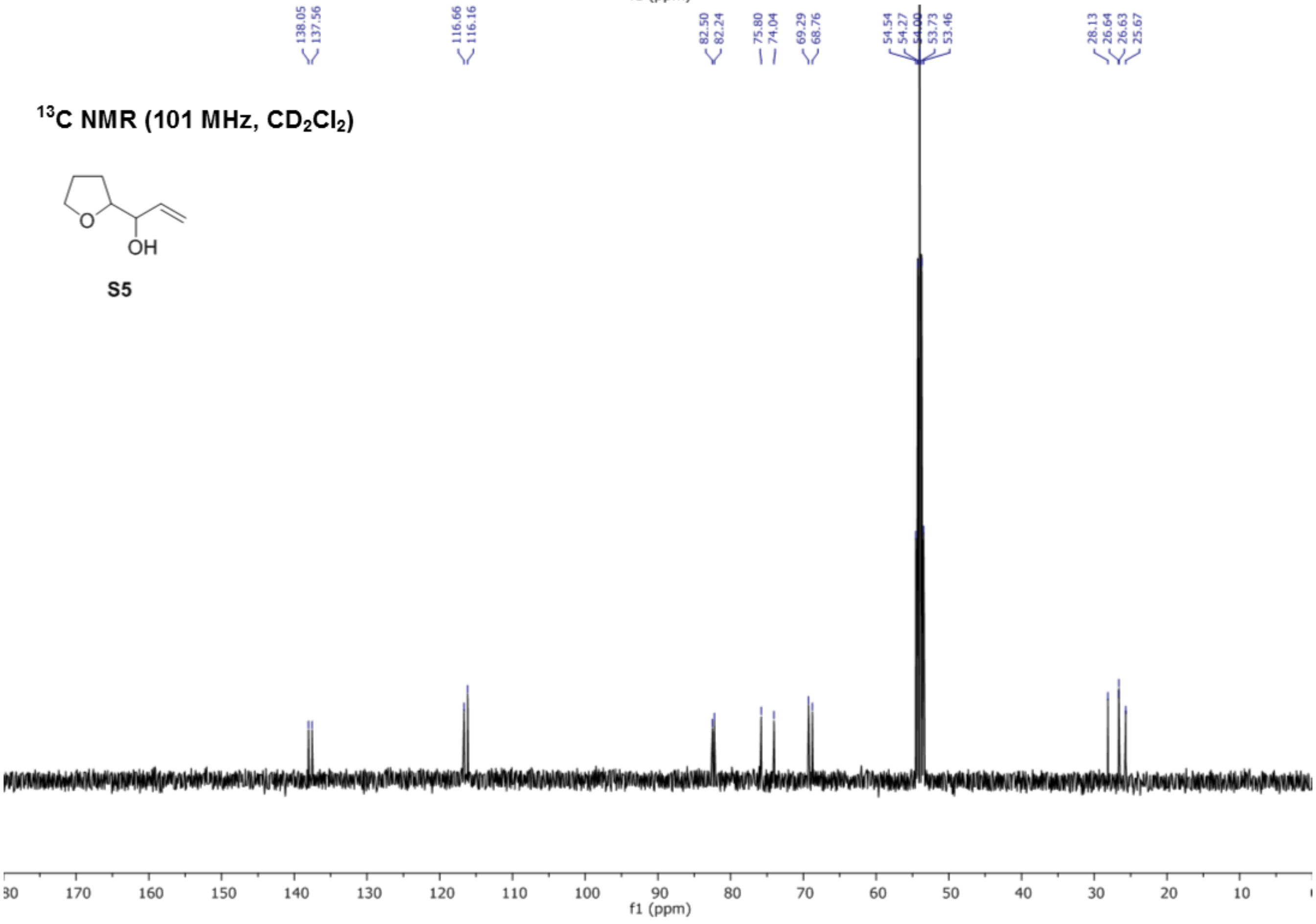
13C NMR (101 MHz, CD2Cl2)
O
OH
S5
138.05
137.56
116.66
116.16
82.50
82.24
75.80
74.04
69.29
68.76
54.54
54.27
54.00
53.73
53.46
28.13
26.64
26.63
25.67
f1 (ppm)

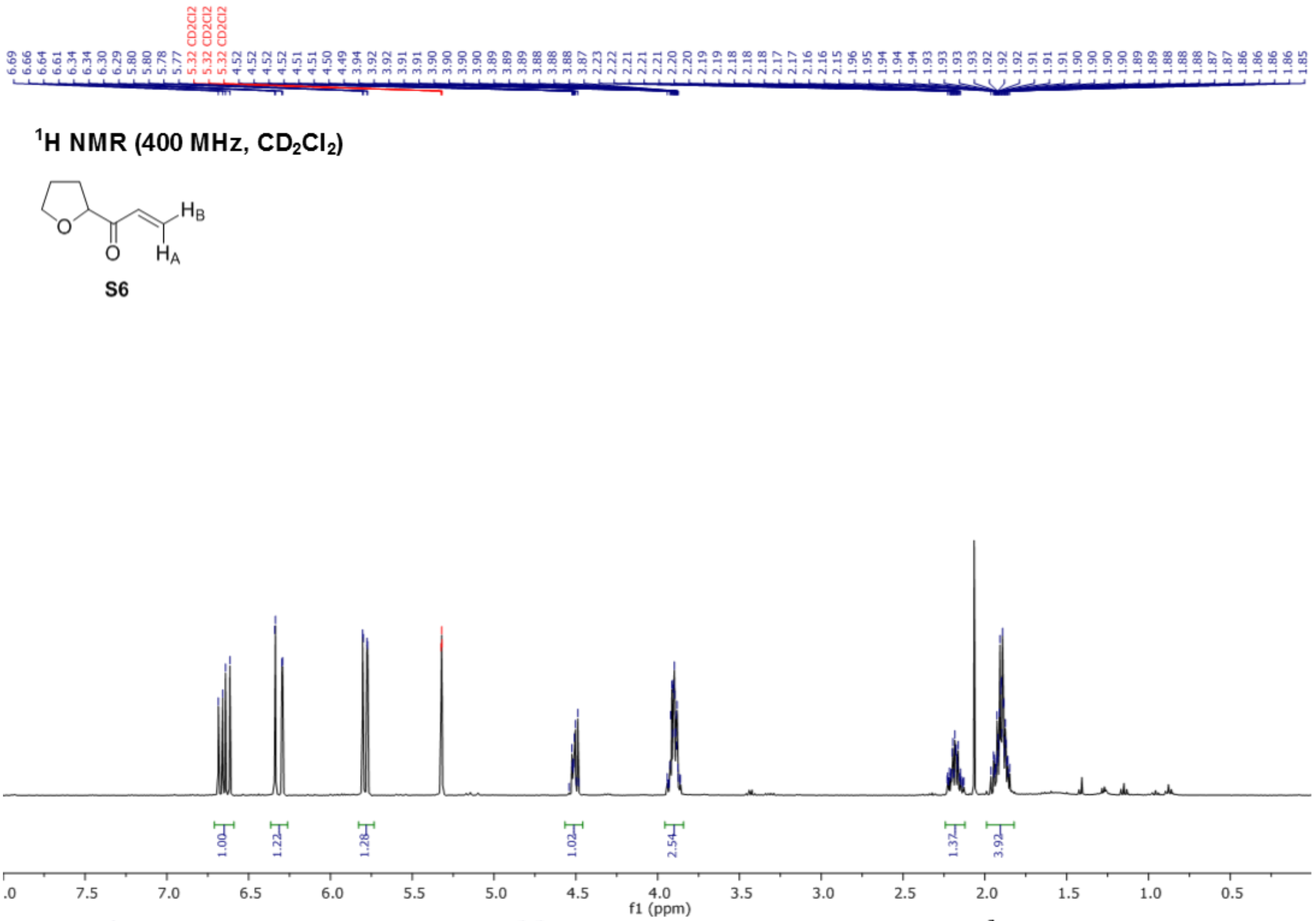
¹H NMR (400 MHz, CD₂Cl₂)
H_B
H_A
O
S6
1.00
1.22
1.28
1.02
2.54
1.37
3.92
f1 (ppm)

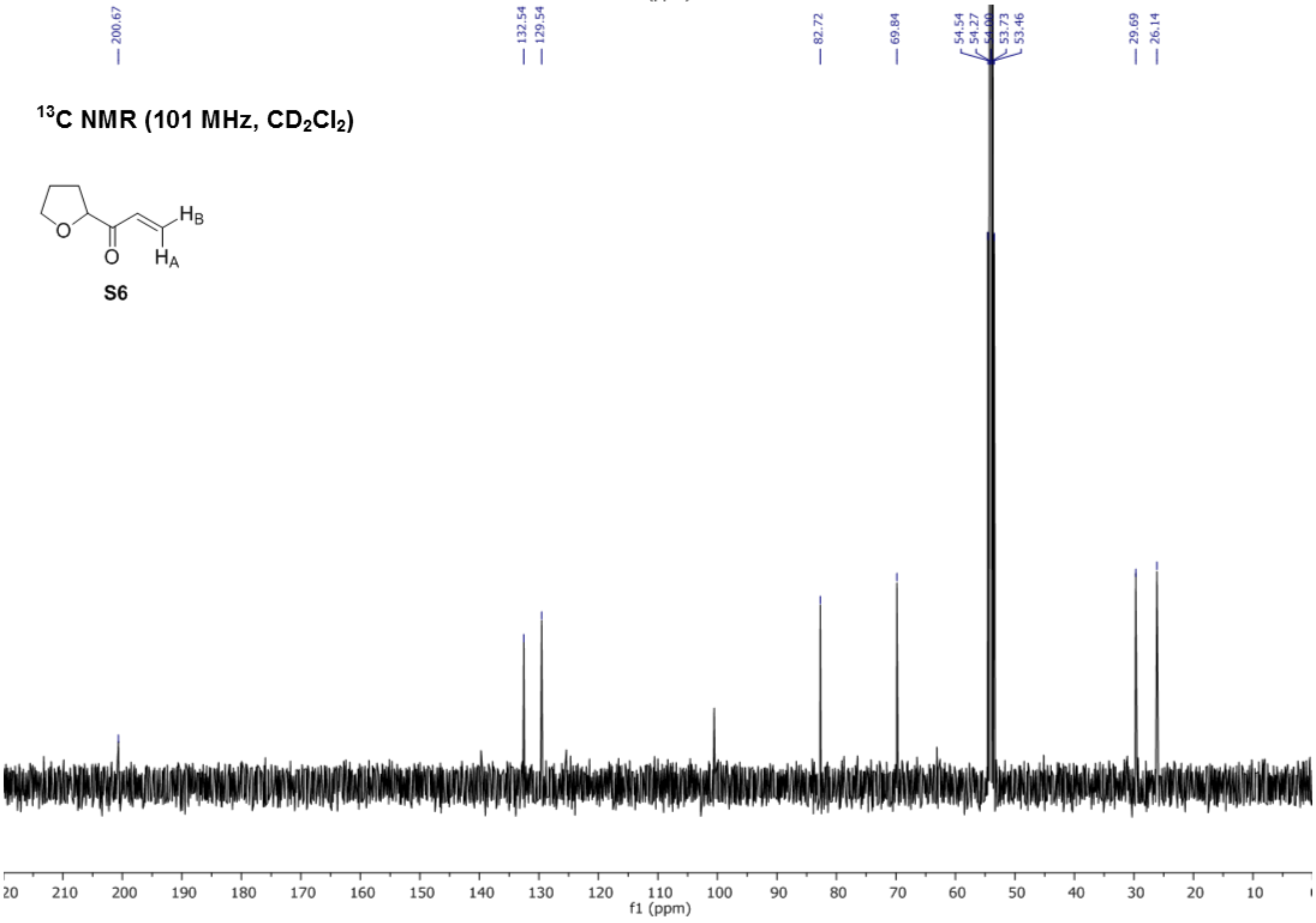
200.67
132.54
129.54
82.72
69.84
54.54
54.27
53.73
53.46
29.69
26.14
¹³C NMR (101 MHz, CD₂Cl₂)
H_B
H_A
O
S6
f1 (ppm)

^{1}H NMR (500 MHz, $CDCl_3$)

S7:S8

1:0.18

S7

S8

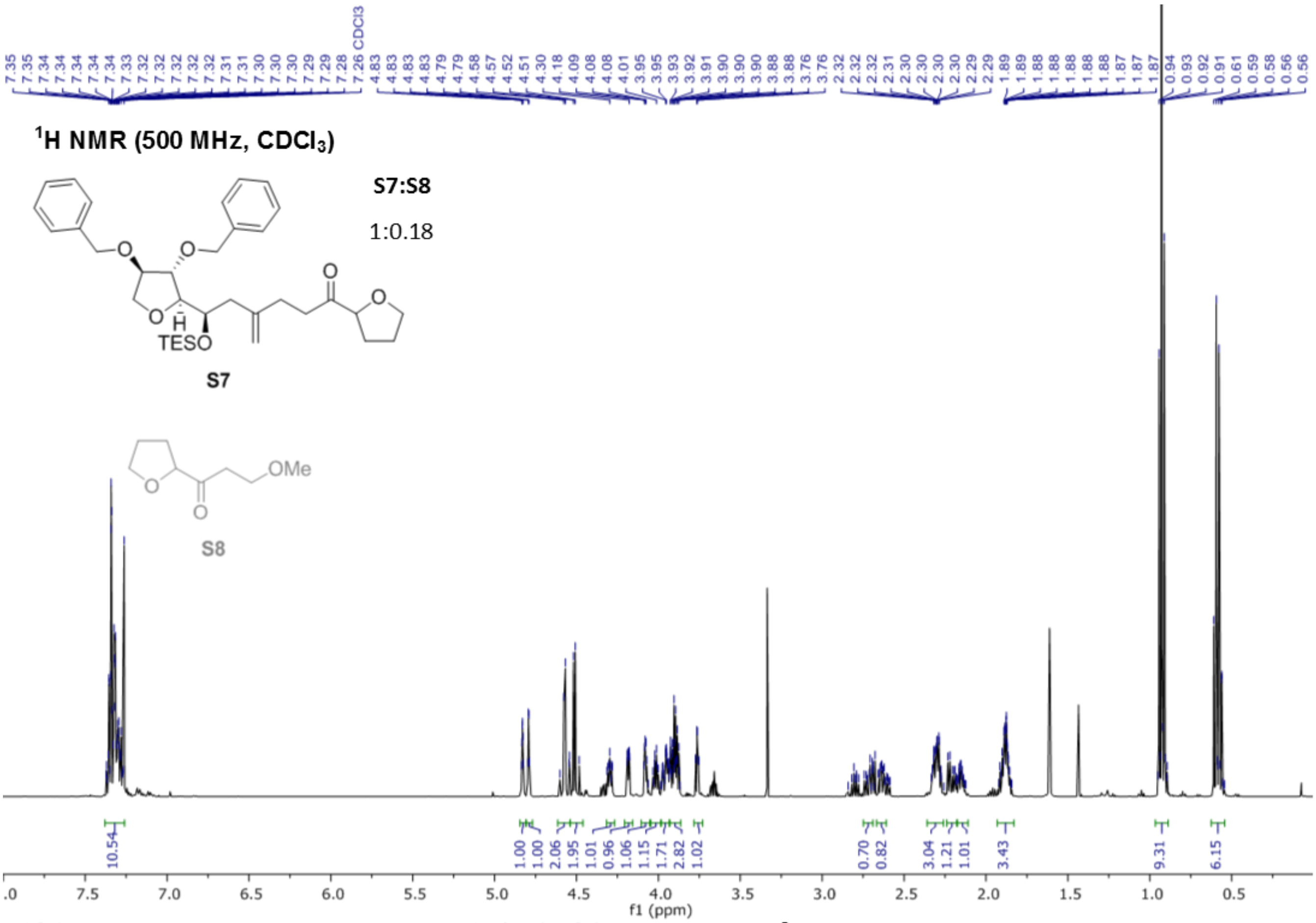

^{13}C NMR (126 MHz, $CDCl_3$)

S7:S8

1:0.18

S7

S8

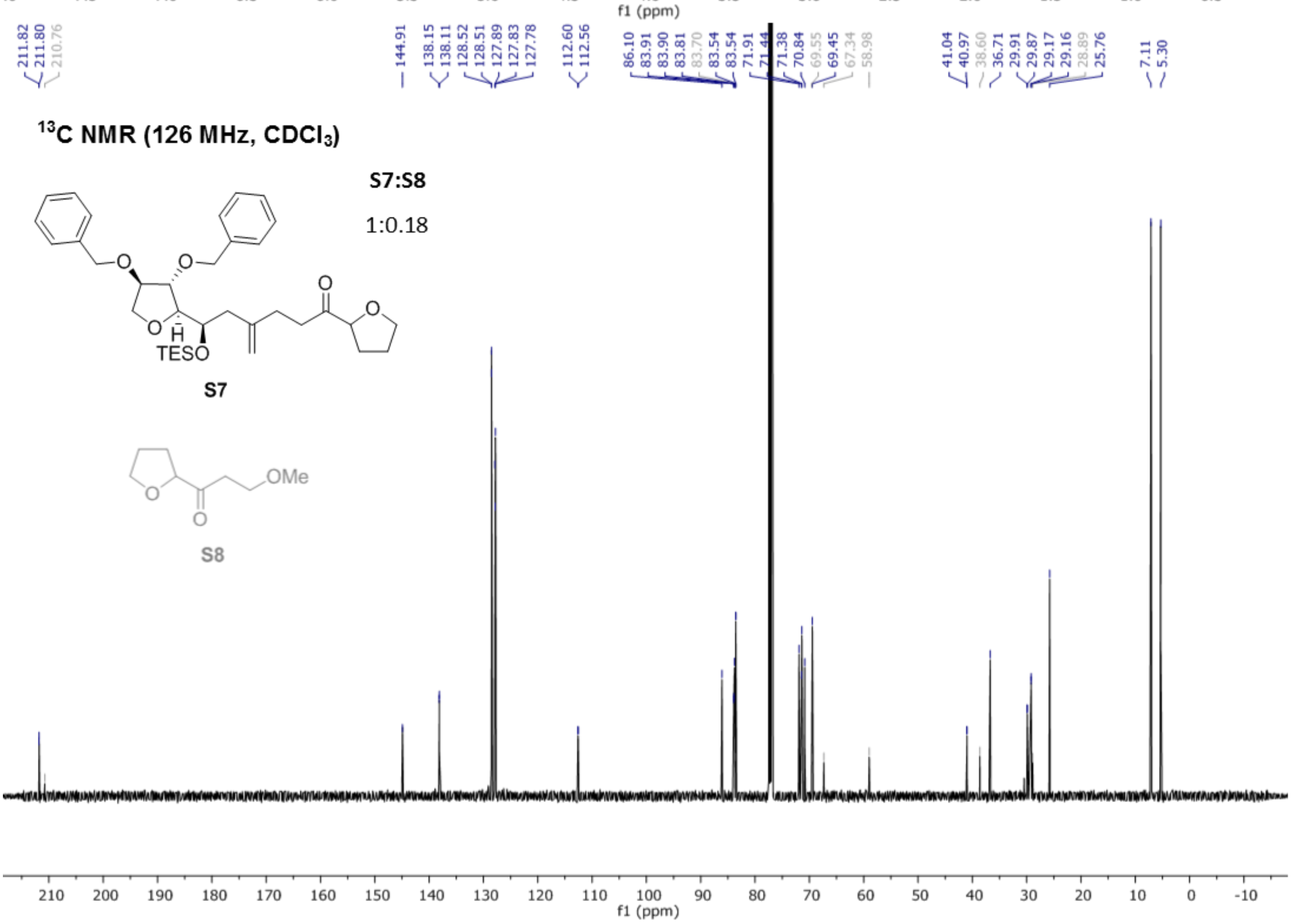

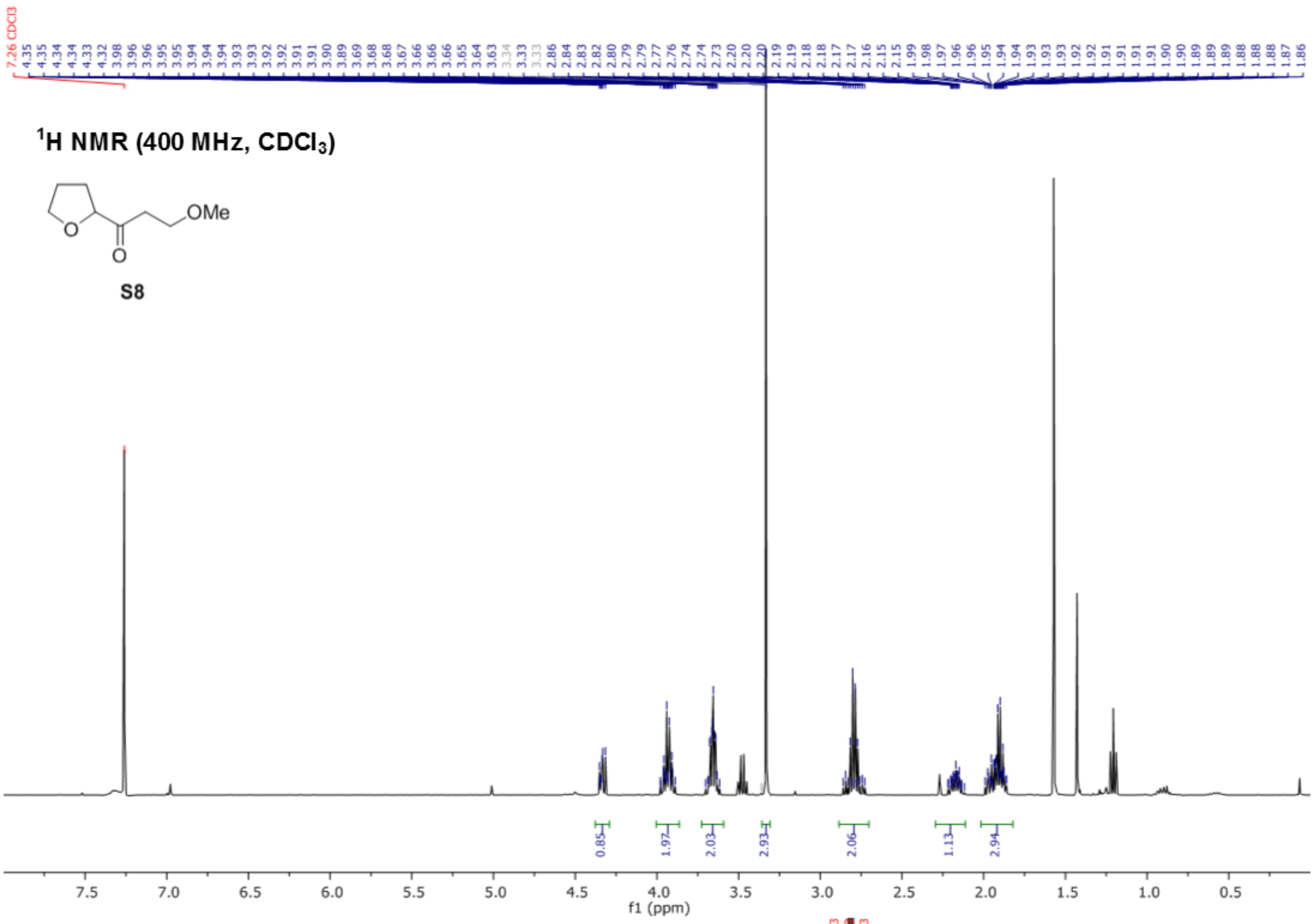

^{1}H NMR (400 MHz, $CDCl_3$)
OMe
S8
f1 (ppm)

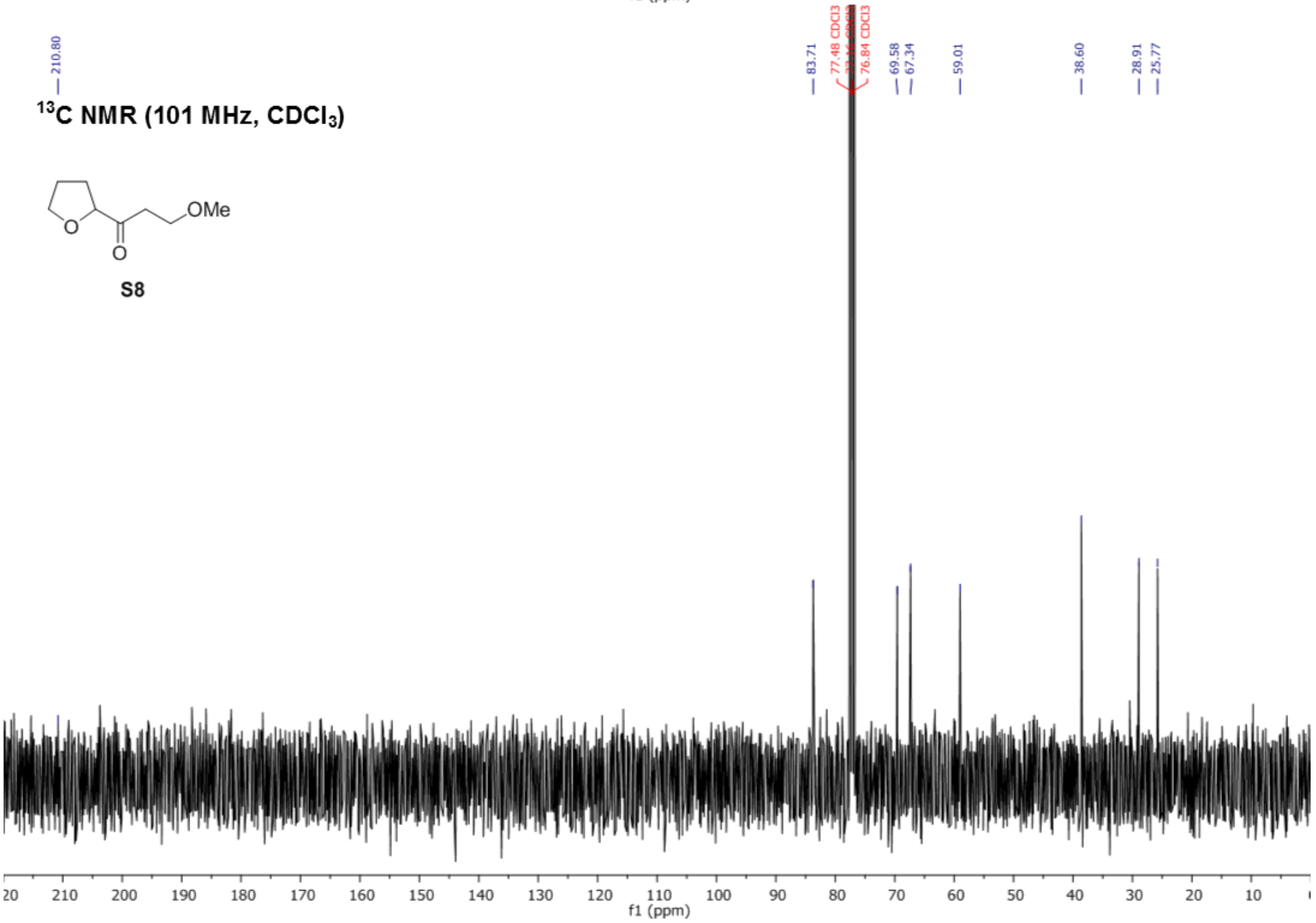

^{13}C NMR (101 MHz, $CDCl_3$)
OMe
S8
f1 (ppm)

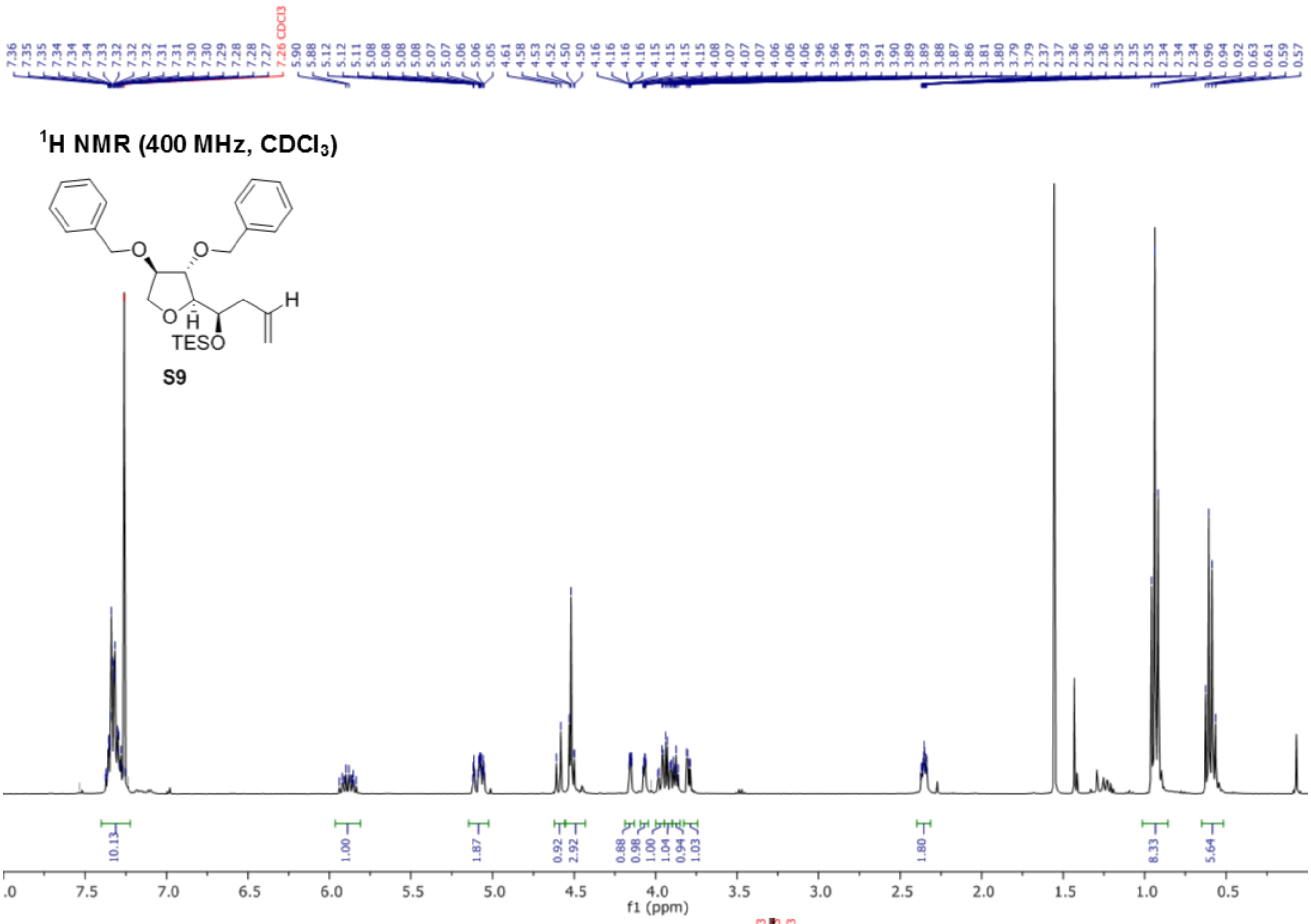
7.36 7.35 7.35 7.34 7.34 7.33 7.32 7.32 7.32 7.31 7.31 7.30 7.30 7.29 7.28 7.28 7.27
7.26 CDCl3
5.90 5.88 5.12 5.12 5.11 5.08 5.08 5.08 5.08 5.07 5.07 5.06 5.06 5.05 4.61 4.58 4.53 4.52 4.50 4.50 4.16 4.16 4.16 4.15 4.15 4.15 4.15 4.08 4.07 4.07 4.07 4.06 4.06 4.06 3.96 3.96 3.94 3.93 3.91 3.90 3.89 3.89 3.88 3.87 3.86 3.81 3.80 3.79 2.37 2.37 2.36 2.36 2.35 2.35 2.35 2.34 2.34 2.34 0.96 0.94 0.92 0.63 0.61 0.59 0.57
^{1}H NMR (400 MHz, CDCl$_3$)
O
O
O
H
H
TESO
S9
10.13
1.00
1.87
0.92
2.92
0.88
0.98
1.00
1.04
0.94
1.03
1.80
8.33
5.64
f1 (ppm)

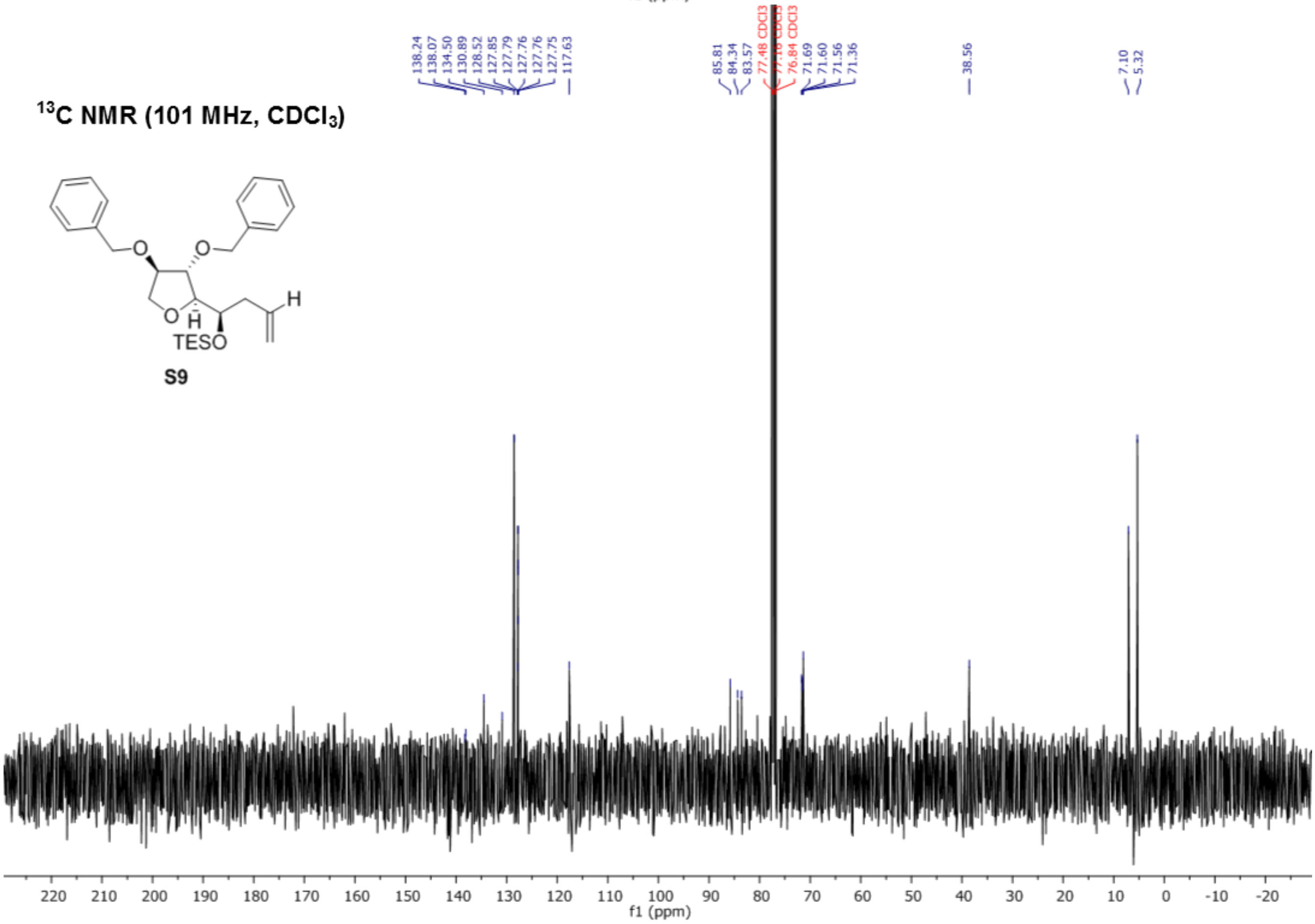
138.24 138.07 134.50 130.89 128.52 127.85 127.79 127.76 127.76 127.75 117.63
85.81 84.34 83.57
77.48 CDCl3
77.16 CDCl3
76.84 CDCl3
71.69 71.60 71.56 71.36
38.56
7.10 5.32
^{13}C NMR (101 MHz, CDCl$_3$)
O
O
O
H
H
TESO
S9
f1 (ppm)

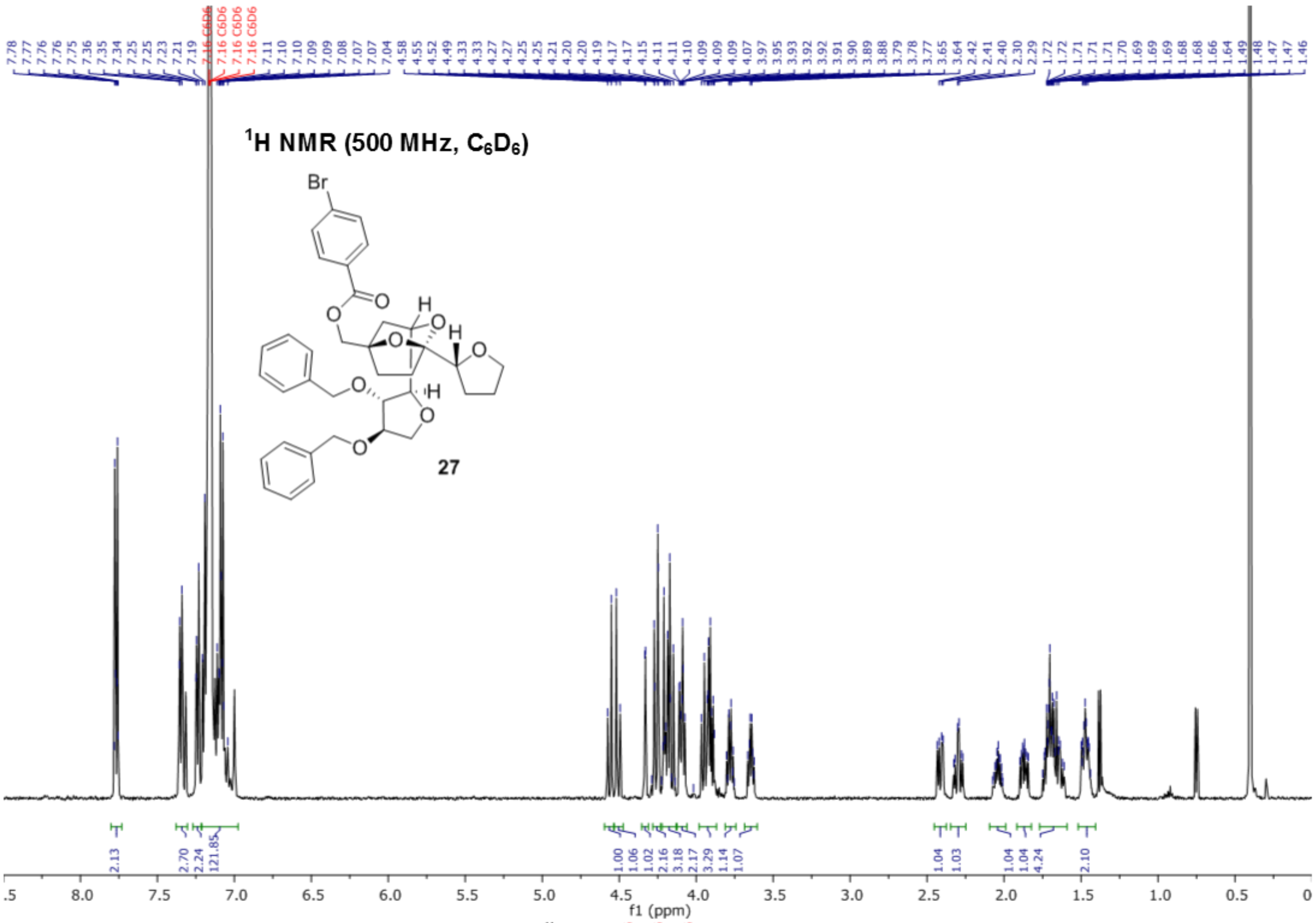

^{1}H NMR (500 MHz, C_6D_6)
27
f1 (ppm)

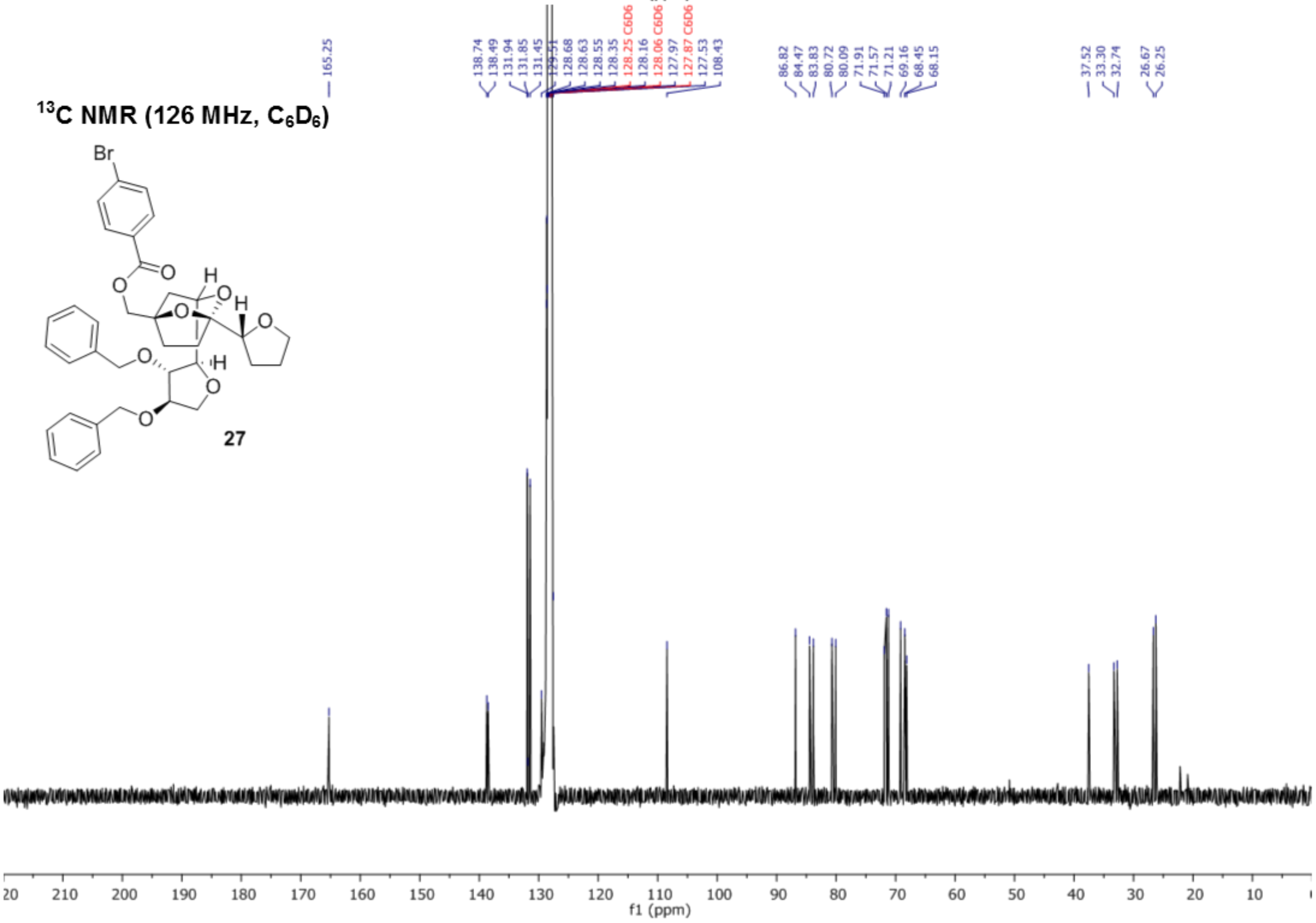

^{13}C NMR (126 MHz, C_6D_6)
27
f1 (ppm)

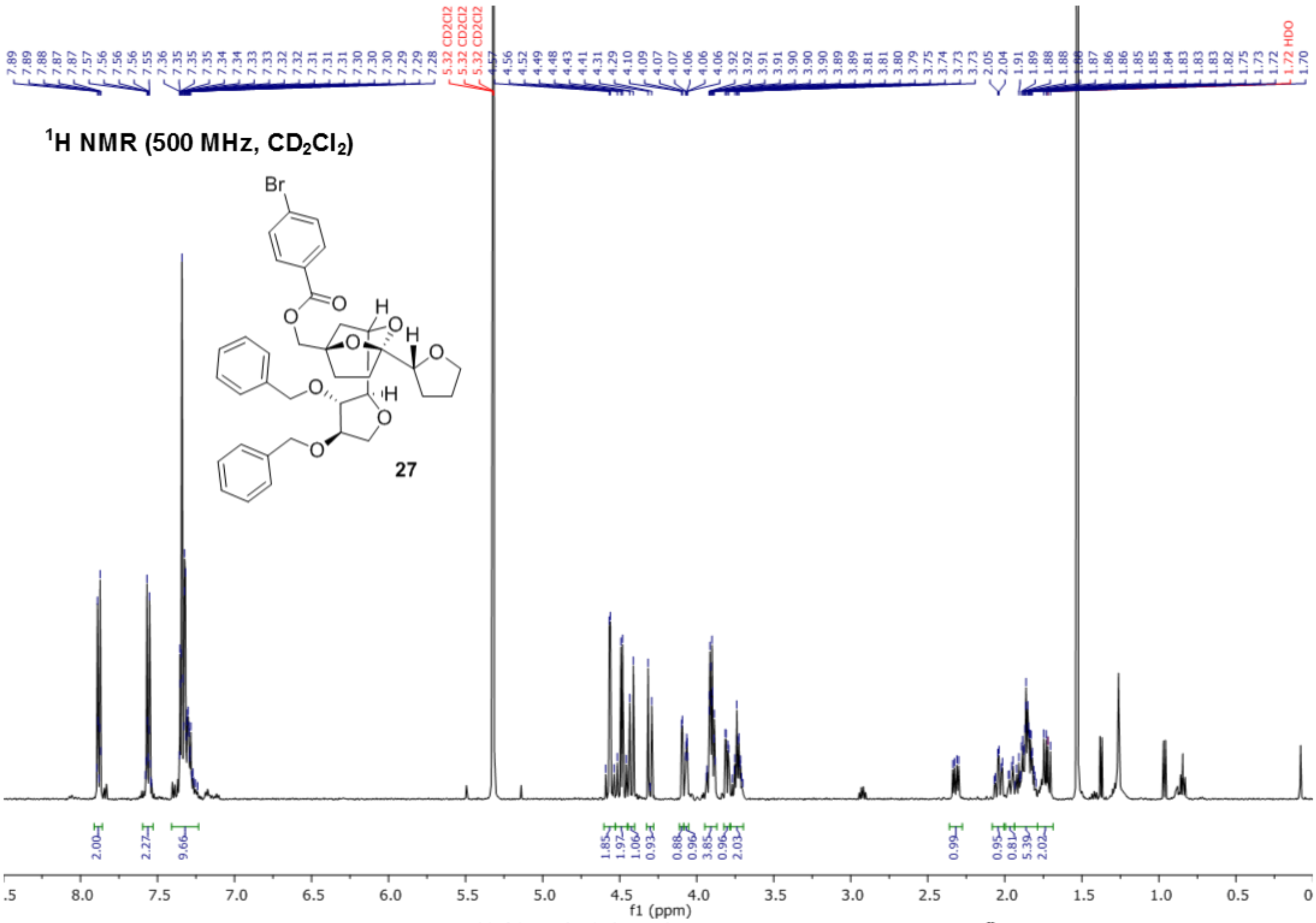
^{1}H NMR (500 MHz, CD_2Cl_2)
27
f1 (ppm)

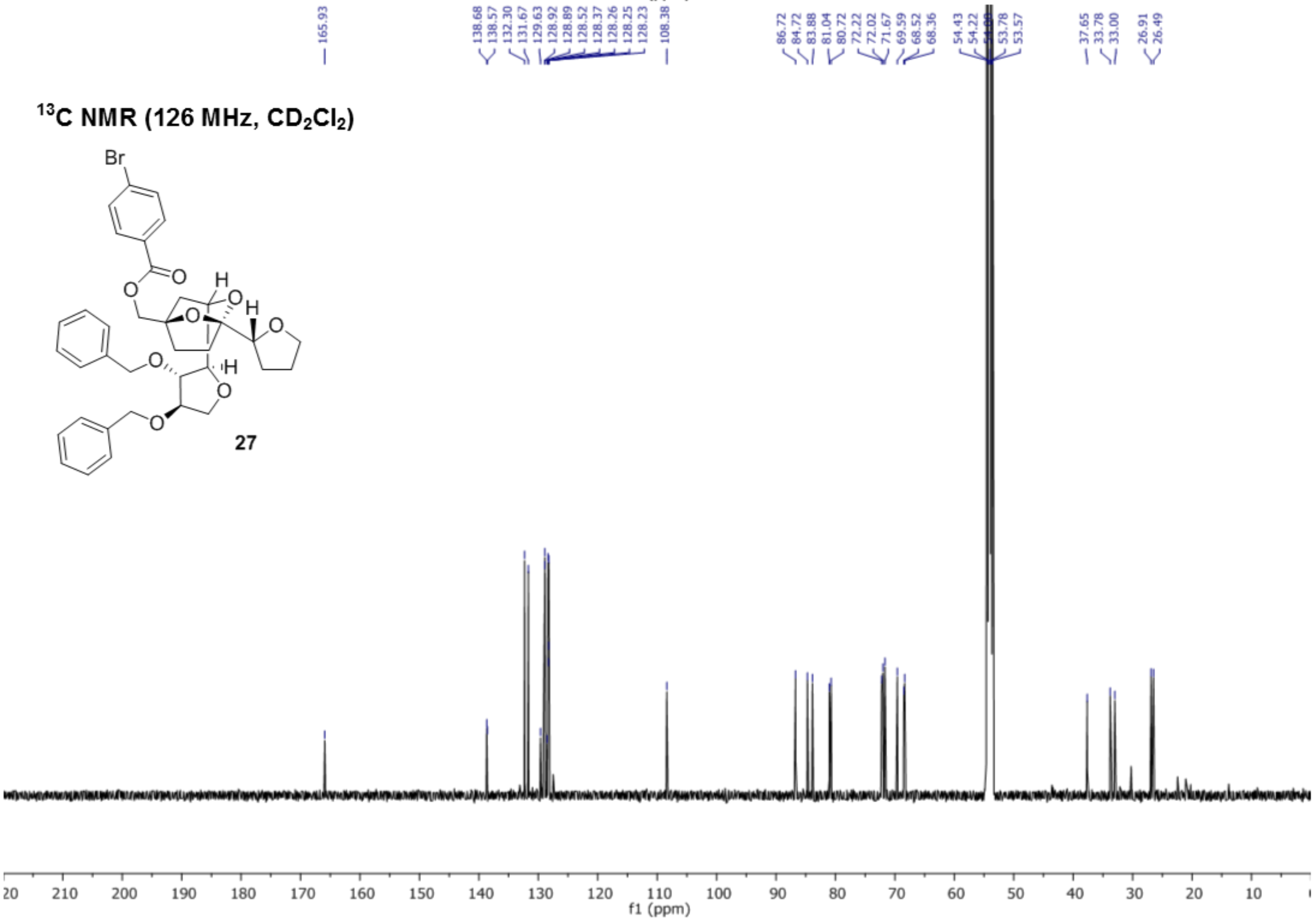
^{13}C NMR (126 MHz, CD_2Cl_2)
27
f1 (ppm)

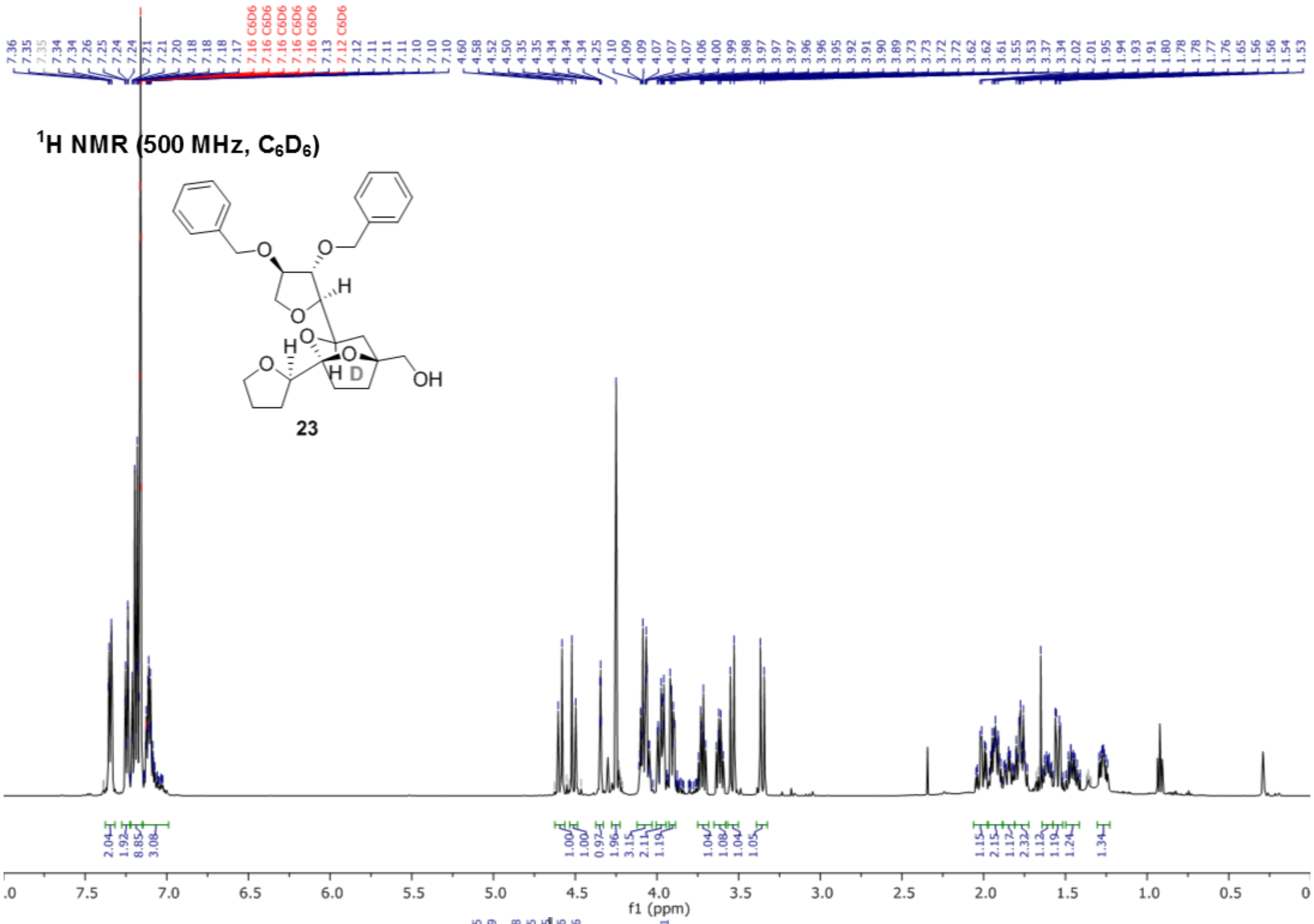

7.36 7.35 7.35 7.34 7.34 7.26 7.25 7.24 7.24 7.21 7.21 7.20 7.18 7.18 7.18 7.17 7.16 C6D6 7.16 C6D6 7.16 C6D6 7.16 C6D6 7.16 C6D6 7.13 7.12 C6D6 7.12 7.11 7.11 7.11 7.10 7.10 4.60 4.58 4.52 4.50 4.35 4.35 4.34 4.34 4.34 4.25 4.10 4.09 4.09 4.07 4.07 4.06 4.00 3.99 3.98 3.97 3.97 3.96 3.96 3.95 3.92 3.91 3.90 3.89 3.73 3.73 3.72 3.72 3.62 3.61 3.55 3.53 3.37 3.34 2.02 2.01 1.95 1.94 1.93 1.91 1.80 1.78 1.78 1.77 1.76 1.65 1.56 1.56 1.54 1.53
1H NMR (500 MHz, C6D6)
O
O
H
O
H
O
O
H
D
OH
O
23
2.04 1.92 8.85 3.08 1.00 1.00 0.97 1.96 3.15 2.11 1.19 1.04 1.08 1.04 1.05 1.15 2.15 1.17 2.32 1.12 1.19 1.24 1.34
f1 (ppm)

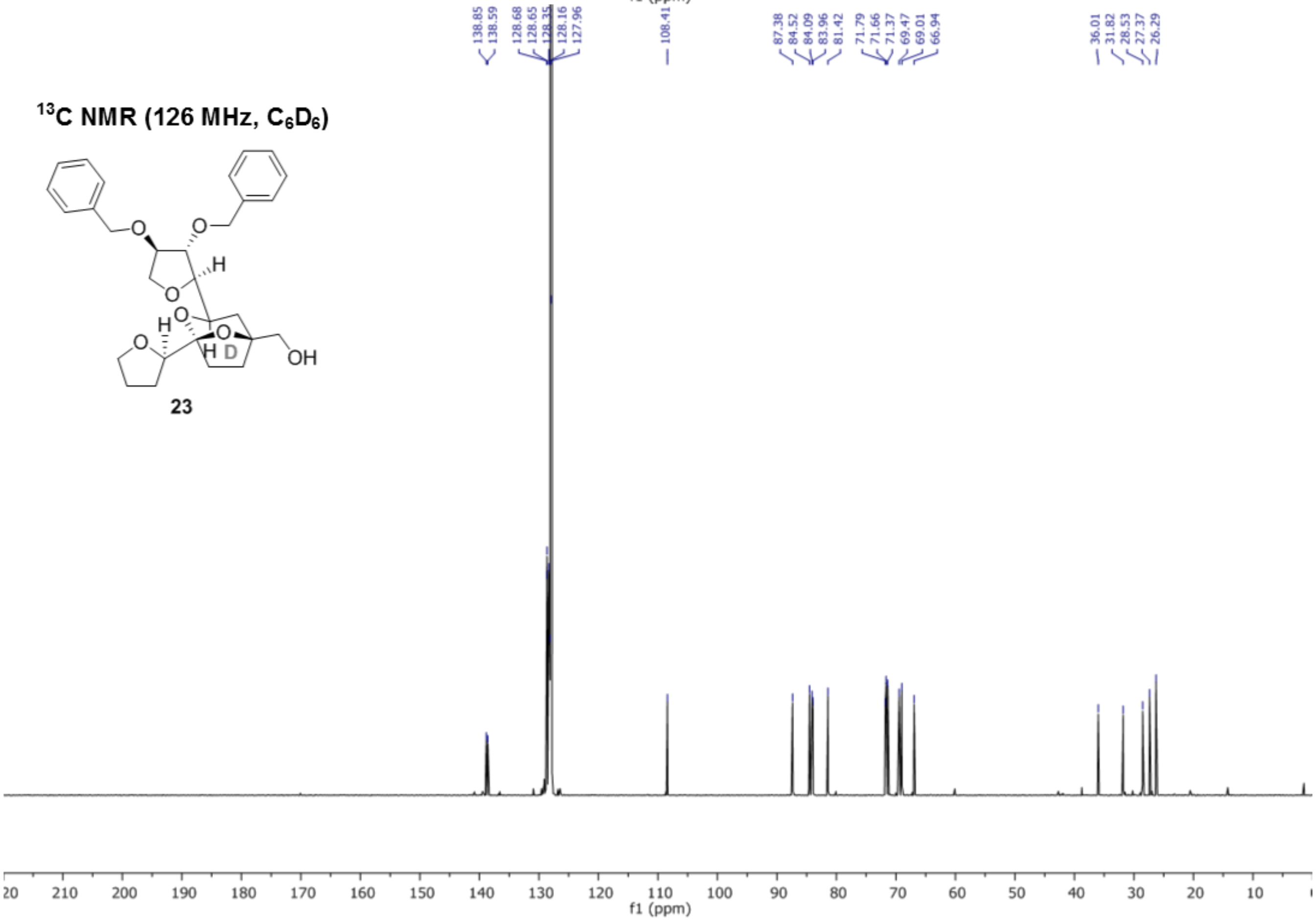

138.85 138.59 128.68 128.65 128.35 128.16 127.96 108.41 87.38 84.52 84.09 83.96 81.42 71.79 71.66 71.37 69.47 69.01 66.94 36.01 31.82 28.53 27.37 26.29
13C NMR (126 MHz, C6D6)
O
O
H
O
H
O
O
H
D
OH
O
23
f1 (ppm)

^{1}H NMR (500 MHz, CD_2Cl_2)

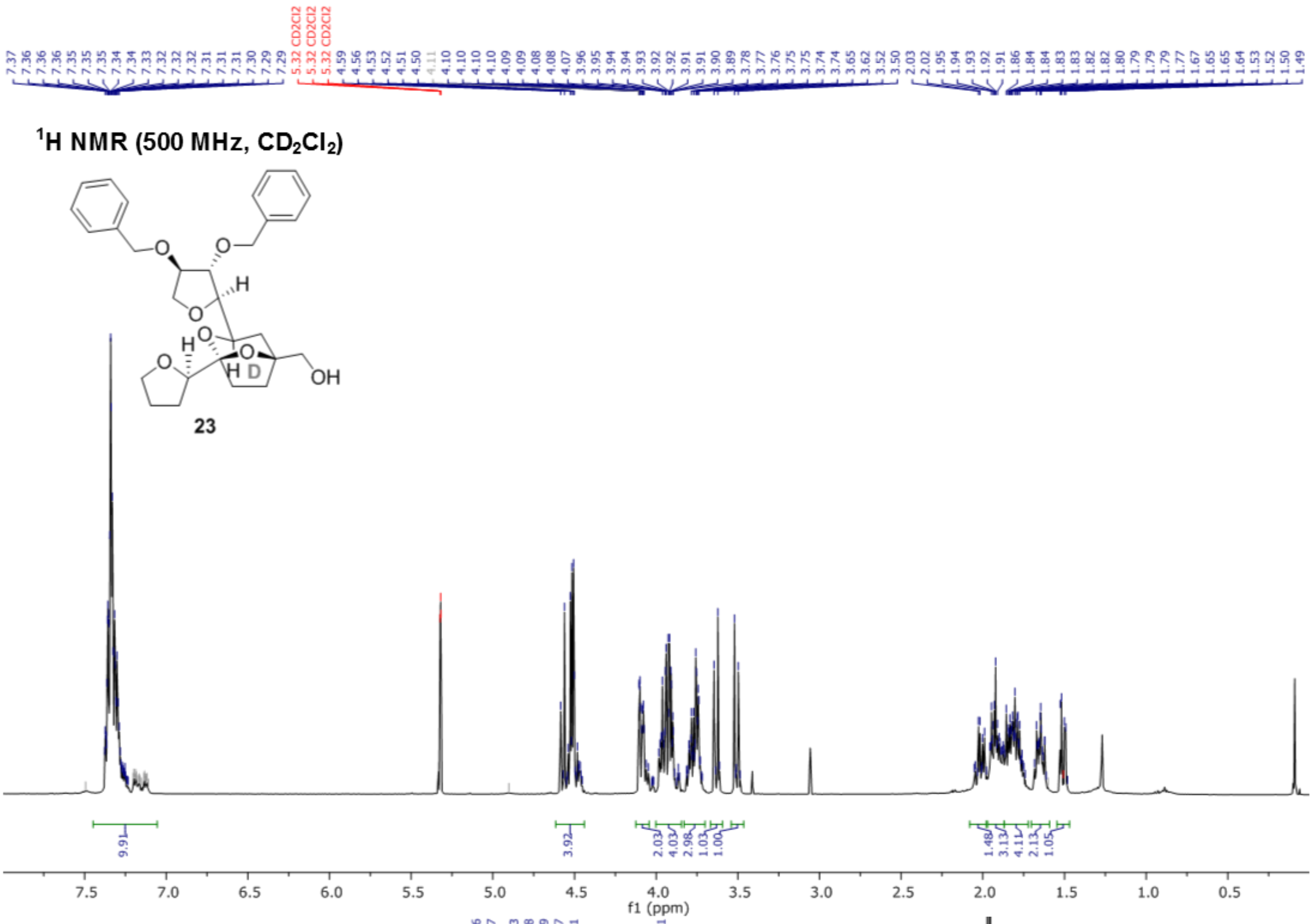

23

^{13}C NMR (126 MHz, CD_2Cl_2)

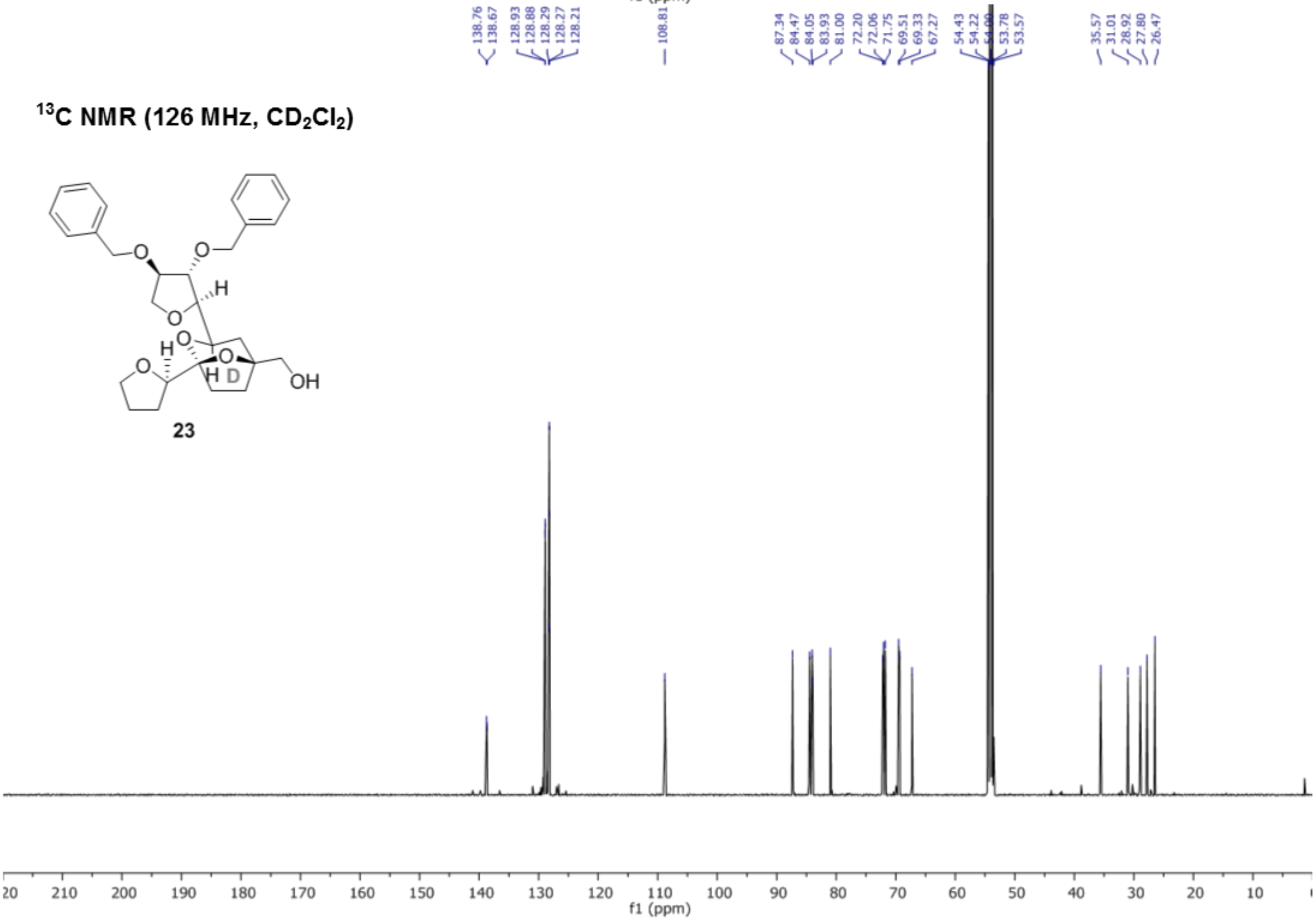

23

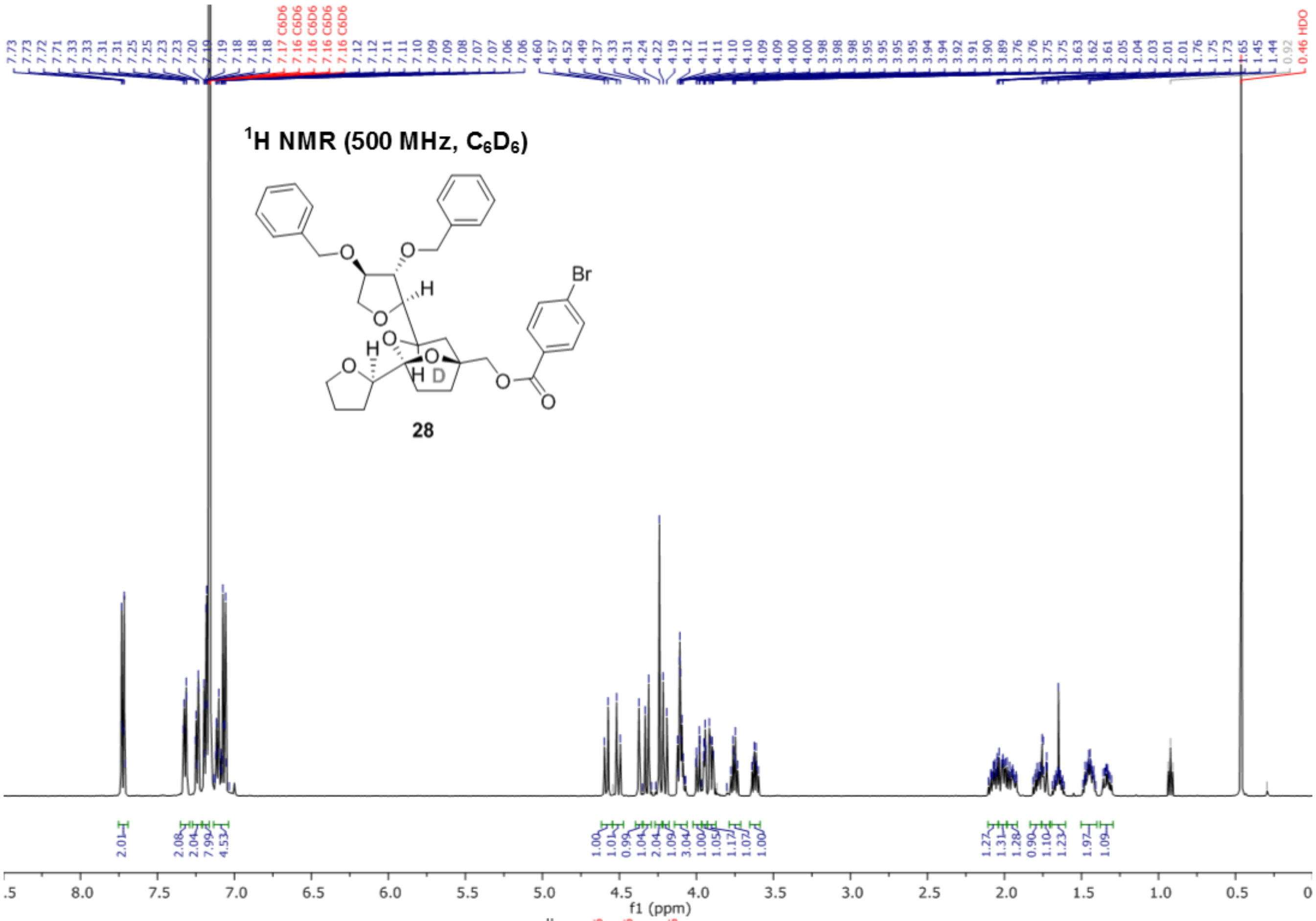

^{1}H NMR (500 MHz, C_6D_6)
Br
H
O
D
28
f1 (ppm)

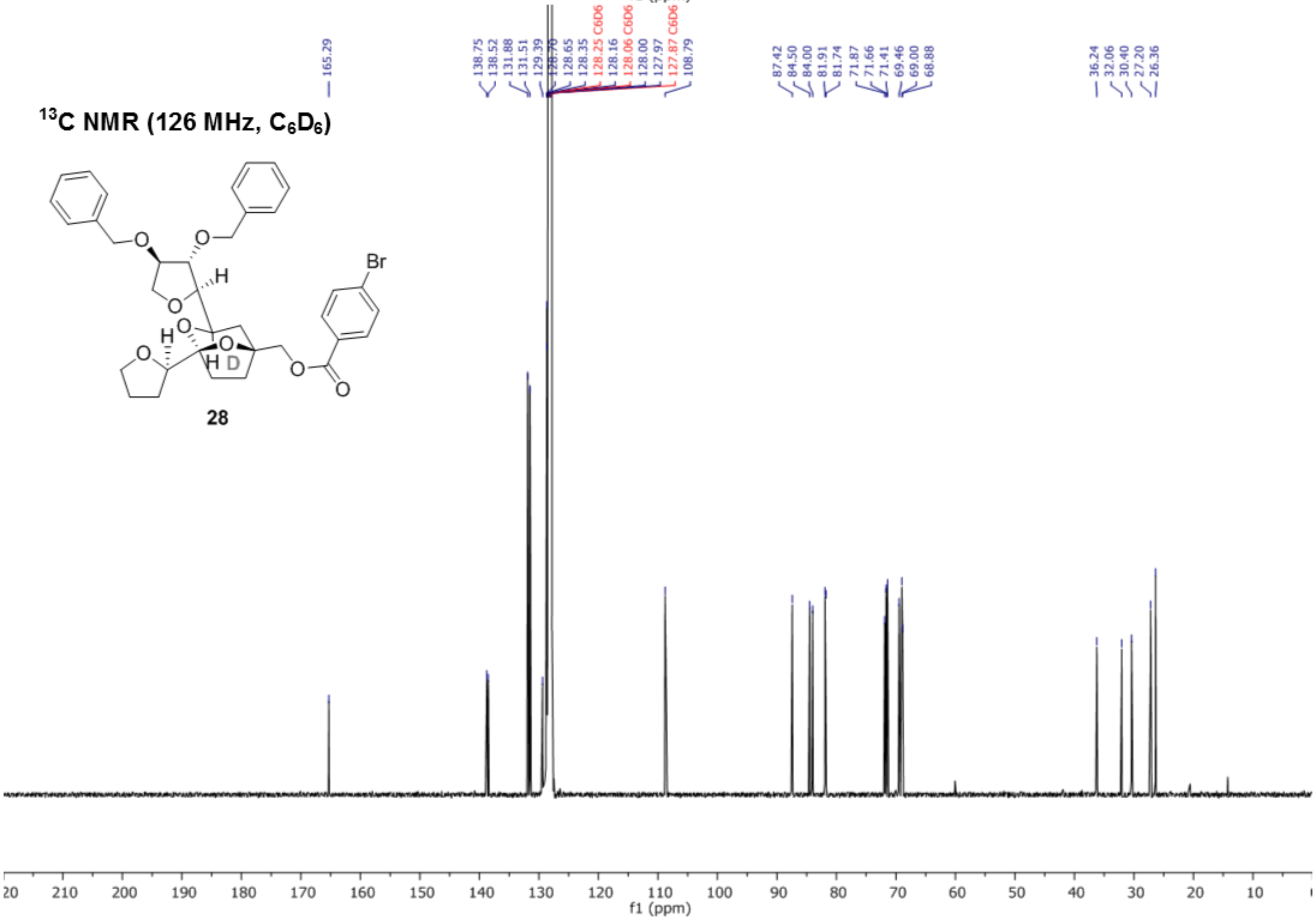

^{13}C NMR (126 MHz, C_6D_6)
Br
H
O
D
28
f1 (ppm)

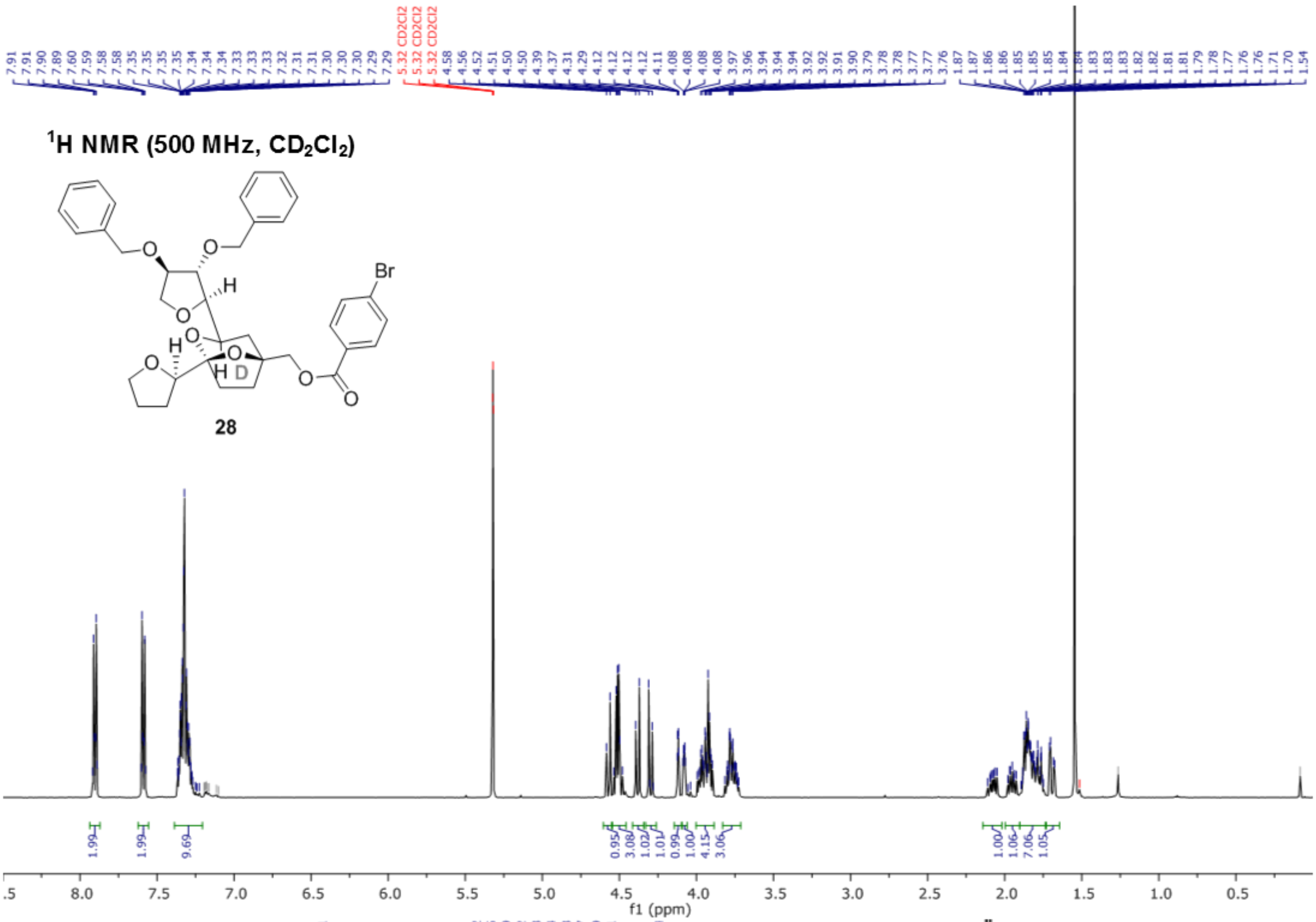
7.91 7.91 7.90 7.89 7.60 7.59 7.58 7.58 7.35 7.35 7.35 7.34 7.34 7.34 7.33 7.33 7.33 7.32 7.31 7.31 7.30 7.30 7.30 7.29
5.32 CD2Cl2 5.32 CD2Cl2 5.32 CD2Cl2
4.58 4.56 4.52 4.51 4.50 4.50 4.39 4.37 4.31 4.29 4.12 4.12 4.12 4.11 4.08 4.08 4.08 3.97 3.96 3.94 3.94 3.94 3.92 3.92 3.91 3.90 3.79 3.78 3.78 3.77 3.77 3.76
1.87 1.87 1.86 1.85 1.85 1.85 1.84 1.84 1.83 1.83 1.83 1.82 1.82 1.81 1.81 1.79 1.78 1.77 1.76 1.71 1.70 1.54
1H NMR (500 MHz, CD2Cl2)
Br
H
O
D
28
1.99 1.99 9.69 0.95 3.08 1.02 1.01 0.99 1.00 4.15 3.06 1.00 1.06 7.06 1.05
f1 (ppm)

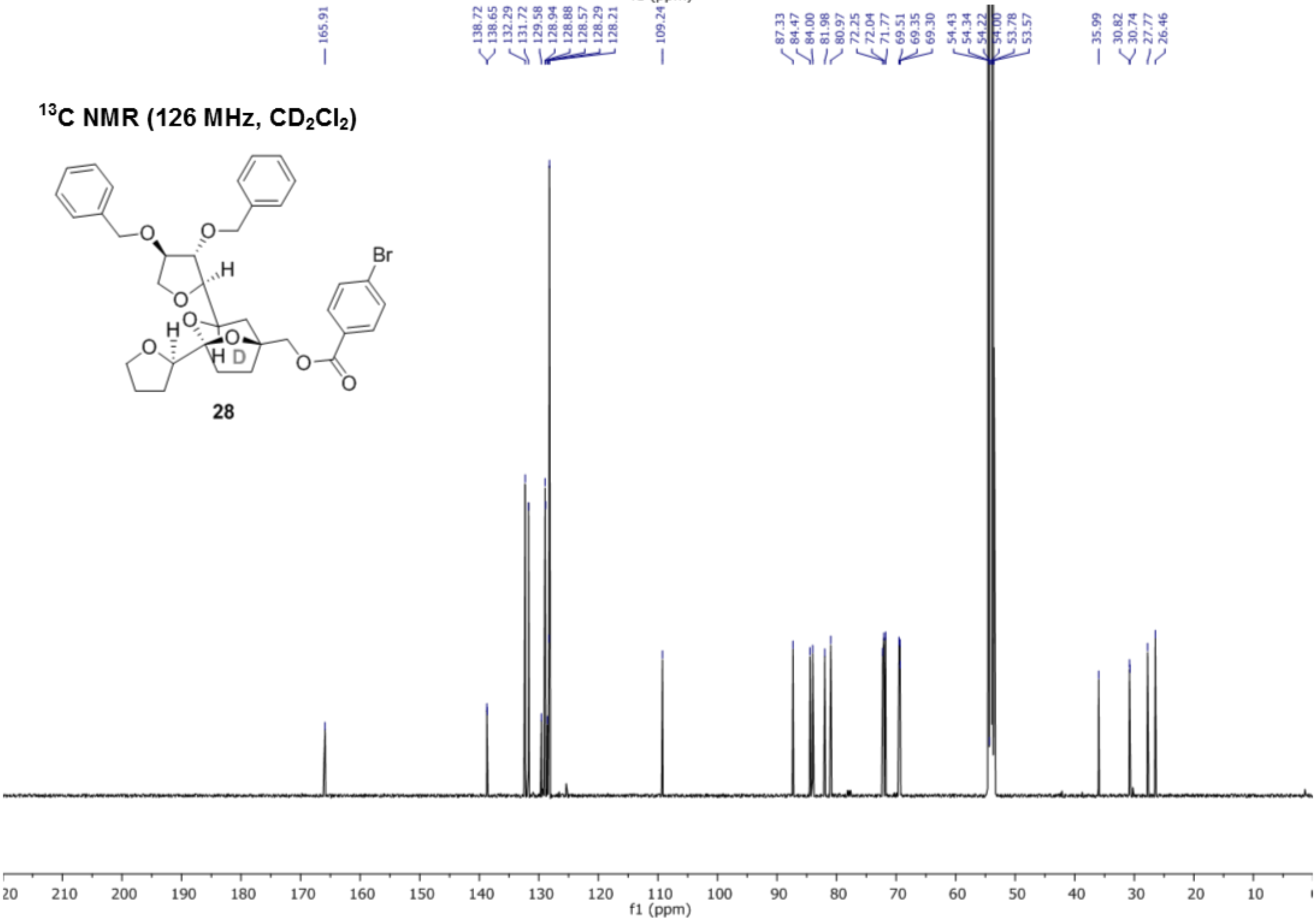
165.91
138.72 138.65 132.29 131.72 129.58 128.94 128.88 128.57 128.29 128.21
109.24
87.33 84.47 84.00 81.98 80.97 72.25 72.04 71.77 69.51 69.35 69.30 54.43 54.34 54.22 54.00 53.78 53.57
35.99 30.82 30.74 27.77 26.46
13C NMR (126 MHz, CD2Cl2)
Br
H
O
D
28
f1 (ppm)

^{1}H NMR (400 MHz, $CDCl_3$)

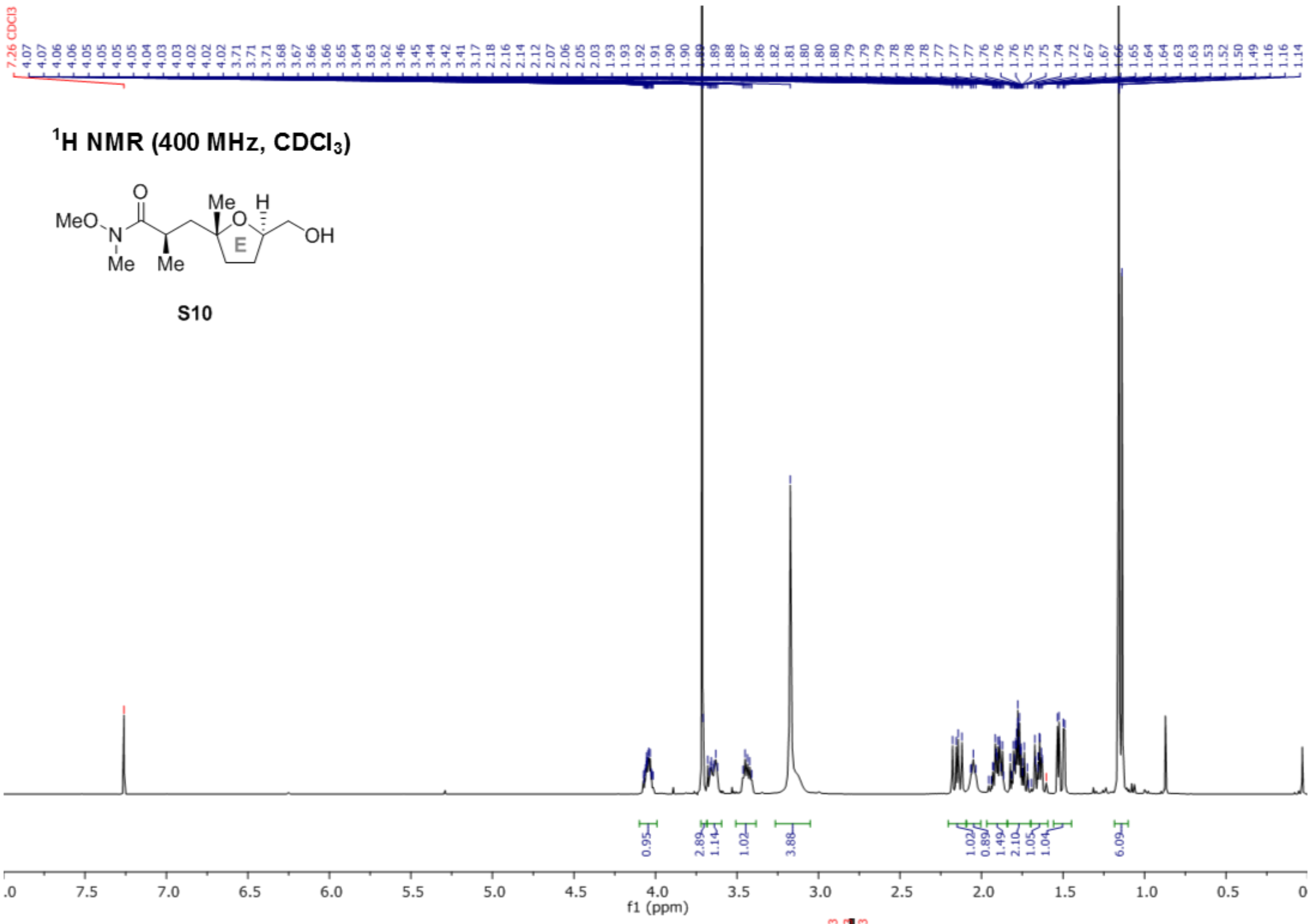

^{13}C NMR (101 MHz, $CDCl_3$)

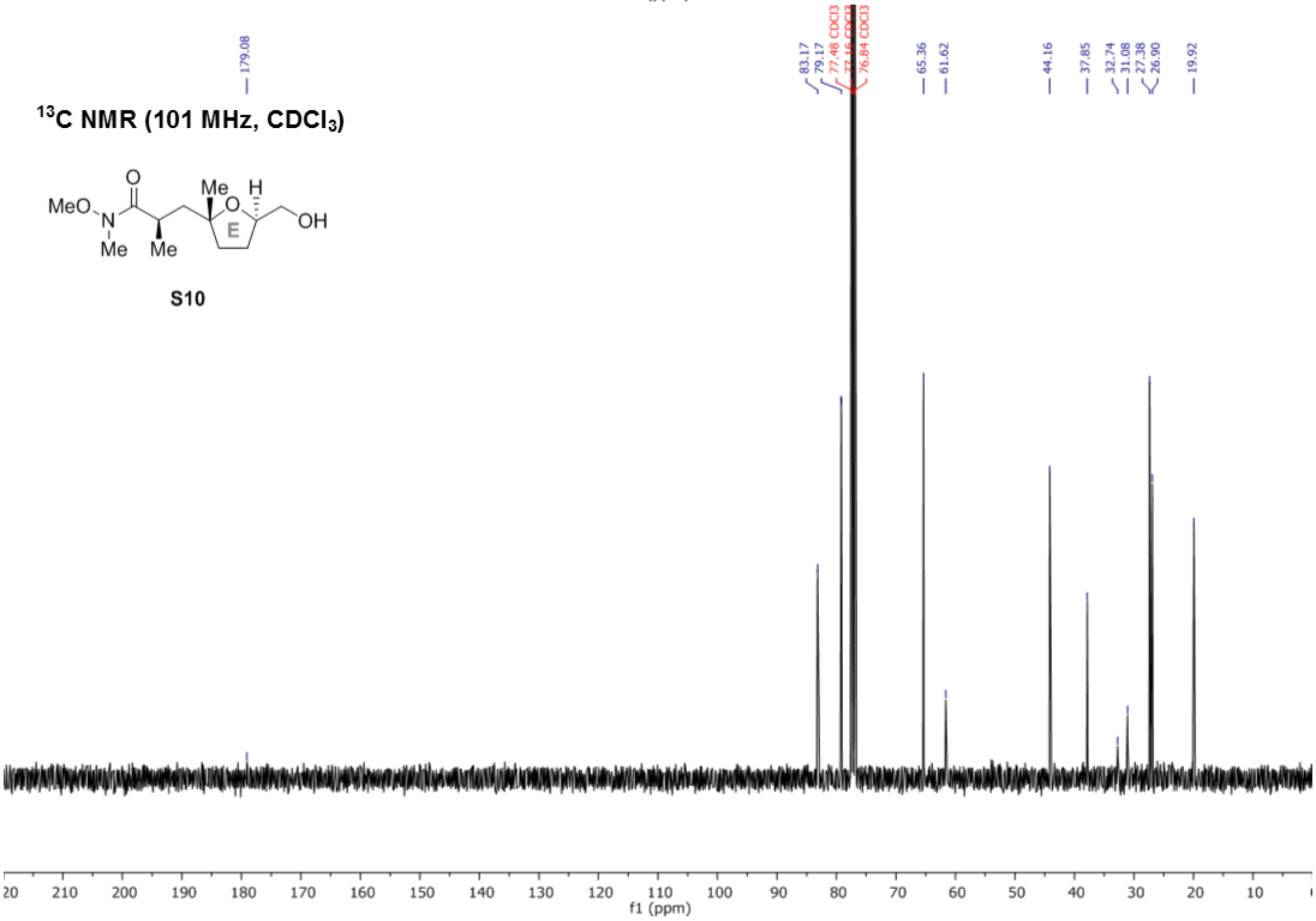

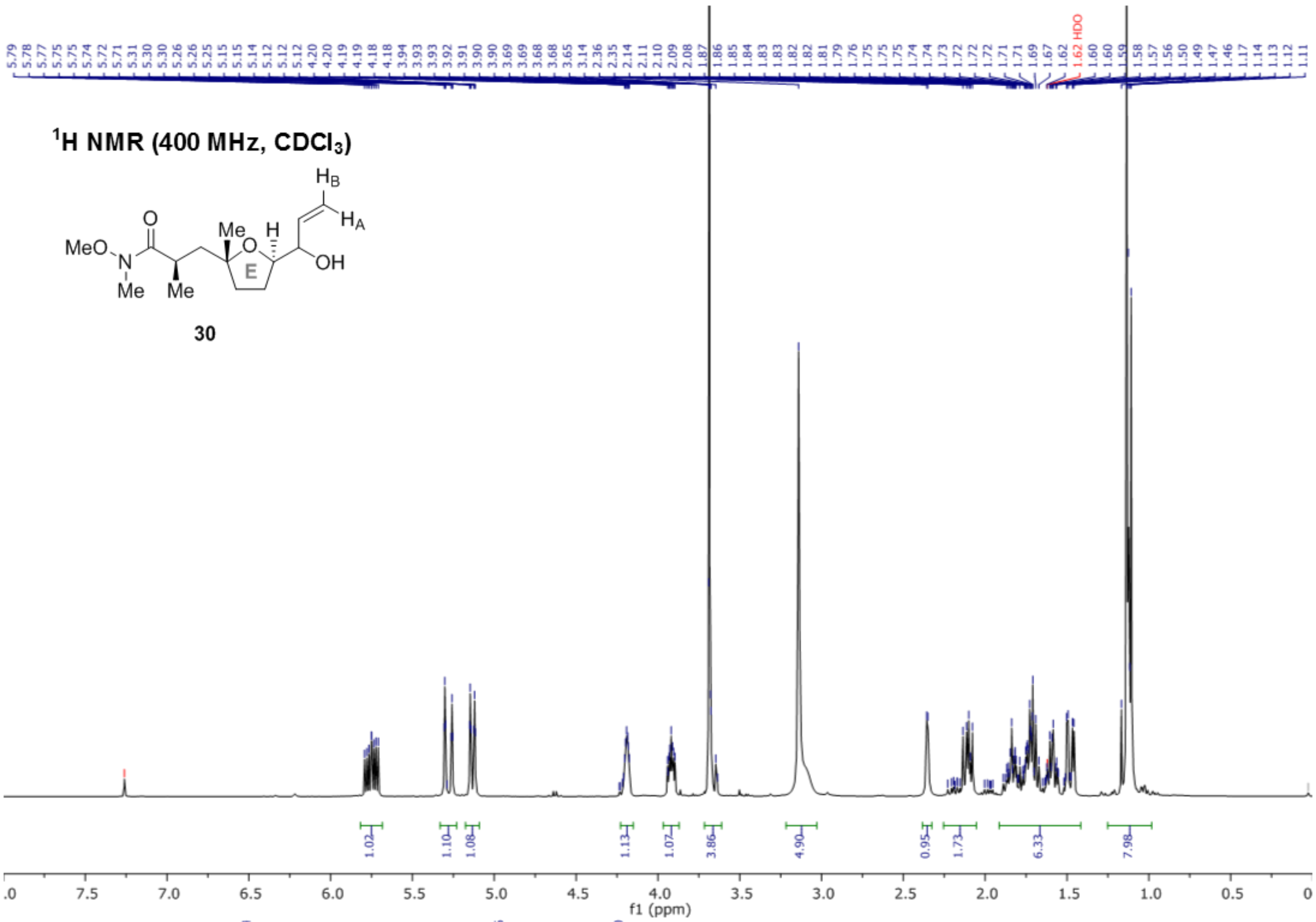
1H NMR (400 MHz, CDCl3)
30
f1 (ppm)

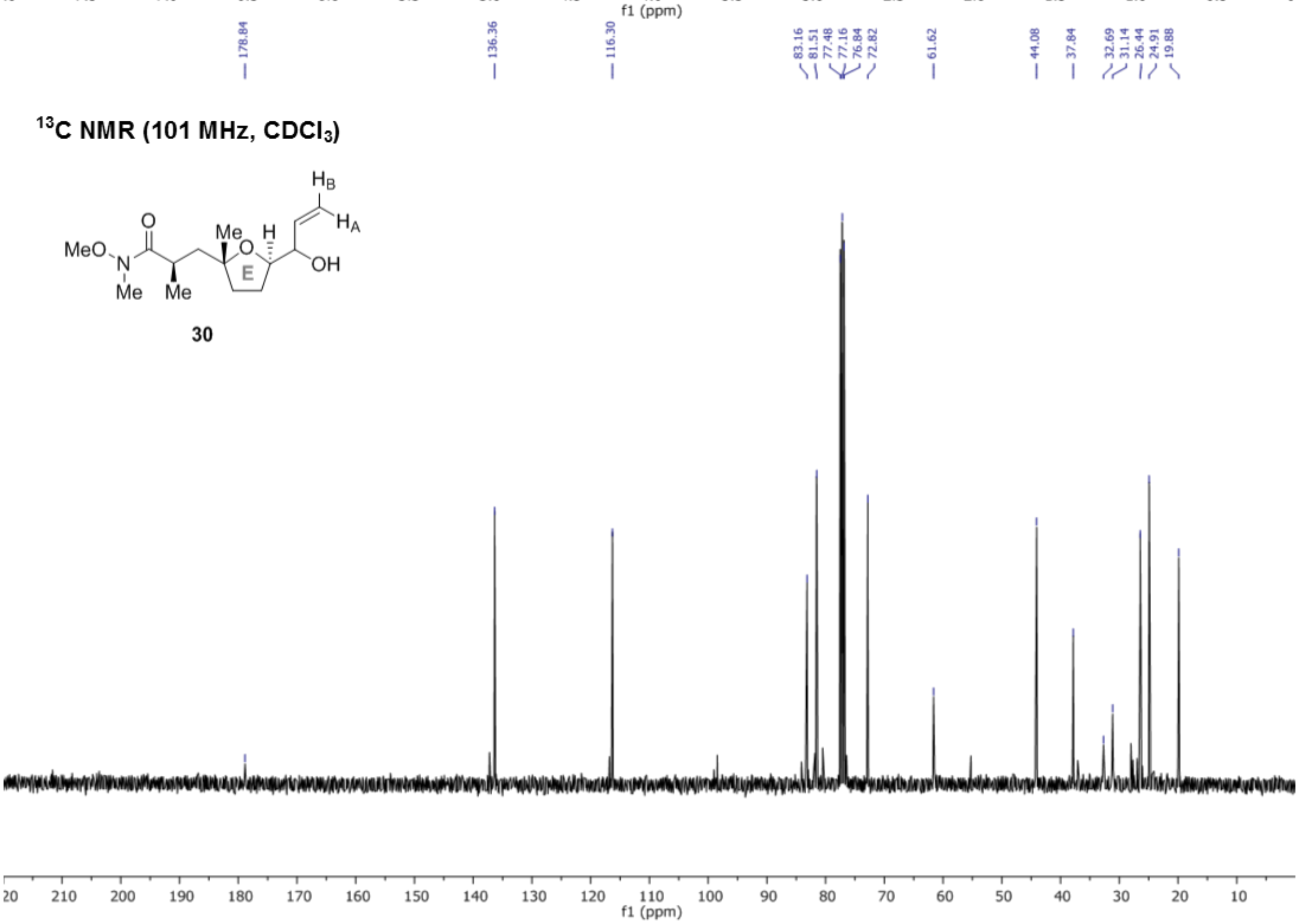
13C NMR (101 MHz, CDCl3)
30
f1 (ppm)

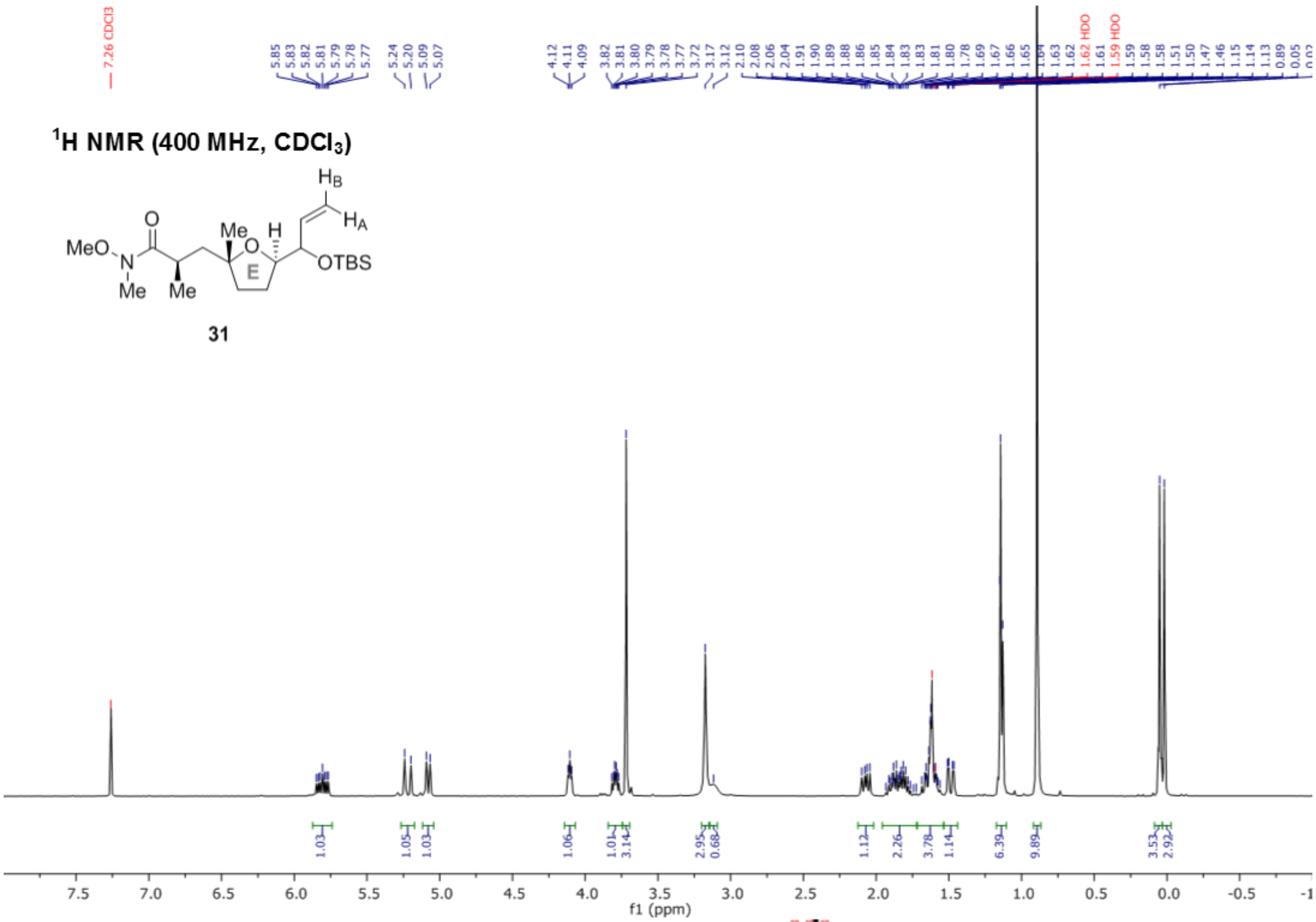

7.26 CDCl3
^{1}H NMR (400 MHz, CDCl$_3$)
MeO
N
Me
O
Me
Me
O
H
E
H$_B$
H$_A$
OTBS
31
1.03
1.05
1.03
1.06
1.01
3.14
2.95
0.68
1.12
2.26
3.78
1.14
6.39
9.89
3.53
2.92
f1 (ppm)

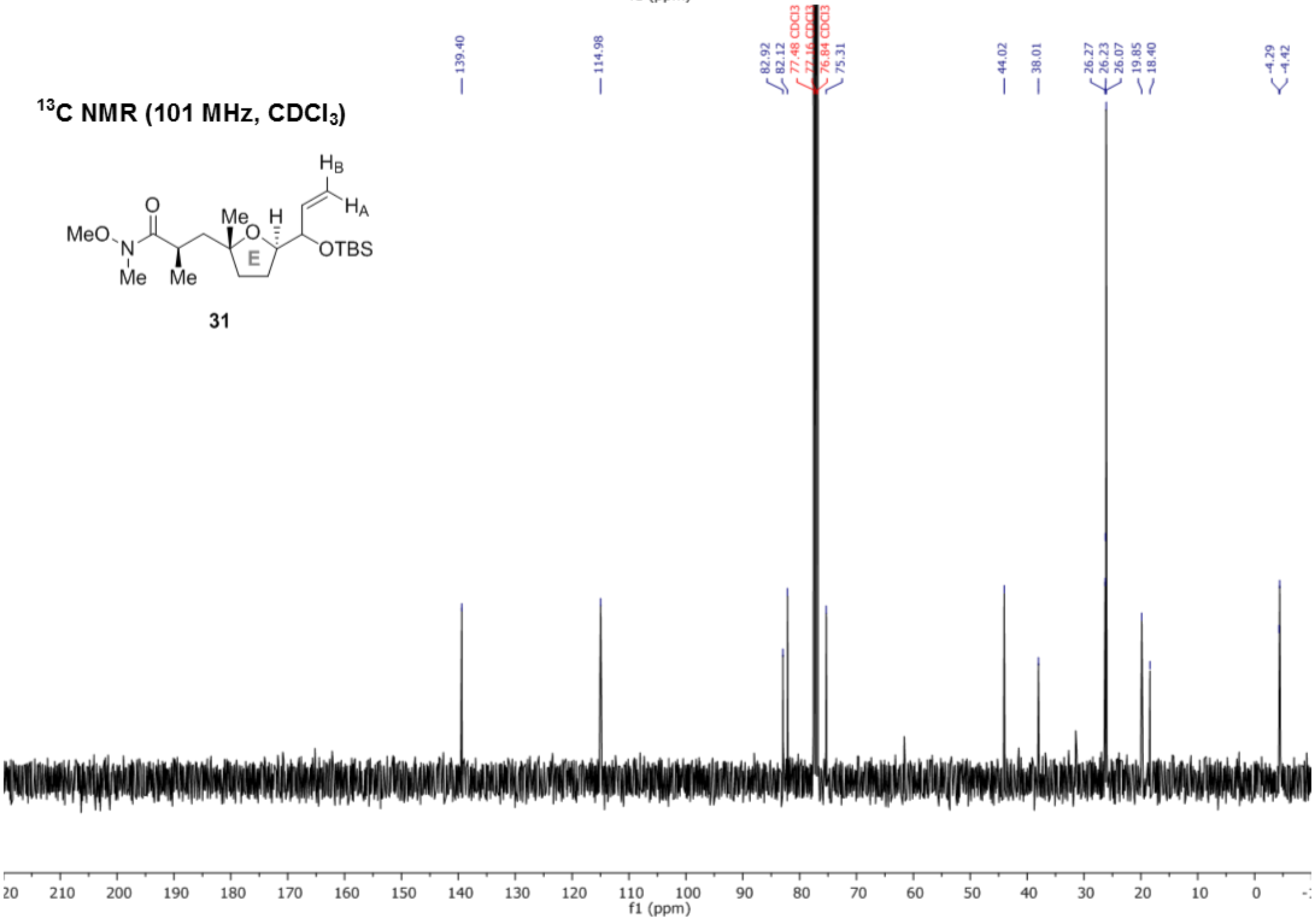

139.40
114.98
82.92
82.12
77.48 CDCl3
77.16 CDCl3
76.84 CDCl3
75.31
44.02
38.01
26.27
26.23
26.07
19.85
18.40
-4.29
-4.42
^{13}C NMR (101 MHz, CDCl$_3$)
MeO
N
Me
O
Me
Me
O
H
E
H$_B$
H$_A$
OTBS
31
f1 (ppm)

^{1}H NMR (500 MHz, $CDCl_3$)

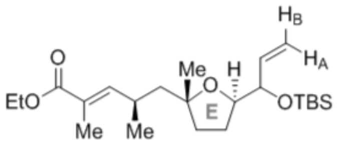

33

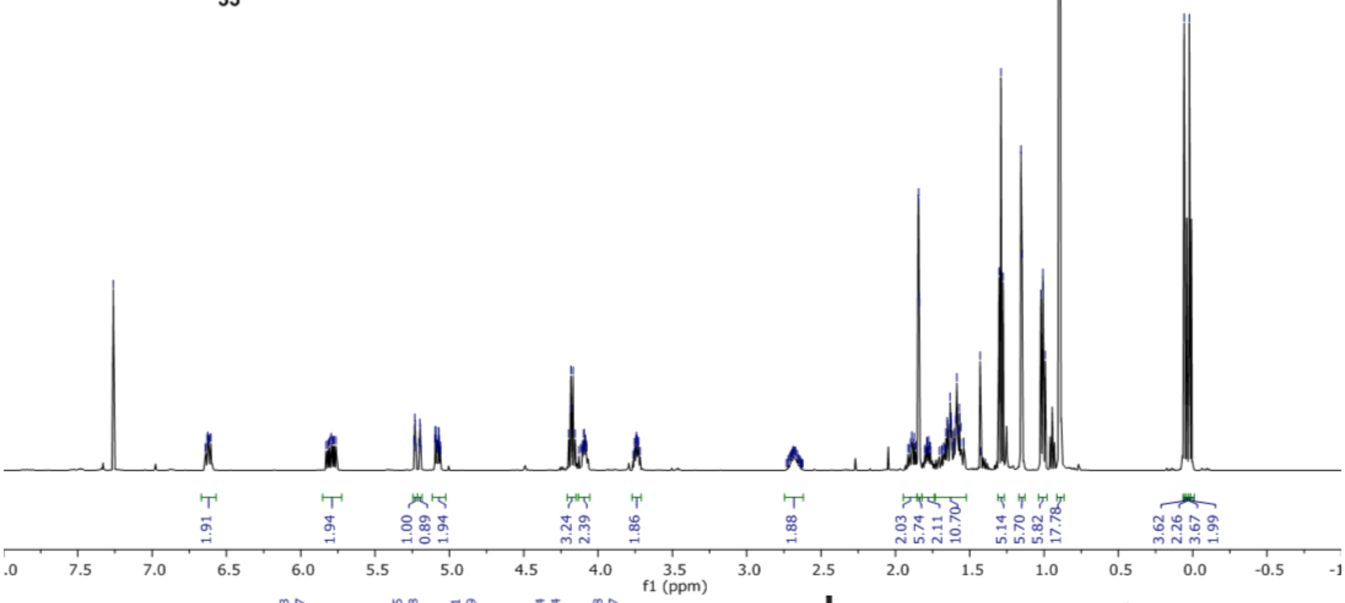

^{13}C NMR (126 MHz, $CDCl_3$)

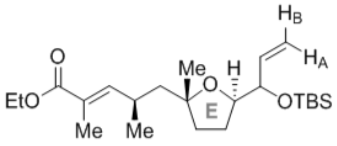

33

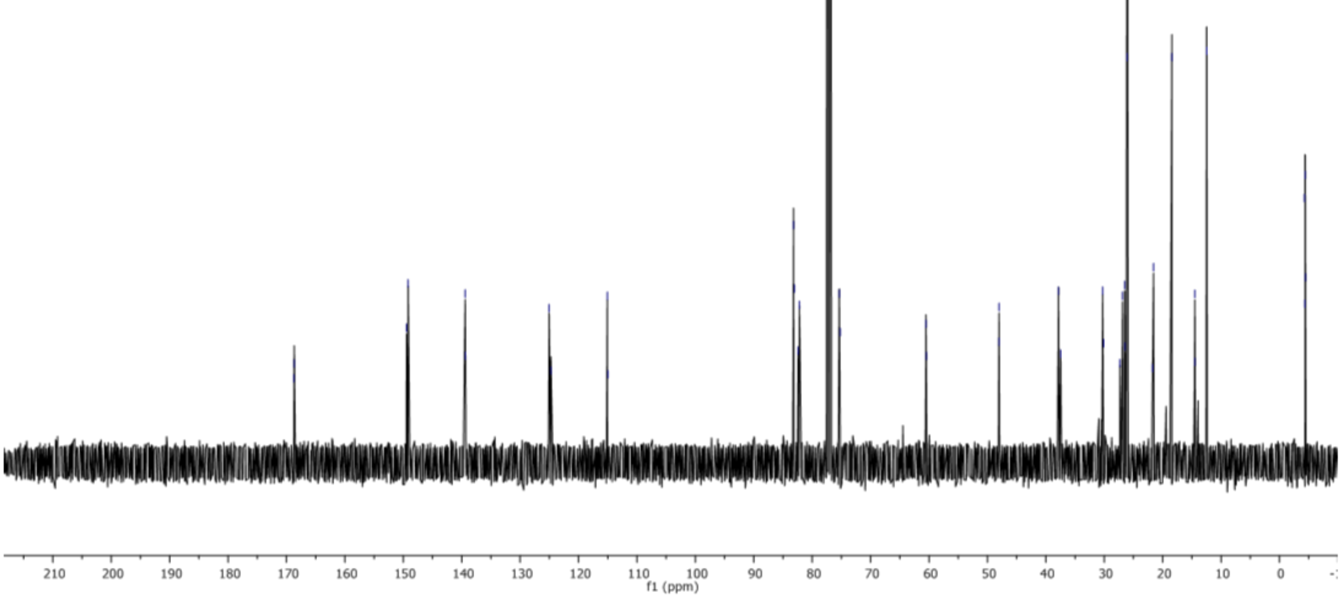

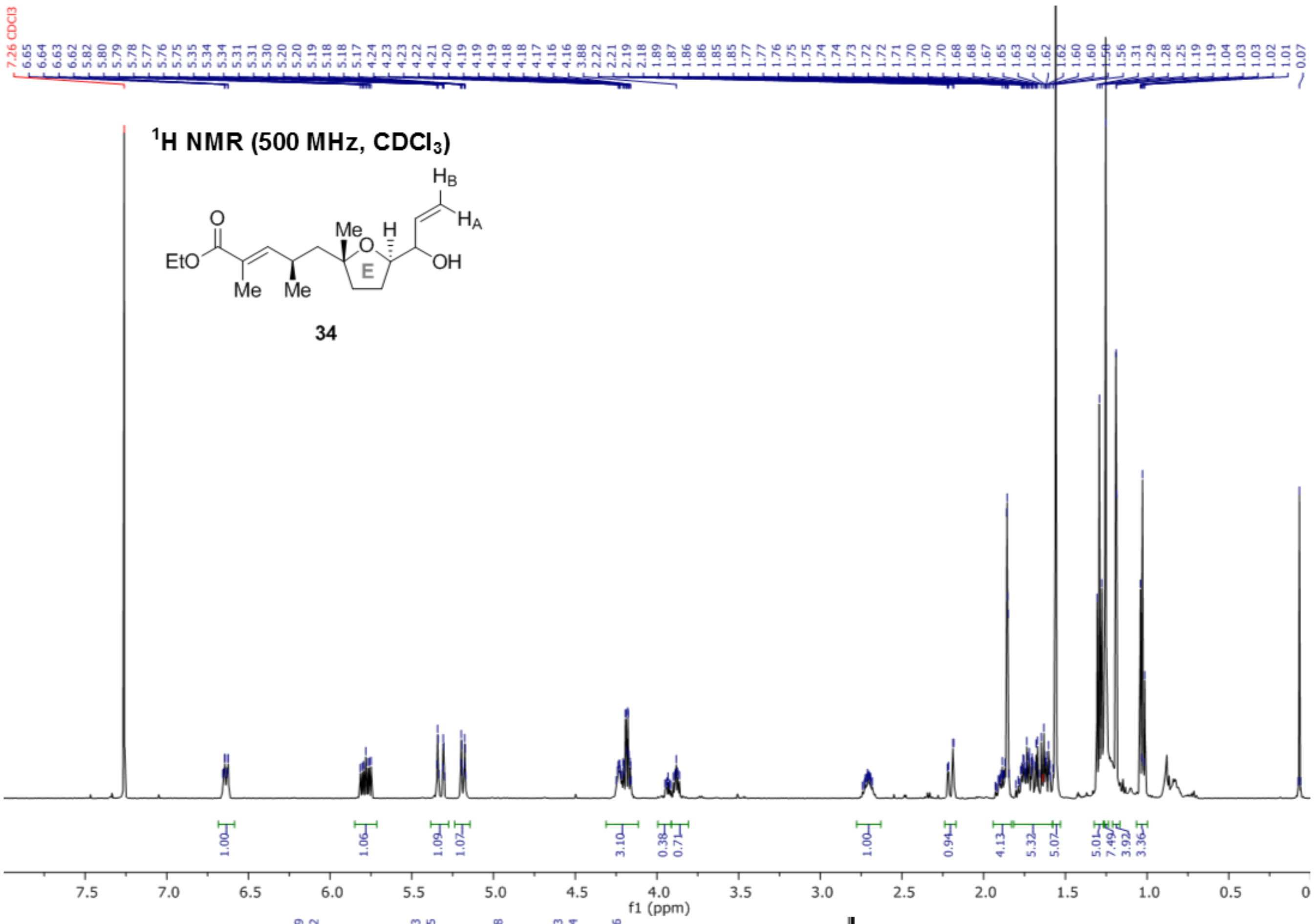

^{1}H NMR (500 MHz, CDCl$_3$)
34
f1 (ppm)

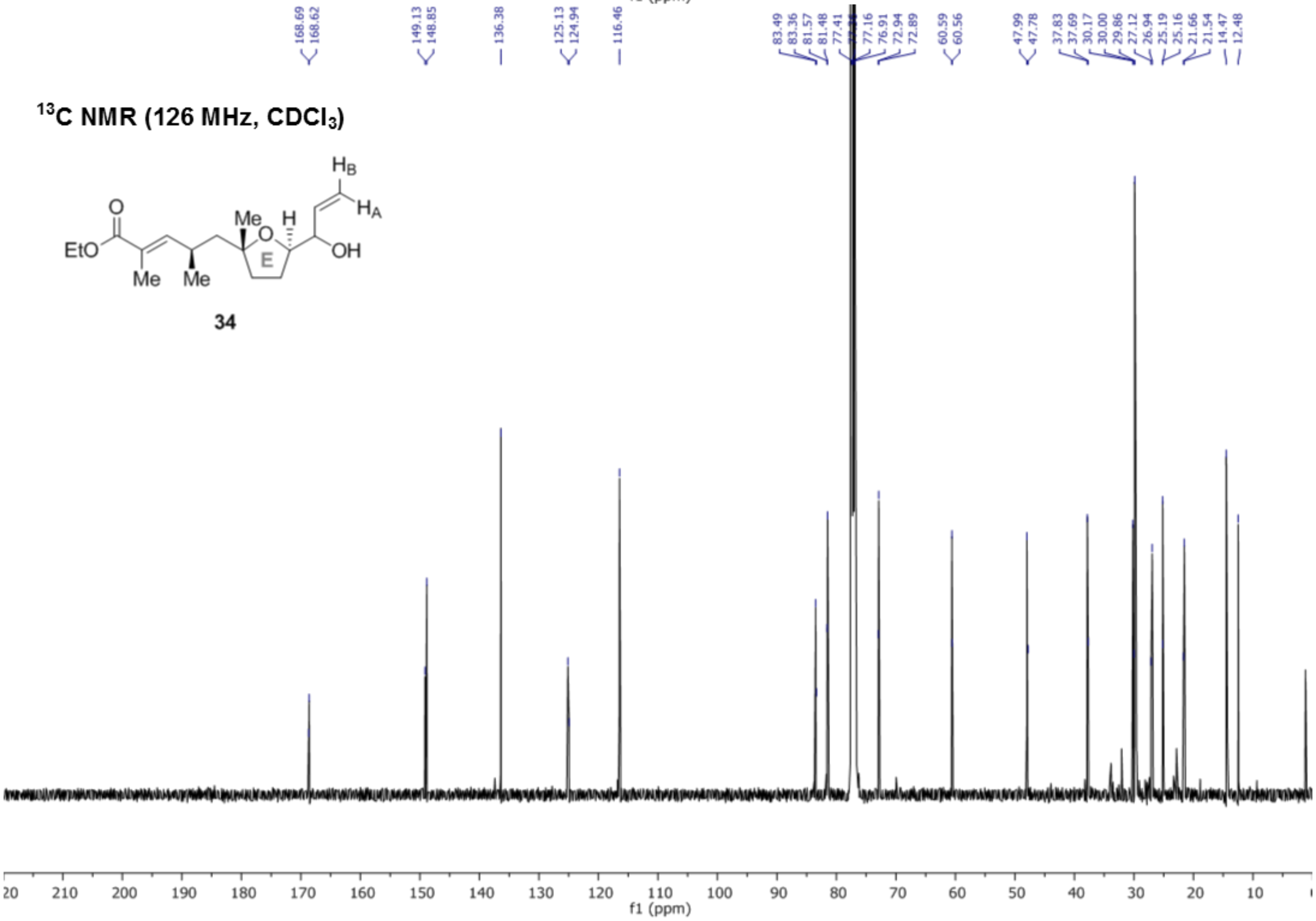

^{13}C NMR (126 MHz, CDCl$_3$)
34
f1 (ppm)

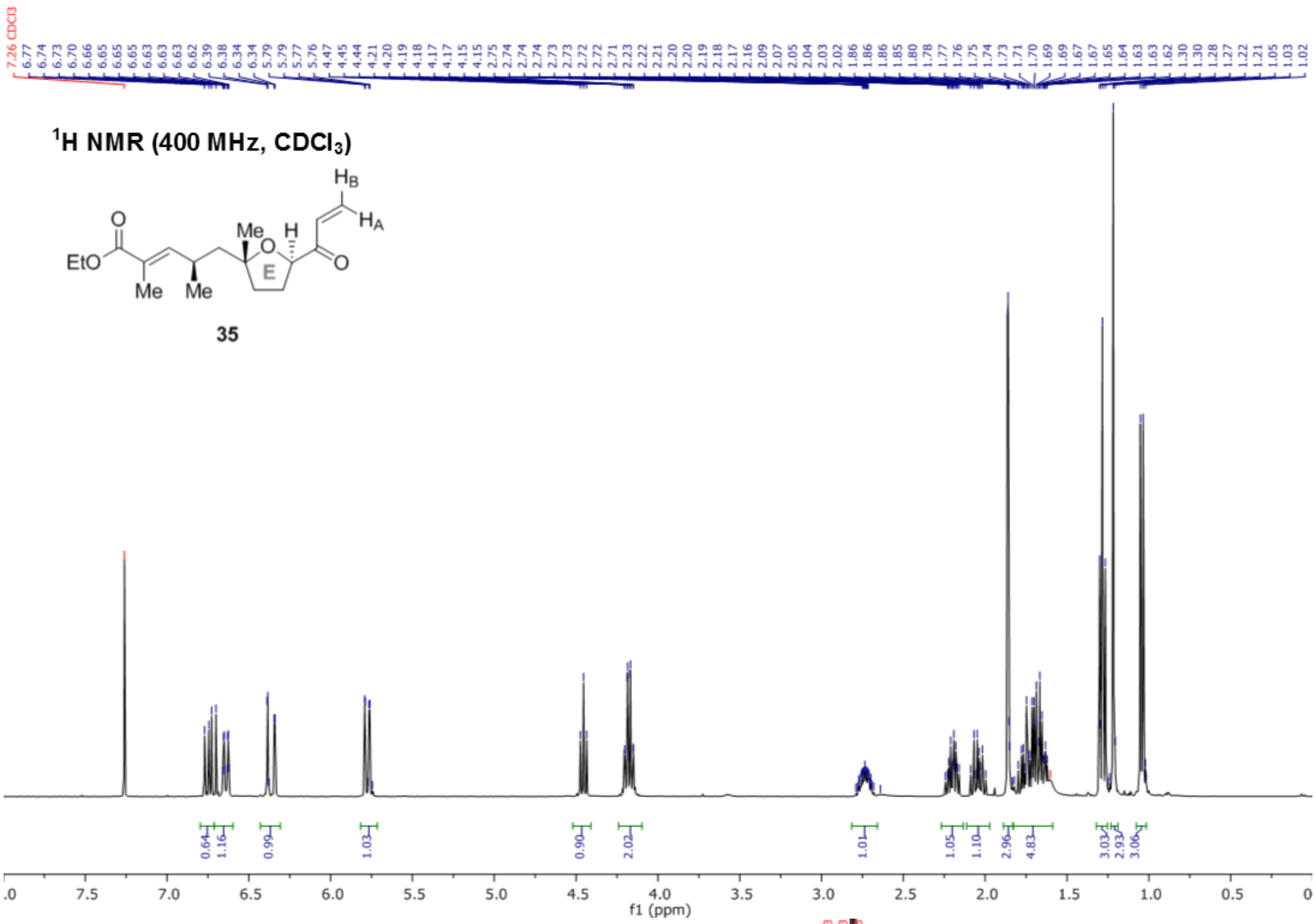
7.26 CDCl3
6.77 6.74 6.73 6.70 6.66 6.65 6.65 6.65 6.63 6.63 6.63 6.62 6.39 6.38 6.34 6.34 5.79 5.79 5.77 5.76 4.47 4.45 4.44 4.21 4.20 4.19 4.18 4.17 4.17 4.15 2.75 2.74 2.74 2.74 2.73 2.73 2.72 2.72 2.71 2.23 2.22 2.21 2.20 2.20 2.19 2.18 2.17 2.16 2.09 2.07 2.05 2.04 2.03 2.02 1.86 1.86 1.85 1.80 1.78 1.77 1.76 1.75 1.74 1.73 1.71 1.70 1.69 1.69 1.67 1.67 1.65 1.64 1.63 1.63 1.62 1.30 1.30 1.28 1.27 1.22 1.21 1.05 1.03 1.02
^{1}H NMR (400 MHz, CDCl$_3$)
H$_B$
H$_A$
O
EtO
Me
O
H
E
O
Me
Me
35
0.64 1.16 0.99 1.03 0.90 2.02 1.01 1.05 1.10 2.96 4.83 3.03 2.93 3.06
7.5 7.0 6.5 6.0 5.5 5.0 4.5 4.0 3.5 3.0 2.5 2.0 1.5 1.0 0.5 0
f1 (ppm)

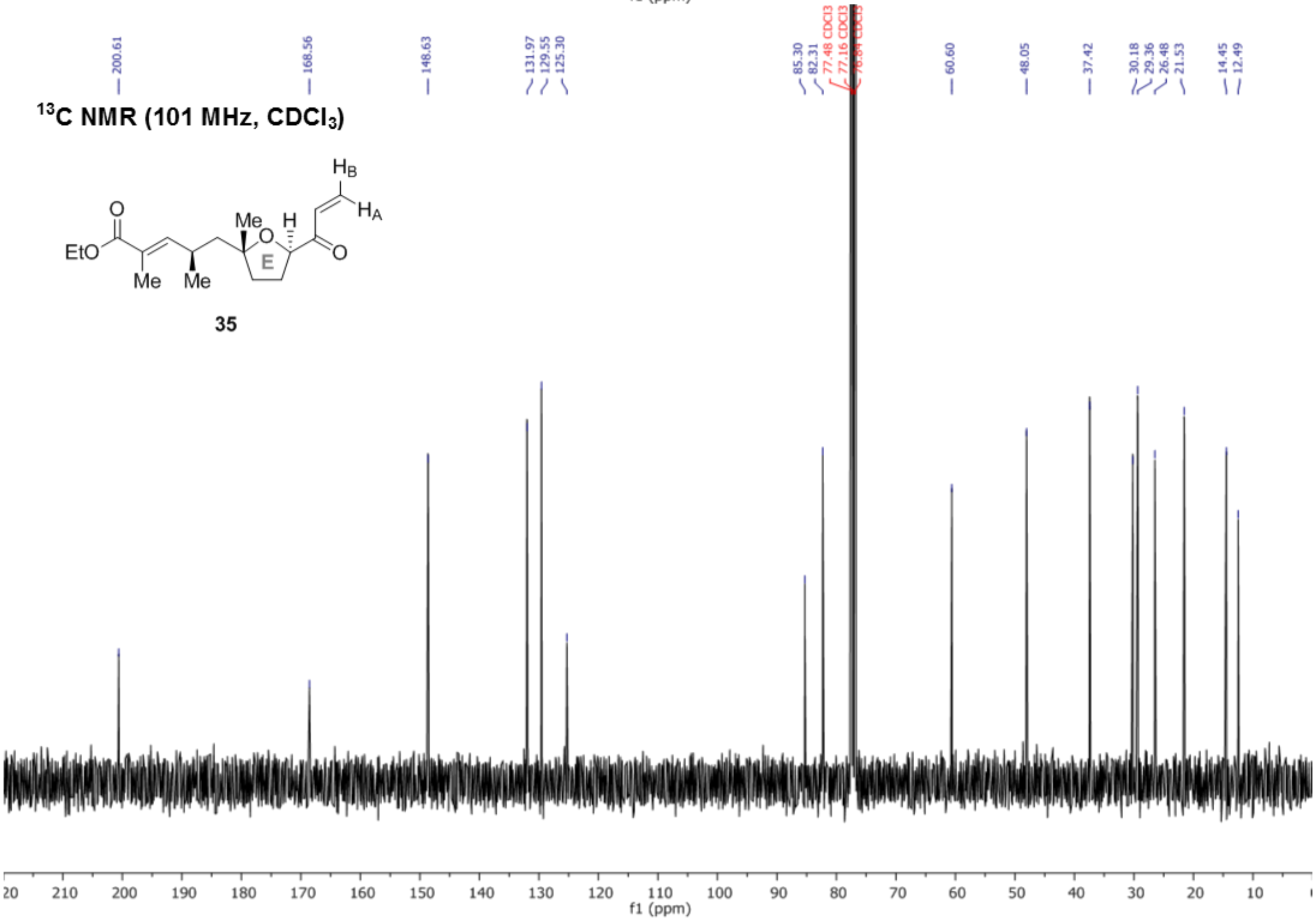
200.61
168.56
148.63
131.97 129.55 125.30
85.30 82.31 77.48 CDCl3 77.16 CDCl3 76.84 CDCl3
60.60
48.05
37.42
30.18 29.36 26.48 21.53
14.45 12.49
^{13}C NMR (101 MHz, CDCl$_3$)
H$_B$
H$_A$
O
EtO
Me
O
H
E
O
Me
Me
35
210 200 190 180 170 160 150 140 130 120 110 100 90 80 70 60 50 40 30 20 10
f1 (ppm)

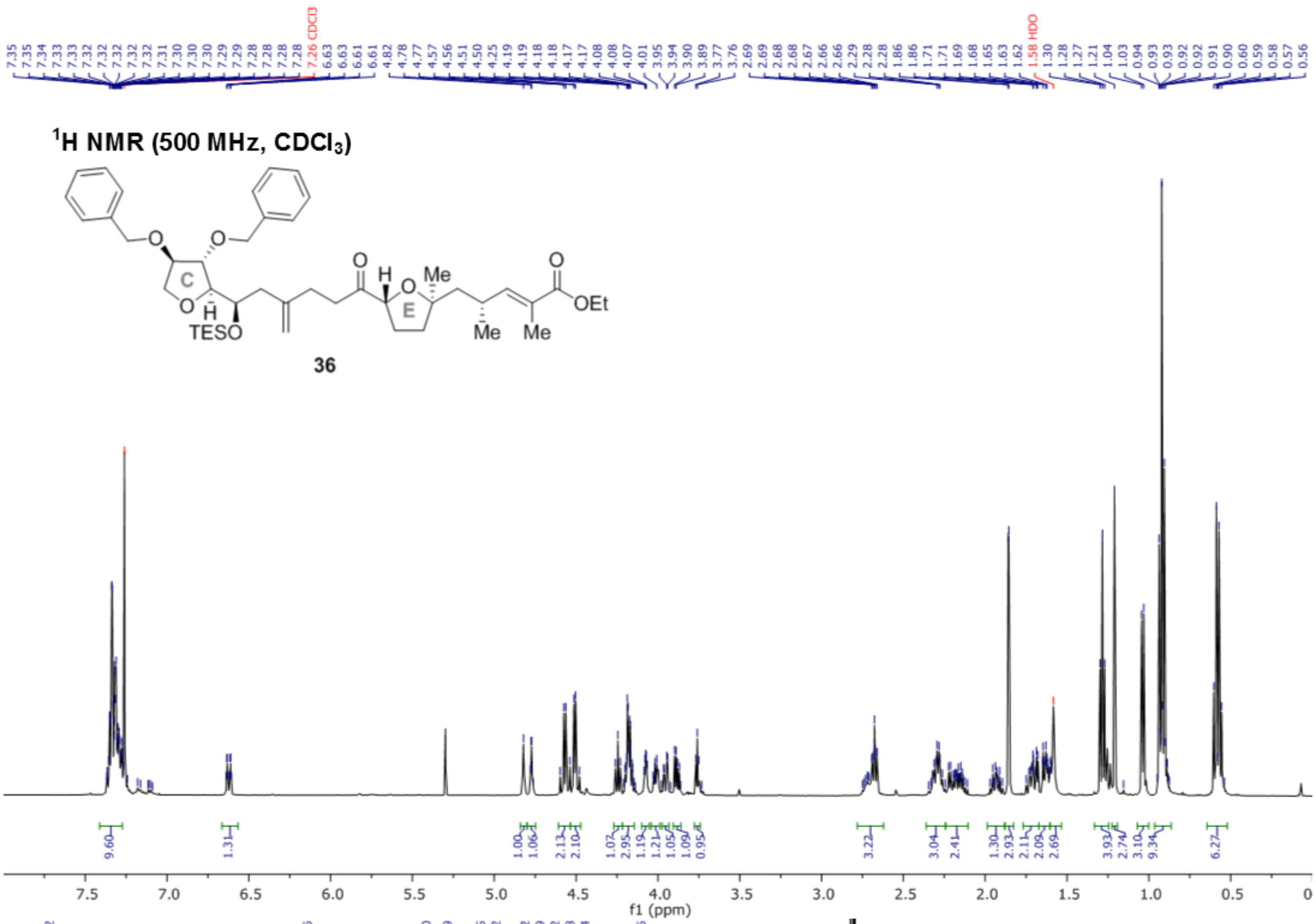

1H NMR (500 MHz, CDCl3)
36
f1 (ppm)

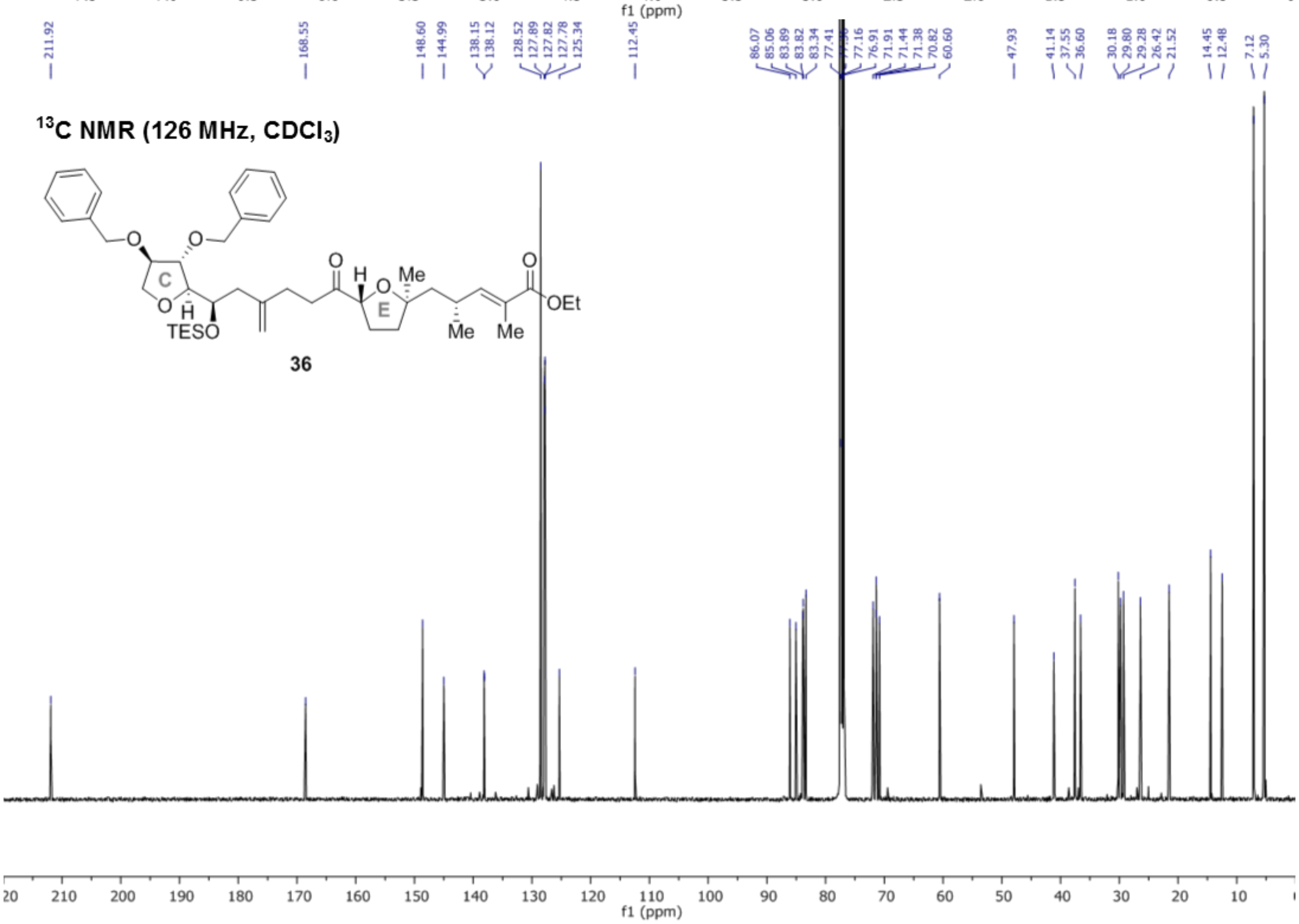

13C NMR (126 MHz, CDCl3)
36
f1 (ppm)

^{1}H NMR (500 MHz, $CDCl_3$)

37

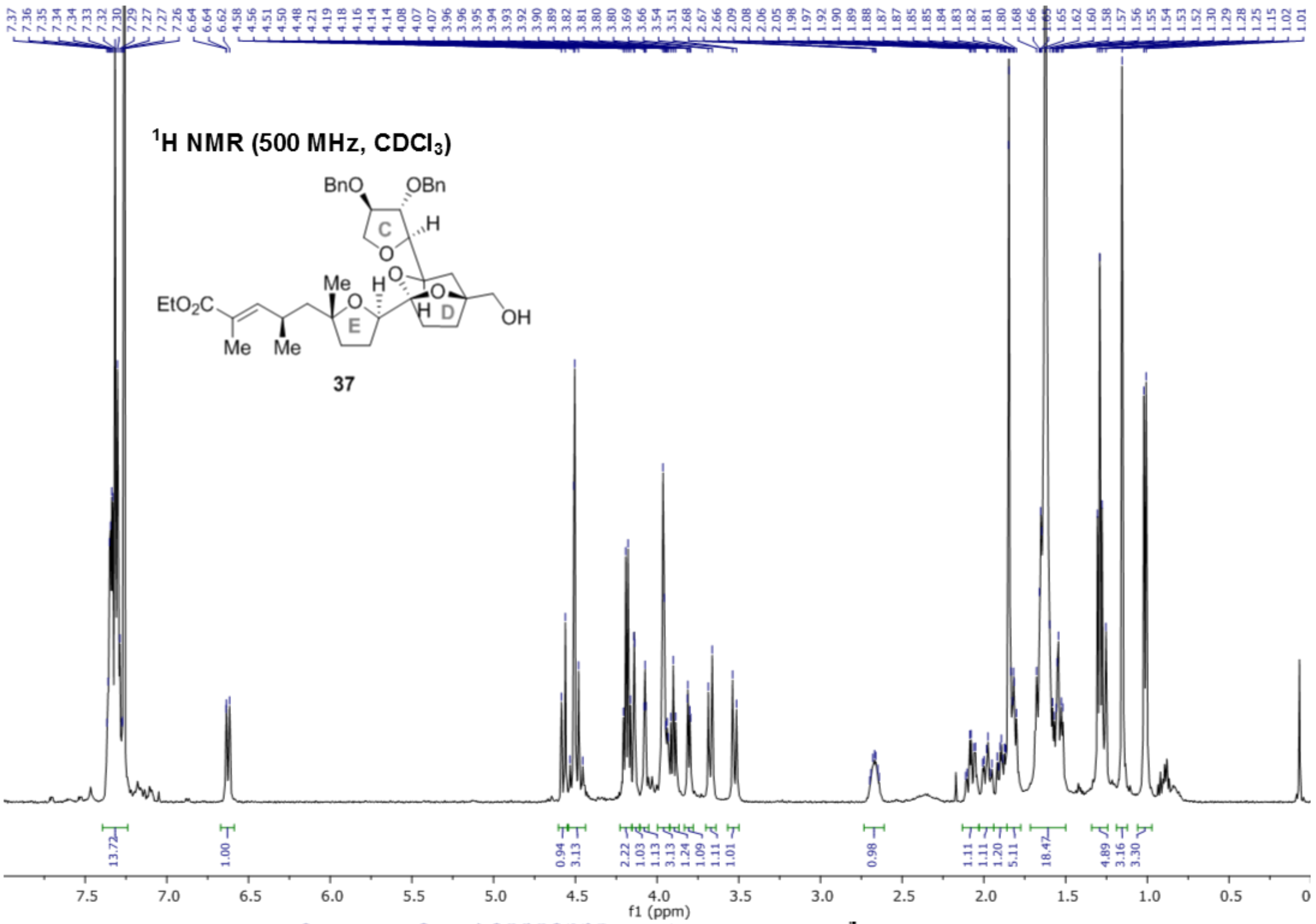

^{13}C NMR (126 MHz, $CDCl_3$)

37

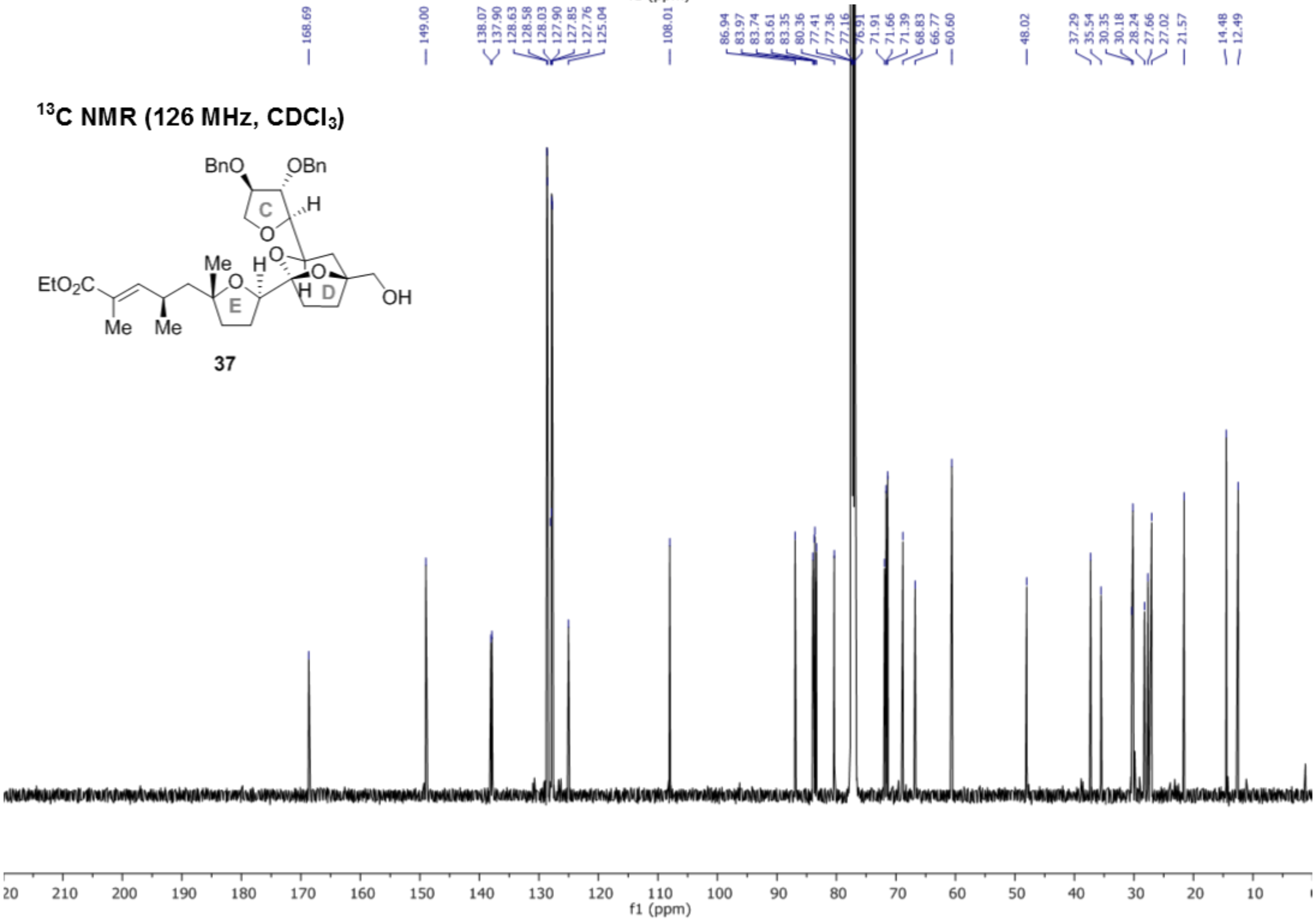

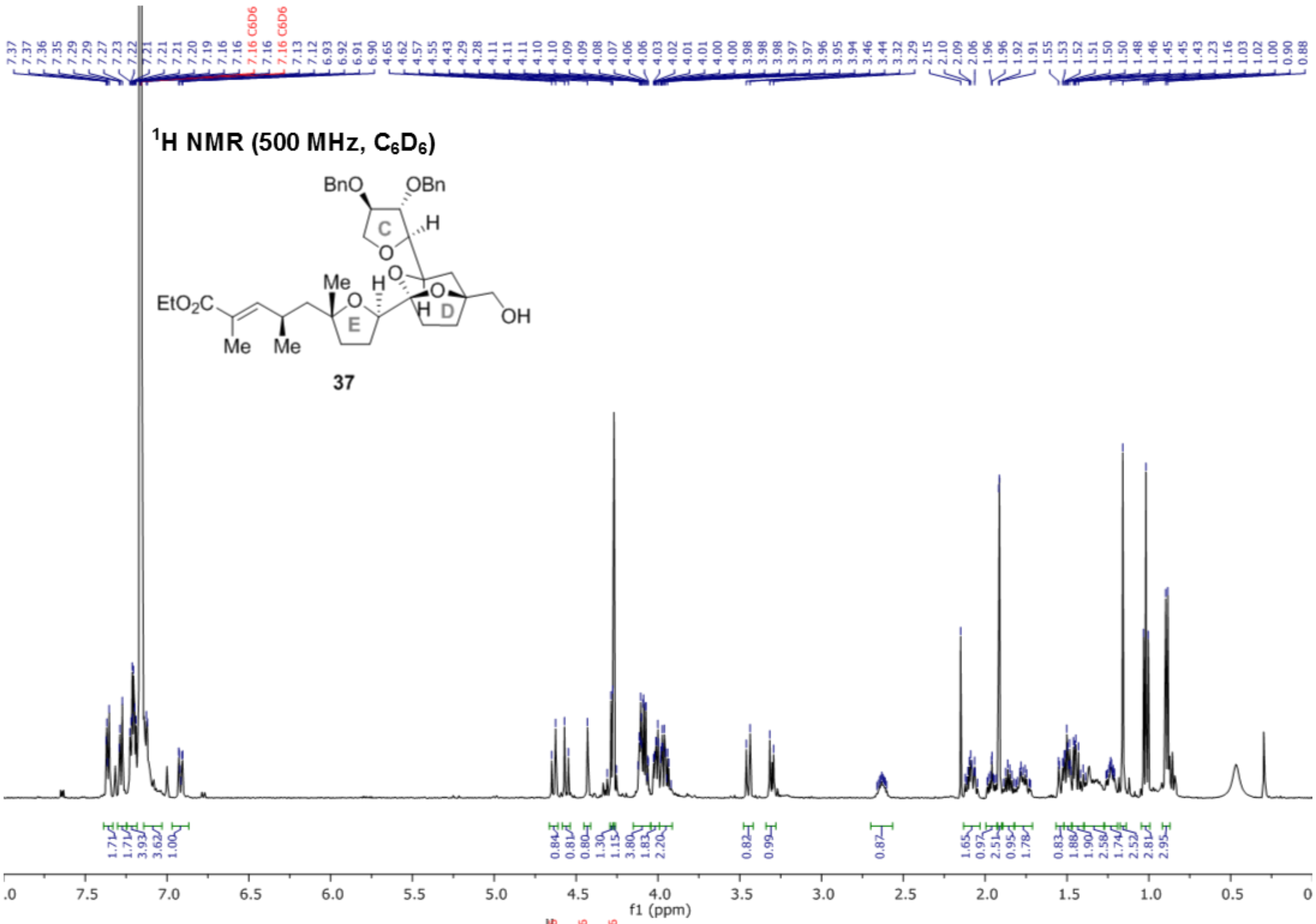
1H NMR (500 MHz, C6D6)
37
f1 (ppm)

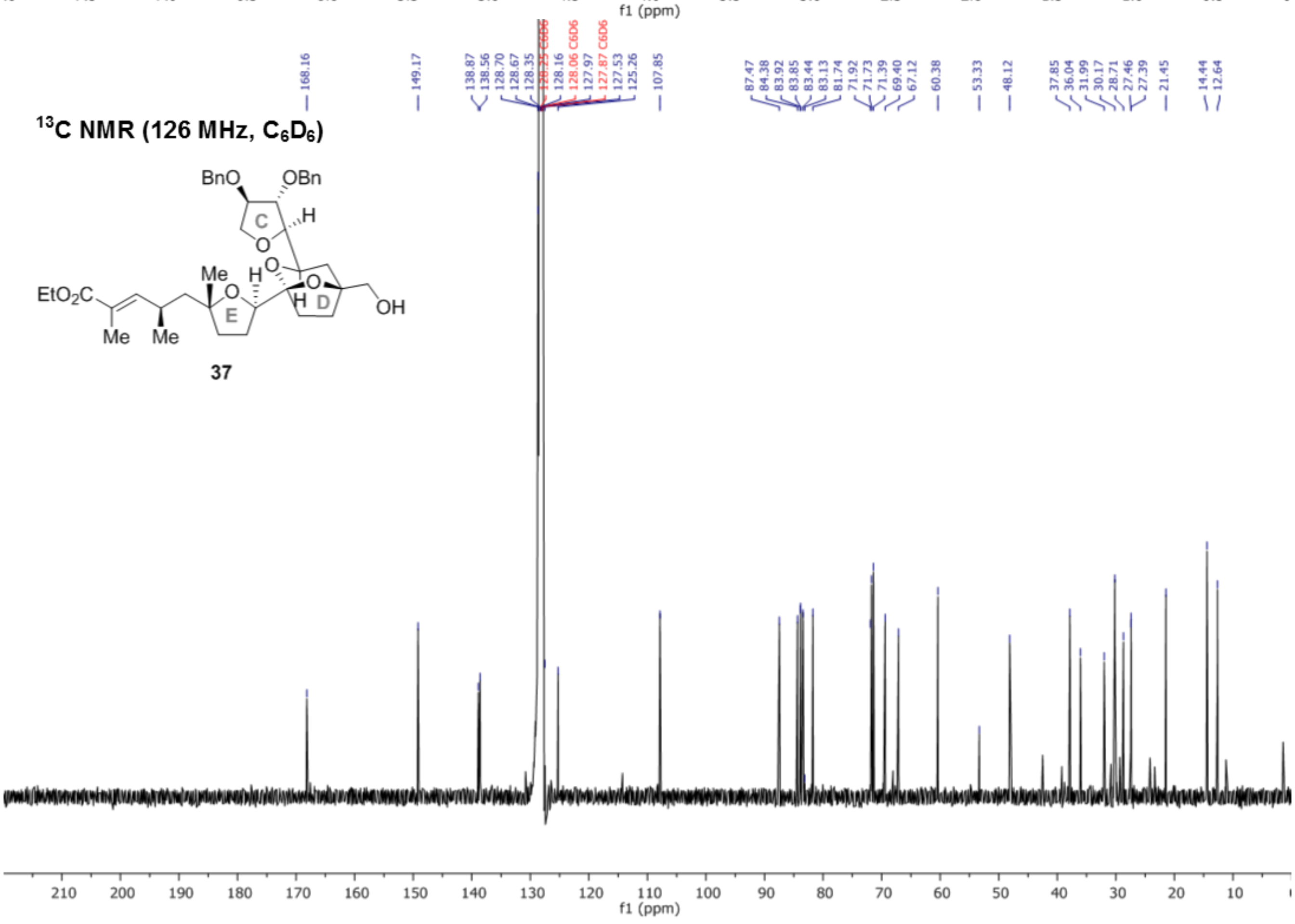
13C NMR (126 MHz, C6D6)
37
f1 (ppm)

Mosher's Ester analysis of 13[7]

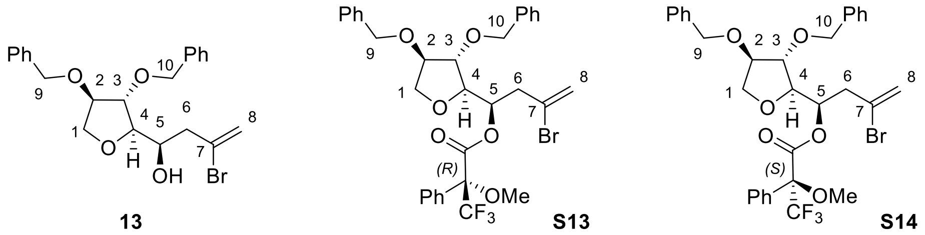

Proton	δS (**S14**) ppm*	δR (**S13**) ppm*	$\Delta\delta^{SR}$ (δS – δR) ppm	$\Delta\delta^{SR}$ × 400 (Hz)
C-8HB	5.44	5.23	+0.21	+84
C-8HA	5.56	5.36	+0.20	+80
C-6HA	2.89	2.77	+0.12	+48
C-6HB	2.78	2.70	+0.08	+32
C-1HB	3.88	3.90	−0.02	−8
C-5H	5.68	5.71	−0.03	−12
C-4H	3.95	3.98	−0.03	−12
C-3H	4.03	4.06	−0.03	−12
C-2H	3.95	4.02	−0.07	−28
C-1HA	3.95	4.06	−0.11	−44

^{1}H NMR (400 MHz, $CDCl_3$) shifts.

* For chemical shifts quoted as a multiplet, the midpoint of the range has been taken.

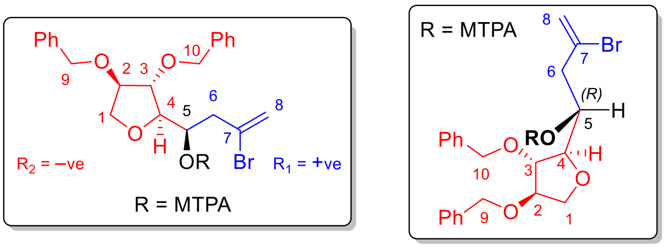

Based upon the model proposed by Mosher, alcohol **13** was assigned the (*R*)-configuration at C-5.

nOe analyses

Analysis of 27

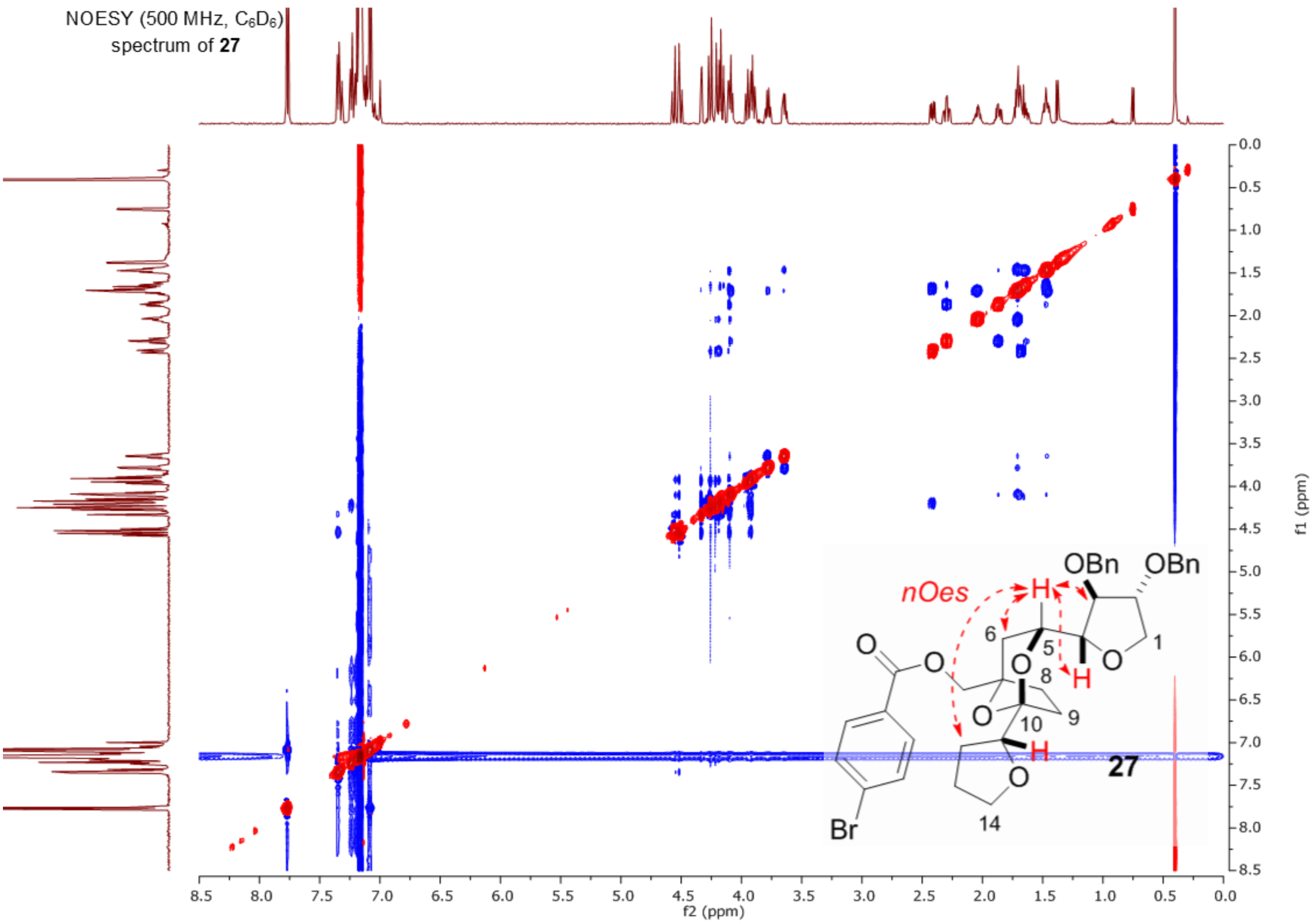

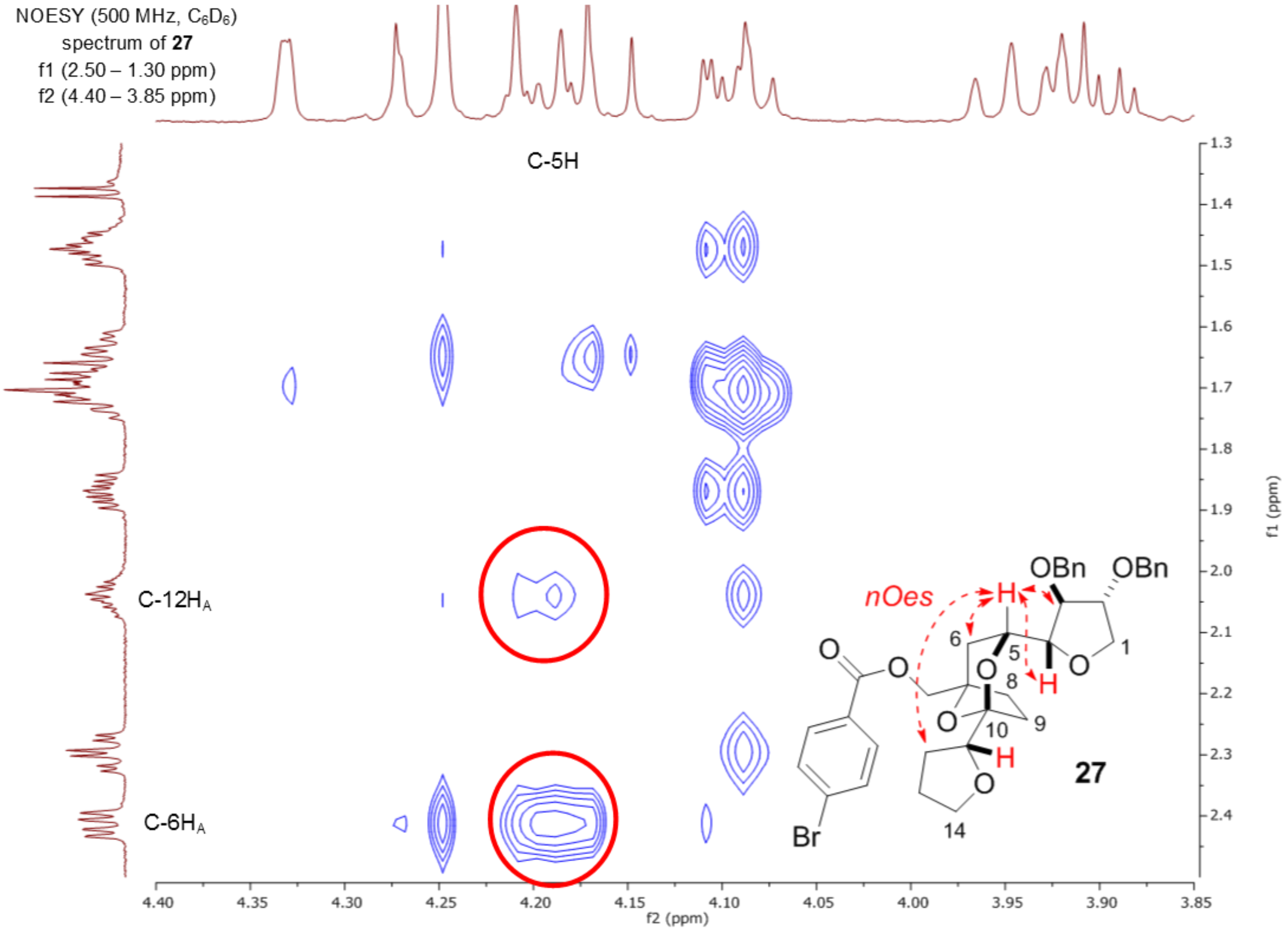

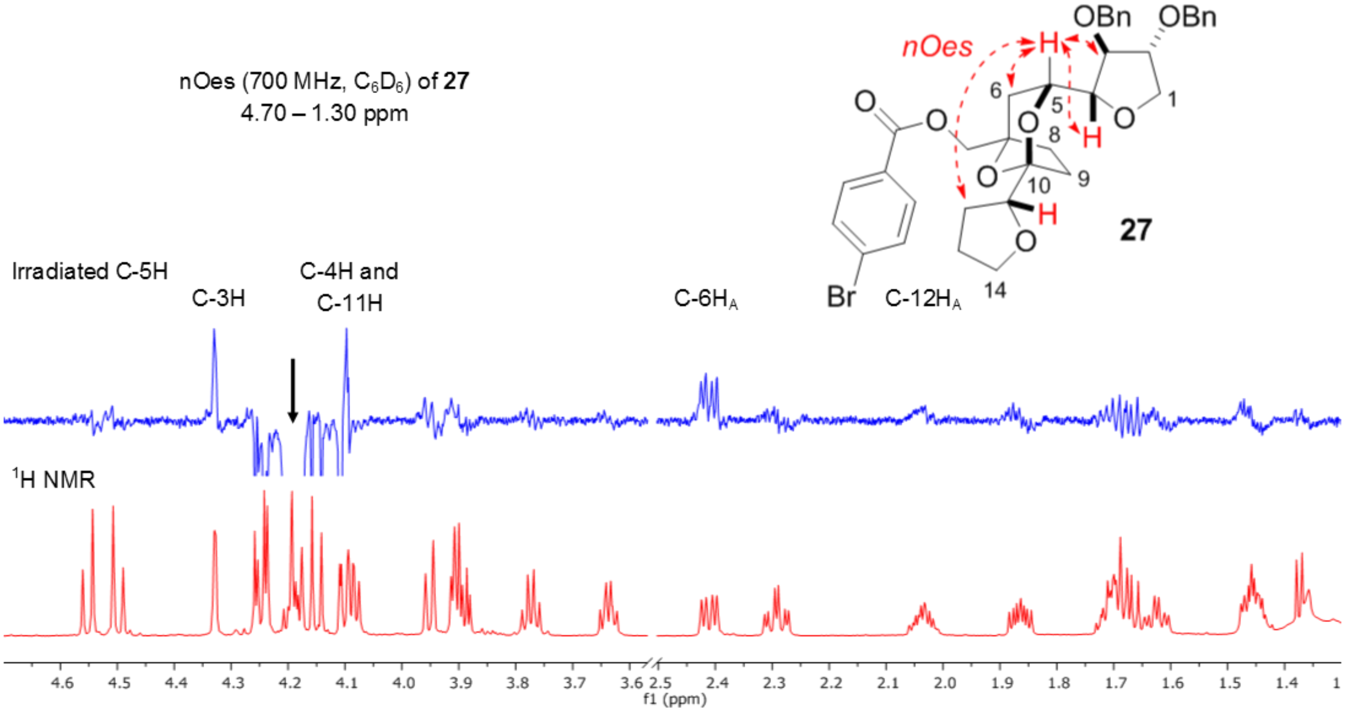

nOes observed: C-5H to C-3H, C-4H, C-6H_A, C-11H and C-12H_A. No observed nOe to C-8$H_{A/B}$ or C-9$H_{A/B}$.

Analysis of 23

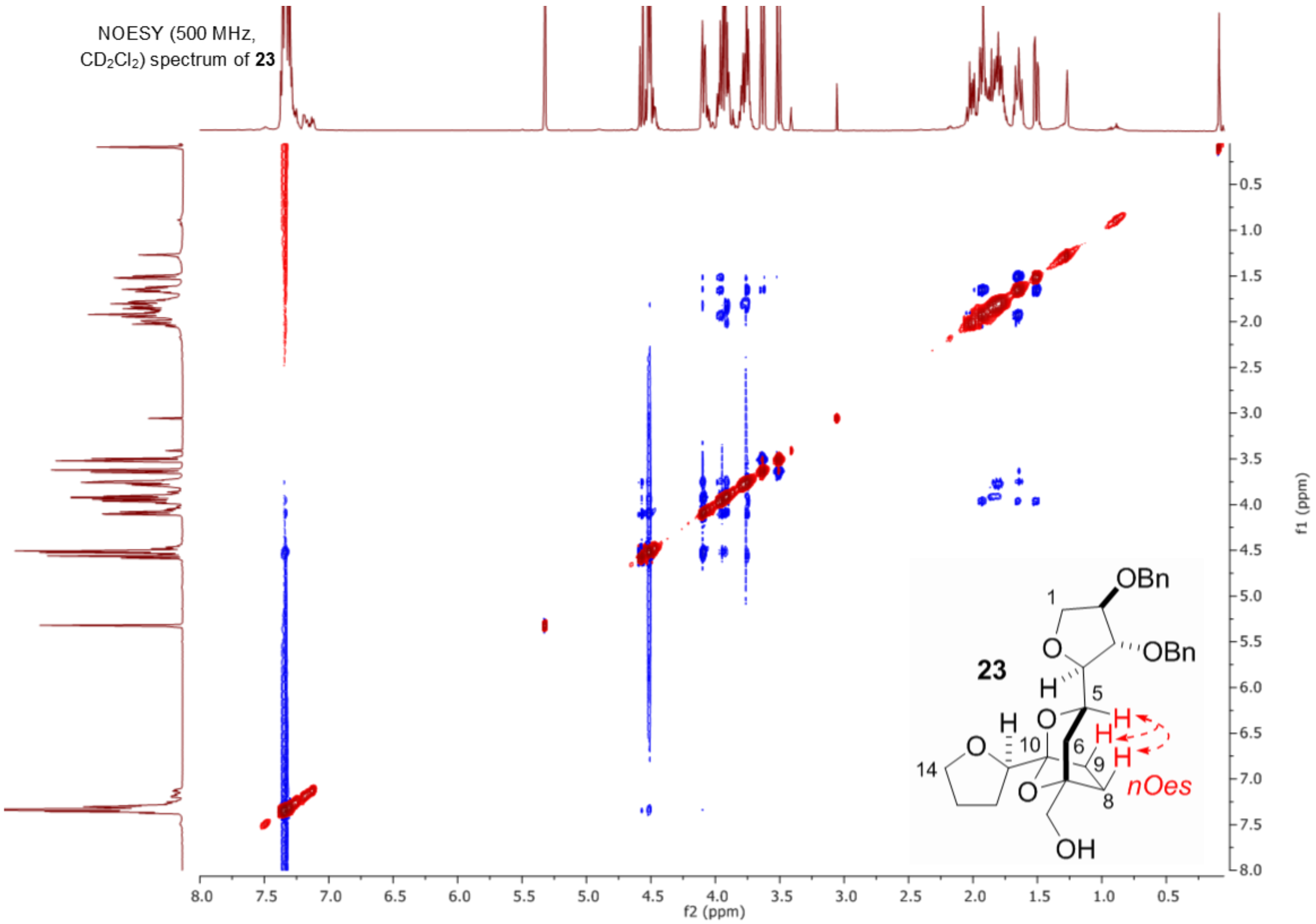

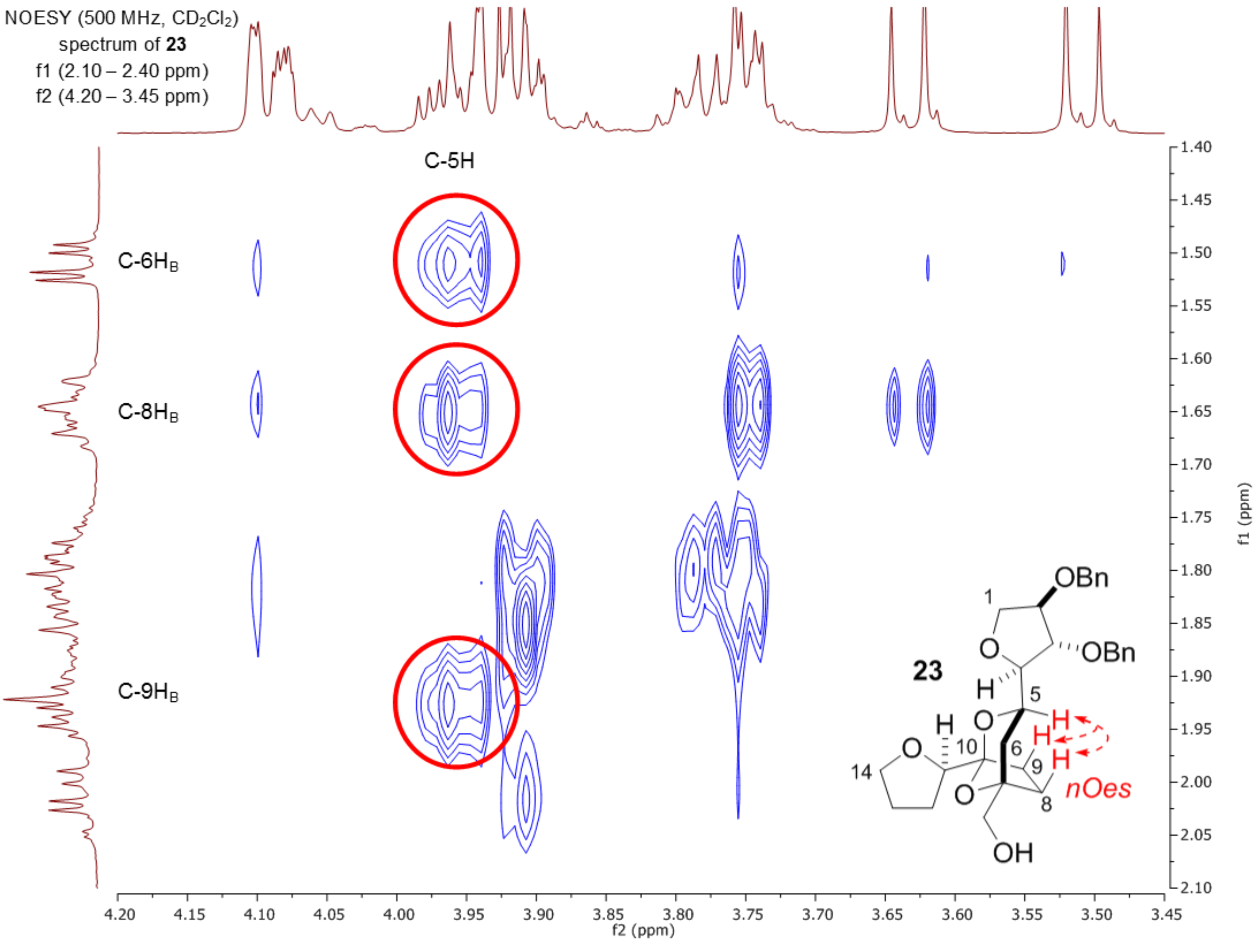

Key nOes observed: C-5H to C-8H$_B$ and C-9H$_B$. Also observe nOes to C-3H, C-4H (not shown) and C-6H$_B$.

Analysis of 28

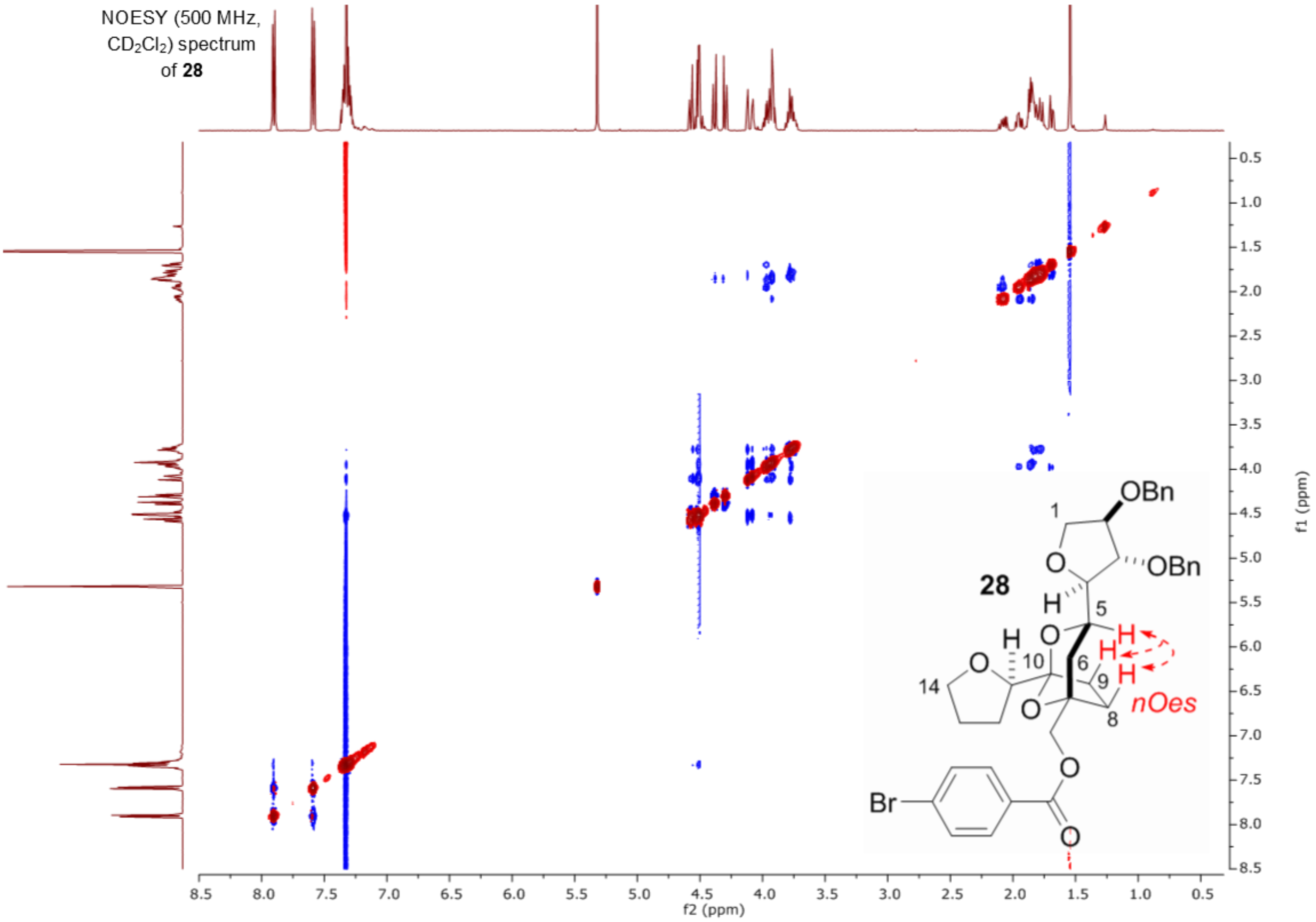

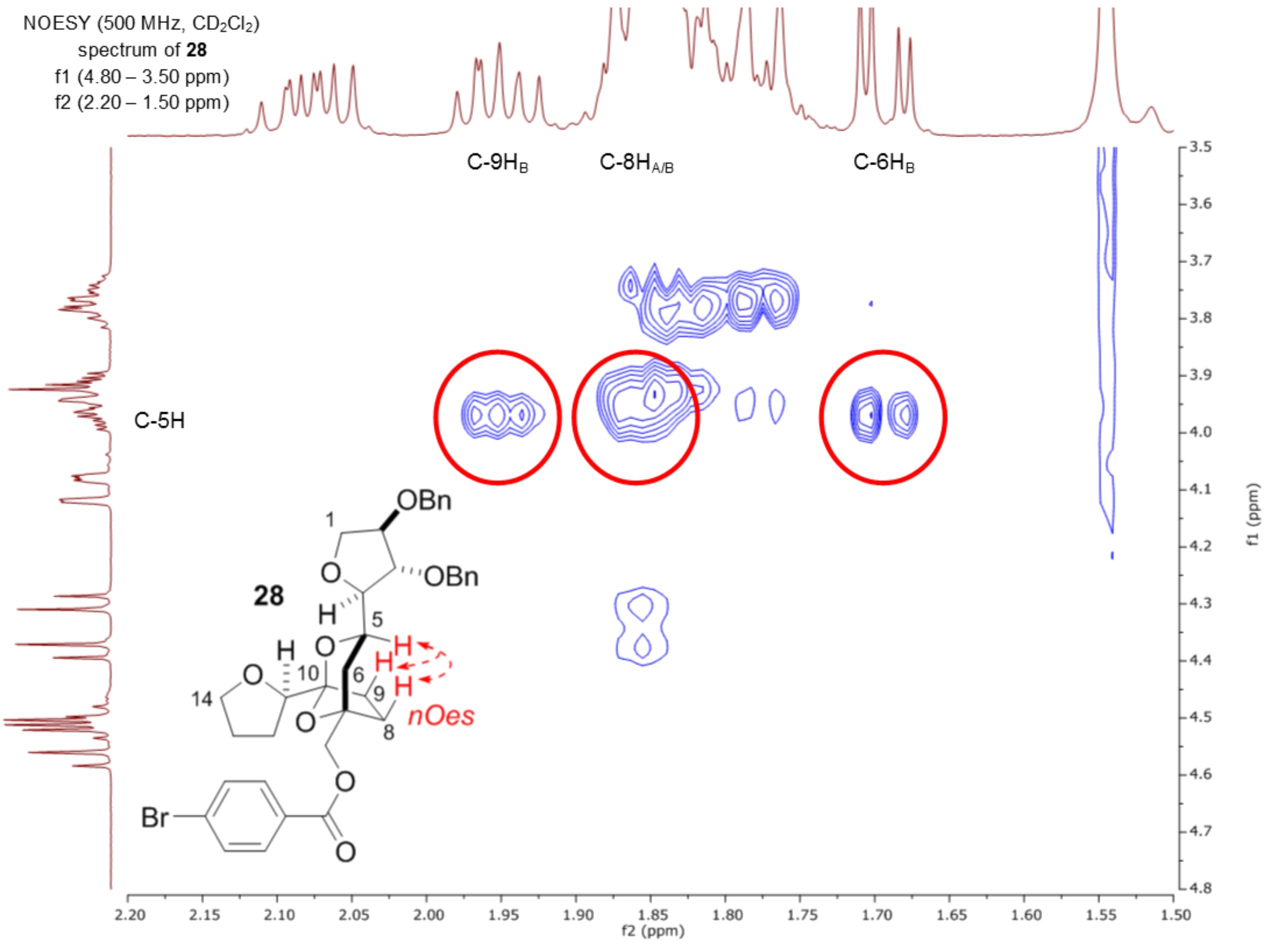

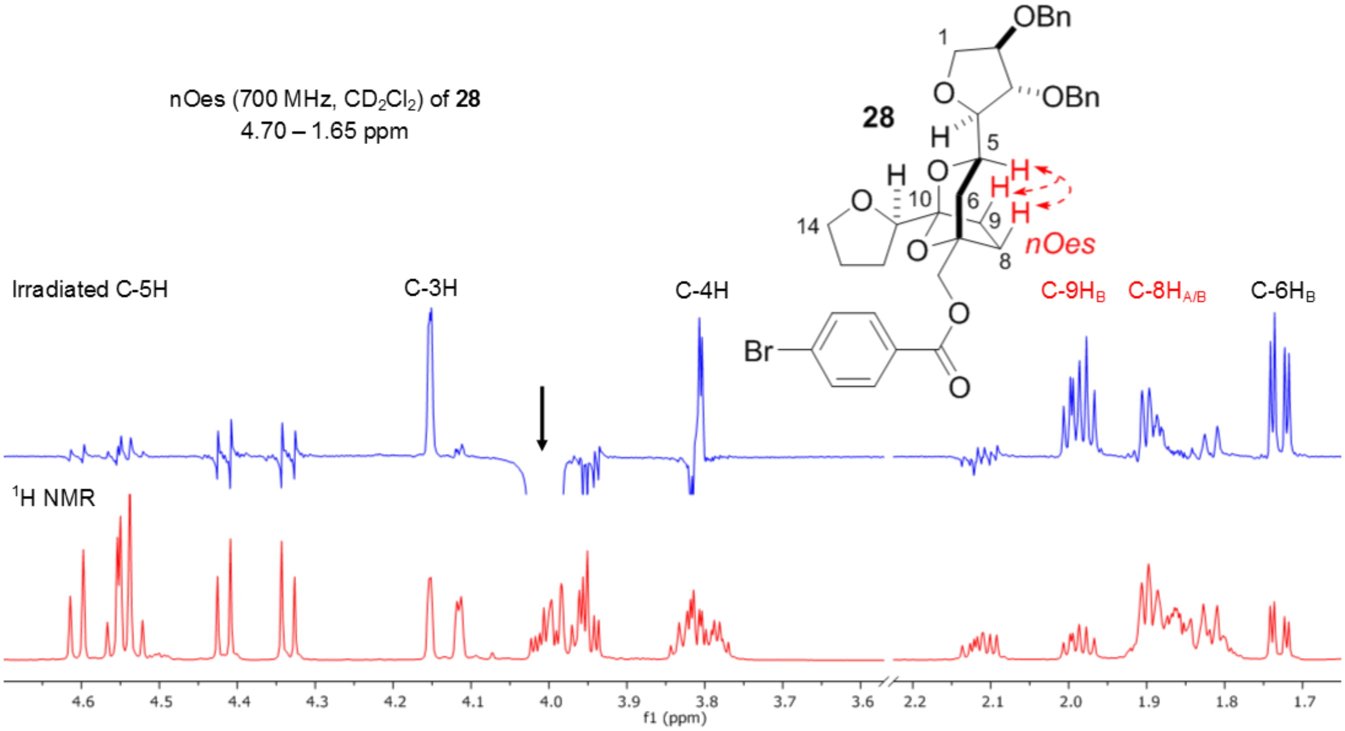

Key nOes observed: C-5H to C-8$H_{A/B}$ and C-9H_B. Also observe nOes to C-3H, C-4H and C-6H_B.

Analysis of 33

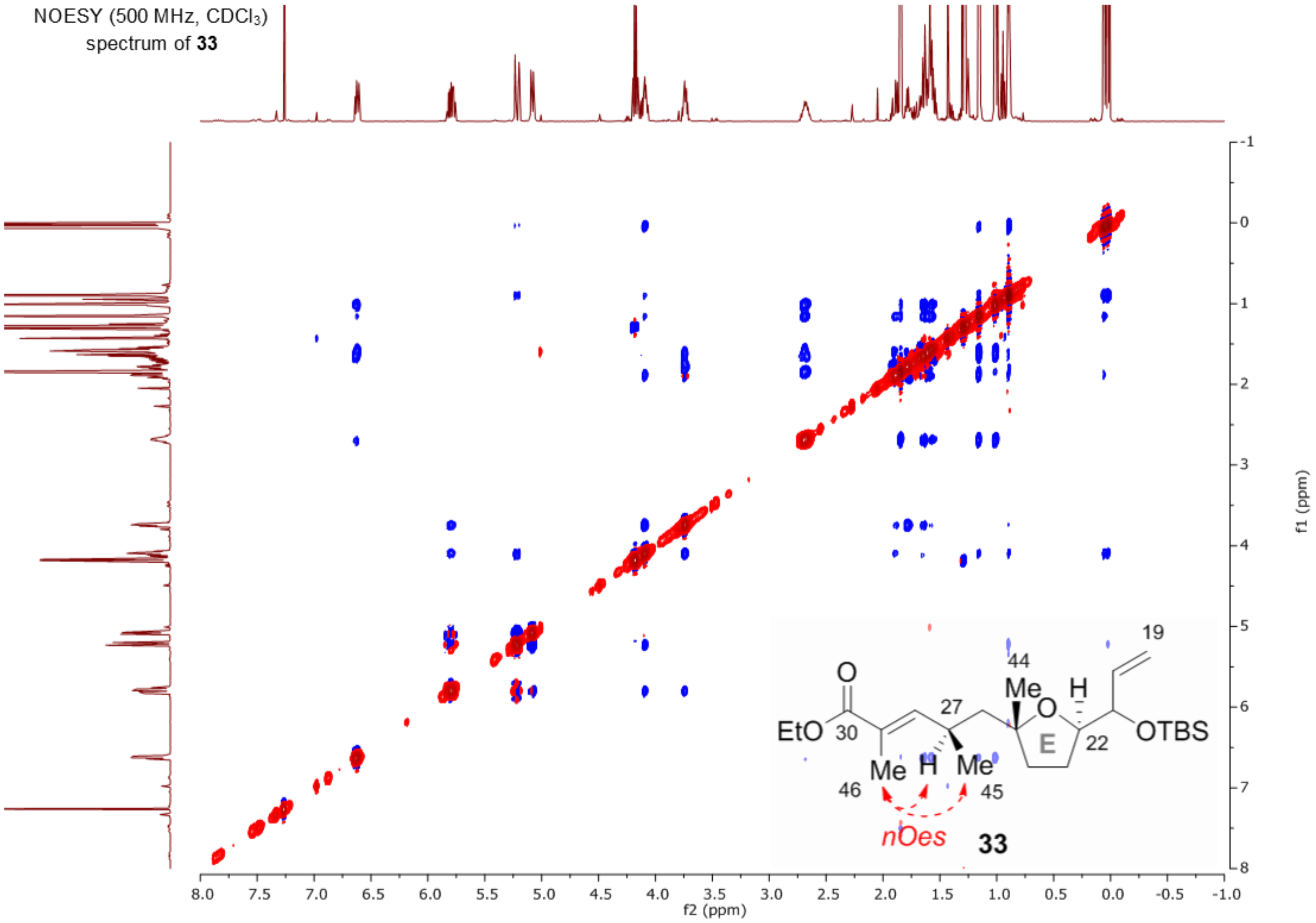

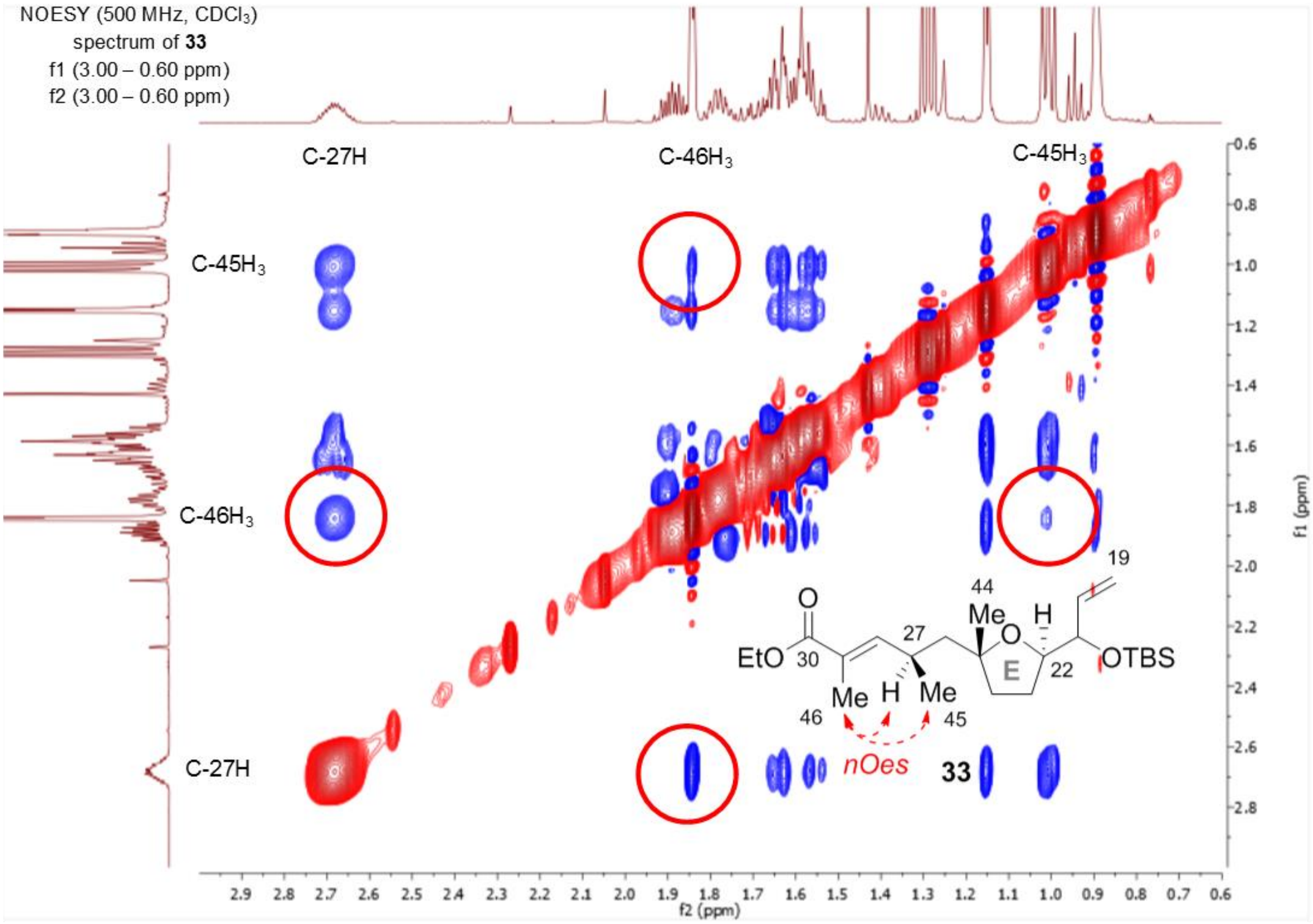

Key nOes observed: C-46H$_3$ to C-27H and C-45H$_3$, therefore (*E*) double bond geometry.

Analysis of 37

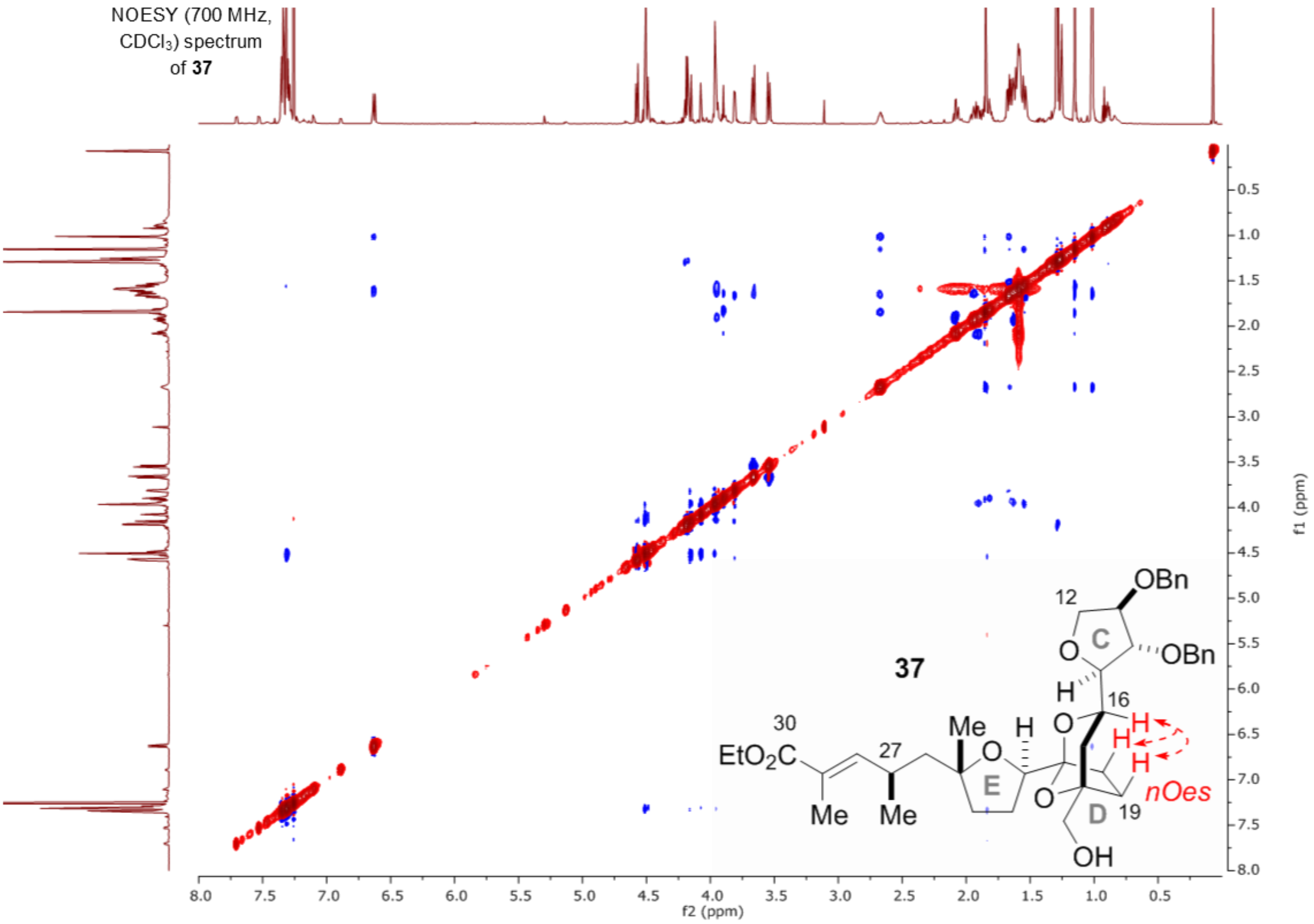

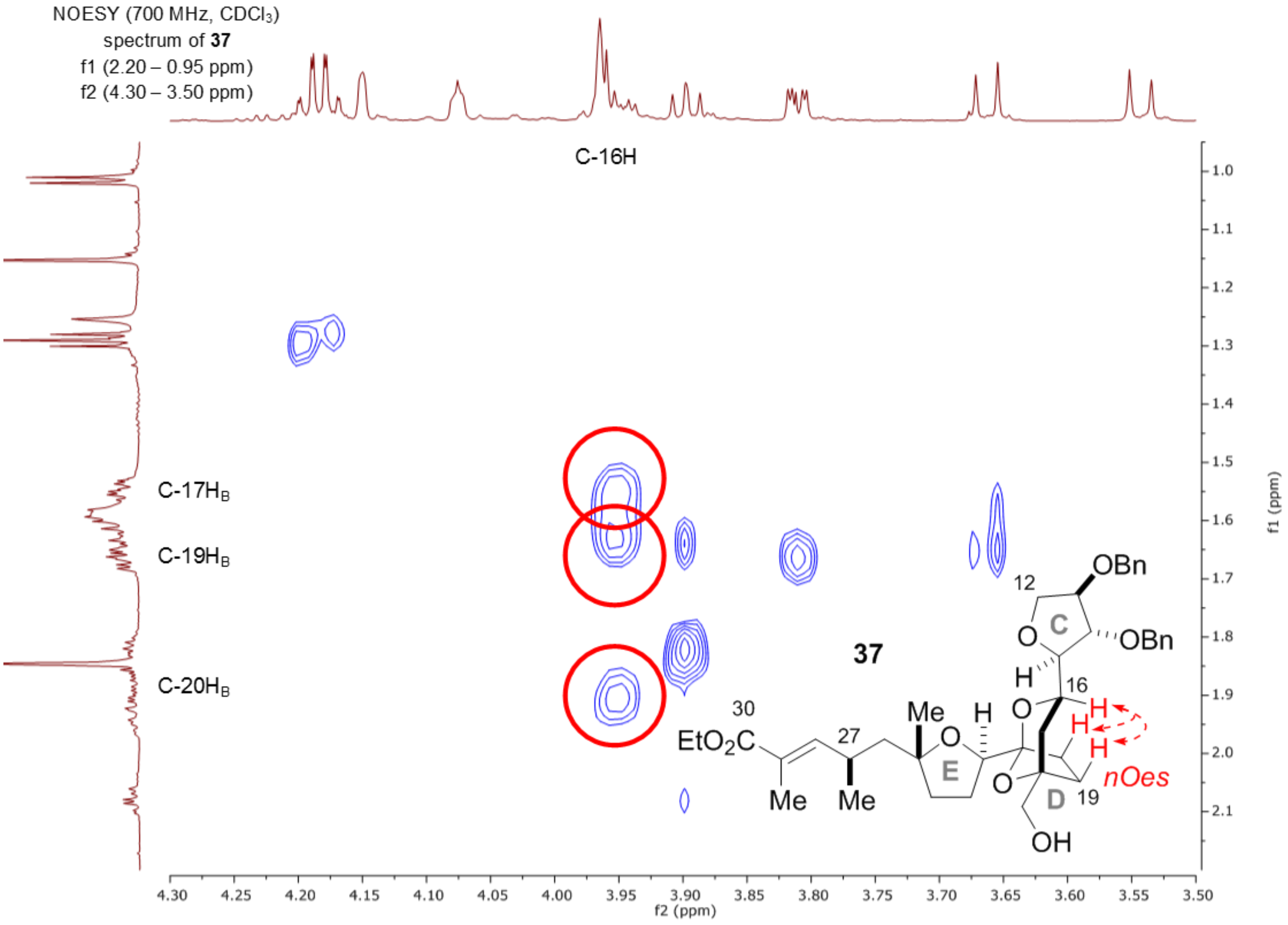

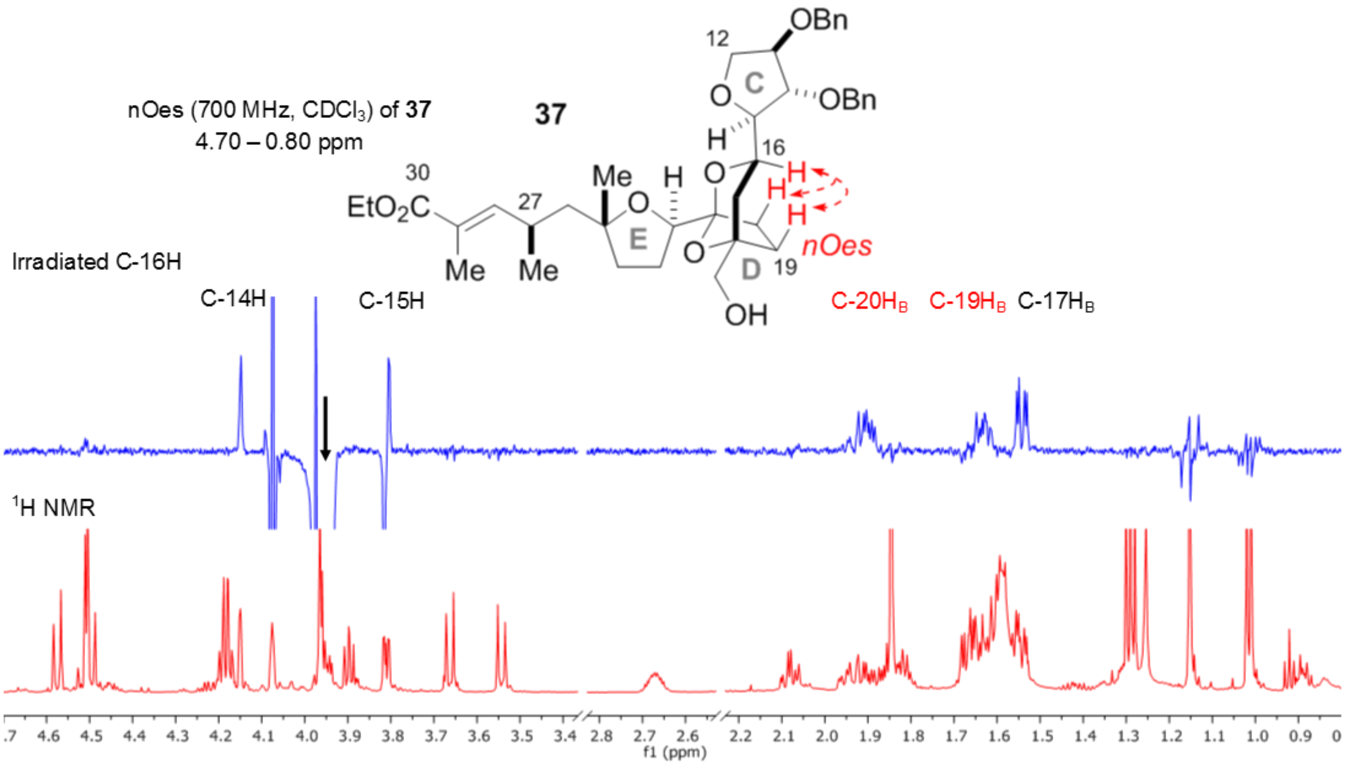

Key nOes observed: C-16H to C-19H_B and C-20H_B. Also observe nOes to C-14H, C-15H and C-17H_B.

VI) References

1 N. Kornblum, A. P. Lurie, *J. Am. Chem. Soc.*, 1959, **81**, 2705–2715.

2 T. Yasumoto, M. Murata, Y. Oshima, M. Sano, G. K. Matsumoto, J. Clardy, *Tetrahedron*, 1985, **41**,1019–1025.

3 J. Takagi, K. Takahashi, T. Ishiyama and N. Miyaura, *J. Am. Chem. Soc.*, 2002, **124**, 8001–8006.

4 M. Sakai, H. Hayashi and N. Miyaura, *Organometallics*, 1997, **16**, 4229–4231.

5 F. Nicotra, L. Panza, G. Russo and L. Zucchelli, *J. Org. Chem.*, 1992, **57**, 2154–-2158.

6 B. M. Trost, T. A. Grese and D. M. T. Chan, *J. Am. Chem. Soc.*, 1991, **113**, 7350–7362.

7 a) J. A. Dale and H. S. Mosher, *J. Am. Chem. Soc.*, 1973, **95**, 512–519.
b) I. Ohtani, T. Kusumi, Y. Kashman and H. Kakisawa, *J. Am. Chem. Soc.*, 1991, **113**, 4092–4096.
c) T. R. Hoye, C. S. Jeffrey and F. Shao, *Nat. Protocols*, 2007, **2**, 2451–2458.

8 P. Bianchi, G. Roda, S. Riva, B. Danieli, A. Zabelinskaja-Mackova and H. Griengl, *Tetrahedron*, 2001, **57**, 2213–2220.
